# Pyridine C─N Transposition via Cycloaddition–Cycloreversion

**DOI:** 10.1002/anie.5249878

**Published:** 2026-06-03

**Authors:** Aífe Conboy, Michael F. Greaney

**Affiliations:** ^1^ Department of Chemistry University of Manchester Manchester UK

**Keywords:** ANRORC, cycloaddition–cycloreversion, pyridine, skeletal‐editing, transposition

## Abstract

The selective incorporation of nitrogen atoms is fundamental to the design and synthesis of drugs and other functional molecules. Here, we describe a skeletal‐editing approach to the transposition of pyridine nitrogen atoms, creating a route to 3‐alkylated pyridines that are difficult to access using current methods. The reaction uses a cycloaddition/cycloreversion strategy (CACR) to transpose easily made 4‐aryl and alkyl pyridines into the more challenging *meta*‐functionalized isomers. The cycloaddition reaction introduces versatile sulfone functionality, that can be harnessed for further C─X and C─C bond formations for pyridine synthesis.

## Graphical abstract

Transposition of pyridine nitrogen from the 4‐ to the 3‐position is described using two open and shut sequences: a nucleophile addition ring‐open ring‐closure, followed by a nitrile Diels–Alder cycloaddition cycloreversion. The nitrogen swap is particularly effective for tertiary alkyl substituents, transforming easily accessible *para*‐alkylated pyridines into the far more challenging *meta* isomers.

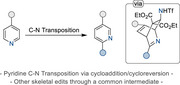

## 1 Introduction

**FIGURE 1:**
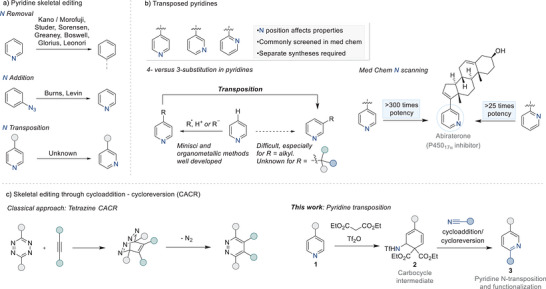
(a) Principal modes of skeletal editing for pyridines. (b) Transposed pyridines [43]. (c) This work: skeletal editing transformations of pyridines through cycloaddition/cycloreversion (CACR) reactions of a carbocycle formed through a pyridine ANRORC reaction.

The introduction, exchange, and transposition of nitrogen atoms in biologically active molecules can critically affect key physical properties such as target binding, solubility, and stability. Skeletal editing has emerged in recent years as a vehicle for achieving such transformations, whilst avoiding the laborious task of repetitive resynthesis [1, 2]. Recent years have seen a surge of interest in such transformations, with particular focus on skeletal editing strategies that facilitate interconversions of arenes and heteroarenes, due to the privileged status of such motifs across chemical disciplines. Skeletal editing reactions can loosely be categorized into three main types: atom insertion [3, 4, 5, 6, 7], deletion [8, 9, 10, 11, 12, 13, 14], and exchange [15, 16, 17, 18, 19, 20, 21, 22, 23, 24, 25, 26, 27, 28, 29], with many examples of each reported in the literature to date. Amongst the exchange skeletal edits, nitrogen transposition strategies are particularly valuable due to their potential in medicinal chemistry (Figure 1b), but they remain under‐developed due to the inherent challenge of swapping N atoms in a given heterocycle. Notable work from the Leonori and Sarpong laboratories has recently described photochemical N‐transposition in 5‐membered heterocycles such as thiazoles, oxazoles and indazoles [20, 21, 22]. Transposition in pyridines, however, has yet to be reported.

We were drawn to the idea of transposing pyridine nitrogen from the 4‐ to the 3‐position. An implicit strength of skeletal editing is its potential to leverage structures that are highly tractable to synthesis, and convert them to difficult targets that lack synthetic expediency. For pyridines, C─C bond formation at the 2‐ and 4‐ positions is readily addressed through radical Minisci methods and anionic organometallic additions, due to the innate reactivity of the heteroarene. *meta* C─C bond formation, however, is far more challenging with 3‐alkylated pyridines in particular being very challenging to synthesize, resulting in commercial scarcity and expense (Figure 1b) [30]. Current approaches to this problem include *meta*‐halogenation, which introduces a functional handle for incipient C─C bond formation, with several innovative approaches recently being disclosed [31, 32, 33, 34]. Wang and coworkers have described a direct approach for primary alkylation, using aldehydes in a boron‐mediated dearomatization reaction [35, 36]. A transposition approach could offer several advantages, especially with respect to hindered tertiary alkyl groups. There are no current methods for direct tertiary alkylation of pyridines, and catalytic cross‐coupling of such sterically hindered groups onto halo‐pyridines is frequently low‐yielding [37].

## 2 Results and Discussion

Our approach to N‐transposition began from our recent observations on pyridine nucleophilic addition ring‐opening ring‐closing (ANRORC) processes using malonate nucleophiles [16]. This reaction (Figure 1c) produces the carbocyclic intermediate **2** in excellent yields from 4‐substituted pyridines, through simple N‐triflation and addition of diethyl malonate. We envisioned that this carbocyclic diene structure could provide a route to N‐transposition, amongst other skeletal‐editing transformations, using cycloaddition–cycloreversion (CACR) chemistry. CACR is a powerful tool for skeletal editing that encapsulates the concept of switching atom pairs in and out of heteroarene structures, illustrated by the classic tetrazine inverse electron demand Diels–Alder reaction, followed by cyclo‐reversion with ejection of nitrogen (Figure 1c) [38]. CACR methods have featured in several recent skeletal editing approaches that exploit the reagent‐less conditions of pericyclic reactions (simple heating) to achieve atom‐swaps on heteroarenes [15, 18, 39, 40, 41, 42]. It is possible that cycloaddition of **2** with nitrile derivatives could create a pathway to transposed pyridines, in addition to other editing possibilities, through ejection of a C─C unit as an alkene derivative.

**SCHEME 1:**
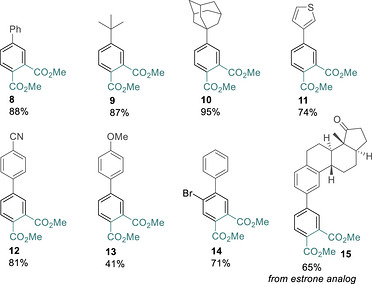
Scope of pyridine to benzene transformation through CACR. Reactions were performed on a 0.2 mmol scale. Isolated yields from the carbocycle. Average yield of ANRORC reaction = 80% (See Supporting Information for conditions).

We began work with a proof of principle study to establish the viability of CACR chemistry with **2**, in particular the cycloreversion step. Classic CACR strategies typically leverage reactive π‐components that can set up an aromatization event by losing a two‐atom unit as a good leaving group (e.g. N_2_ or CN). Here, we require **2** to fragment with loss of a two carbon enamine derivative, a less good leaving group and something not previously described in the literature. Our initial studies began by investigating the reaction of carbocycle **4** – formed through an ANRORC reaction of 4‐phenyl pyridine with diethyl malonate—with the alkyne dienophile DMAD (Table 1). Pleasingly, on heating to 100°C in a 1 M solution of toluene we could isolate the desired benzene product **6** in modest yield (Table 1, entry 2), indicating that the CACR approach was viable. Increasing the temperature and dienophile equivalents lead to a much‐improved yield of 89% (Table 1, entry 5). We did not observe the intermediate adduct **5** in the reaction, indicating that cycloreversion is fast under the reaction conditions. We could, however, observe enamine byproduct **7** in the crude ^1^H NMR spectrum of the reaction mixture, supporting the proposed CACR mechanism. With efficient conditions in hand, we could investigate the scope of this pyridine to benzene transformation (**8**–**15**, Scheme 1). The process proved amenable to a range of 4‐alkyl, aryl, and heteroaryl substituted pyridine substrates, including a biologically active estrone derivative which gave the corresponding benzene product **15** in good yield. The 3‐bromo‐4‐phenyl pyridine substrate was likewise selectively converted into the tetra‐substituted benzene **14** in good yield.

**TABLE 1:** Optimization of pyridine to benzene transformation.

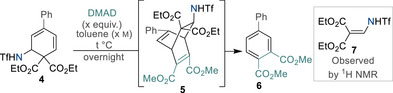						
Entry	Solvent	DMAD equiv.	Temp. (°C)	Conc. (M)	Conv. of 4 (%)^a^	Yield of 6 (%)^a^
1	Toluene	1.5	100	0.1	0	0
2	Toluene	1.5	100	1.0	28	26
3	MeCN	1.5	100	1.0	51	38
4	Toluene	1.5	120	1.0	76	74
5	Toluene	3.0	120	1.0	91	89

Reactions were performed on a 0.2 mmol scale.

^a^ Determined by ^1^H NMR using mesitylene as an internal standard.

**SCHEME 2:**
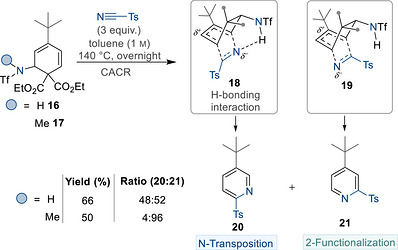
Initial observations for pyridine C─N transposition.

Having established the viability of the CACR approach to editing carbocycle **2**, we turned our attention to nitrile dienophiles and transposition (Scheme 2). Using tosyl cyanide as the CACR dienophile, and *tert*‐butyl substituted carbocycle **16** diene, we were delighted to observe successful reaction to give the corresponding pyridine products **20** and **21**. As with the previous benzene synthesis, the intermediate Diels–Alder adduct was not observed in the reaction. Both regioisomers were formed in an approximately 1:1 ratio, separable by column chromatography—regioisomer **20** represents the pyridine N‐transposition product, whilst regioisomer **21** represents 2‐tosylation of the pyridine substrate through formal C─H functionalization.

To understand the factors controlling hetero Diels–Alder selectivity, we initially examined the role of the triflamide—known to be an excellent H‐bond donor. Removing the possibility of H‐bonding via N‐methylation (**17**) gave the 2‐functionalized product **21** almost exclusively. This suggests an innate electronic bias for the polarized transition state **19**, with partial positive charge stabilized by the *t*‐butyl substituent. A triflamide H‐bonding interaction to the cyanide dienophile would counteract this selectivity, stabilizing the transposition selectivity through arrangement **18**. This was further supported by the observation that selectivity for the N‐transposition product **20** is highest in toluene, with the 2‐functionalization regioisomer **21** favored in more polar solvents, which could disrupt this putative hydrogen bonding interaction (see Supporting Information for details).

**SCHEME 3:**
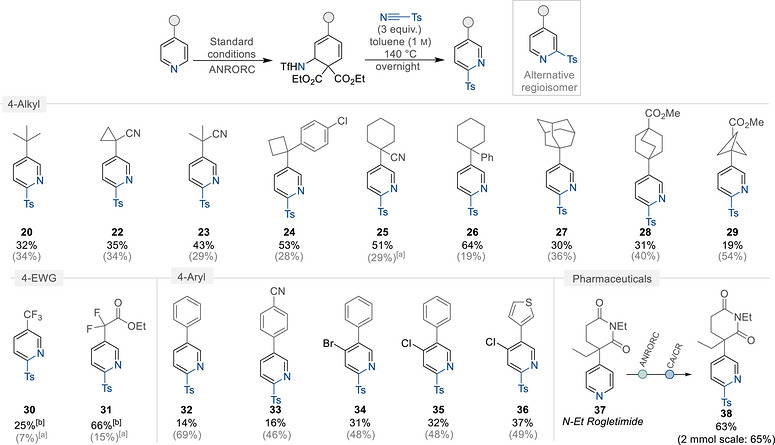
Scope of C─N transposition. Reactions were performed on a 0.2 mmol scale. Isolated yields of the transposed pyridine from the carbocycle. Isolated yields of the alternative regioisomer are in parenthesis. Average yield of ANRORC reaction = 80% (See Supporting Information for conditions). [a] Yield determined by ^1^H NMR. [b] Reaction time was 72 h.

For the scope of the reaction, we were particularly interested in transposing tertiary alkyl pyridines, given the current lack of methods for installing these hindered groups at the pyridine 3‐position (Scheme 3). A variety of 4‐substituted pyridines were readily synthesized through variants of the Minisci reaction (see Supporting Information), and we were pleased to find the transformation successfully gave the corresponding 3‐alkyl N‐transposition products in synthetically useful yields. The 2‐functionalization products could also be isolated. We noted steric factors influencing Diels–Alder selectivity, with increasing size of the diene alkyl substituent favouring the transposition product. Selectivity improved from 1:1 (**22**) to 3.4:1 (**26**, 64% yield) suggesting unfavourable steric interactions between the sulfonate group on the dienophile and larger tertiary alkyl groups on the diene disfavoring the arrangement **19**. The nitrile‐containing products **22**, **23**, and **25** are useful building blocks for medicinal chemistry, enabling further nitrogenated heterocycles to be built from the cyano functionality. We saw highest selectivity for the N‐transposition Diels–Alder geometry with substrates containing a 4‐electron withdrawing substituent, which electronically destabilizes the retention transition (EWG in place of *t‐*butyl in **19**). The CF_3_ and CF_2_CO_2_Et groups gave an approximately 4:1 preference for the transposition products **30** and **31**, although conversion was slow for these substrates. Aryl substrates, by contrast, were less effective for transposition, favoring the 2‐functionalization product (e.g. 3.5:1 for the phenyl‐substituted product **32**). 3,4‐Disubstiuted pyridines could also be transposed, effectively swapping the position of two substituents relative to nitrogen—transposition of 3‐halo‐4‐aryl substrates gave trisubstituted pyridines **34–36** in 31%–37% yield. Finally, the transformation was applied to an N‐protected analog of the pharmaceutical rogletimide (**37**, an aromatase inhibitor)—giving the N‐transposition product **38** with high selectivity and in good overall yield (63%).

**SCHEME 4:**
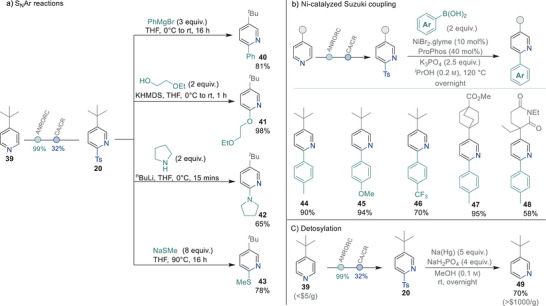
Derivatizations of C─N transposition products. (a) S_N_Ar reactions. (b) Ni‐catalyzed Suzuki coupling. (c) Detosylation. Reactions were performed on a 0.1 mmol scale. Isolated yields after chromatographic purification.

Attempts to expand the scope of the nitrile dienophile showed tosyl cyanide to be uniquely effective in this transformation. Therefore, further derivatization reactions of the resultant tosylated pyridine products were next investigated (Scheme 4). 2‐Tosyl pyridines are known to be effective substrates for S_N_Ar reactions, and underwent displacement of the tosyl group by a variety of nucleophiles, enabling facile C─C, C─O, C─N, and C─S bond formations (**40**–**43**, Scheme 4a). We were also interested in developing a milder means to derivatize these products that could enable useful C─C bond formations. Inspired by some precedent from Niu and coworkers, we were able to develop a nickel‐catalyzed Suzuki coupling using 2‐tosyl pyridines as the electrophilic coupling partner (Scheme 4b) [44]. Our protocol employs NiBr_2_.glyme as an air‐stable precatalyst, thereby avoiding the need for a glovebox. Additionally, the ProPhos ligand recently reported by Diao and coworkers was found to significantly enhance the yield and rate of conversion [45]. High yields were achieved for a variety of pyridine substrates and boronic acid coupling partners (**44**–**48**), and functional group tolerance was also good, with the more complex rogletimide‐derived substrate **37** forming the transposed aryl derivative in 58% yield. We have further demonstrated the ability to remove the tosyl group of pyridine **20** through reduction with sodium amalgam. Overall, this converts 4‐*t*‐butyl pyridine, a cheap and commercially available chemical, into the transposed 3‐isomer that is only accessible through custom synthesis.

**SCHEME 5:**
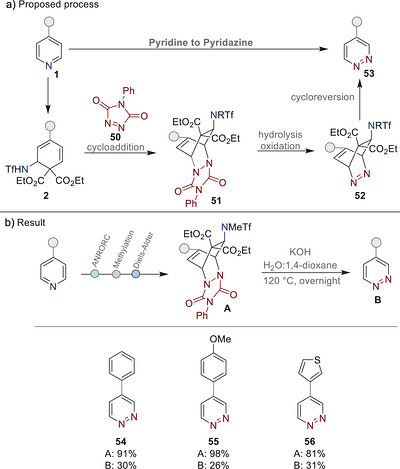
Transformation of pyridines to pyridazines. Reactions were performed on a 0.1 mmol scale. Isolated yields after chromatographic purification. A is the yield of the cycloadduct from the carbocycle. B is the yield of the pyridazine from the cycloadduct.

Our CACR strategy could in principle enable the conversion of pyridines to pyridazines, formally swapping a carbon for a nitrogen atom (Scheme 5a), through reaction with a suitable N═N dienophile. Pyridazines are valued for their physical properties in drug design, but under‐represented in marketed drugs relative to other aza‐heteroarene congeners [46]. This is likely due in part to difficulties in synthesising the pyridazine cores, with synthetic routes being restricted to hydrazine‐based condensation approaches. The Levin, Hong, and Studer groups have recently described novel editing approaches to this transformation, using photo nitrene rearrangement, ANRORC reaction of N‐aminopyridinium salts, and pyridine dearomatization cycloaddition, respectively [24, 25, 47]. To explore our CACR idea, we employed 4‐phenyl‐1,2,4‐triazole‐3,5‐dione (PTAD, **50**) as the dienophile, and found that the initial cycloaddition proceeded smoothly and in high yield with the phenyl‐substituted carbocycle **4**. In contrast to our previous systems, the resultant cycloadduct **51** proved stable and did not undergo cycloreversion under the reaction conditions, likely due to this reaction not having an immediate re‐aromatization pathway as a driving force. We attempted to access diazo intermediate **52** as a cycloreversion substrate for pyridazine formation, via hydrolysis and aerobic oxidation of the cycloadduct **51** (Scheme 5a) [48]. However, we found that upon heating the cycloadduct with KOH, this instead yielded the benzene product formed through nitrogen extrusion and aromatization (See Supporting Information for mechanism of this transformation). Gratifyingly, by methylating the carbocycle **4** to prevent benzene re‐aromatization, we could subject the cycloadduct to simple hydrolytic conditions in the presence of air to give the pyridazine product **54** in 30% yield. The reaction was extended to the 4‐anisyl and (3‐thiophenyl) pyridine substrates (**55** and **56**), demonstrating the viability of this pyridine to pyridazine edit to access 3‐substituted pyridazines as building blocks (Scheme 5b).

## 3 Conclusion

In conclusion, we have developed a nitrogen transposition reaction for pyridines, formally switching the N atom between the 4‐ and 3‐positions for substituted pyridines. The sulfonate group installed from the TsCN dienophile proved versatile, enabling a suite of S_N_Ar C─X bond formations and Ni‐catalyzed arylations. The strategy is particularly effective for leveraging tertiary alkyl groups that are easy to introduce into the 4‐position, and transposing them to the far more challenging 3‐position.

## Author Contributions

**Aífe Conboy**: conceptualization, methodology, investigation, data curation, writing ‐ original draft. **Michael F. Greaney**: conceptualization, supervision, funding acquisition, project administration, resources, writing ‐ original draft.

## Conflicts of Interest

The authors declare no conflicts of interest.

## Supporting information




**Supporting File**: The authors have cited additional references within the Supporting Information [49, 50, 51, 52, 53, 54, 55].

## Data Availability

The data that supports the findings of this study are available in the Supporting Information of this article.
