# Supplementary material for: Pyridine C─N Transposition via Cycloaddition–Cycloreversion
Source: Angew Chem Int Ed Engl. 2026 Jun 3;65(29):e5249878. doi: 10.1002/anie.5249878 (PMC13360665; doi:10.1002/anie.5249878)
Supplement: Supplementary file 1 — Supporting File: The authors have cited additional references within the Supporting Information [49, 50, 51, 52, 53, 54, 55]. [file ANIE-65-e5249878-s001.pdf]

Supplemental experimental procedures

Table of Contents

1 General Remarks 2
2 Pyridine N-Transposition Optimisation 3
2.1 Additive Screen 3
2.2 N-Alkyl Group Screen 3
2.3 Solvent Screen 4
3 Pyridine to Pyridazine Optimisation 5
4 2-Tosyl Pyridine Suzuki Coupling Optimisation 7
5 Limitations 7
6 General Synthetic Procedures 8
7 Synthesis of Substrates 10
8 Synthesis of Carbocycles 15
9 Synthesis of Benzenes via CACR 27
10 Pyridine N-Transposition via CACR 31
11 Derivatisations of 2-Tosyl Pyridines 45
12 Pyridine to Pyridazine Conversion 51
13 References 54
14 NMR Spectra 56

1 General Remarks

All air and/or moisture sensitive reactions were performed under an atmosphere of dry nitrogen using anhydrous solvents and standard Schlenk techniques. The glassware used for such reactions was oven-dried. Reagents and solvents were purchased from commercial sources and used as supplied unless otherwise noted. Small amounts of liquids were handled using 50, 100 and 250 µL Hamilton® 700 Series, 800 Series and GASTIGHT PTFE Luer-lock 1700 Series microsyringes, fit with Luer-lock needles. Thin layer chromatography (TLC) was carried out using aluminium TLC plates coated with Silica gel 60 F254 from Sigma-Aldrich®, and spots were illuminated by a Spectroline® UV light lamp (365 nm). Column chromatography was carried out using 35-70 µ, 60 Å silica gel. ^{1}H, ^{13}C and ^{19}F NMR spectroscopy were recorded on either 500 MHz (Bruker® AVII+ 500, Bruker AVIII HD 500) or 400 MHz (Bruker AVIII HD 400, Bruker AVIII 400) NMR spectrometers. Chemical shifts (δ) are reported in parts per million (ppm) and multiplicities are reported as singlets (s), broad singlets (brs), doublets (d), triplets (t), quartets (q), pentets (p), sextets (sx), heptets (h), combinations thereof (dt meaning a doublet of triplets), or multiplets (m). Coupling constants (*J*) are reported in Hertz (Hz). All ^{1}H NMR and ^{13}C NMR shifts were referenced to the residual solvent peak of $CDCl_3$ (^{1}H referenced to 7.26 ppm and ^{13}C referenced to 77.16 ppm). All ^{19}F chemical shifts were unadjusted from raw data. 2D heteronuclear single quantum coherence (HSQC), heteronuclear multiple bond correlation (HMBC), and homonuclear correlation (COSY) NMR spectroscopy were used to assist the assignment of signals. NMR yields were calculated from ^{1}H NMR spectra using mesitylene as an internal standard unless otherwise stated. Mass spectrometry measurements were carried out by the Mass Spectrometry Service in the Department of Chemistry at the University of Manchester (experiments and analysis was carried out by Gareth Smith and Emma Enston). High resolution mass spectrometry (HRMS) was recorded on ThermoFisher Scientific Q-ExactiveTM, Thermo Scientific Exactive plus EMR, and Agilent 6530 Q-TOF instruments, using either electrospray ionisation (ESI) or atmospheric-pressure chemical ionisation (APCI) – in some cases APCI was carried out using an atmospheric solids analysis probe (ASAP) – as ionisation methods in the positive and negative mode. Compound names are those generated by ChemDraw® (PerkinElmer®) following International Union of Pure and Applied Chemistry (IUPAC) nomenclature.

Safety Note: Care should be taken when heating solvents above their boiling point, and an appropriate pressure-resistant vessel should be used. As a precaution, a blast shield was used for large scale reactions (>1 mmol) that were heated above the solvent boiling point.

2 Pyridine N-transposition Optimisation

2.1 Additive Screen

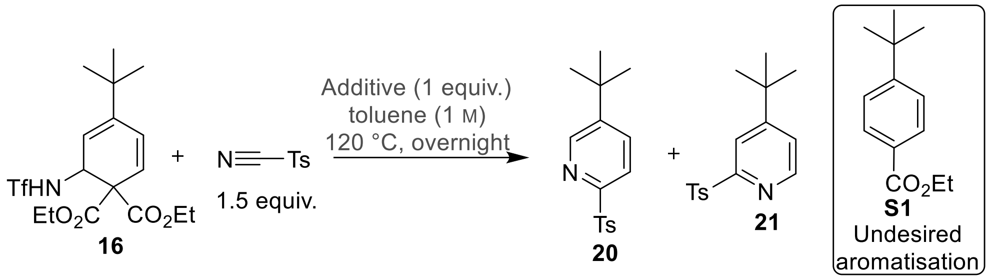

Entry	Additive	Conversion (%)	Yield (%) (20+21)	rr (20:21)	Yield (%) S1
**1**	None	64	54	48:52	0
**2**	$AlCl_3$	100	0	-	0
**3**	$Yb(OTf)_3$	100	0	-	55
**4**	$Cu(OTf)_2$	100	0	-	71
**5**	TMSOTf	100	0	-	91
**6**	K_2CO_3	100	0	-	71
**7**	CuI	64	0	-	33
**8**	TsOH	90	0	-	23
**9**	$Pd(OAc)_2$	69	0	-	0
**10**	Dibutyl phosphate	40	27	30:70	0
**11**	KF	11	4	50:50	0
**12**	LiCl	59	26	50:50	0

Table S1. Additive Screen for Pyridine N-Transposition. Yields and rr determined by ^{1}H NMR using mesitylene as an internal standard.

2.2 N-alkyl Group Screen

<u>Proposal:</u>

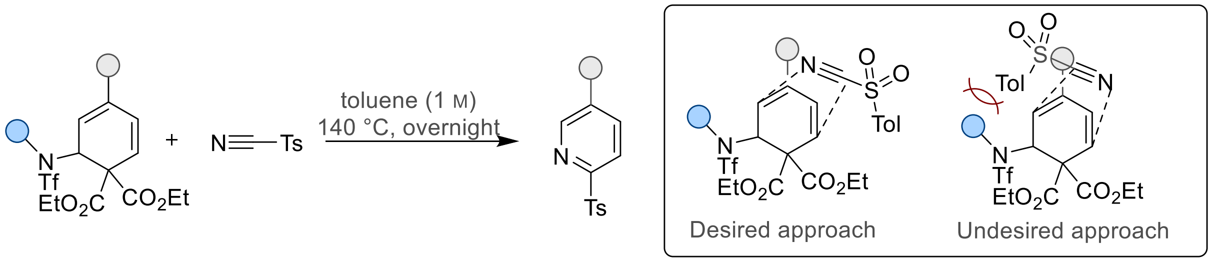

Figure S1. Proposed Regioselectivity for N-Alkylated carbocycles.

- It was proposed that a bulky group on the triflamide may favour the desired approach of the dienophile leading to the N-transposition product (Figure S1). We envisioned that steric clash between the tosyl group and N-substituent could occur in the alternative undesired approach, thus disfavouring this pathway.

Results:

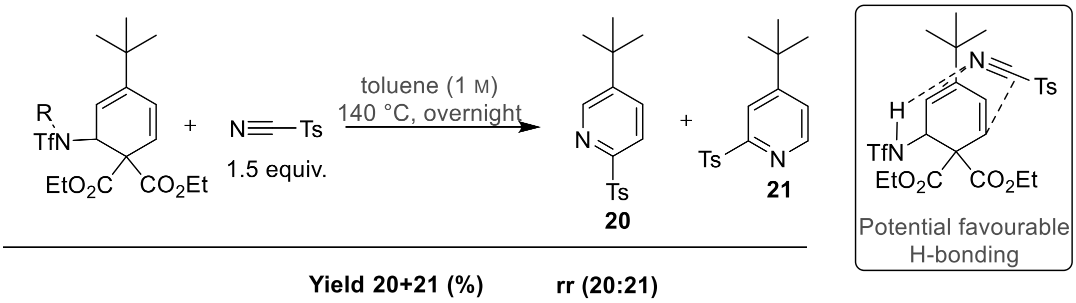

R	Yield 20+21 (%)	rr (20:21)
R = H	66	48:52
Me	50	4:96
Et	36	16:84
Bn	18	32:68

Table S2. Alkyl Group Screen for Pyridine N-Transposition. Yields and rr determined by ^{1}H NMR using mesitylene as an internal standard.

- In contrast with our proposal, methylation of the triflyl group gave near compete selectivity for the 2-functionalisation regioisomer **21** (Table S2).
- We propose this may be due to a hydrogen bonding interaction between the triflamide H and the nitrile N playing an important role in determining the regiochemical outcome of the cycloaddition – when this interaction is eliminated the transposition pathway is suppressed.
- Larger groups (Et and Bn) gave increasing amounts of the transposition isomer **20** compared with a methyl group, although conversion was significantly reduced and the 2-functionalisation product **21** remained the major regioisomer.

2.3 Solvent Screen

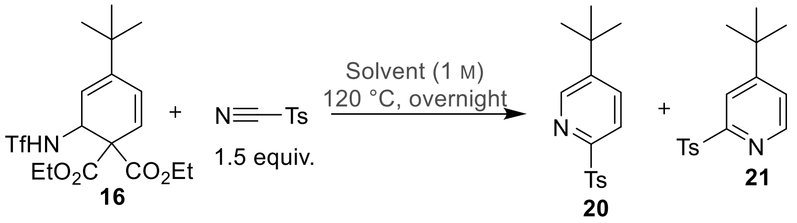

Entry	Solvent	Conversion (%)	Yield (%)	rr (20:21)
**1**	Toluene	64	54	48:52
**2**	MeCN	67	22	28:72
**3**	1,4-Dioxane	66	58	36:64
**4**	DCE	57	43	44:56
**5**	Toluene:Hexane (1:1)	58	57	45:55

Table S3. Solvent Screen for Pyridine N-Transposition. Yields and rr determined by ^{1}H NMR using mesitylene as an internal standard.

- Selectivity for the N-transposition product appears to be highest in non-hydrogen bonding, low-polarity solvents (Table S3). This is in-keeping with a hydrogen bonding interaction playing a role in the formation of the transposition product **20** (Table S2).

3 Pyridine to Pyridazine Optimisation

Proposed mechanism:

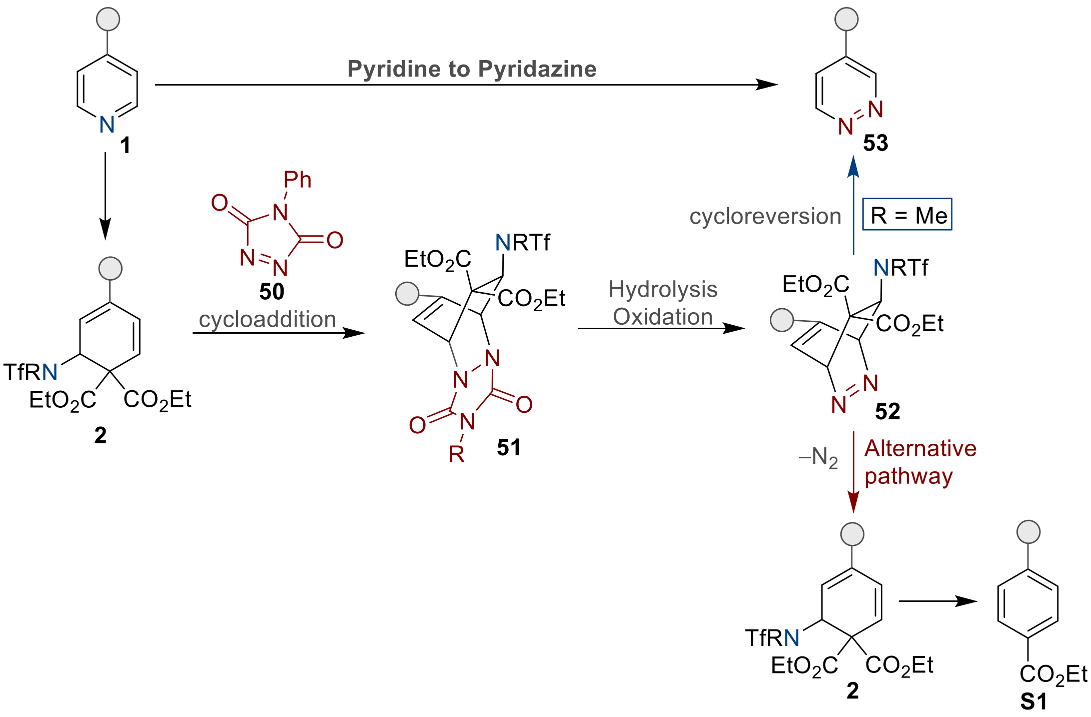

Scheme S1. Proposed Mechanism for Pyridine to Pyridazine Conversion via CACR.

- The above pathway is proposed for the developed pyridine to pyridazine conversion (Scheme S1). Although no oxidant is added, it is postulated that oxidation by air enables formation of intermediate **52** when cycloadduct **51** is subjected to hydrolysis conditions, which can undergo cycloreversion to the corresponding pyridazine **53**.
- It was noted during reaction development that methylation of the triflamide group is necessary in order to obtain the pyridazine product. Without the methyl group, under certain hydrolysis conditions the cycloadduct **51** was observed to give the benzene product **S1**, formed through N_2 extrusion and subsequent aromatisation as shown in Scheme S1. This alternative pathway is likely in competition with the desired cycloreversion process.
- It was also observed that cycloaddition of the methylated carbocycle with PTAD **50** in all cases gave the cycloadduct **51** as a single diastereomer. Although the configuration has not been determined it is proposed to be as shown in Scheme S1 based on steric considerations.

<u>Attempted optimisation:</u>

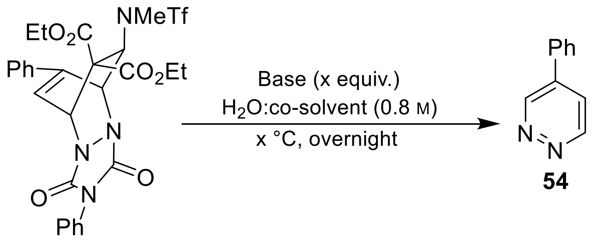

Entry	Base	Temp. (°C)	Base equiv.	Co-solvent	Yield (%)
**1**	KOH	120	250	1,4-Dioxane	29
**2**	NaOH	120	250	1,4-Dioxane	17
**3**	LiOH	120	250	1,4-Dioxane	1
**4**	KOH	140	250	1,4-Dioxane	19
**5**	KOH	100	250	1,4-Dioxane	27
**6**	KOH	60	250	1,4-Dioxane	21
**7**	KOH	120	125	1,4-Dioxane	3
**8**	KOH	120	375	1,4-Dioxane	31
**9**	KOH	120	250	EtOH	16
**10**	KOH	120	250	Ethylene glycol	14

Table S4. Optimisation of Pyridazine Formation. Yields determined by ^1H NMR using mesitylene as an internal standard.

4 2-Tosyl Pyridine Suzuki Coupling Optimisation

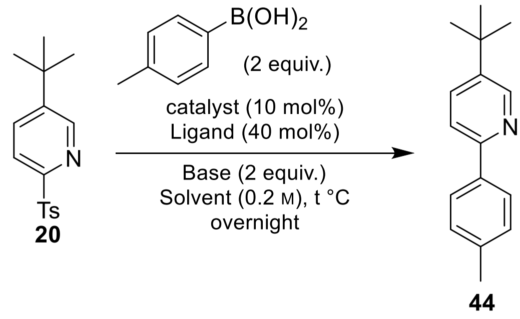

Entry	Catalyst	Ligand	Base	Solvent	Temp. (°C)	Yield (%)
**1**	$NiCl_2$.glyme	$Cy_3P.HBF_4$	KOH	THF	80	0
**2**	$NiBr_2$.glyme	$Cy_3P.HBF_4$	KOH	^tBuOH	80	26
**3**	$Ni(PCy_3)Cl_2$	-	KOH	^tBuOH	120	55
**4**	$Ni(PCy_3)Cl_2$	-	K_3PO_4	^tBuOH	120	30
**5**	$NiBr_2$.glyme	ProPhos	K_3PO_4[a]	^tBuOH	120	76
**6**	$NiBr_2$.glyme	ProPhos	K_3PO_4[a]	^iPrOH	120	92

Table S5. 2-Tosyl Pyridine Suzuki Coupling Optimisation. Yields determined by ^1H NMR using mesitylene as an internal standard. [a]2.5 equiv.

5 Limitations

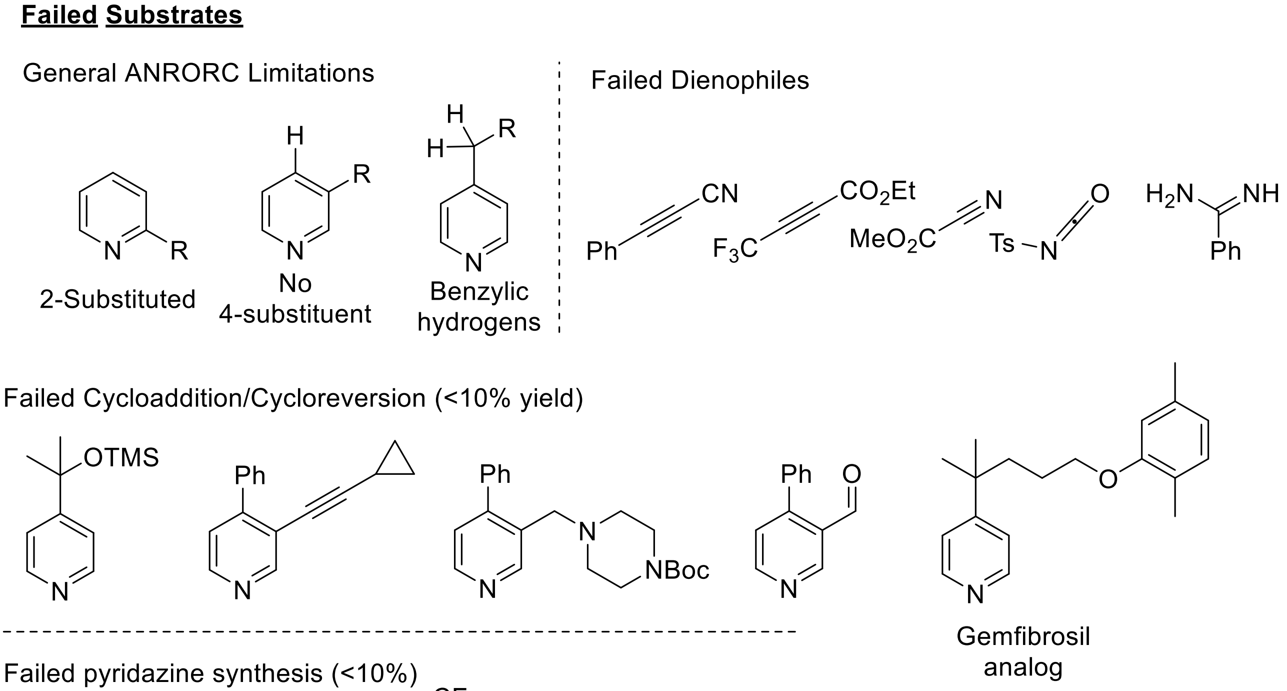

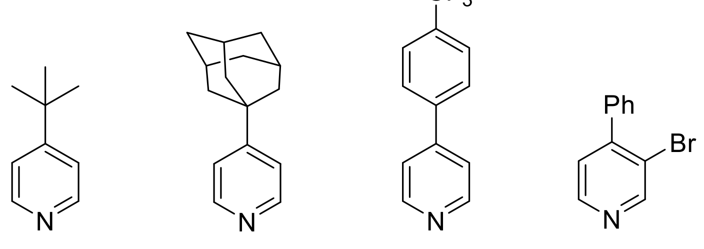

Figure S2. Limitations.

6 General Synthetic Procedures

General Procedure 1: Carbocycle formation via ANRORC

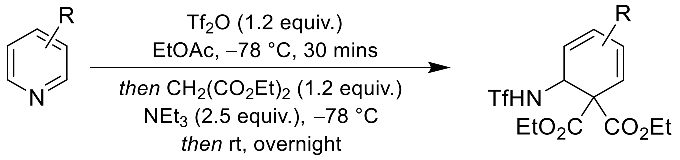

To a microwave vial equipped with a stir bar was added the appropriate pyridine (1 equiv.) and the vessel was capped and evacuated and refilled with N_2 three times on a Schlenk line (volatile pyridines were added after the final N_2 refill). EtOAc (0.1 M) was added, and the resulting solution was cooled to −78 °C. Tf_2O (1.2 equiv.) was added dropwise, and the resulting precipitate stirred for 30 mins. Diethyl malonate (1.2 equiv.) was added, followed by NEt_3 (2.5 equiv.) and the reaction mixture was allowed to warm to room temperature and stirred overnight. The solvent was removed *in vacuo* to give the crude product which was purified by silica column chromatography.

General Procedure 2: Cycloaddition/cycloreversion reactions

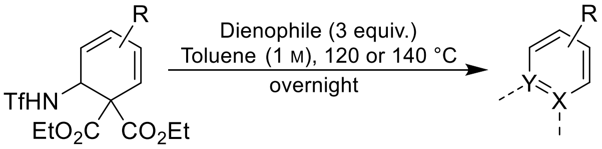

To a microwave vial equipped with a stir bar was added the appropriate carbocycle (1 equiv.) and dienophile (3 equiv.) and the vessel was capped and evacuated and refilled with N_2 three times on a Schlenk line (volatile dienophiles were added after the final N_2 refill). Toluene (1 M) was added and the resulting mixture was heated with stirring at 120 °C or 140 °C as stated overnight. The reaction mixture was allowed to cool and concentrated *in vacuo* to give the crude product which was purified by silica column chromatography.

General Procedure 3: Suzuki coupling of 2-tosyl pyridines and aryl boronic acids

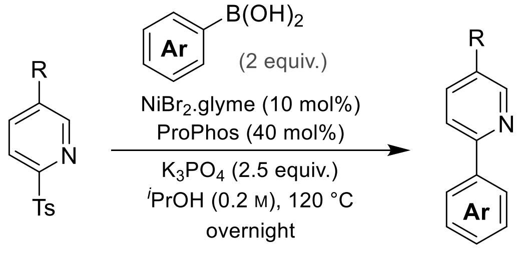

To a microwave vial equipped with a stir bar was added the appropriate 2-tosyl pyridine (1 equiv.), the appropriate boronic acid (2 equiv.), K_3PO_4 (2.5 equiv.), $NiBr_2$.glyme (10 mol%), and ProPhos (40 mol%). The vessel was capped and evacuated and refilled with N_2 three times

on a Schlenk line before addition of ^{i}PrOH (0.2 M). The resulting suspension was sparged with N_2 for 2 minutes before heating to 120 °C overnight with stirring. The mixture was allowed to cool to room temperature, diluted with H_2O, and extracted three times with CH_2Cl_2. The combined organic extracts were dried over $MgSO_4$, filtered, and concentrated *in vacuo* to give the crude product which was purified by silica column chromatography.

General Procedure 4: Methylation and PTAD cycloaddition

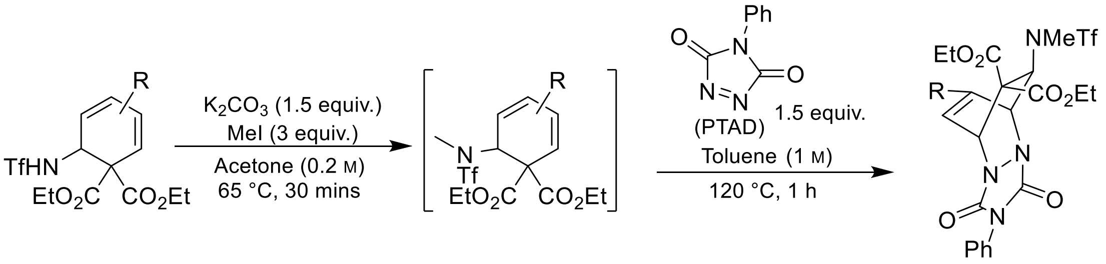

To a microwave vial equipped with a stir bar was added the appropriate carbocycle (1 equiv.) and K_2CO_3 (1.5 equiv.) and the vessel was capped and evacuated and refilled with N_2 three times on a Schlenck line. Acetone (0.2 M) was added, followed by MeI (3 equiv.), and the mixture was heated to 65 °C and stirred for 30 mins. The mixture was allowed to cool to room temperature and concentrated *in vacuo*. The residue was diluted with water and extracted three times with CH_2Cl_2 to give the methylated carbocycle.

To a microwave vial equipped with a stir bar was added the methylated carbocycle and PTAD (1.5 equiv.) and the vial was capped. Toluene (1 M) was added and the resulting solution was heated with stirring at 120 °C for 1 h. The reaction mixture was allowed to cool to room temperature and concentrated *in vacuo* to give the crude product which was purified by silica column chromatography.

General Procedure 5: Pyridazine formation

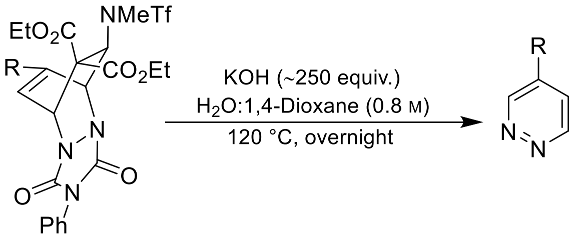

To a microwave vial equipped with a stir bar was added the appropriate cycloadduct (1 equiv.) and KOH (approx. 250 equiv.), followed by a 1:1 mixture of 1,4-dioxane and H_2O (0.8 M). The vial was capped and heated to 120 °C overnight with stirring. The mixture was allowed to cool to room temperature, diluted with H_2O, and extracted three times with EtOAc. The combined

organic extracts were dried over $MgSO_4$, filtered, and concentrated *in vacuo* to give the crude product which was purified by silica column chromatography.

7 Synthesis of Substrates

Unless otherwise stated below, all pyridine substrates were either commercially available, or synthesised as described by our group previously.[1,2]

1-(Pyridin-4-yl)cyclopropane-1-carbonitrile (S3)

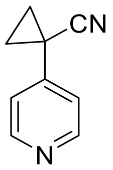

Following a literature procedure,[3] to a microwave vial equipped with a stir bar was added 2-(pyridin-4-yl)acetonitrile (118.1 mg, 1 mmol) and vinyl diphenylsulfonium triflate (434.9 mg, 1.2 mmol). The vial was capped and evacuated and refilled with N_2 three times on a Schlenk line. DMSO (5 mL) was added and the mixture was stirred for two minutes before addition of DBU (0.45 mL, 3 mmol). The resulting mixture was stirred overnight before addition of sat. aq. NH_4Cl. The aqueous phase was extracted three times with CH_2Cl_2 and the combined organic extracts were dried ($MgSO_4$), filtered, and concentrated *in vacuo* to give the crude product, which was purified by silica column chromatography (*n*-hexane/EtOAc, 100:0 to 1:1) to yield the product (136.3 mg, 95%) as a white solid with data in accordance with the literature.[3]

^{1}H NMR (500 MHz, $CDCl_3$): δ 8.52 (d, J = 5.2 Hz, 2H), 7.12 (d, J = 6.3 Hz, 2H), 1.82 (q, J = 5.4 Hz, 2H), 1.47 (q, J = 5.4 Hz, 2H);

^{13}C{^{1}H} NMR (126 MHz, $CDCl_3$): δ 150.2, 145.6, 120.8, 119.5, 19.9, 13.5.

2-Methyl-2-(pyridin-4-yl)propanenitrile (S4)

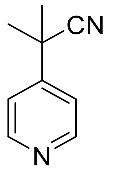

Following a modified literature procedure,[4] to a microwave vial equipped with a stir bar was added 2-(pyridin-4-yl)acetonitrile (236.2 mg, 2 mmol) and the vial was capped and evacuated and refilled with N_2 three times on a Schlenk line. THF (4 mL) was added, followed by MeI (0.38 mL, 6 mmol). ^{t}BuOK (1 M solution in THF, 6 mL, 6 mmol) was added slowly and the

resulting mixture was stirred overnight. Water was added and the aqueous layer was extracted three times with CH_2Cl_2. The combined organic extracts were dried ($MgSO_4$), filtered, and concentrated *in vacuo* to give the crude product which was purified by silica column chromatography (*n*-hexane/EtOAc, 100:0 to 1:1) to yield the product (286.6 mg, 98%) as a yellow oil with data in accordance with the literature.[4]

^{1}H NMR (500 MHz, $CDCl_3$): δ 8.64 (d, *J* = 6.3 Hz, 2H), 7.39 (d, *J* = 6.3 Hz, 2H), 1.73 (s, 6H);
^{13}C{^{1}H} NMR (126 MHz, $CDCl_3$): δ 150.6, 150.2, 123.1, 120.2, 37.1, 28.5.

4-(1-(4-Chlorophenyl)cyclobutyl)pyridine (S5)

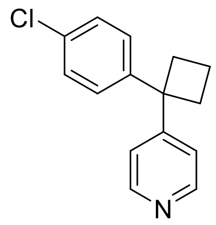

Following a literature procedure,[5] to an oven dried microwave vial equipped with a stir bar was added 4-cyanopyridine (312.3 mg, 3 mmol), 1,3-dioxoisoindolin-2-yl 1-(4-chlorophenyl)cyclobutane-1-carboxylate (711.5 mg, 2 mmol), $B_2(pin)_2$ (609.5 mg, 2.4 mmol), and $ZnCl_2$ (27.3 mg, 0.2 mmol). The vessel was evacuated and refilled with N_2 three times on a Schlenck line. MTBE (10 mL) was added and the solution was sparged with N_2 for 5 minutes before heating to 80 °C overnight. The reaction mixture was allowed to cool to room temperature before quenching with 2 M Na_2CO_3 solution (10 mL). The mixture was extracted with EtOAc, and the combined organic extracts dried ($MgSO_4$), filtered, and concentrated *in vacuo* to give the crude product which was purified by silica column chromatography (*n*-hexane/EtOAc, 100:0 to 1:1) to yield the product (284.2 mg, 58%) as a colourless oil of sufficient purity for use without further purification.

^{1}H NMR (500 MHz, $CDCl_3$): δ 8.54 (d, *J* = 5.3 Hz, 2H), 7.34–7.26 (m, 4H), 7.22 (d, *J* = 8.6 Hz, 2H), 2.89–2.58 (m, 4H), 2.15–1.83 (m, 2H).

1-(Pyridin-4-yl)cyclohexane-1-carbonitrile (S6)

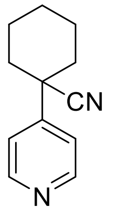

Following a literature procedure,[6] NaH (160.0 mg, 4 mmol) was suspended in anhydrous DMF (2 mL) under N_2 and cooled to 0 °C. A solution of 2-(pyridin-4-yl)acetonitrile (236.3 mg, 2 mmol) and 1,5-dibromopentane (0.27 ml, 2 mmol) in DMF (1 mL) was added dropwise and the solution was allowed to warm to rt and stirred overnight. The solution was quenched with 2 M HCl and extracted with EtOAc. The combined organic extracts were dried ($MgSO_4$), filtered, and concentrated *in vacuo* to give the crude product, which was purified by silica column chromatography (*n*-hexane/EtOAc, 100:0 to 1:1) to yield the product (337.5 mg, 91%) as a white solid with data in accordance with the literature.[6]

^{1}H NMR (500 MHz, $CDCl_3$): δ 8.65 (d, *J* = 6.3 Hz, 2H), 7.45 (d, *J* = 6.3 Hz, 2H), 2.21–2.07 (m, 2H), 1.99–1.64 (m, 7H), 1.38–1.15 (m, 1H);

^{13}C{^{1}H} NMR (126 MHz, $CDCl_3$): δ 150.5, 150.1, 121.4, 120.6, 44.3, 36.7, 24.9, 23.3.

4-(1-Phenylcyclohexyl)pyridine (S7)

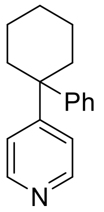

Following a literature procedure,[5] to an oven dried microwave vial equipped with a stir bar was added 4-cyanopyridine (312.3 mg, 3 mmol), 1,3-dioxoisoindolin-2-yl 1-phenylcyclohexane-1-carboxylate (698.8 mg, 2 mmol), $B_2(pin)_2$ (609.5 mg, 2.4 mmol), and $ZnCl_2$ (27.3 mg, 0.2 mmol). The vessel was evacuated and refilled with N_2 three times on a Schlenck line. MTBE (10 mL) was added and the solution was sparged with N_2 for 5 minutes before heating to 80 °C overnight. The reaction mixture was allowed to cool to room temperature before quenching with 2 M Na_2CO_3 solution (10 mL). The mixture was extracted with EtOAc, and the combined organic extracts dried ($MgSO_4$), filtered, and concentrated *in vacuo* to give the crude product which was purified by silica column chromatography (*n*-hexane/EtOAc, 100:0 to 1:1) to yield the product (285.9 mg, 60%) as a colourless oil with data in accordance with the literature.[5]

^{1}H NMR (500 MHz, $CDCl_3$): δ 8.52 (d, *J* = 6.6 Hz, 2H), 7.38 (dd, *J* = 5.0, 1.6 Hz, 3H), 7.36–7.26 (m, 5H), 7.25–7.12 (m, 1H), 2.37 (dd, *J* = 15.4, 5.4 Hz, 2H), 2.24 (dd, *J* = 16.2, 6.3 Hz, 4H), 1.69–1.46 (m, 6H);

^{13}C{^{1}H} NMR (126 MHz, $CDCl_3$): δ 158.0, 149.8, 146.7, 128.6, 127.3, 126.1, 122.6, 46.2, 36.5, 26.2, 22.8.

Ethyl 2,2-difluoro-2-(pyridin-4-yl)acetate (S8)

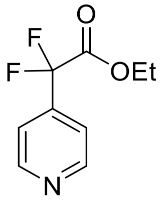

Following a literature procedure,[7] to a microwave vial equipped with a stir bar was added ethyl 2-(pyridin-4-yl)acetate (330.4 mg, 2 mmol) followed by MeCN (10 mL). To the resulting solution was slowly added Li_2CO_3 (326.0 mg, 4.4 mmol) and NFSI (1.262 g, 4 mmol). The mixture was stirred overnight and then filtered, washing the residue with MeCN. The filtrate was concentrated *in vacuo* to give the crude product which was purified by silica column chromatography (*n*-hexane/EtOAc, 100:0 to 4:1) to yield the product (233.8 mg, 58%) as a yellow oil with data in accordance with the literature.[7] Note: Care was taken during isolation due to the volatility of the product.

^{1}H NMR (500 MHz, $CDCl_3$): δ 8.76 (d, J = 5.1 Hz, 2H), 7.64–7.43 (m, 2H), 4.32 (q, J = 7.1 Hz, 2H), 1.31 (t, J = 7.1 Hz, 3H);

^{19}F{^{1}H} NMR (377 MHz, $CDCl_3$): δ −106.6.

3-Chloro-4-phenylpyridine (S9)

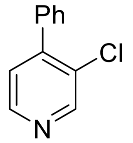

Following a modified literature procedure,[1] to a microwave vial equipped with a stir bar was added 3-chloro-4-iodopyridine (478.9 mg, 2 mmol), phenyl boronic acid (292.6 mg, 2 mmol), K_2CO_3 (1.1057 g, 8 mmol), $Pd(OAc)_2$ (22.5 mg, 0.1 mmol), and PPh_3 (104.9 mg, 0.4 mmol), and the vessel was evacuated and refilled with N_2 three times on a Schlenk line. A 1:1 mixture of DME and water (8 mL), which was previously degassed by bubbling through N_2 for five minutes, was added and the resulting biphasic mixture was heated to 90 °C with stirring overnight. The mixture was allowed to cool to room temperature, diluted with H_2O, and extracted three times with EtOAc. The combined organic extracts were dried over $MgSO_4$, filtered, and concentrated in vacuo to yield the crude product, which was purified by silica column chromatography (*n*-hexane/EtOAc, 100:0 to 4:1) to yield the product (371.1 mg, 98%) as a yellow oil with data in accordance with the literature.

^{1}H NMR (500 MHz, $CDCl_3$): δ 8.68 (s, 1H), 8.53 (d, J = 5.0, 1H), 7.49 – 7.46 (m, 5H), 7.29 (dd, J = 5.0, 1.8, 1H);

$^{13}C\{^1H\}$ **NMR** (126 MHz, $CDCl_3$): δ 150.1, 147.8, 147.7, 136.5, 130.2, 128.9, 128.9, 128.5, 125.4.

3-Chloro-4-(thiophen-3-yl)pyridine (S10)

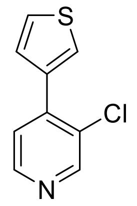

Following a modified literature procedure,[1] to a microwave vial equipped with a stir bar was added 3-chloro-4-iodopyridine (478.9 mg, 2 mmol), thiophen-3-ylboronic acid (255.9 mg, 2 mmol), K_2CO_3 (1.1057 g, 8 mmol), $Pd(OAc)_2$ (22.5 mg, 0.1 mmol), and PPh_3 (104.9 mg, 0.4 mmol), and the vessel was evacuated and refilled with N_2 three times on a Schlenk line. A 1:1 mixture of DME and water (8 mL), which was previously degassed by bubbling through N_2 for five minutes, was added and the resulting biphasic mixture was heated to 90 °C with stirring overnight. The mixture was allowed to cool to room temperature, diluted with H_2O, and extracted three times with EtOAc. The combined organic extracts were dried over $MgSO_4$, filtered, and concentrated in vacuo to yield the crude product, which was purified by silica column chromatography (*n*-hexane/EtOAc, 100:0 to 4:1) to yield the product (389.0 mg, 99%) as a yellow oil.

^1H **NMR** (500 MHz, $CDCl_3$): δ 8.65 (s, 1H), 8.47 (d, J = 5.0, 1H), 7.69 (dd, J = 2.9, 1.4, 1H), 7.42 (dd, J = 5.0, 2.9, 1H), 7.39 (dd, J = 5.0, 1.4, 1H), 7.35 (d, J = 5.0, 1H);

$^{13}C\{^1H\}$ **NMR** (126 MHz, $CDCl_3$): δ 150.6, 148.0, 142.1, 136.6, 129.8, 128.1, 126.3, 126.0, 124.7.

1,3-Diethyl-3-(pyridin-4-yl)piperidine-2,6-dione (37)

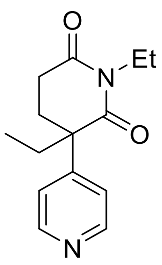

Following a modified literature procedure,[8] to a solution of ethyl 2-(pyridin-4-yl)acetate (153.1 μL, 2 mmol) in anhydrous ^{t}BuOH (3.4 mL) in a dry microwave vial under N_2 was added KO^{t}Bu (1 M in THF, 2.2 mL, 2.2 mmol). To the resulting mixture was added EtI (0.16 mL, 2 mmol) dropwise and the solution was stirred for 1.5 h. Acrylamide (213.2 mg, 3.0 mmol) was added

as a solution in anhydrous ^{t}BuOH (1.7 mL). ^{t}BuOK (1 M in THF, 2.2 mL, 2.2 mmol) was added and the solution stirred overnight. The reaction mixture was diluted with H_2O and acidified to neutral pH by addition of 1M HCl. The mixture was extracted three times with EtOAc, and the combined organic extracts dried ($MgSO_4$), filtered, and concentrated *in vacuo* to give the crude product which was purified by silica column chromatography (0-10% MeOH in CH_2Cl_2) to yield 3-ethyl-3-(pyridin-4-yl)piperidine-2,6-dione (397.2 mg, 91%) as a white solid.

Following a literature procedure,[6] to a solution of 3-ethyl-3-(pyridin-4-yl)piperidine-2,6-dione (397.2 mg, 1.82 mmol) in MeCN (0.2 M) was added Cs_2CO_3 (1.79 g, 5.46 mmol) and bromoethane (0.15 mL, 2.00 mmol) and the resulting solution heated at reflux for 30 minutes. The reaction mixture was concentrated *in vacuo* to give the crude product, which was purified by silica column chromatography (0-10% MeOH in CH_2Cl_2) to yield the product (434.8 mg, 97%) as a colourless oil with data in accordance with the literature.[8]

^{1}H NMR (500 MHz, $CDCl_3$): δ 8.57 (d, J = 4.7 Hz, 2H), 7.15–7.10 (m, 2H), 3.95–3.76 (m, 2H), 2.74–2.57 (m, 1H), 2.47–2.15 (m, 3H), 2.06 (dtd, J = 14.8, 7.4, 1.2 Hz, 1H), 1.86 (dtd, J = 14.8, 7.4, 1.2 Hz, 1H), 1.12 (td, J = 7.0, 1.3 Hz, 3H), 0.85 (td, J = 7.4, 1.3 Hz, 3H);

^{13}C{^{1}H} NMR (126 MHz, $CDCl_3$): δ 173.7, 171.3, 150.6, 149.1, 121.5, 51.3, 35.6, 33.4, 29.7, 25.5, 13.3, 9.1.

8 Synthesis of Carbocycles

Diethyl 3-((trifluoromethyl)sulfonamido)-[1,1'-biphenyl]-4,4(3*H*)-dicarboxylate (4)

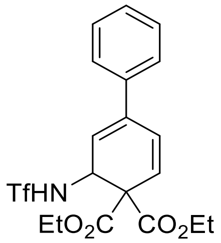

Following **General Procedure 1**, 4-phenylpyridine (155.2 mg, 1 mmol), Tf_2O (0.2 mL, 1.2 mmol), diethyl malonate (0.18 mL, 1.2 mmol), and NEt_3 (0.35 mL, 2.5 mmol) gave the crude product, which was purified by silica column chromatography (*n*-hexane/EtOAc, 100:0 to 4:1) to yield the product (363.6 mg, 81%) as a white solid.

^{1}H NMR (500 MHz, $CDCl_3$): δ 7.43–7.29 (m, 5H), 6.51 (dd, J = 9.7, 1.4 Hz, 1H), 6.43 (d, J = 10.3 Hz, 1H), 6.23 (d, J = 9.7 Hz, 1H), 6.02 (d, J = 4.0 Hz, 1H), 5.11 (dd, J = 10.3, 4.0 Hz, 1H), 3.52–4.48 (m, 4H), 1.34–1.24 (m, 6H);

^{13}C{^{1}H} NMR (126 MHz, $CDCl_3$): δ 168.5, 168.1, 137.4, 136.6, 128.9, 128.7, 127.1, 125.8, 125.6, 122.6, 119.6 (q, J = 317.2 Hz), 63.0, 62.8, 58.9, 55.3, 14.0, 13.9;

^{19}F{^{1}H} NMR (377 MHz, $CDCl_3$): δ −77.9;

HRMS (ESI$^+$) $C_{19}H_{20}F_3NO_6SNa$ [M+Na]$^+$ found 470.0860, requires 470.0856 (+0.85 ppm).

Diethyl 4-(tert-butyl)-6-((trifluoromethyl)sulfonamido)cyclohexa-2,4-diene-1,1-dicarboxylate (16)

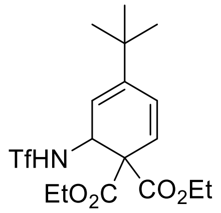

Following **General Procedure 1**, 4-(*tert*-butyl)pyridine (146.5 μL, 1 mmol), Tf_2O (0.2 mL, 1.2 mmol), diethyl malonate (0.18 mL, 1.2 mmol), and NEt_3 (0.35 mL, 2.5 mmol) gave the crude product, which was purified by silica column chromatography (*n*-hexane/EtOAc, 100:0 to 4:1) to yield the product (427.5 mg, >99%) as a white solid.

^{1}H NMR (500 MHz, $CDCl_3$): δ 6.32 (d, *J* = 10.2 Hz, 1H), 6.24 (d, *J* = 9.8 Hz, 1H), 6.06 (d, *J* = 9.8 Hz, 1H), 5.52 (d, *J* = 3.7 Hz, 1H), 4.90 (dd, *J* = 10.2, 3.7 Hz, 1H), 4.32–4.06 (m, 4H), 1.26 (qd, *J* = 7.2, 0.8 Hz, 6H);

^{13}C{^{1}H} NMR (126 MHz, $CDCl_3$): δ 168.7, 168.4, 145.7, 126.3, 124.6, 119.6 (q, *J* = 320.5 Hz), 118.4, 62.8, 62.5, 58.8, 55.3, 34.0, 28.3, 14.0;

^{19}F{^{1}H} NMR (377 MHz, $CDCl_3$): δ −78.0;

HRMS (APCI) $C_{17}H_{25}F_3NO_6S$ [M+H]$^+$ found 428.1345, requires 428.1349 (−0.98 ppm).

Diethyl 4-((3r,5r,7r)-adamantan-1-yl)-6-((trifluoromethyl)sulfonamido)cyclohexa-2,4-diene-1,1-dicarboxylate (S11)

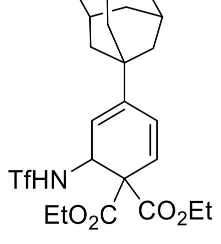

Following **General Procedure 1**, 4-((3*r*,5*r*,7*r*)-adamantan-1-yl)pyridine (213.3 mg, 1 mmol), Tf_2O (0.2 mL, 1.2 mmol), diethyl malonate (0.18 mL, 1.2 mmol), and NEt_3 (0.35 mL, 2.5 mmol) gave the crude product, which was purified by silica column chromatography (*n*-hexane/EtOAc, 100:0 to 4:1) to yield the product (482.0 mg, 95%) as a white solid.

¹H NMR (500 MHz, $CDCl_3$): δ 6.36–6.21 (m, 2H), 6.04 (d, J = 9.9 Hz, 1H), 5.43 (d, J = 3.6 Hz, 1H), 4.90 (dd, J = 10.3, 3.6 Hz, 1H), 4.32–4.11 (m, 4H), 2.15–1.92 (m, 3H), 1.76–1.69 (m, 3H), 1.67–1.60 (m, 3H), 1.60–1.52 (m, 6H), 1.45–1.12 (m, 6H);

¹³C{¹H} NMR (126 MHz, $CDCl_3$): δ 168.7, 168.4, 145.8, 125.6, 124.4, 119.6 (q, J = 320.6 Hz), 118.8, 62.7, 62.5, 58.9, 55.3, 40.2, 36.7, 35.6, 28.3, 14.0;

¹⁹F{¹H} NMR (377 MHz, $CDCl_3$): δ = −78.0;

HRMS (APCI) $C_{23}H_{30}F_3NO_6SNa$ $[M+Na]^+$ found 528.1622, requires 528.1638 (−3.06 ppm).

Diethyl 4-(thiophen-3-yl)-6-((trifluoromethyl)sulfonamido)cyclohexa-2,4-diene-1,1-dicarboxylate (S12)

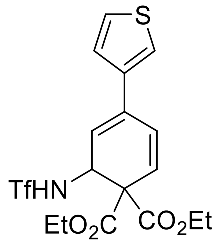

Following **General Procedure 1**, 4-(thiophen-3-yl)pyridine (161.2 mg, 1 mmol), Tf_2O (0.2 mL, 1.2 mmol), diethyl malonate (0.18 mL, 1.2 mmol), and NEt_3 (0.35 mL, 2.5 mmol) gave the crude product, which was purified by silica column chromatography (*n*-hexane/EtOAc, 100:0 to 4:1) to yield the product (422.7 mg, 93%) as a white solid.

¹H NMR (500 MHz, $CDCl_3$): δ 7.35–7.24 (m, 2H), 7.22–7.17 (m, 1H), 6.54–6.46 (m, 2H), 6.22 (dd, J = 9.9, 2.0 Hz, 1H), 6.05 (d, J = 4.4 Hz, 1H), 5.17–4.94 (m, 1H), 4.31–4.16 (m, 4H), 1.31–1.22 (m, 6H);

¹³C{¹H} NMR (126 MHz, $CDCl_3$): δ 168.3, 167.8, 138.7, 131.1, 126.7, 126.5, 125.4, 125.1, 121.5, 120.2, 119.5 (q, J = 320.6 Hz), 62.9, 62.8, 58.9, 54.6;

¹⁹F{¹H} NMR (377 MHz, $CDCl_3$): δ −77.9;

HRMS (APCI) $C_{17}H_{19}F_3NO_6S_2$ $[M+H]^+$ found 454.0613, requires 454.0600 (+2.78 ppm).

Diethyl 4'-cyano-3-((trifluoromethyl)sulfonamido)-[1,1'-biphenyl]-4,4(3H)-dicarboxylate (S13)

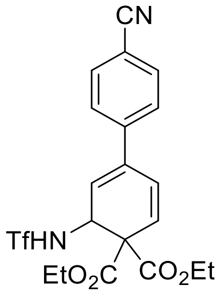

Following **General Procedure 1**, 4-(pyridin-4-yl)benzonitrile (180.2 mg, 1 mmol), Tf_2O (1.2 mL, 1.2 mmol), diethyl malonate (0.18 mL, 1.2 mmol), and NEt_3 (0.35 mL, 2.5 mmol) gave the crude product, which was purified by silica column chromatography (*n*-hexane/EtOAc, 100:0 to 4:1) to yield the product (416.4 mg, 88%, ~90% purity) as a white solid which was used without further purification.

^{1}H NMR (500 MHz, $CDCl_3$): δ 7.68 (d, J = 8.5 Hz, 2H), 7.48 (d, J = 8.5 Hz, 2H), 6.54 (dd, J = 10.2, 3.4 Hz, 1H), 6.48 (dd, J = 9.8, 1.4 Hz, 1H), 6.30 (d, J = 9.8 Hz, 1H), 6.13 (d, J = 3.8 Hz, 1H), 5.14 (dd, J = 10.2, 3.8 Hz, 1H), 4.47–4.22 (m, 4H), 1.31 (dt, J = 10.2, 7.2 Hz, 6H);

^{13}C{^{1}H} NMR (126 MHz, $CDCl_3$): δ 168.2, 167.9, 141.7, 135.3, 132.7, 126.7, 126.5, 125.8, 125.5, 121.9, 119.5 (q, J = 320.5 Hz), 118.6, 112.3, 63.2, 62.9, 58.7, 55.1, 14.0, 13.9;

^{19}F{^{1}H} NMR (377 MHz, $CDCl_3$): δ −77.9;

HRMS (APCI) $C_{20}H_{20}F_3N_2O_6S$ $[M+H]^+$ found 473.0981, requires 473.0989 (−1.62 ppm).

Diethyl 4'-methoxy-3-((trifluoromethyl)sulfonamido)-[1,1'-biphenyl]-4,4(3H)-dicarboxylate (S14)

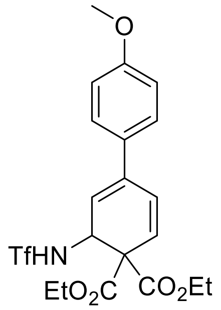

Following **General Procedure 1**, 4-(4-methoxyphenyl)pyridine (185.2 mg, 1 mmol), Tf_2O (0.2 mL, 1.2 mmol), diethyl malonate (0.18 mL, 1.2 mmol), and NEt_3 (0.35 mL, 2.5 mmol) gave the crude product, which was purified by silica column chromatography (*n*-hexane/EtOAc, 100:0 to 4:1) to yield the product (340.2 mg, 71%) as a white solid.

^{1}H NMR (500 MHz, $CDCl_3$): δ 7.31 (d, J = 8.5 Hz, 2H), 6.89 (d, J = 8.5 Hz, 2H), 6.50 (dd, J = 9.7, 1.4 Hz, 1H), 6.32 (br s, 1H), 6.21 (d, J = 9.7 Hz, 1H), 5.93 (d, J = 4.0 Hz, 1H), 5.08 (d, J = 4.0 Hz, 1H), 4.38–4.12 (m, 4H), 3.82 (s, 3H), 1.28 (dt, J = 9.2, 7.1 Hz, 6H);

$^{13}C\{^1H\}$ NMR (126 MHz, $CDCl_3$): δ 168.5, 168.0, 160.0, 135.8, 129.9, 127.1, 127.0, 125.4, 120.4, 119.6 (q, J = 320.5 Hz), 114.2, 62.9, 62.7, 58.9, 55.5, 55.0, 14.0, 13.9;

$^{19}F\{^1H\}$ NMR (377 MHz, $CDCl_3$): δ −77.9;

HRMS (APCI) $C_{20}H_{23}F_3NO_7S$ $[M+H]^+$ found 478.1148, requires 478.1142 (+1.29 ppm).

Diethyl 6-bromo-3-((trifluoromethyl)sulfonamido)-[1,1'-biphenyl]-4,4(3H)-dicarboxylate (S15)

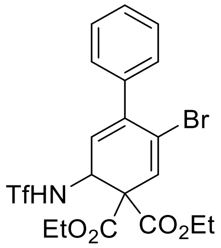

Following **General Procedure 1**, 3-bromo-4-phenylpyridine (234.1 mg, 1 mmol), Tf_2O (0.2 mL, 1.2 mmol), diethyl malonate (0.18 mL, 1.2 mmol), and NEt_3 (0.35 mL, 2.5 mmol) gave the crude product, which was purified by silica column chromatography (*n*-hexane/EtOAc, 100:0 to 4:1) to yield the product (236.3 mg, 45%, ~90% purity) as a white solid which was used without further purification (impurity due to co-elution with diethyl malonate).

^1H NMR (500 MHz, $CDCl_3$): δ 7.41–7.30 (m, 3H), 7.23–7.15 (m, 2H), 6.59 (d, J = 10.3 Hz, 1H), 5.91 (d, J = 3.7 Hz, 1H), 5.10 (dd, J = 10.2, 3.7 Hz, 1H), 4.41–4.25 (m, 4H), 1.92–1.26 (m, 6H);

$^{13}C\{^1H\}$ NMR (126 MHz, $CDCl_3$): δ 167.7, 167.0, 139.7, 137.2, 128.7, 128.4, 128.2, 127.8, 127.0, 121.9, 119.5 (q, J = 320.5 Hz), 63.4, 63.2, 61.1, 54.8, 14.0;

$^{19}F\{^1H\}$ NMR (377 MHz, $CDCl_3$): δ −77.9;

HRMS (APCI) $C_{19}H_{20}BrF_3NO_6S$ $[M+H]^+$ found 526.0124, requires 526.0141 (−3.29 ppm).

Diethyl 4-((8R,9S,13R,14R)-14-methyl-15-oxo-7,8,9,11,12,13,14,15,16,17-decahydro-6H-cyclopenta[a]phenanthren-2-yl)-6-((trifluoromethyl)sulfonamido)cyclohexa-2,4-diene-1,1-dicarboxylate (S16)

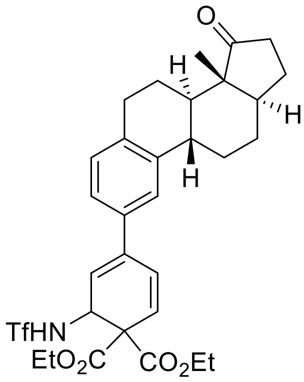

Following **General Procedure 1**, (8*R*,9*S*,13*S*,14*S*)-13-methyl-3-(pyridin-4-yl)-6,7,8,9,11,12,13,14,15,16-decahydro-17*H*-cyclopenta[a]phenanthren-17-one (331.5 mg, 1 mmol), Tf_2O (0.2 mL, 1.2 mmol), diethyl malonate (0.18 mL, 1.2 mmol), and NEt_3 (0.35 mL, 2.5 mmol) gave the crude product, which was purified by silica column chromatography (*n*-hexane/EtOAc, 100:0 to 4:1) to yield the product (342.1 mg, 55%) as a white solid.

^{1}H NMR (500 MHz, $CDCl_3$): δ 7.30 (d, *J* = 8.1 Hz, 1H), 7.17 (dd, *J* = 8.1, 2.0 Hz, 1H), 7.11 (d, *J* = 2.0 Hz, 1H), 6.56–6.47 (m, 2H), 6.21 (d, *J* = 9.7 Hz, 1H), 6.00 (d, *J* = 4.1 Hz, 1H), 5.08 (dd, *J* = 10.2, 4.1 Hz, 1H), 4.24 (tq, *J* = 7.1, 3.6 Hz, 4H), 2.93 (dd, *J* = 9.2, 4.3 Hz, 2H), 2.51 (dd, *J* = 18.8, 8.6 Hz, 1H), 2.47–2.37 (m, 1H), 2.35–2.24 (m, 1H), 2.00–2.21 (m, 3H), 1.96 (dd, *J* = 9.0, 2.8 Hz, 1H), 1.76–1.39 (m, 6H), 1.28 (td, *J* = 7.1, 5.8 Hz, 6H), 0.90 (s, 3H);

^{13}C{^{1}H} NMR (126 MHz, $CDCl_3$): δ 221.2, 168.4, 168.1, 140.5, 137.1, 136.2, 134.9, 127.1, 126.3, 125.9, 125.3, 123.2, 121.7, 119.54 (q, *J* = 320.3 Hz), 62.9, 62.7, 58.8, 55.1, 50.5, 48.1, 44.5, 38.2, 35.9, 31.6, 29.5, 26.5, 25.8, 21.7, 14.0, 13.9, 13.9;

^{19}F{^{1}H} NMR (377 MHz, $CDCl_3$): δ −77.9;

HRMS (APCI) $C_{31}H_{37}F_3NO_7S$ $[M+H]^+$ found 624.2227, requires 624.2237 (−1.66 ppm).

Diethyl 4-(1-cyanocyclopropyl)-6-((trifluoromethyl)sulfonamido)cyclohexa-2,4-diene-1,1-dicarboxylate (S17)

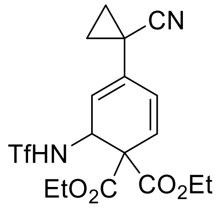

Following **General Procedure 1**, 2-methyl-2-(pyridin-4-yl)propanenitrile (144.2 mg, 1 mmol), Tf_2O (0.2 mL, 1.2 mmol), diethyl malonate (0.18 mL, 1.2 mmol), and NEt_3 (0.35 mL, 2.5 mmol) gave the crude product, which was purified by silica column chromatography (*n*-hexane/EtOAc, 100:0 to 4:1) to yield the product (393.8 mg, 90%) as a white solid.

^{1}H NMR (500 MHz, $CDCl_3$): δ 6.44 (d, *J* = 10.3 Hz, 1H), 6.17 (d, *J* = 9.8 Hz, 1H), 6.01 (d, *J* = 9.8 Hz, 1H), 5.76 (d, *J* = 4.0 Hz, 1H), 4.94 (dd, *J* = 10.3, 4.0 Hz, 1H), 4.23 (dhept, *J* = 10.6, 3.5 Hz, 4H), 1.56–1.43 (m, 2H), 1.27 (t, *J* = 7.1 Hz, 6H), 1.23–1.07 (m, 2H);
^{13}C{^{1}H} NMR (126 MHz, $CDCl_3$): δ 168.0, 167.6, 132.3, 126.9, 123.8, 123.0, 120.9, 119.5 (q, *J* = 320.4 Hz), 63.1, 63.0, 58.8, 54.3, 15.3, 15.1, 13.9, 13.9, 13.2;
^{19}F{^{1}H} NMR (377 MHz, $CDCl_3$): δ −78.0;
HRMS (APCI) $C_{17}H_{20}F_3NO_6S$ $[M+H]^+$ found, 437.0991 requires 437.0989 (+0.53 ppm).

Diethyl 4-(2-cyanopropan-2-yl)-6-((trifluoromethyl)sulfonamido)cyclohexa-2,4-diene-1,1-dicarboxylate (S18)

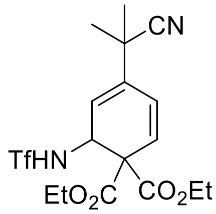

Following **General Procedure 1**, 2-methyl-2-(pyridin-4-yl)propanenitrile (146.2 mg, 1 mmol), Tf_2O (0.2 mL, 1.2 mmol), diethyl malonate (0.18 mL, 1.2 mmol), and NEt_3 (0.35 mL, 2.5 mmol) gave the crude product, which was purified by silica column chromatography (*n*-hexane/EtOAc, 100:0 to 4:1) to yield the product (390.9 mg, 89%) as a red oil.
^{1}H NMR (500 MHz, $CDCl_3$): δ 6.56 (dd, *J* = 10.3, 2.4 Hz, 1H), 6.21 (app. t, *J* = 1.2 Hz, 2H), 5.83 (d, *J* = 3.9 Hz, 1H), 4.96 (dd, *J* = 10.3, 3.9 Hz, 1H), 4.31–4.02 (m, 4H), 1.48 (s, 3H), 1.46 (s, 3H), 1.26 (td, *J* = 7.1, 3.0 Hz, 6H);
^{13}C{^{1}H} NMR (126 MHz, $CDCl_3$): δ 168.1, 167.6, 137.2, 127.0, 124.0, 122.5, 121.7, 119.5 (q, *J* = 320.5 Hz), 63.1, 62.9, 58.8, 54.4, 35.8, 25.9, 25.7, 13.9, 13.9;
^{19}F{^{1}H} NMR (377 MHz, $CDCl_3$): δ −78.0;
HRMS (APCI) $C_{17}H_{20}F_3NO_6S$ $[M-H]^-$ found 437.0990, requires 437.1000 (−2.21 ppm).

Diethyl 4-(1-(4-chlorophenyl)cyclobutyl)-6-((trifluoromethyl)sulfonamido)cyclohexa-2,4-diene-1,1-dicarboxylate (S19)

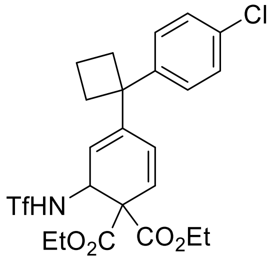

Following **General Procedure 1**, 4-(1-(4-chlorophenyl)cyclobutyl)pyridine (243.7 mg, 1 mmol), Tf_2O (0.2 mL, 1.2 mmol), diethyl malonate (0.18 mL, 1.2 mmol), and NEt_3 (0.35 mL, 2.5 mmol) gave the crude product, which was purified by silica column chromatography (*n*-hexane/EtOAc, 100:0 to 4:1) to yield the product (511.3 mg, 95%) as a white solid.

^{1}H NMR (500 MHz, $CDCl_3$): δ 7.23 (d, *J* = 8.5 Hz, 2H), 7.09 (d, *J* = 8.5 Hz, 2H), 6.44 (d, *J* = 10.2 Hz, 1H), 5.97 (d, *J* = 9.7 Hz, 1H), 5.83 (d, *J* = 4.1 Hz, 1H), 5.78 (dd, *J* = 9.7, 1.4 Hz, 1H), 4.98 (dd, *J* = 10.3, 4.1 Hz, 1H), 4.30–4.04 (m, 2H), 2.54–2.19 (m, 4H), 1.87 (p, *J* = 7.6 Hz, 2H), 1.27–1.11 (m, 6H);

^{13}C{^{1}H} NMR (126 MHz, $CDCl_3$): δ 168.4, 167.8, 144.5, 142.0, 132.1, 128.6, 127.6, 126.4, 125.4, 118.8, 62.7, 62.6, 59.0, 54.7, 49.6, 31.8, 31.6, 16.1, 13.9, 13.8;

^{19}F{^{1}H} NMR (377 MHz, $CDCl_3$): δ −78.0;

HRMS (APCI) $C_{23}H_{26}ClF_3NO_6S$ $[M+H]^+$ found, 536.1124 requires 536.1116 (+1.50 ppm).

Diethyl 1'-cyano-3-((trifluoromethyl)sulfonamido)-[1,1'-bi(cyclohexane)]-1,5-diene-4,4-dicarboxylate (S20)

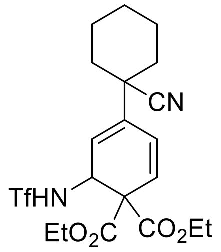

Following **General Procedure 1**, 1-(pyridin-4-yl)cyclohexane-1-carbonitrile (186.3 mg, 1 mmol), Tf_2O (0.2 mL, 1.2 mmol), diethyl malonate (0.18 mL, 1.2 mmol), and NEt_3 (0.35 mL, 2.5 mmol) gave the crude product, which was purified by silica column chromatography (*n*-hexane/EtOAc, 100:0 to 4:1) to yield the product (434.9 mg, 91%) as a white solid.

^{1}H NMR (500 MHz, $CDCl_3$): δ 6.51 (d, *J* = 10.2 Hz, 1H), 6.25 (dd, *J* = 9.8, 1.5 Hz, 1H), 6.18 (d, *J* = 9.8 Hz, 1H), 5.80 (dd, *J* = 3.9, 1.5 Hz, 1H), 4.96 (dd, *J* = 10.2, 3.9 Hz, 1H), 3.99–4.41 (m, 4H), 2.02–1.91 (m, 2H), 1.87–1.60 (m, 5H), 1.42 (tdd, *J* = 12.9, 9.2, 3.7 Hz, 2H), 1.27 (td, *J* = 7.1, 5.2 Hz, 6H), 1.23–1.07 (m, 1H);

^{13}C{^{1}H} NMR (126 MHz, $CDCl_3$): δ 168.2, 167.8, 137.2, 126.7, 124.6, 122.5, 120.9, 119.5 (q, *J* = 320.4 Hz), 63.1, 62.9, 58.8, 54.7, 42.7, 34.2, 34.1, 24.9, 23.0, 13.9;

^{19}F{^{1}H} NMR (377 MHz, $CDCl_3$): δ −78.0;

HRMS (APCI) $C_{20}H_{24}F_3N_2O_6S$ $[M+H]^+$ found, 477.1299 requires 477.1313 (−2.86 ppm).

Diethyl 1'-phenyl-3-((trifluoromethyl)sulfonamido)-[1,1'-bi(cyclohexane)]-1,5-diene-4,4-dicarboxylate (S21)

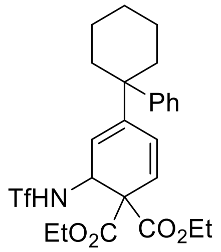

Following **General Procedure 1**, 4-(1-phenylcyclohexyl)pyridine (237.3 mg, 1 mmol), Tf_2O (0.2 mL, 1.2 mmol), diethyl malonate (0.18 mL, 1.2 mmol), and NEt_3 (0.35 mL, 2.5 mmol) gave the crude product, which was purified by silica column chromatography (*n*-hexane/EtOAc, 100:0 to 4:1) to yield the product (460.2 mg, 87%) as a white solid which was of sufficient purity for use without further purification.

^{1}H NMR (500 MHz, $CDCl_3$): δ 7.34–7.14 (m, 5H), 6.39 (d, *J* = 10.3 Hz, 1H), 5.94 (d, *J* = 10.3 Hz, 1H), 5.87–5.78 (m, 2H), 4.99 (dd, *J* = 10.3, 3.9 Hz, 1H), 4.36–4.09 (m, 2H), 2.01–1,91 (m, 4H), 1.66–1.38 (m, 6H), 1.38–1.23 (m, 6H);

^{13}C{^{1}H} NMR (126 MHz, $CDCl_3$): δ 168.6, 168.1, 142.6, 128.5, 126.9, 126.9, 126.3, 124.5, 121.2, 62.7, 62.5, 58.8, 55.0, 41.8, 34.8, 34.5, 26.2, 22.5, 14.1;

^{19}F{^{1}H} NMR (377 MHz, $CDCl_3$): δ −78.0.

Diethyl 4-(4-(methoxycarbonyl)bicyclo[2.2.2]octan-1-yl)-6-((trifluoromethyl)sulfonamido)cyclohexa-2,4-diene-1,1-dicarboxylate (S22)

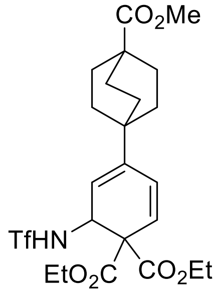

Following **General Procedure 1**, methyl 4-(pyridin-4-yl)bicyclo[2.2.2]octane-1-carboxylate (245.3 mg, 1 mmol), Tf_2O (0.2 mL, 1.2 mmol), diethyl malonate (0.18 mL, 1.2 mmol), and NEt_3 (0.35 mL, 2.5 mmol) gave the crude product, which was purified by silica column chromatography (*n*-hexane/EtOAc, 100:0 to 4:1) to yield the product (514.2 mg, 96%) as a white solid.

^{1}H NMR (500 MHz, $CDCl_3$): δ 6.32 (d, *J* = 10.2 Hz, 1H), 6.20 (dd, *J* = 9.9, 1.4 Hz, 1H), 6.04 (d, *J* = 9.9 Hz, 1H), 5.44 (d, *J* = 3.8 Hz, 1H), 4.88 (dd, *J* = 10.2, 3.8 Hz, 1H), 4.28–4.10 (m, 4H), 3.64 (s, 3H), 1.96–1.72 (m, 6H), 1.62–1.45 (m, 6H), 1.26 (dt, *J* = 8.1, 7.1 Hz, 6H);
^{13}C{^{1}H} NMR (126 MHz, $CDCl_3$): δ 178.2, 168.7, 168.4, 143.8, 125.9, 124.7, 119.7, 119.5 (q, *J* = 320.6 Hz), 62.9, 62.6, 58.7, 55.4, 51.9, 39.0, 34.2, 28.8, 28.2, 14.0;
^{19}F{^{1}H} NMR (377 MHz, $CDCl_3$): δ −78.0;
HRMS (APCI) $C_{23}H_{29}F_3NO_8S$ $[M-H]^-$ found 536.1561, requires 536.1571 (−1.95 ppm).

Diethyl 4-(3-(methoxycarbonyl)bicyclo[1.1.1]pentan-1-yl)-6-((trifluoromethyl)sulfonamido)cyclohexa-2,4-diene-1,1-dicarboxylate (S23)

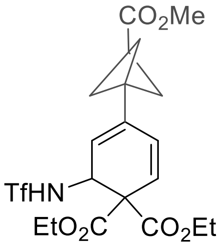

Following **General Procedure 1**, methyl 3-(pyridin-4-yl)bicyclo[1.1.1]pentane-1-carboxylate (203.2 mg, 1 mmol), Tf_2O (0.20 mL, 1.2 mmol), diethyl malonate (0.18 mL, 1.2 mmol), and NEt_3 (0.35 mL, 2.5 mmol) gave the crude product, which was purified by silica column chromatography (*n*-hexane/EtOAc, 100:0 to 4:1) to yield the product (426.9 mg, 86%) as a white solid.

^{1}H NMR (500 MHz, $CDCl_3$): δ 6.23 (d, *J* = 10.3 Hz, 1H), 5.91–6.15 (m, 2H), 5.51 (d, *J* = 3.9 Hz, 1H), 4.92 (dd, *J* = 10.3, 3.9 Hz, 1H), 4.23 (dddd, *J* = 13.4, 7.8, 5.6, 2.6 Hz, 4H), 3.68 (s, 3H), 2.10 (s, 6H), 1.26 (tt, *J* = 7.1, 1.7 Hz, 6H);
^{13}C{^{1}H} NMR (126 MHz, $CDCl_3$): δ 170.3, 168.4, 168.0, 135.4, 125.1, 124.7, 121.6, 119.5 (q, *J* = 320.5 Hz), 62.9, 62.7, 58.9, 54.5, 52.2, 51.9, 40.6, 37.4, 14.0, 13.9;
^{19}F{^{1}H} NMR (377 MHz, $CDCl_3$): δ −78.0.

Diethyl 4-(trifluoromethyl)-6-((trifluoromethyl)sulfonamido)cyclohexa-2,4-diene-1,1-dicarboxylate (S24)

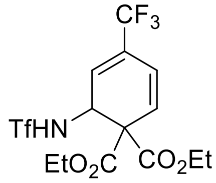

Following **General Procedure 1**, 4-(trifluoromethyl)pyridine (116.0 μL, 1 mmol), Tf_2O (0.2 mL, 1.2 mmol), diethyl malonate (0.18 mL, 1.2 mmol), and NEt_3 (0.35 mL, 2.5 mmol) gave the crude product, which was purified by silica column chromatography (*n*-hexane/EtOAc, 100:0 to 4:1) to yield the product (111.3 mg, 25%) as an orange solid.

^{1}H NMR (500 MHz, $CDCl_3$): δ 6.30 (s, 1H), 6.23 (d, *J* = 9.8 Hz, 1H), 6.17 (d, *J* = 9.8 Hz, 1H), 5.22–4.90 (m, 1H), 4.32–4.12 (m, 4H), 1.26 (td, *J* = 7.3, 5.2 Hz, 6H);

^{13}C{^{1}H} NMR (126 MHz, $CDCl_3$): δ 167.8, 167.3, 128.2 (q, *J* = 33.1 Hz), 128.0 (q, *J* = 5.9 Hz), 127.6, 121.9 (q, *J* = 271.9 Hz), 120.4 (d, *J* = 2.3 Hz), 119.5 (q, *J* = 320.2 Hz), 63.4, 63.2, 58.8, 53.4, 13.8, 13.8;

^{19}F{^{1}H} NMR (377 MHz, $CDCl_3$): δ −69.1, −78.1;

HRMS (APCI) $C_{14}H_{16}F_6NO_6S$ $[M+H]^+$ found 440.0600, requires 440.0597 (+0.67 ppm).

Diethyl 4-(2-ethoxy-1,1-difluoro-2-oxoethyl)-6-((trifluoromethyl)sulfonamido)cyclohexa-2,4-diene-1,1-dicarboxylate (S25)

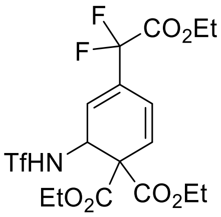

Following **General Procedure 1**, ethyl 2,2-difluoro-2-(pyridin-4-yl)acetate (201.2 mg, 1 mmol), Tf_2O (0.2 mL, 1.2 mmol), diethyl malonate (0.18 mL, 1.2 mmol), and NEt_3 (0.35 mL, 2.5 mmol) gave the crude product, which was purified by silica column chromatography (*n*-hexane/EtOAc, 100:0 to 4:1) to yield the product (253.7 mg, 51%) as a yellow oil of sufficient purity for use without further purification.

^{1}H NMR (500 MHz, $CDCl_3$): δ 6.65 (d, *J* = 10.2 Hz, 1H), 5.99–6.40 (m, 3H), 5.04 (dq, *J* = 10.2, 3.2 Hz, 1H), 4.52–3.96 (m, 6H), 1.49–1.18 (m, 9H);

^{13}C{^{1}H} NMR (126 MHz, $CDCl_3$): δ 167.8, 167.4, 162.8 (t, *J* = 34.0 Hz), 130.5 (t, *J* = 25.2 Hz), 127.6 (t, *J* = 8.4 Hz), 126.7, 121.3 (t, *J* = 4.3 Hz), 119.5 (q, *J* = 320.2 Hz), 111.4 (t, *J* = 252.2 Hz), 63.6, 63.2, 63.1, 58.8, 53.8, 13.9, 13.8, 13.8;

^{19}F{^{1}H} NMR (377 MHz, $CDCl_3$): δ −108.8, −108.6, −78.1.

Diethyl 6-chloro-3-((trifluoromethyl)sulfonamido)-[1,1'-biphenyl]-4,4(3H)-dicarboxylate (S26)

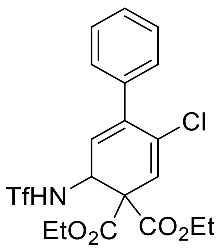

Following **General Procedure 1**, 3-chloro-4-phenylpyridine (189.6 mg, 1 mmol), Tf_2O (0.2 mL, 1.2 mmol), diethyl malonate (0.18 mL, 1.2 mmol), and NEt_3 (0.35 mL, 2.5 mmol) gave the crude product, which was purified by silica column chromatography (*n*-hexane/EtOAc, 100:0 to 4:1) to yield the product (191.8 mg, 40%) as a white solid which was used without further purification.

^{1}H NMR (500 MHz, $CDCl_3$): δ 7.47–7.30 (m, 3H), 7.30–7.14 (m, 2H), 6.39 (s, 1H), 5.99 (d, *J* = 4.1, 1H), 5.13 (dd, *J* = 10.2, 4.1, 1H), 4.38–4.26 (m, 4H), 1.42–1.27 (m, 6H);

^{13}C{^{1}H} NMR (126 MHz, $CDCl_3$): δ 167.7, 167.0, 138.7, 137.8, 136.0, 131.9, 128.6, 128.5, 128.2, 127.8, 127.0, 122.8, 119.5 (q, *J* = 320.5) 63.3, 63.1, 61.6, 60.5, 54.2, 41.7, 21.3, 14.1, 13.9, 13.9;

^{19}F{^{1}H} NMR (377 MHz, $CDCl_3$): δ −77.9;

HRMS (APCI) $C_{19}H_{20}ClF_3NO_6S$ $[M+H]^+$ found 480.0512, requires 580.0501 (+2.30 ppm).

Diethyl 3-chloro-4-(thiophen-3-yl)-6-((trifluoromethyl)sulfonamido)cyclohexa-2,4-diene-1,1-dicarboxylate (S27)

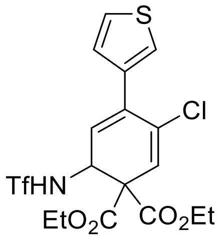

Following **General Procedure 1**, 3-chloro-4-(thiophen-3-yl)pyridine (195.7 mg, 1 mmol), Tf_2O (0.2 mL, 1.2 mmol), diethyl malonate (0.18 mL, 1.2 mmol), and NEt_3 (0.35 mL, 2.5 mmol) gave the crude product, which was purified by silica column chromatography (*n*-hexane/EtOAc, 100:0 to 4:1) to yield the product (225.9 mg, 46%) as a white solid.

^{1}H NMR (500 MHz, $CDCl_3$): δ 7.37–7.26 (m, 2H), 7.10–6.95 (m, 1H), 6.58 (d, *J* = 10.2, 1H), 6.33 (s, 1H), 6.02 (d, *J* = 3.9, 1H), 5.07 (dd, *J* = 10.2, 3.9, 1H), 4.28 (ttd, *J* = 7.1, 4.4, 2.3, 4H), 1.30 (q, *J* = 7.2, 6H);

^{13}C{^{1}H} NMR (126 MHz, $CDCl_3$): δ 167.7, 167.1, 136.2, 133.4, 131.7, 127.9, 127.3, 125.5, 124.7, 122.7, 119.5 (q, *J* = 320.4). 63.4, 63.2, 60.3, 54.3, 31.7, 22.8, 13.9, 13.9;

$^{19}F\{^1H\}$ **NMR** (377 MHz, CDCl$_3$): δ −77.9;

HRMS (APCI) $C_{17}H_{17}ClNO_6S_2Na$ [M+H]$^+$ found 510.0019, requires 510.0030 (−2.18 ppm).

Diethyl 4-(1,3-diethyl-2,6-dioxopiperidin-3-yl)-6-((trifluoromethyl)sulfonamido)cyclohexa-2,4-diene-1,1-dicarboxylate (S28)

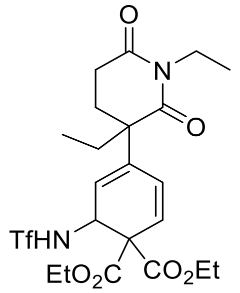

Following **General Procedure 1**, 1,3-diethyl-3-(pyridin-4-yl)piperidine-2,6-dione (246.3 mg, 1 mmol), Tf_2O (0.2 mL, 1.2 mmol), diethyl malonate (0.18 mL, 1.2 mmol), and NEt_3 (0.35 mL, 2.5 mmol) gave the crude product, which was purified by silica column chromatography (*n*-hexane/EtOAc, 100:0 to 4:1) to yield the product (512.4 mg, 95%) as a white solid as a mixture of diastereomers (77:23 dr).

^1H **NMR** (500 MHz, CDCl$_3$): δ 6.69 (d, J = 10.2 Hz, 1H, minor diastereomer), 6.61 (d, J = 10.2 Hz, 1H, major diastereomer), 6.40–5.88 (m, 2H), 5.53 (d, J = 3.9 Hz, 1H), 5.05–4.77 (m, 1H), 4.20 (p, J = 7.2 Hz, 4H), 3.93–3.49 (m, 2H), 2.70–2.53 (m, 1H), 2.50–2.30 (m, 1H), 2,04–1.86 (m, 2H), 1.85–1.56 (m, 2H), 1.24 (dt, J = 8.3, 7.2 Hz, 6H), 1.07 (td, J = 7.0, 3.0 Hz, 3H), 0.84 (td, J = 7.4, 2.8 Hz, 3H);

$^{13}C\{^1H\}$ **NMR** (126 MHz, CDCl$_3$): δ 173.7, 173.6, 171.5, 171.5, 168.3, 168.1, 167.7, 135.8, 126.6, 126.5, 124.6, 124.4, 124.2, 119.5 (q, J = 320.4 Hz), 63.1, 63.1, 62.9, 62.9, 58.6, 58.6, 54.5, 54.4, 50.1, 49.9, 35.5, 29.7, 29.6, 29.4, 29.3, 24.4, 24.3, 13.9, 13.9, 13.1, 8.7, 8.6;

$^{19}F\{^1H\}$ **NMR** (377 MHz, CDCl$_3$): δ −78.0;

HRMS (APCI) $C_{22}H_{29}F_3N_2O_8SNa$ [M+Na]$^+$ found 561.1487, requires 561.1489 (−0.34 ppm).

9 Synthesis of Benzenes via CACR

Dimethyl [1,1'-biphenyl]-3,4-dicarboxylate (8)

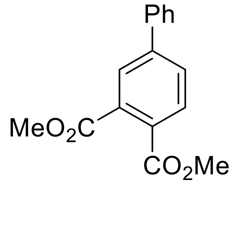

Following **General Procedure 2**, carbocycle **4** (89.5 mg, 0.2 mmol) and DMAD (73.7 µL, 0.6 mmol) gave the crude product, which was purified by silica column chromatography (*n*-hexane/EtOAc, 100:0 to 4:1) to yield the product (48.0 mg, 88%) as a white solid.
^{1}H NMR (500 MHz, $CDCl_3$): δ 7.91 (d, J = 1.9 Hz, 1H), 7.84 (d, J = 8.0 Hz, 1H), 7.75 (dd, J = 8.0, 1.9 Hz, 1H), 7.67–7.56 (m, 2H), 7.55–7.44 (m, 2H), 7.44–7.36 (m, 1H), 3.94 (s, 3H), 3.93 (s, 3H);
^{13}C{^{1}H} NMR (126 MHz, $CDCl_3$): δ 168.5, 167.8, 144.5, 139.1, 133.2, 130.0, 129.9, 129.5, 129.2, 128.6, 127.5, 127.4, 52.9, 52.8;
HRMS (APCI) $C_{16}H_{15}O_4$ $[M+H]^+$ found 271.0960, requires 271.0965 (−1.79 ppm).

Dimethyl 4-(tert-butyl)phthalate (9)

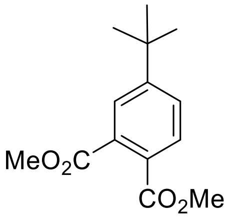

Following **General Procedure 2**, carbocycle **16** (85.5 mg, 0.2 mmol) and DMAD (73.7 µL, 0.6 mmol) gave the crude product, which was purified by silica column chromatography (*n*-hexane/EtOAc, 100:0 to 4:1) to yield the product (43.7 mg, 87%) as a white solid.
^{1}H NMR (500 MHz, $CDCl_3$): δ 7.70 (d, J = 8.2 Hz, 1H), 7.67 (d, J = 2.0 Hz, 1H), 7.54 (dd, J = 8.2, 2.0 Hz, 1H), 3.91 (s, 3H), 3.89 (s, 3H);
^{13}C{^{1}H} NMR (126 MHz, $CDCl_3$): δ 169.0, 167.9, 155.3, 132.5, 129.2, 128.5, 128.0, 125.8, 52.8, 52.6, 35.2, 31.1;
HRMS (APCI) $C_{14}H_{19}O_4$ $[M+H]^+$ found 251.1271, requires 251.1278 (−2.73 ppm).

Dimethyl 4-((3r,5r,7r)-adamantan-1-yl)phthalate (10)

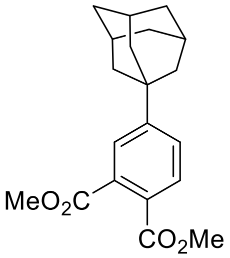

Following **General Procedure 2**, carbocycle **S11** (101.1 mg, 0.2 mmol) and DMAD (73.7 µL, 0.6 mmol) gave the crude product, which was purified by silica column chromatography (*n*-hexane/EtOAc, 100:0 to 4:1) to yield the product (62.6 mg, 95%) as a white solid.

^{1}**H NMR** (500 MHz, $CDCl_3$): δ 7.70 (d, *J* = 8.2 Hz, 1H), 7.65 (d, *J* = 2.0 Hz, 1H), 7.51 (dd, *J* = 8.2, 2.0 Hz, 1H), 3.90 (s, 3H), 3.88 (s, 3H), 2.20–2.04 (m, 3H), 1.90 (d, *J* = 2.9 Hz, 6H), 1.83–1.66 (m, 6H);

^{13}**C{^{1}H} NMR** (126 MHz, $CDCl_3$): δ 169.1, 168.0, 155.3, 132.5, 129.2, 128.5, 127.7, 127.6, 125.6, 125.5, 52.7, 52.6, 42.9, 36.7, 36.7, 28.8;

HRMS (APCI) $C_{20}H_{25}O_4$ $[M+H]^+$ found 329.1745, requires 329.1747 (−0.72 ppm).

Dimethyl 4-(thiophen-3-yl)phthalate (11)

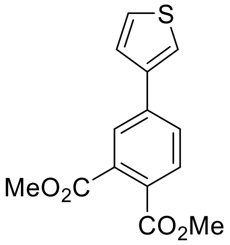

Following **General Procedure 2**, carbocycle **S12** (90.7 mg, 0.2 mmol) and DMAD (73.7 μL, 0.6 mmol) gave the crude product, which was purified by silica column chromatography (*n*-hexane/EtOAc, 100:0 to 4:1) to yield the product (41.0 mg, 74%) as a white solid.

^{1}**H NMR** (500 MHz, $CDCl_3$): δ 7.88 (d, *J* = 1.8 Hz, 1H), 7.81 (d, *J* = 8.1 Hz, 1H), 7.74 (dd, *J* = 8.1, 1.8 Hz, 1H), 7.58 (dd, *J* = 2.7, 1.6 Hz, 1H), 7.48–7.36 (m, 2H), 3.94 (s, 3H), 3.92 (s, 3H);

^{13}**C{^{1}H} NMR** (126 MHz, $CDCl_3$): δ 168.5, 167.5, 140.2, 138.9, 133.4, 129.9, 129.3, 128.4, 127.0, 126.5, 126.0, 122.4, 52.8, 52.7;

HRMS (APCI) $C_{14}H_{13}O_4S$ $[M+H]^+$ found 277.0528, requires 277.0529 (−0.38 ppm).

Dimethyl 4'-cyano-[1,1'-biphenyl]-3,4-dicarboxylate (12)

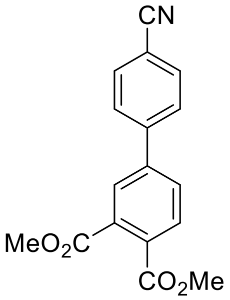

Following **General Procedure 2**, carbocycle **S13** (94.5 mg, 0.2 mmol) and DMAD (73.7 μL, 0.6 mmol) gave the crude product, which was purified by silica column chromatography (*n*-hexane/EtOAc, 100:0 to 4:1) to yield the product (47.8 mg, 81%) as a white solid.

^{1}**H NMR** (500 MHz, $CDCl_3$): δ 7.91 (d, *J* = 1.9 Hz, 1H), 7.86 (d, *J* = 8.0 Hz, 1H), 7.82–7.62 (m, 5H), 3.94 (s, 3H), 3.94 (s, 3H);

^{13}**C{^{1}H} NMR** (126 MHz, $CDCl_3$): δ 168.0, 167.6, 143.5, 142.2, 133.2, 133.0, 131.6, 130.1, 129.7, 128.0, 127.8, 118.6, 112.3, 53.1, 53.0;

HRMS (APCI) $C_{17}H_{14}O_4N$ $[M+H]^+$ found 296.0908, requires 296.0917 (−3.16 ppm).

Dimethyl 4'-methoxy-[1,1'-biphenyl]-3,4-dicarboxylate (13)

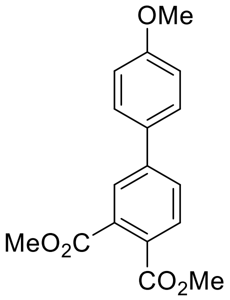

Following **General Procedure 2**, carbocycle **S14** (95.5 mg, 0.2 mmol) and DMAD (73.7 µL, 0.6 mmol) gave the crude product, which was purified by silica column chromatography (*n*-hexane/EtOAc, 100:0 to 4:1) to yield the product (24.4 mg, 41%) as a white solid.

^{1}**H NMR** (500 MHz, $CDCl_3$): δ 7.84 (d, *J* = 1.9 Hz, 1H), 7.82 (d, *J* = 8.2 Hz, 1H), 7.70 (dd, *J* = 8.1, 1.9 Hz, 1H), 7.56 (d, *J* = 8.9 Hz, 1H), 6.99 (d, *J* = 8.9 Hz, 1H), 3.94 (s, 3H), 3.92 (s, 3H), 3.86 (s, 3H);

^{13}**C{^{1}H} NMR** (126 MHz, $CDCl_3$): δ 168.9, 167.9, 160.2, 144.2, 133.4, 131.5, 129.9, 129.0, 128.8, 128.5, 126.8, 114.6, 55.5, 52.9, 52.8;

HRMS (APCI) $C_{17}H_{17}O_5$ $[M+H]^+$ found 301.1056, requires 301.1071 (−4.82 ppm).

Dimethyl 6-bromo-[1,1'-biphenyl]-3,4-dicarboxylate (14)

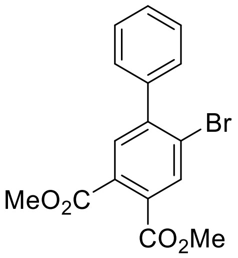

Following **General Procedure 2**, carbocycle **S15** (105.3 mg, 0.2 mmol) and DMAD (73.7 µL, 0.6 mmol) gave the crude product, which was purified by silica column chromatography (*n*-hexane/Et_2O, 100:0 to 4:1) to yield the product (49.7 mg, 71%) as a colourless oil.

^{1}H NMR (500 MHz, $CDCl_3$): δ 8.04 (s, 1H), 7.68 (s, 1H), 7.56–7.35 (m, 5H), 3.94 (s, 3H), 3.90 (s, 3H);

^{13}C{^{1}H} NMR (126 MHz, $CDCl_3$): δ 167.3, 166.7, 145.7, 139.4, 133.8, 131.9, 131.6, 131.1, 129.2, 128.6, 128.4, 125.8, 53.1, 53.0;

HRMS (APCI) $C_{16}H_{14}O_4Br$ $[M+H]^+$ found 349.0085, requires 349.0070 (+4.30 ppm).

Dimethyl 4-((8R,9S,13R,14R)-14-methyl-15-oxo-7,8,9,11,12,13,14,15,16,17-decahydro-6H-cyclopenta[a]phenanthren-2-yl)phthalate (15)

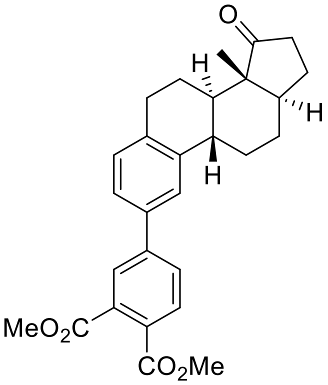

Following **General Procedure 2**, carbocycle **S16** (124.7 mg, 0.2 mmol) and DMAD (73.7 µL, 0.6 mmol) gave the crude product, which was purified by silica column chromatography (*n*-hexane/Et_2O, 100:0 to 4:1) to yield the product (58.2 mg, 65%) as a colourless oil.

^{1}H NMR (500 MHz, $CDCl_3$): δ 7.88 (d, *J* = 1.8 Hz, 1H), 7.82 (d, *J* = 8.1 Hz, 1H), 7.73 (dd, *J* = 8.1, 1.8 Hz, 1H), 7.32–7.48 (m, 3H), 3.94 (s, 3H), 3.92 (s, 3H), 3.08–2.91 (m, 2H), 2.64–2.42 (m, 2H), 2.35 (td, *J* = 10.9, 4.1 Hz, 1H), 2.28–1.88 (m, 4H), 1.76–1.37 (m, 6H), 0.93 (s, 3H);

^{13}C{^{1}H} NMR (126 MHz, $CDCl_3$): δ 220.9, 168.6, 167.8, 144.3, 140.5, 137.4, 136.5, 133.2, 129.8, 129.7, 129.1, 127.8, 127.2, 126.2, 124.6, 52.9, 52.7, 50.6, 48.1, 44.5, 38.2, 36.0, 31.7, 29.6, 26.5, 25.8, 21.7, 14.0;

HRMS (APCI) $C_{28}H_{31}O_5$ $[M+H]^+$ found 447.2166, requires 417.2166 (0.00 ppm).

10 Pyridine N-Transposition via CACR

5-(Tert-butyl)-2-tosylpyridine (20)

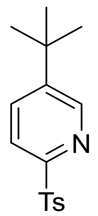

Following **General Procedure 2**, carbocycle **18** (84.4 mg, 0.2 mmol) and tosyl cyanide (108.8 mg, 0.6 mmol) gave the crude product which was purified by silica column chromatography (*n*-hexane/EtOAc, 100:0 to 1:1) to yield the product (18.8 mg, 32%) as a white solid.

^{1}H NMR (500 MHz, $CDCl_3$): δ 8.71 (dd, *J* = 2.4, 0.8 Hz, 1H), 8.09 (dd, *J* = 8.3, 0.8 Hz, 1H), 7.93 (d, *J* = 8.2 Hz, 2H), 7.87 (dd, *J* = 8.3, 2.4 Hz, 1H), 7.33 (d, *J* = 8.2 Hz, 2H), 2.41 (s, 3H), 1.33 (s, 9H);

^{13}C{^{1}H} NMR (126 MHz, $CDCl_3$): δ 156.1, 150.3, 148.6, 144.9, 136.3, 135.0, 130.0, 129.0, 121.8, 34.3, 30.9, 21.8;

HRMS (APCI) $C_{16}H_{20}O_2NS$ $[M+H]^+$ found 290.1196, requires 290.1209 (−4.57 ppm).

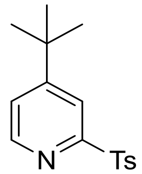

The alternative regioisomer **21** was also isolated (19.8 mg, 34%) as a colourless oil.

^{1}H NMR (500 MHz, $CDCl_3$): δ 8.54 (dd, *J* = 5.1, 0.7 Hz, 1H), 8.18 (dd, *J* = 1.9, 0.7 Hz, 1H), 7.94 (d, *J* = 8.2 Hz, 2H), 7.40 (dd, *J* = 5.1, 1.9 Hz, 1H), 7.32 (d, *J* = 8.2 Hz, 2H), 2.40 (s, 3H), 1.34 (s, 9H);

^{13}C{^{1}H} NMR (126 MHz, $CDCl_3$): δ 163.1, 159.2, 150.4, 144.8, 136.2, 129.8, 129.1, 123.9, 119.0, 35.5, 30.5, 21.8;

HRMS (APCI) $C_{16}H_{20}O_2NS$ $[M+H]^+$ found 290.1219, requires 290.1209 (+3.36 ppm).

1-(6-Tosylpyridin-3-yl)cyclopropane-1-carbonitrile (22)

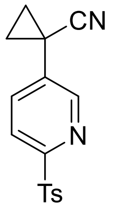

Following **General Procedure 2**, carbocycle **S17** (87.2 mg, 0.2 mmol) and tosyl cyanide (108.8 mg, 0.6 mmol) gave the crude product which was purified by silica column chromatography (*n*-hexane/EtOAc, 100:0 to 1:1) to yield the product (21.1 mg, 35%) as an orange solid.

^{1}**H NMR** (500 MHz, $CDCl_3$): δ 8.55 (dd, *J* = 2.4, 0.8 Hz, 1H), 8.16 (dd, *J* = 8.2, 0.8 Hz, 1H), 7.92 (d, *J* = 8.4 Hz, 2H), 7.82 (dd, *J* = 8.2, 2.4 Hz, 1H), 7.33 (dd, *J* = 8.4, 0.7 Hz, 2H), 2.42 (s, 3H), 2.00–1.78 (m, 2H), 1.68–1.36 (m, 2H);

^{13}**C{^{1}H} NMR** (126 MHz, $CDCl_3$): δ 158.3, 147.6, 145.3, 136.1, 135.6, 135.4, 130.0, 129.1, 122.0, 120.6, 21.8, 19.4, 12.2;

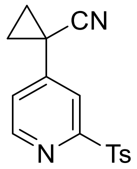

The alternative regioisomer **S29** was also isolated (20.4 mg, 34%) as an orange oil.

^{1}**H NMR** (500 MHz, $CDCl_3$): δ 8.61 (dd, *J* = 5.1, 0.7 Hz, 1H), 7.93 (d, *J* = 8.4 Hz, 2H), 7.84 (dd, *J* = 2.0, 0.7 Hz, 1H), 7.49 (dd, *J* = 5.1, 2.0 Hz, 1H), 7.34 (d, *J* = 8.4 Hz, 2H), 2.42 (s, 3H), 2.02–1.92 (m, 2H), 1.65–1.60 (m, 2H);

^{13}**C{^{1}H} NMR** (126 MHz, $CDCl_3$): δ 160.2, 150.9, 148.9, 145.4, 135.4, 130.0, 129.2, 123.7, 120.1, 116.6, 21.9, 21.0, 14.1;

HRMS (APCI) $C_{16}H_{15}O_2N_2S$ $[M+H]^+$ found 299.0857, requires 299.0849 (+2.76 ppm).

2-Methyl-2-(6-tosylpyridin-3-yl)propanenitrile (23)

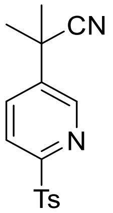

Following **General Procedure 2**, carbocycle **S18** (87.7 mg, 0.2 mmol) and tosyl cyanide (108.8 mg, 0.6 mmol) gave the crude product which was purified by silica column chromatography (*n*-hexane/EtOAc, 100:0 to 1:1) to yield the product (25.9 mg, 43%) as a white solid. Note: Two columns were required to enable complete separation of the regioisomers. To clearly distinguish the regioisomers by TLC, the plate was run twice in 1:1 *n*-hexane/EtOAc.

^{1}**H NMR** (500 MHz, $CDCl_3$): δ 8.77 (dd, *J* = 2.4, 0.8 Hz, 1H), 8.20 (dd, *J* = 8.3, 0.8 Hz, 1H), 8.02 (dd, *J* = 8.3, 2.4 Hz, 1H), 7.93 (d, *J* = 8.2 Hz, 2H), 7.34 (d, *J* = 8.2 Hz, 2H), 2.41 (s, 3H), 1.74 (s, 6H);

^{13}**C{^{1}H} NMR** (126 MHz, $CDCl_3$): δ 158.8, 147.8, 145.3, 140.7, 135.6, 135.3, 130.0, 129.1, 122.6, 122.1, 35.9, 28.8, 21.8;

HRMS (APCI) $C_{16}H_{17}O_2N_2S$ $[M+H]^+$ found 301.1004, requires 301.1005 (−0.42 ppm).

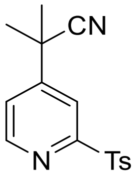

The alternative regioisomer **S30** was also isolated (17.4 mg, 29%) as a colourless oil.

^{1}H NMR (500 MHz, $CDCl_3$): δ 8.68 (d, *J* = 5.1 Hz, 1H), 8.22 (d, *J* = 1.9 Hz, 1H), 7.95 (d, *J* = 8.2 Hz, 2H), 7.60 (dd, *J* = 5.1, 1.9 Hz, 1H), 7.35 (d, *J* = 8.2 Hz, 2H), 2.42 (s, 3H), 1.77 (s, 6H);

^{13}C{^{1}H} NMR (126 MHz, $CDCl_3$): δ 160.4, 153.2, 151.4, 145.4, 135.4, 130.0, 129.3, 123.7, 122.4, 118.1, 37.5, 28.5, 21.8;

HRMS (APCI) $C_{16}H_{17}N_2O_2S$ $[M+H]^+$ found 301.1004, requires 301.1005 (−0.42 ppm).

5-(1-(4-Chlorophenyl)cyclobutyl)-2-tosylpyridine (24)

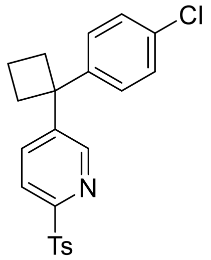

Following **General Procedure 2**, carbocycle **S19** (107.0 mg, 0.2 mmol) and tosyl cyanide (108.8 mg, 0.6 mmol) gave the crude product which was purified by silica column chromatography (*n*-hexane/EtOAc, 100:0 to 4:1) to yield the product (42.5 mg, 53%) as a colourless oil.

^{1}H NMR (500 MHz, $CDCl_3$): δ 8.60 (dd, *J* = 2.3, 0.8 Hz, 1H), 8.09 (dd, *J* = 8.2, 0.8 Hz, 1H), 7.94 (d, *J* = 8.3 Hz, 2H), 7.76 (dd, *J* = 8.2, 2.3 Hz, 1H), 7.33 (d, *J* = 7.6 Hz, 2H), 7.29–7.24 (m, 2H), 7.20–7.15 (m, 2H), 2.84–2.64 (m, 4H), 2.42 (s, 3H), 2.01 (dddd, *J* = 15.4, 8.3, 6.8, 3.7 Hz, 2H);

^{13}C{^{1}H} NMR (126 MHz, $CDCl_3$): δ 156.5, 149.1, 148.3, 145.7, 144.9, 136.1, 135.4, 132.4, 129.9, 129.1, 129.0, 127.7, 121.8, 49.3, 34.8, 21.8, 16.8;

HRMS (APCI) $C_{22}H_{21}O_2NClS$ $[M+H]^+$ found 398.0971, requires 398.0976 (−1.27 ppm).

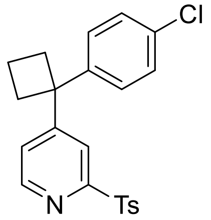

The alternative regioisomer **S31** was also isolated (22.2 mg, 28%) as a colourless oil.

^{1}H NMR (500 MHz, $CDCl_3$): δ 8.51 (d, *J* = 5.0 Hz, 1H), 8.13 (d, *J* = 1.8 Hz, 1H), 7.96–7.88 (m, 2H), 7.32 (d, *J* = 8.1 Hz, 2H), 7.30–7.23 (m, 3H), 7.22–7.16 (m, 2H), 2.83–2.65 (m, 4H), 2.41 (s, 3H), 2.01 (ttd, *J* = 8.4, 6.8, 5.0 Hz, 2H);
$^{13}C\{^1H\}$ NMR (126 MHz, $CDCl_3$): δ 161.0, 159.5, 150.6, 145.2, 145.0, 135.9, 132.6, 129.9, 129.2, 129.0, 127.7, 124.5, 119.3, 50.8, 34.8, 21.8, 16.7;
HRMS (APCI) $C_{22}H_{21}O_2NClS$ $[M+H]^+$ found 398.0978, requires 398.0976 (+0.49 ppm).

1-(6-Tosylpyridin-3-yl)cyclohexane-1-carbonitrile (25)

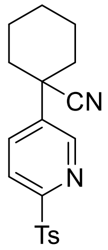

Following **General Procedure 2**, carbocycle **S20** (95.7 mg, 0.2 mmol) and tosyl cyanide (108.8 mg, 0.6 mmol) gave the crude product which was purified by silica column chromatography (*n*-hexane/EtOAc, 100:0 to 4:1) to yield the product (34.9 mg, 51%) as a colourless oil.
^{1}H NMR (500 MHz, $CDCl_3$): δ 8.78 (dd, *J* = 2.4, 0.8 Hz, 1H), 8.20 (dd, *J* = 8.3, 0.8 Hz, 1H), 8.02 (dd, *J* = 8.3, 2.4 Hz, 1H), 7.94 (d, *J* = 8.2 Hz, 2H), 7.34 (d, *J* = 8.2 Hz, 2H), 2.42 (s, 3H), 2.16–2.10 (m, 2H), 1.94–1.63 (m, 8H);
$^{13}C\{^1H\}$ NMR (126 MHz, $CDCl_3$): δ 158.7, 148.3, 145.3, 140.7, 135.7, 135.7, 130.0, 129.2, 122.1, 120.9, 43.1, 37.1, 24.7, 23.4, 21.8;
HRMS (APCI) $C_{16}H_{15}O_2N_2S$ $[M+H]^+$ found 299.0857, requires 299.0849 (+2.76 ppm).

5-(1-Phenylcyclohexyl)-2-tosylpyridine (26)

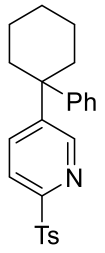

Following **General Procedure 2**, carbocycle **S21** (105.9 mg, 0.2 mmol) and tosyl cyanide (108.8 mg, 0.6 mmol) gave the crude product which was purified by silica column chromatography (*n*-hexane/EtOAc, 100:0 to 4:1) to yield the product (50.3 mg, 64%) as a colourless oil.

^{1}H NMR (500 MHz, $CDCl_3$): δ 8.57 (d, *J* = 2.4 Hz, 1H), 8.05 (dd, *J* = 8.4, 0.7 Hz, 1H), 7.93 (d, *J* = 8.4 Hz, 2H), 7.74 (dd, *J* = 8.4, 2.4 Hz, 1H), 7.45–7.22 (m, 6H), 7.21–7.12 (m, 1H), 2.42 (s, 3H), 2.40–2.32 (m, 2H), 2.28–2.14 (m, 2H), 1.62–1.47 (m, 6H);
^{13}C{^{1}H} NMR (126 MHz, $CDCl_3$): δ 156.0, 150.0, 148.6, 145.5, 144.9, 136.5, 136.1, 129.9, 129.1, 128.9, 127.3, 126.4, 121.7, 45.5, 36.7, 26.1, 22.6, 21.8;
HRMS (APCI) $C_{24}H_{26}O_2NS$ $[M+H]^+$ found 392.1663, requires 392.1679 (−4.02 ppm).

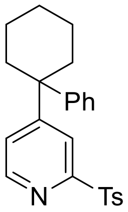

The alternative regioisomer **S32** was also isolated (14.7 mg, 19%) as a colourless oil.
^{1}H NMR (500 MHz, $CDCl_3$): δ 8.47 (d, *J* = 5.1 Hz, 1H), 8.15 (d, *J* = 1.8 Hz, 1H), 7.90 (d, *J* = 8.4 Hz, 2H), 7.34–7.28 (m, 4H), 7.25–7.16 (m, 3H), 2.41 (s, 3H), 2.39–2.30 (m, 2H), 2.19–2.30 (m, 2H), 1.61–1.48 (m, 6H);
^{13}C{^{1}H} NMR (126 MHz, $CDCl_3$): δ 159.3, 150.6, 145.2, 144.8, 136.2, 129.9, 129.1, 129.0, 127.2, 126.6, 125.8, 120.3, 46.8, 36.4, 26.1, 22.7, 21.8;
HRMS (APCI) $C_{24}H_{26}O_2NS$ $[M+H]^+$ found 392.1662, requires 392.1679 (−4.27 ppm).

5-((3r,5r,7r)-Adamantan-1-yl)-2-tosylpyridine (27)

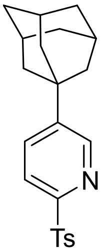

Following **General Procedure 2**, carbocycle **S11** (101.1 mg, 0.2 mmol) and tosyl cyanide (108.8 mg, 0.6 mmol) gave the crude product which was purified by silica column chromatography (*n*-hexane/EtOAc, 100:0 to 1:1) to yield the product (22.2 mg, 30%) as a white solid.
^{1}H NMR (500 MHz, $CDCl_3$): δ 8.66 (d, *J* = 2.3 Hz, 1H), 8.09 (d, *J* = 8.3 Hz, 1H), 7.94 (d, *J* = 8.3 Hz, 2H), 7.82 (dd, *J* = 8.3, 2.4 Hz, 1H), 7.32 (d, *J* = 8.3 Hz, 2H), 2.40 (s, 3H), 2.14–1.97 (m, 3H), 1.88 (d, *J* = 2.9 Hz, 6H), 1.83–1.74 (m, 3H), 1.75–1.60 (m, 3H);
^{13}C{^{1}H} NMR (126 MHz, $CDCl_3$): δ 156.1, 150.1, 148.4, 144.7, 136.4, 134.6, 129.9, 129.0, 121.8, 42.6, 36.4, 35.8, 28.6, 21.8;
HRMS (APCI) $C_{22}H_{26}O_2NS$ $[M+H]^+$ found 368.1688, requires 368.1679 (+2.51 ppm).

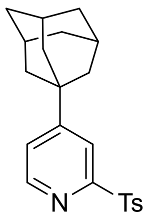

The alternative regioisomer **S33** was also isolated (26.4 mg, 36%) as a white solid.

^{1}H NMR (500 MHz, $CDCl_3$): δ 8.55 (d, *J* = 5.1 Hz, 1H), 8.16 (d, *J* = 1.8 Hz, 1H), 7.93 (d, *J* = 8.2 Hz, 2H), 7.37 (dd, *J* = 5.1, 1.8 Hz, 1H), 7.32 (d, *J* = 8.2 Hz, 2H), 2.40 (s, 3H), 2.23–2.04 (m, 3H), 1.89 (d, *J* = 2.9 Hz, 6H), 1.85–1.77 (m, 3H), 1.79–1.68 (m, 3H);

^{13}C{^{1}H} NMR (126 MHz, $CDCl_3$): δ 163.1, 159.0, 150.5, 144.8, 136.3, 129.9, 129.0, 123.6, 118.9, 42.3, 37.1, 36.4, 28.5, 21.8;

HRMS (APCI) $C_{22}H_{26}O_2NS$ $[M+H]^+$ found 368.1670, requires 368.1679 (−2.38 ppm).

Methyl 4-(6-tosylpyridin-3-yl)bicyclo[2.2.2]octane-1-carboxylate (28)

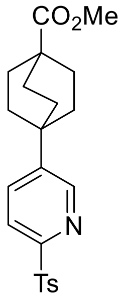

Following **General Procedure 2**, carbocycle **S22** (107.5 mg, 0.2 mmol) and tosyl cyanide (108.8 mg, 0.6 mmol) gave the crude product which was purified by silica column chromatography (*n*-hexane/Et_2O, 1:1 to 1:4) to yield the product (25.0 mg, 31%) as a white solid.

^{1}H NMR (500 MHz, $CDCl_3$): δ 8.62 (d, *J* = 2.4 Hz, 1H), 8.08 (d, *J* = 8.2 Hz, 1H), 7.93 (d, *J* = 8.2 Hz, 2H), 7.79 (dd, *J* = 8.2, 2.4 Hz, 1H), 7.32 (d, *J* = 8.2 Hz, 2H), 3.66 (s, 3H), 2.40 (s, 3H), 2.03–1.88 (m, 6H), 1.88–1.69 (m, 6H);

^{13}C{^{1}H} NMR (126 MHz, $CDCl_3$): δ 177.8, 156.5, 148.7, 148.3, 144.8, 136.3, 135.2, 129.9, 129.1, 129.0, 121.7, 52.0, 39.0, 34.3, 31.4, 28.4, 21.8;

HRMS (APCI) $C_{22}H_{26}O_4NS$ $[M+H]^+$ found 400.1571, requires 400.1577 (−1.51 ppm).

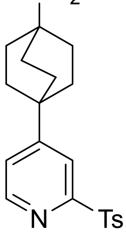

The alternative regioisomer **S34** was also isolated (32.1 mg, 40%) as a white solid.

^{1}H NMR (500 MHz, $CDCl_3$): δ 8.53 (dd, *J* = 5.1, 0.7 Hz, 1H), 8.12 (dd, *J* = 1.9, 0.7 Hz, 1H), 7.93 (d, *J* = 8.3 Hz, 2H), 7.47–7.28 (m, 3H), 3.67 (s, 3H), 2.40 (s, 3H), 1.99–1.89 (m, 6H), 1.89–1.81 (m, 6H);

^{13}C{^{1}H} NMR (126 MHz, $CDCl_3$): δ 177.8, 161.2, 159.2, 150.5, 144.8, 136.1, 129.9, 129.1, 124.1, 119.3, 52.0, 39.1, 35.7, 31.2, 28.4, 21.8;

HRMS (APCI) $C_{22}H_{26}O_4NS$ $[M+H]^+$ found 400.1571, requires 400.1577 (−1.51 ppm).

Methyl 3-(6-tosylpyridin-3-yl)bicyclo[1.1.1]pentane-1-carboxylate (29)

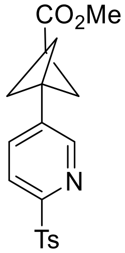

Following **General Procedure 2**, carbocycle **S23** (99.1 mg, 0.2 mmol) and tosyl cyanide (108.8 mg, 0.6 mmol) gave the crude product which was purified by silica column chromatography (*n*-hexane/Et_2O, 1:1 to 1:4) to yield the product (13.9 mg, 19%) as a white solid.

^{1}H NMR (500 MHz, $CDCl_3$): δ 8.50 (d, *J* = 2.1 Hz, 1H), 8.12 (d, *J* = 8.0 Hz, 1H), 7.92 (d, *J* = 8.1 Hz, 2H), 7.70 (dd, *J* = 8.0, 2.1 Hz, 1H), 7.32 (d, *J* = 8.1 Hz, 2H), 3.71 (s, 3H), 2.43–3.30 (m, 3H), 2.41–2.33 (m, 6H);

^{13}C{^{1}H} NMR (126 MHz, $CDCl_3$): δ 169.7, 157.6, 148.7, 144.8, 136.0, 135.7, 129.8, 128.9, 121.7, 53.5, 51.9, 39.5, 37.6, 21.7;

HRMS (APCI) $C_{19}H_{20}NO_4S$ $[M+H]^+$ found 358.1119, requires 358.1108 (+3.20 ppm).

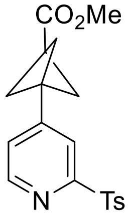

The alternative regioisomer **S35** was also isolated (38.8 mg, 54%) as a white solid.

^{1}H NMR (500 MHz, $CDCl_3$): δ 8.56 (dd, *J* = 4.9, 0.8 Hz, 1H), 8.00 (dd, *J* = 1.6, 0.8 Hz, 1H), 7.94 (d, *J* = 8.0 Hz, 2H), 7.33 (d, *J* = 8.0 Hz, 2H), 7.24 (dd, *J* = 4.9, 1.6 Hz, 1H), 3.73 (s, 3H), 2.41 (s, 3H), 2.39 (s, 6H);

^{13}C{^{1}H} NMR (126 MHz, $CDCl_3$): δ 169.8, 159.4, 151.0, 150.5, 145.0, 135.9, 129.9, 129.2, 124.5, 119.7, 53.5, 52.1, 40.9, 37.5, 21.8;

HRMS (APCI) $C_{19}H_{20}NO_4S$ $[M+H]^+$ found 358.1115, requires 358.1108 (+2.08 ppm).

2-Tosyl-5-(trifluoromethyl)pyridine (30)

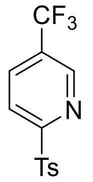

Following **General Procedure 2**, carbocycle **S24** (87.9 mg, 0.2 mmol) and tosyl cyanide (108.8 mg, 0.6 mmol) gave the crude product which was purified by silica column chromatography (*n*-hexane/EtOAc, 100:0 to 4:1) to yield the product (15.3 mg, 25%) as a white solid.

^{1}H NMR (500 MHz, $CDCl_3$): δ 8.93–8.87 (m, 1H), 8.33 (d, J = 8.2 Hz, 1H), 8.17 (ddd, J = 8.2, 2.3, 0.8 Hz, 1H), 7.95 (d, J = 8.1 Hz, 1H), 7.36 (d, J = 8.1 Hz, 2H), 2.43 (s, 4H);

^{13}C{^{1}H} NMR (126 MHz, $CDCl_3$): δ 162.3, 147.4 (q, J = 3.9 Hz), 145.6, 135.7 (q, J = 3.6 Hz), 134.8, 130.0, 129.3, 129.3 (q, J = 33.8 Hz), 122.5 (q, J = 273.1 Hz), 121.7, 21.7;

^{19}F{^{1}H} NMR (377 MHz, $CDCl_3$): δ −62.8;

HRMS (APCI) $C_{13}H_{11}F_3NO_2S$ $[M+H]^+$ found 302.0459, requires 302.0457 (+0.63 ppm).

Ethyl 2,2-difluoro-2-(6-tosylpyridin-3-yl)acetate (31)

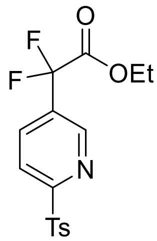

Following **General Procedure 2**, carbocycle **S25** (98.7 mg, 0.2 mmol) and tosyl cyanide (108.8 mg, 0.6 mmol) gave the crude product which was purified by silica column chromatography (*n*-hexane/EtOAc, 100:0 to 4:1) to yield the product (47.2 mg, 66%) as a colourless oil.

^{1}H NMR (500 MHz, $CDCl_3$): δ 8.87 (dd, J = 2.2, 0.9 Hz, 1H), 8.28 (dd, J = 8.2, 0.9 Hz, 1H), 8.15 (dd, J = 8.2, 2.2 Hz, 1H), 7.95 (d, J = 8.1 Hz, 2H), 7.36 (d, J = 8.1 Hz, 2H), 4.31 (q, J = 7.1 Hz, 2H), 2.43 (s, 3H), 1.31 (t, J = 7.1 Hz, 3H);

^{13}C{^{1}H} NMR (126 MHz, $CDCl_3$): δ 162.7 (t, J = 33.9 Hz), 161.5 (t, J = 2.0 Hz), 148.0 (t, J = 6.2 Hz), 145.6, 136.1 (t, J = 5.9 Hz), 135.1, 132.0 (t, J = 26.2 Hz), 130.1, 129.4, 121.7, 111.9 (t, J = 254.1 Hz), 64.2, 21.9, 14.0;

^{19}F{^{1}H} NMR (377 MHz, $CDCl_3$): δ −104.6;

HRMS (APCI) $C_{16}H_{16}F_2NO_4S$ $[M+H]^+$ found 356.0766, requires 356.0763 (+0.64 ppm).

5-Phenyl-2-tosylpyridine (32)

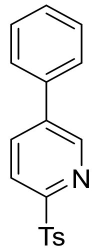

Following **General Procedure 2**, carbocycle **4** (89.5 mg, 0.2 mmol) and tosyl cyanide (108.8 mg, 0.6 mmol) gave the crude product which was purified by silica column chromatography (*n*-hexane/EtOAc, 100:0 to 1:1) to yield the product (8.9 mg, 14%) as a white solid.

^{1}H NMR (500 MHz, $CDCl_3$): δ 8.87 (dd, *J* = 2.3, 0.8 Hz, 1H), 8.24 (dd, *J* = 8.2, 0.8 Hz, 1H), 8.06 (dd, *J* = 8.1, 2.3 Hz, 1H), 7.98 (d, *J* = 8.4 Hz, 2H), 7.59–7.51 (m, 2H), 7.54–7.41 (m, 3H), 7.35 (d, *J* = 7.9 Hz, 1H);

^{13}C{^{1}H} NMR (126 MHz, $CDCl_3$): δ 157.6, 149.1, 145.0, 140.0, 136.2, 136.2, 130.0, 129.5, 129.4, 129.1, 127.6, 122.3, 21.8;

HRMS (APCI) $C_{18}H_{16}O_2NS$ $[M+H]^+$ found 310.0898, requires 310.0896 (+0.56 ppm).

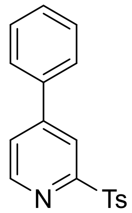

The alternative regioisomer **S36** was also isolated (42.9 mg, 69%) as a colourless oil.

^{1}H NMR (500 MHz, $CDCl_3$): δ 8.68 (d, *J* = 5.0 Hz, 1H), 8.41 (d, *J* = 1.6 Hz, 1H), 7.98 (d, *J* = 8.2 Hz, 2H), 7.79–7.63 (m, 1H), 7.63 (dd, *J* = 5.0, 1.6 Hz, 1H), 7.56–7.42 (m, 3H), 7.33 (d, *J* = 8.2 Hz, 2H), 2.41 (s, 3H);

^{13}C{^{1}H} NMR (126 MHz, $CDCl_3$): δ 159.8, 151.0, 145.0, 136.6, 136.0, 130.2, 129.9, 129.5, 129.1, 127.2, 124.4, 119.9, 21.8;

HRMS (APCI) $C_{18}H_{16}O_2NS$ $[M+H]^+$ found 310.0894, requires 310.0896 (−0.73 ppm).

4-(6-Tosylpyridin-3-yl)benzonitrile (33)

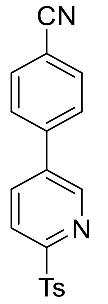

Following **General Procedure 2**, carbocycle **S14** (94.5 mg, 0.2 mmol) and tosyl cyanide (108.8 mg, 0.6 mmol) gave the crude product which was purified by silica column chromatography (*n*-hexane/EtOAc, 100:0 to 1:1) to yield the product (10.9 mg, 16%) as a white solid.

^{1}H NMR (500 MHz, $CDCl_3$): δ 8.85 (dd, *J* = 2.3, 0.8 Hz, 1H), 8.29 (d, *J* = 8.2 Hz, 1H), 8.08 (dd, *J* = 8.2, 2.3 Hz, 1H), 7.97 (d, *J* = 8.1 Hz, 2H), 7.79 (d, *J* = 8.5 Hz, 2H), 7.66 (d, *J* = 8.5 Hz, 2H), 7.36 (d, *J* = 8.1 Hz, 2H), 2.43 (s, 3H);

^{13}C{^{1}H} NMR (126 MHz, $CDCl_3$): δ 159.0, 149.0, 145.3, 140.6, 138.0, 136.6, 135.8, 133.3, 130.1, 129.2, 128.2, 122.4, 118.3, 113.3, 21.8;

HRMS (APCI) $C_{18}H_{16}O_2NS$ $[M+H]^+$ found 335.0851, requires 335.0849 (+0.67 ppm).

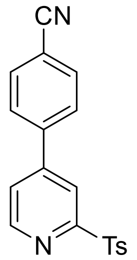

The alternative regioisomer **S37** was also isolated (30.7 mg, 46%) as a white solid.

^{1}H NMR (500 MHz, $CDCl_3$): δ 8.75 (d, *J* = 5.0 Hz, 1H), 8.40 (d, *J* = 1.8 Hz, 1H), 7.97 (d, *J* = 8.2 Hz, 2H), 7.83 (d, *J* = 8.5 Hz, 2H), 7.78 (d, *J* = 8.5 Hz, 2H), 7.63 (dd, *J* = 5.0, 1.8 Hz, 1H), 7.35 (d, *J* = 8.2 Hz, 2H), 2.42 (s, 3H);

^{13}C{^{1}H} NMR (126 MHz, $CDCl_3$): δ 160.3, 151.4, 149.0, 145.3, 141.0, 135.6, 133.3, 130.0, 129.2, 128.1, 124.5, 119.9, 118.2, 113.9, 21.8;

HRMS (APCI) $C_{18}H_{16}O_2NS$ $[M+H]^+$ found 335.0850, requires 335.0849 (+0.37 ppm).

4-Bromo-5-phenyl-2-tosylpyridine (34)

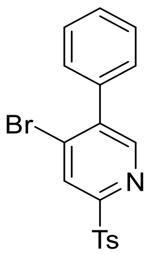

Following **General Procedure 2**, carbocycle **S15** (105.3 mg, 0.2 mmol) and tosyl cyanide (108.8 mg, 0.6 mmol) gave the crude product which was purified by silica column chromatography (*n*-hexane/EtOAc, 100:0 to 3:1) to yield the product (23.9 mg, 31%) as a colourless oil. Note: Two columns were required to enable complete separation of the regioisomers. To clearly distinguish between the two regioisomers by TLC the plate was run twice, first in 80:20 and then in 70:30 *n*-hexane/Et_2O.

^{1}H NMR (500 MHz, $CDCl_3$): δ 8.82 (s, 1H), 8.14 (s, 1H), 7.96 (d, J = 8.1 Hz, 2H), 7.64–7.40 (m, 5H), 7.35 (d, J = 8.1 Hz, 2H), 2.43 (s, 3H);

^{13}C{^{1}H} NMR (126 MHz, $CDCl_3$): δ 158.1, 153.3, 152.0, 145.3, 137.0, 135.7, 130.1, 129.8, 129.2, 128.9, 128.8, 124.6, 124.0, 21.9;

HRMS (APCI) $C_{18}H_{15}NO_2BrS$ $[M+H]^+$ found 387.9993, requires 388.0001 (−2.16 ppm).

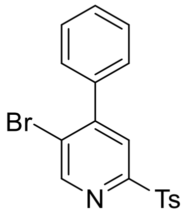

The alternative regioisomer **S38** was also isolated (37.1 mg, 48%) as a colourless oil.

^{1}H NMR (500 MHz, $CDCl_3$): δ 8.52 (s, 1H), 8.46 (s, 1H), 7.98 (d, J = 8.0 Hz, 2H), 7.53–7.41 (m, 3H), 7.41–7.32 (m, 4H), 2.44 (s, 3H);

^{13}C{^{1}H} NMR (126 MHz, $CDCl_3$): δ 158.1, 151.5, 145.5, 141.9, 135.8, 135.6, 134.8, 130.1, 129.4, 129.3, 129.2, 128.8, 126.6, 21.9;

HRMS (APCI) $C_{18}H_{15}NO_2BrS$ $[M+H]^+$ found 387.9993, requires 388.0001 (−2.16 ppm).

4-Chloro-5-phenyl-2-tosylpyridine (35)

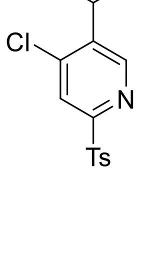

Following **General Procedure 2**, carbocycle **S26** (96.4 mg, 0.2 mmol) and tosyl cyanide (108.8 mg, 0.6 mmol) gave the crude product which was purified by silica column chromatography (*n*-hexane/EtOAc, 100:0 to 3:1) to yield the product (22.2 mg, 32%) as a colourless oil.

^{1}H NMR (500 MHz, $CDCl_3$): δ 8.67 (s, 1H), 8.17 (s, 1H), 7.96 (d, *J* = 8.3, 2H), 7.68–7.43 (m, 5H), 7.35 (d, *J* = 8.0, 2H), 2.43 (s, 3H);

$^{13}C\{^1H\}$ NMR (126 MHz, $CDCl_3$): δ 157.4, 150.9, 149.7, 145.3, 135.8, 135.3, 133.7, 130.1, 129.9, 129.2, 129.0, 128.9, 123.9, 21.9;

HRMS (APCI) $C_{18}H_{15}NO_2ClS$ $[M+H]^+$ found 344.0510, requires 344.0507 (+1.01 ppm).

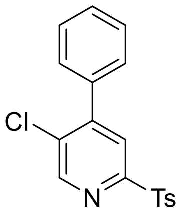

The alternative regioisomer **S39** was also isolated (32.9 mg, 48%) as a colourless oil.

^{1}H NMR (500 MHz, $CDCl_3$): δ 8.57 (s, 1H), 8.29 (s, 1H), 7.99 (d, *J* = 8.3, 2H), 7.61–7.43 (m, 3H), 7.43–7.29 (m, 4H), 2.44 (s, 3H);

$^{13}C\{^1H\}$ NMR (126 MHz, $CDCl_3$): δ 158.6, 152.1, 145.4, 144.4, 139.5, 135.6, 134.1, 130.1, 129.4, 129.3, 128.8, 123.5, 21.9;

HRMS (APCI) $C_{18}H_{15}NO_2ClS$ $[M+H]^+$ found 344.0517, requires 344.0507 (+3.04 ppm).

4-Chloro-5-(thiophen-3-yl)-2-tosylpyridine (36)

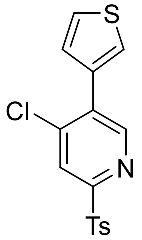

Following **General Procedure 2**, carbocycle **S27** (97.6 mg, 0.2 mmol) and tosyl cyanide (108.8 mg, 0.6 mmol) gave the crude product which was purified by silica column chromatography (*n*-hexane/EtOAc, 100:0 to 3:1) to yield the product (26.2 mg, 37%) as a yellow oil.

^{1}H NMR (500 MHz, $CDCl_3$): δ 8.67 (s, 1H), 8.26 (s, 1H), 7.96 (d, *J* = 8.2, 2H), 7.59 (dd, *J* = 3.0, 1.4, 1H), 7.46 (dd, *J* = 5.1, 3.0, 1H), 7.36 (d, *J* = 8.2, 2H), 7.32 (dd, *J* = 5.1, 1.4, 1H), 2.43 (s, 3H);

$^{13}C\{^1H\}$ NMR (126 MHz, $CDCl_3$): δ 158.0, 151.7, 145.4, 143.8, 135.6, 134.5, 134.1, 130.1, 129.2, 128.1, 126.7, 126.6, 123.8, 21.9;

HRMS (APCI) $C_{16}H_{13}NO_2ClS_2$ $[M+H]^+$ found 350.0058, requires 350.0071 (−3.65 ppm).

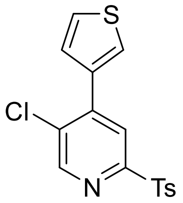

The alternative regioisomer **S40** was also isolated (34.1 mg, 49%) as a yellow oil.

^1H NMR (500 MHz, $CDCl_3$): δ 8.64 (s, 1H), 8.25 (s, 1H), 7.95 (d, *J* = 8.2, 2H), 7.81 (dd, *J* = 3.0, 1.4, 1H), 7.48 (dd, *J* = 5.1, 3.0, 1H), 7.44 (dd, *J* = 5.1, 1.4, 1H), 7.34 (d, *J* = 8.2, 2H), 2.43 (s, 3H);

$^{13}C\{^1H\}$ NMR (126 MHz, $CDCl_3$): δ 157.4, 151.3, 145.3, 144.0, 135.7, 135.3, 132.9, 130.0, 129.2, 127.9, 127.7, 126.7, 123.1, 21.8;

HRMS (APCI) $C_{16}H_{13}NO_2ClS_2$ $[M+H]^+$ found 350.0058, requires 350.0071 (−3.65 ppm).

1,3-Diethyl-3-(6-tosylpyridin-3-yl)piperidine-2,6-dione (38)

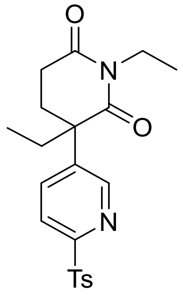

Following **General Procedure 2**, carbocycle **S28** (107.7 mg, 0.2 mmol) and tosyl cyanide (108.8 mg, 0.6 mmol) gave the crude product which was purified by silica column chromatography (*n*-hexane/EtOAc, 100:0 to 1:1) to yield the product (50.7 mg, 63%) as a tan solid.

^1H NMR (500 MHz, $CDCl_3$): δ 8.53 (s, 1H), 8.12 (d, *J* = 8.4 Hz, 1H), 7.90 (d, *J* = 7.8 Hz, 2H), 7.80 (dd, *J* = 8.4, 2.4 Hz, 1H), 7.31 (d, *J* = 7.8 Hz, 2H), 3.93–3.68 (m, 2H), 2.76–2.55 (m, 1H), 2.38–2.17 (m, 3H), 2.38 (s, 3H), 2.06 (dq, *J* = 14.8, 7.4 Hz, 1H), 1.86 (dq, *J* = 14.5, 7.3 Hz, 1H), 1.07 (t, *J* = 7.0 Hz, 3H), 0.81 (t, *J* = 7.4 Hz, 3H);

$^{13}C\{^1H\}$ NMR (126 MHz, $CDCl_3$): δ 173.2, 170.8, 158.1, 148.7, 145.2, 139.6, 136.5, 135.5, 130.0, 129.1, 122.0, 50.1, 35.7, 33.4, 29.4, 25.4, 21.7, 13.3, 8.9;

HRMS (APCI) $C_{21}H_{25}O_4N_2S$ $[M+H]^+$ found 401.1526, requires 401.1530 (−0.88 ppm).

11 Derivatisations of 2-Tosyl Pyridines

5-(Tert-butyl)-2-phenylpyridine (40)

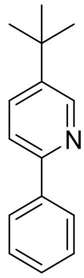

To a microwave vial equipped with a stir bar was added 5-(tert-butyl)-2-tosylpyridine (28.9 mg, 0.1 mmol). The vial was capped and evacuated and refilled with N_2 three times on a Schlenk line. THF (0.5 M, 0.5 mL) was added and the solution was cooled to 0 °C in an ice bath. Phenylmagnesium bromide (3 M solution in Et_2O, 0.1 mL, 3 equiv.) was added dropwise. The solution was allowed to warm to room temperature and stirred overnight. Water was added and the aqueous layer was extracted three times with CH_2Cl_2. The combined organic extracts were dried ($MgSO_4$), filtered, and concentrated in vacuo to give the crude product, which was purified by silica column chromatography (*n*-hexane/EtOAc, 100:0 to 4:1) to yield the product (17.2 mg, 81%) as a colourless oil.

^{1}H NMR (500 MHz, $CDCl_3$): δ 8.75 (d, *J* = 2.5 Hz, 1H), 7.99 (d, *J* = 7.2 Hz, 2H), 7.77 (dd, *J* = 8.3, 2.5 Hz, 1H), 7.67 (d, *J* = 8.3 Hz, 1H), 7.47 (t, *J* = 7.5 Hz, 2H), 7.43–7.35 (m, 1H), 1.39 (s, 9H);

^{13}C{^{1}H} NMR (126 MHz, $CDCl_3$): δ 154.5, 147.0, 144.8, 139.1, 134.3, 128.9, 128.9, 126.9, 120.1, 33.7, 31.1;

HRMS (APCI) $C_{15}H_{18}N$ $[M+H]^+$ found 212.1435, requires 212.1434 (+0.58 ppm).

5-(Tert-butyl)-2-(2-ethoxyethoxy)pyridine (41)

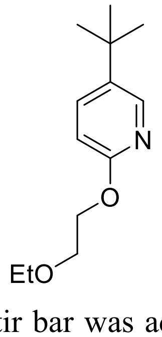

To a microwave vial equipped with a stir bar was added 5-(tert-butyl)-2-tosylpyridine (28.9 mg, 0.1 mmol). The vial was capped and evacuated and refilled with N_2 three times on a Schlenk line. THF (0.5 M, 0.5 mL) was added and the solution was cooled to 0 °C in an ice bath. 2-Ethoxyethan-1-ol (19.5 µL, 0.2 mmol) was added, followed by dropwise addition of

KHMDS (1 M solution in THF, 0.2 mL, 0.2 mmol). The solution was allowed to warm to room temperature and stirred for 1 h. Water was added and the aqueous layer was extracted three times with CH_2Cl_2. The combined organic extracts were dried ($MgSO_4$), filtered, and concentrated in vacuo to give the crude product, which was purified by silica column chromatography (*n*-hexane/EtOAc, 100:0 to 4:1) to yield the product (21.9 mg, 98%) as a colourless oil.

^{1}H NMR (500 MHz, $CDCl_3$): δ 8.13 (d, *J* = 2.7 Hz, 1H), 7.59 (dd, *J* = 8.7, 2.7 Hz, 1H), 6.74 (d, *J* = 8.7 Hz, 1H), 4.53–4.28 (m, 2H), 4.01–3.68 (m, 2H), 3.59 (q, *J* = 7.0 Hz, 2H), 1.30 (s, 9H), 1.26–1.20 (m, 3H);

^{13}C{^{1}H} NMR (126 MHz, $CDCl_3$): δ 161.9, 143.2, 139.0, 136.6, 110.7, 69.2, 66.8, 65.1, 33.1, 31.3, 15.3;

HRMS (APCI) $C_{13}H_{21}NO_2$ $[M+H]^+$ found 224.1646, requires 224.1645 (+0.45 ppm).

5-(Tert-butyl)-2-(pyrrolidin-1-yl)pyridine (42)

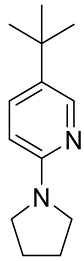

A microwave vial equipped with a stir bar was and evacuated and refilled with N_2 three times on a Schlenk line. Pyrrolidine (16.4 μL, 0.2 mmol) was added, followed by THF (0.5 M, 0.5 mL) The solution was cooled to 0 °C in an ice bath and ^{n}BuLi (2.5 M in hexane, 80 μL, 0.2 mmol) was added dropwise. The solution was allowed to warm to room temperature and stirred for 30 mins. To this solution was added a solution of 5-(tert-butyl)-2-tosylpyridine (28.9 mg, 0.1 mmol) in THF (0.5 mL). The solution was stirred for 15 mins before quenching with water. The aqueous layer was extracted three times with CH_2Cl_2 and the combined organic extracts were dried ($MgSO_4$), filtered, and concentrated in vacuo to give the crude product, which was purified by silica column chromatography (*n*-hexane/Et_2O, 1:1 to 1:4) to yield the product (13.3 mg, 65%) as a colourless oil.

^{1}H NMR (500 MHz, $CDCl_3$): δ 8.18 (d, *J* = 2.6 Hz, 1H), 7.50 (dd, *J* = 8.8, 2.6 Hz, 1H), 6.35 (d, *J* = 8.8 Hz, 1H), 3.46 (t, *J* = 6.3 Hz, 4H), 2.41–1.85 (m, 4H), 1.28 (s, 9H);

^{13}C{^{1}H} NMR (126 MHz, $CDCl_3$): δ 155.2, 144.0, 135.4, 133.2, 106.5, 47.0, 32.8, 31.3, 25.7;

HRMS (APCI) $C_{13}H_{21}N_2$ $[M+H]^+$ found 205.1702, requires 205.1699 (+1.34 ppm).

5-(Tert-butyl)-2-(methylthio)pyridine (43)

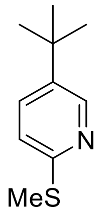

To a microwave vial equipped with a stir bar was added 5-(tert-butyl)-2-tosylpyridine (28.9 mg, 0.1 mmol) and NaSMe (56.1 mg, 0.8 mmol). The vial was capped and evacuated and refilled with N_2 three times on a Schlenk line. THF (0.1 M, 1 mL) was added and the solution was heated at 90°C overnight with stirring. The solution was allowed to cool to room temperature and water was added. The aqueous layer was extracted three times with CH_2Cl_2 and the combined organic extracts were dried ($MgSO_4$), filtered, and concentrated in vacuo to give the crude product, which was purified by silica column chromatography (*n*-hexane/EtOAc, 100:0 to 4:1) to yield the product (14.2 mg, 78%) as a colourless oil.

^{1}H NMR (500 MHz, $CDCl_3$): δ 8.65 (d, *J* = 2.5 Hz, 1H), 7.67 (dd, *J* = 8.4, 2.5 Hz, 1H), 7.27 (d, *J* = 8.4 Hz, 1H), 2.71 (s, 3H), 1.47 (s, 9H);

^{13}C{^{1}H} NMR (126 MHz, $CDCl_3$): δ 156.6, 146.9, 141.7, 133.6, 121.0, 33.3, 31.1, 13.5;

HRMS (APCI) $C_{10}H_{16}NS$ $[M+H]^+$ found 182.1001, requires 182.0998 (+1.65 ppm).

5-(Tert-butyl)-2-(p-tolyl)pyridine (44)

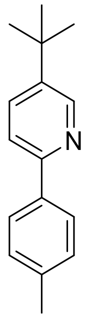

Following **General Procedure 3**, 5-(tert-butyl)-2-tosylpyridine (28.9 mg, 0.1 mmol), p-tolylboronic acid (27.2 mg, 0.2 mmol), $NiBr_2$.glyme (3.1 mg, 0.01 mmol), ProPhos (9.8 mg, 0.04 mmol), and K_3PO_4 (53.1 mg, 0.25 mmol) gave the crude product which was purified by silica column chromatography (*n*-hexane/EtOAc, 100:0 to 4:1) to yield the product (20.3 mg, 90%) as a colourless oil.

^{1}H NMR (500 MHz, $CDCl_3$): δ 8.73 (d, *J* = 2.5 Hz, 1H), 7.89 (d, *J* = 8.0 Hz, 2H), 7.75 (dd, *J* = 8.4, 2.5 Hz, 1H), 7.65 (d, *J* = 8.4 Hz, 1H), 7.28 (d, *J* = 8.0 Hz, 2H), 2.40 (s, 3H), 1.38 (s, 9H);

^{13}C{^{1}H} NMR (126 MHz, $CDCl_3$): δ 154.5, 146.8, 144.4, 138.9, 134.4, 129.6, 126.8, 119.9, 33.6, 31.1, 21.4;

HRMS (APCI) $C_{16}H_{20}N$ $[M+H]^+$ found 226.1588, requires 226.1590 (−1.00 ppm).

5-(Tert-butyl)-2-(4-methoxyphenyl)pyridine (45)

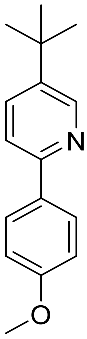

Following **General Procedure 3**, 5-(tert-butyl)-2-tosylpyridine (28.9 mg, 0.1 mmol), 4-methoxyphenyl boronic acid (30.4 mg, 0.2 mmol), $NiBr_2$.glyme (3.1 mg, 0.01 mmol), ProPhos (9.8 mg, 0.04 mmol), and K_3PO_4 (53.1 mg, 0.25 mmol) gave the crude product which was purified by silica column chromatography (*n*-hexane/EtOAc, 100:0 to 1:1) to yield the product (22.7 mg, 94%) as a white solid.

^{1}H NMR (500 MHz, $CDCl_3$): δ 8.70 (d, J = 2.5 Hz, 1H), 7.94 (d, J = 8.8 Hz, 2H), 7.73 (dd, J = 8.4, 2.5 Hz, 1H), 7.61 (d, J = 8.4 Hz, 1H), 6.99 (d, J = 8.8 Hz, 2H), 3.86 (s, 3H), 1.38 (s, 9H);
^{13}C{^{1}H} NMR (126 MHz, $CDCl_3$): δ 160.4, 154.3, 146.9, 143.9, 134.2, 131.8, 128.1, 119.4, 114.3, 55.5, 33.6, 31.2;
HRMS (APCI) $C_{16}H_{20}ON$ $[M+H]^+$ found 242.1533, requires 242.1539 (−2.65 ppm).

5-(Tert-butyl)-2-(4-(trifluoromethyl)phenyl)pyridine (46)

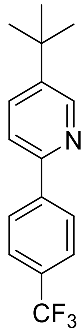

Following **General Procedure 3**, 5-(tert-butyl)-2-tosylpyridine (28.9 mg, 0.1 mmol), 4-(trifluoromethyl)phenyl boronic acid (38.0 mg, 0.2 mmol), $NiBr_2$.glyme (3.1 mg, 0.01 mmol), ProPhos (9.8 mg, 0.04 mmol), and K_3PO_4 (53.1 mg, 0.25 mmol) gave the crude product which was purified by silica column chromatography (*n*-hexane/EtOAc, 100:0 to 1:1) to yield the product (19.6 mg, 70%) as a white solid.

^{1}H NMR (500 MHz, $CDCl_3$): δ 8.78 (dd, *J* = 2.5, 0.9 Hz, 1H), 8.10 (d, *J* = 7.9 Hz, 2H), 7.80 (dd, *J* = 8.3, 2.5 Hz, 1H), 7.76–7.47 (m, 3H), 1.40 (s, 9H);

^{13}C{^{1}H} NMR (126 MHz, $CDCl_3$): δ 152.6, 146.8, 146.2, 135.2, 131.0 (q, *J* = 32.6 Hz), 127.8, 127.3, 125.9 (q, *J* = 3.8 Hz), 124.3 (q, *J* = 272.2 Hz), 120.8, 33.8, 31.1;

^{19}F{^{1}H} NMR (377 MHz, $CDCl_3$): δ −62.6;

HRMS (APCI) $C_{16}H_{17}NF_3$ $[M+H]^+$ found 280.1308, requires 280.1308 (+0.14 ppm).

Methyl 4-(6-(p-tolyl)pyridin-3-yl)bicyclo[2.2.2]octane-1-carboxylate (47)

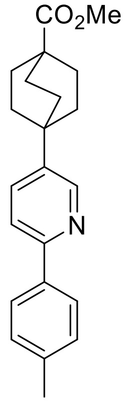

Following **General Procedure 3**, methyl 4-(6-tosylpyridin-3-yl)bicyclo[2.2.2]octane-1-carboxylate (40.0 mg, 0.1 mmol), p-tolylboronic acid (27.2 mg, 0.2 mmol), $NiBr_2$.glyme (3.1 mg, 0.01 mmol), ProPhos (9.8 mg, 0.04 mmol), and K_3PO_4 (53.1 mg, 0.25 mmol) gave the crude product which was purified by silica column chromatography (*n*-hexane/EtOAc, 100:0 to 1:1) to yield the product (31.9 mg, 95%) as a white solid.

^{1}H NMR (500 MHz, $CDCl_3$): δ 8.64 (d, *J* = 2.3 Hz, 1H), 7.87 (d, *J* = 7.8 Hz, 2H), 7.72–7.55 (m, 2H), 7.27 (d, *J* = 7.8 Hz, 2H), 3.68 (s, 3H), 2.40 (s, 3H), 2.18–1.59 (m, 12H);

^{13}C{^{1}H} NMR (126 MHz, $CDCl_3$): δ 178.3, 154.9, 147.2, 142.5, 138.9, 136.3, 134.3, 129.6, 126.7, 119.8, 51.9, 39.1, 33.6, 31.6, 28.7, 21.4;

HRMS (APCI) $C_{22}H_{26}O_2N$ $[M+H]^+$ found 336.1967, requires 336.1958 (+2.66 ppm).

1,3-Diethyl-3-(6-(p-tolyl)pyridin-3-yl)piperidine-2,6-dione (48)

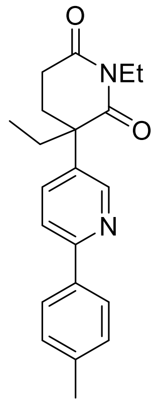

Following **General Procedure 3**, 1,3-diethyl-3-(6-tosylpyridin-3-yl)piperidine-2,6-dione (40.0 mg, 0.1 mmol), p-tolylboronic acid (27.2 mg, 0.2 mmol), $NiBr_2$.glyme (3.1 mg, 0.01 mmol), ProPhos (9.8 mg, 0.04 mmol), and K_3PO_4 (53.1 mg, 0.25 mmol) gave the crude product which was purified by silica column chromatography (*n*-hexane/EtOAc, 100:0 to 1:1) to yield the product (19.5 mg, 58%) as a white solid.

^{1}H NMR (500 MHz, $CDCl_3$): δ 8.56 (d, *J* = 2.6 Hz, 1H), 7.88 (d, *J* = 8.2 Hz, 2H), 7.70 (d, *J* = 8.4 Hz, 1H), 7.61 (d, *J* = 8.2 Hz, 2H), 3.90 (tdd, *J* = 12.5, 7.0, 5.6 Hz, 2H), 2.72 (ddd, *J* = 18.0, 4.7, 3.0 Hz, 1H), 2.49 (ddd, *J* = 18.0, 13.4, 5.1 Hz, 1H), 2.45–2.32 (m, 4H), 2.24 (td, *J* = 13.8, 4.7 Hz, 1H), 2.13 (dq, *J* = 14.6, 7.4 Hz, 1H), 1.97 (dq, *J* = 14.6, 7.3 Hz, 1H), 1.16 (t, *J* = 7.0 Hz, 3H), 0.91 (t, *J* = 7.4 Hz, 3H);

^{13}C{^{1}H} NMR (126 MHz, $CDCl_3$): δ 173.9, 171.2, 155.5, 145.7, 140.7, 137.7, 134.5, 130.0, 127.3, 121.2, 49.8, 35.7, 33.4, 29.7, 25.5, 21.5, 13.4, 9.0;

HRMS (APCI) $C_{21}H_{25}O_2N_2$ $[M+H]^+$ found 337.1916, requires 337.1911 (+1.62 ppm).

3-(Tert-butyl)pyridine (49)

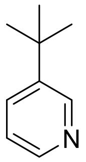

To a microwave vial equipped with a stir bar was added 5-(tert-butyl)-2-tosylpyridine (28.9 mg, 0.1 mmol) and $NaH_2PO_4.H_2O$ (55.2 mg, 0.4 mmol) and 20 % Na(Hg) (59.0 mg, 0.5 mmol). The vial was capped and evacuated and refilled with N_2 three times on a Schlenk line. MeOH (0.1 M, 1 mL) was added and the resulting mixture was stirred overnight. Water was added and the aqueous layer was extracted three times with CH_2Cl_2. The combined organic extracts were dried ($MgSO_4$), filtered, and concentrated in vacuo to give the crude product, which was purified by silica column chromatography (*n*-hexane/EtOAc, 100:0 to 4:1) to yield the product (9.5 mg, 70%) as a colourless oil.

^{1}H NMR (500 MHz, $CDCl_3$): δ 8.68 (br s, 1H), 8.43 (d, J = 4.6 Hz, 1H), 7.70 (dt, J = 8.0, 2.0 Hz, 1H), 7.23 (dd, J = 8.0, 4.6 Hz, 1H), 1.36 (s, 9H);
^{13}C{^{1}H} NMR (126 MHz, $CDCl_3$): δ 147.5, 146.9, 145.9, 132.8, 122.9, 33.6, 31.0.

12 Pyridine to Pyridazine Conversion

Diethyl 1,3-dioxo-2,6-diphenyl-11-((1,1,1-trifluoro-N-methylmethyl)sulfonamido)-2,3,5,8-tetrahydro-1H-5,8-ethano[1,2,4]triazolo[1,2-a]pyridazine-10,10-dicarboxylate (S2)

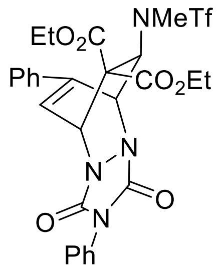

Following **General Procedure 4**, carbocycle **4** (89.5 mg, 0.2 mmol), K_2CO_3 (41.5 mg, 0.3 mmol), MeI (37.5 µL, 0.6 mmol), and PTAD (52.5 mg, 0.3 mmol) gave the crude product which was purified by silica column chromatography (*n*-hexane/EtOAc, 100:0 to 1:1) to yield the product (116.4 mg, 91%) as a white solid as a single diastereomer. Note: The configuration of the product diastereomer has not been determined.
^{1}H NMR (500 MHz, $CDCl_3$): δ 7.61–7.52 (m, 2H), 7.50–7.35 (m, 5H), 7.35–7.29 (m, 3H), 6.99 (d, J = 6.3 Hz, 1H), 6.13 (d, J = 2.9 Hz, 1H), 5.68 (dd, J = 6.3, 1.2 Hz, 1H), 5.42 (br s, 1H), 4.51–4.29 (m, 4H), 4.25–4.08 (m, 1H), 2.51 (s, 3H), 1.33 (dtd, J = 11.2, 7.1, 1.2 Hz, 6H);
^{13}C{^{1}H} NMR (126 MHz, $CDCl_3$): δ 166.7, 166.3, 156.1, 155.6, 133.3, 130.9, 129.9, 129.8, 129.2, 128.7, 125.3, 125.3, 122.9, 63.7, 62.8, 58.4, 55.8, 33.2, 14.1, 13.7;
^{19}F{^{1}H} NMR (377 MHz, $CDCl_3$): δ −75.4;
HRMS (APCI) $C_{28}H_{28}O_8N_4F_3S$ $[M+H]^+$ found 637.1578, requires 637.1574 (+0.56 ppm).

Diethyl 6-(4-methoxyphenyl)-1,3-dioxo-2-phenyl-11-((1,1,1-trifluoro-N-methylmethyl)sulfonamido)-2,3,5,8-tetrahydro-1H-5,8-ethano[1,2,4]triazolo[1,2-a]pyridazine-10,10-dicarboxylate (S41)

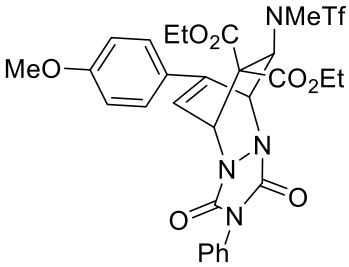

Following **General Procedure 4**, carbocycle **S14** (95.5 mg, 0.2 mmol), K_2CO_3 (41.5 mg, 0.3 mmol), MeI (37.5 µL, 0.6 mmol), and PTAD (52.5 mg, 0.3 mmol) gave the crude product which was purified by silica column chromatography (*n*-hexane/EtOAc, 100:0 to 1:1) to yield the product (131.1 mg, 98%) as a white solid as a single diastereomer. Note: The configuration of the product diastereomer has not been determined.

^{1}H NMR (500 MHz, $CDCl_3$): δ 7.50 (d, *J* = 8.9 Hz, 2H), 7.44–7.35 (m, 2H), 7.35–7.27 (m, 3H), 6.94 (d, *J* = 8.9 Hz, 2H), 6.86 (dd, *J* = 6.3, 2.2 Hz, 1H), 6.11 (d, *J* = 3.0 Hz, 1H), 5.64 (d, *J* = 6.3 Hz, 1H), 5.38 (dd, *J* = 3.0, 2.2 Hz, 1H), 4.56–4.29 (m, 3H), 4.21–4.07 (m, 1H), 3.82 (s, 3H), 2.52 (s, 3H), 1.33 (dt, *J* = 10.5, 7.1 Hz, 6H);

^{13}C{^{1}H} NMR (126 MHz, $CDCl_3$): δ 166.7, 166.4, 161.0, 156.3, 155.7, 138.9, 130.9, 129.2, 128.7, 126.7, 125.7, 125.4, 120.5, 115.2, 63.7, 63.6, 62.8, 58.4, 55.9, 55.5, 33.1, 14.1, 13.8;

^{19}F{^{1}H} NMR (377 MHz, $CDCl_3$): δ −75.5;

HRMS (APCI) $C_{29}H_{30}O_9N_4F_3S$ $[M+H]^+$ found 667.1676, requires 667.1680 (−0.61 ppm).

Diethyl 1,3-dioxo-2-phenyl-6-(thiophen-3-yl)-11-((1,1,1-trifluoro-N-methylmethyl)sulfonamido)-2,3,5,8-tetrahydro-1H-5,8-ethano[1,2,4]triazolo[1,2-a]pyridazine-10,10-dicarboxylate (S42)

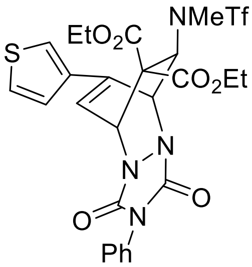

Following **General Procedure 4**, carbocycle **S12** (90.7 mg, 0.2 mmol), K_2CO_3 (41.5 mg, 0.3 mmol), MeI (37.5 µL, 0.6 mmol), and PTAD (52.5 mg, 0.3 mmol) gave the crude product which was purified by silica column chromatography (*n*-hexane/EtOAc, 100:0 to 1:1) to yield the product (103.7 mg, 81%) as a white solid as a single diastereomer. Note: The configuration of the product diastereomer has not been determined.

^{1}**H NMR** (500 MHz, $CDCl_3$): δ 7.54 (dd, *J* = 3.0, 1.4 Hz, 1H), 7.45–7.35 (m, 3H), 7.37–7.29 (m, 3H), 7.29–7.24 (m, 1H), 6.85 (dd, *J* = 6.2, 2.1 Hz, 1H), 6.11 (d, *J* = 3.0 Hz, 1H), 5.63 (d, *J* = 6.2 Hz, 1H), 5.31 (dd, *J* = 3.0, 2.1 Hz, 1H), 4.50–4.25 (m, 3H), 4.22–4.10 (m, 1H), 2.56 (s, 3H), 1.33 (dt, *J* = 10.0, 7.1 Hz, 6H);
^{13}**C{^{1}H} NMR** (126 MHz, $CDCl_3$): δ 166.7, 166.3, 156.3, 155.8, 135.8, 134.5, 130.9, 129.3, 128.7, 128.5, 125.3, 124.5, 123.3, 121.1, 63.7, 62.9, 58.2, 55.8, 32.8, 14.1, 13.8;
^{19}**F{^{1}H} NMR** (377 MHz, $CDCl_3$): δ −75.4;
HRMS (APCI) $C_{22}H_{26}O_8N_4F_3S_2$ $[M+H]^+$ found 643.1139, requires 643.1139 (+0.05 ppm).

4-Phenylpyridazine (54)

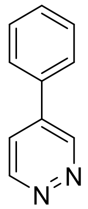

Following **General Procedure 5**, cycloadduct **S2** (63.7 mg, 0.1 mmol) and KOH (1.5 g) gave the crude product which was purified by silica column chromatography (*n*-hexane/EtOAc, 1:1 to 0:100) to yield the product (4.7 mg, 30%) as a pale brown solid.
^{1}**H NMR** (500 MHz, $CDCl_3$): δ 9.54 (s, 1H), 9.32 (d, *J* = 5.3 Hz, 1H), 7.98–7.80 (m, 1H), 7.79–7.65 (m, 2H), 7.66–7.45 (m, 3H);
^{13}**C{^{1}H} NMR** (126 MHz, $CDCl_3$): δ 150.6, 141.0, 133.7, 131.2, 130.0, 127.5, 124.9;
HRMS (APCI) $C_{10}H_9N_2$ $[M+H]^+$ found 157.0763, requires 157.0760 (+1.75 ppm).

4-(4-Methoxyphenyl)pyridazine (55)

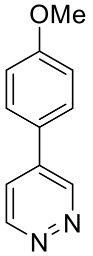

Following **General Procedure 5**, cycloadduct **S41** (66.7 mg, 0.1 mmol) and KOH (1.5 g) gave the crude product which was purified by silica column chromatography (*n*-hexane/EtOAc, 1:1 to 0:100) to yield the product (4.8 mg, 26%) as a pale brown solid.
^{1}**H NMR** (500 MHz, $CDCl_3$): δ 9.44 (dd, *J* = 2.5, 1.1 Hz, 1H), 9.17 (d, *J* = 5.4 Hz, 1H), 7.68–7.52 (m, 3H), 7.05 (d, *J* = 8.8 Hz, 2H), 3.88 (s, 3H);

$^{13}C\{^{1}H\}$ NMR (126 MHz, $CDCl_3$): δ 161.6, 151.3, 149.7, 138.4, 128.5, 126.5, 122.7, 115.2, 55.6;

HRMS (APCI) $C_{11}H_{11}ON_2$ $[M+H]^+$ found 187.0867, requires 187.0866 (+0.59 ppm).

4-(Thiophen-3-yl)pyridazine (56)

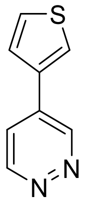

Following **General Procedure 5**, cycloadduct **S42** (64.3 mg, 0.1 mmol) and KOH (1.5 g) gave the crude product which was purified by silica column chromatography (*n*-hexane/EtOAc, 1:1 to 0:100) to yield the product (5.1 mg, 31%) as a pale brown solid.

^{1}H NMR (500 MHz, $CDCl_3$): δ 9.46 (d, *J* = 1.9 Hz, 1H), 9.19 (d, *J* = 5.4 Hz, 1H), 7.83 (dd, *J* = 2.9, 1.4 Hz, 1H), 7.64 (dd, *J* = 5.4, 2.4 Hz, 1H), 7.53 (dd, *J* = 5.1, 2.9 Hz, 1H), 7.49 (dd, *J* = 5.1, 1.4 Hz, 1H);

$^{13}C\{^{1}H\}$ NMR (126 MHz, $CDCl_3$): δ 151.5, 149.4, 135.7, 133.4, 128.2, 125.3, 125.0, 122.3;

HRMS (APCI) $C_8H_7N_2S$ $[M+H]^+$ found 163.0325, requires 163.0324 (+0.33 ppm).

13 References

1. Conboy, A. & Greaney, M. F. Synthesis of benzenes from pyridines via N to C switch. *Chem* **10**, 1940–1949 (2024).

2. J. Choi, G. Laudadio, E. Godineau, P. S. Baran, "Practical and Regioselective Synthesis of C-4-Alkylated Pyridines" *J. Am. Chem. Soc.* **2021**, *143*, 11927–11933.

3. M. Zhou, Y. Hu, K. En, X. Tan, H. C. Shen, X. Qian, "Efficient cyclopropanation of aryl/heteroaryl acetates and acetonitriles with vinyl diphenyl sulfonium triflate" *Tetrahedron Letters* **2018**, *59*, 1443–1445.

4. Q. Wang, B. Zhang, G. Xia, S. Shu, G. Huo, Z. Xiang, G. Wu, T. Liang, C. Shi, Y. Zhao, L. Li, Y. Ke, "RORγt modulator, and preparation method therefor and application", WO 2022/143771 A1, **2022**.

5. L. Gao, G. Wang, J. Cao, H. Chen, Y. Gu, X. Liu, X. Cheng, J. Ma, S. Li, "Lewis Acid-Catalyzed Selective Reductive Decarboxylative Pyridylation of *N* -Hydroxyphthalimide

Esters: Synthesis of Congested Pyridine-Substituted Quaternary Carbons" *ACS Catal.* **2019**, *9*, 10142–10151.

6. H. W. Pauls, R. Laufer, Y. Liu, S.-W. Li, B. T. Forrest, Y. Lang, N. K. B. Patel, L. G. Edwards, G. Ng, P. B. Sampson, M. Feher, "Indazole compounds as kinase inhibitors and method of treating cancer with same", WO 2013/053051 A1, **2013**.

7. O. M. Shavrina, P. P. Onys`ko, Y. V. Rassukana, "Mono- and difluorination of methylene group in isomeric pyrimidinyl- and pyridinylacetates with N-fluorobenzenesulfonimide" *Journal of Fluorine Chemistry* **2022**, *261–262*, 110027.

8. R. McCague, M. Jarman, M. G. Rowlands, J. Mann, C. P. Thickitt, D. W. Clissold, S. Neidle, G. Webster, "Synthesis of the aromatase inhibitor 3-ethyl-3-(4-pyridyl)piperidine-2,6-dione and its enantiomers" *J. Chem. Soc., Perkin Trans. 1* **1989**, 196.

14 NMR Spectra

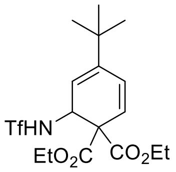

16

^{1}H NMR ($CDCl_3$, 500 MHz)

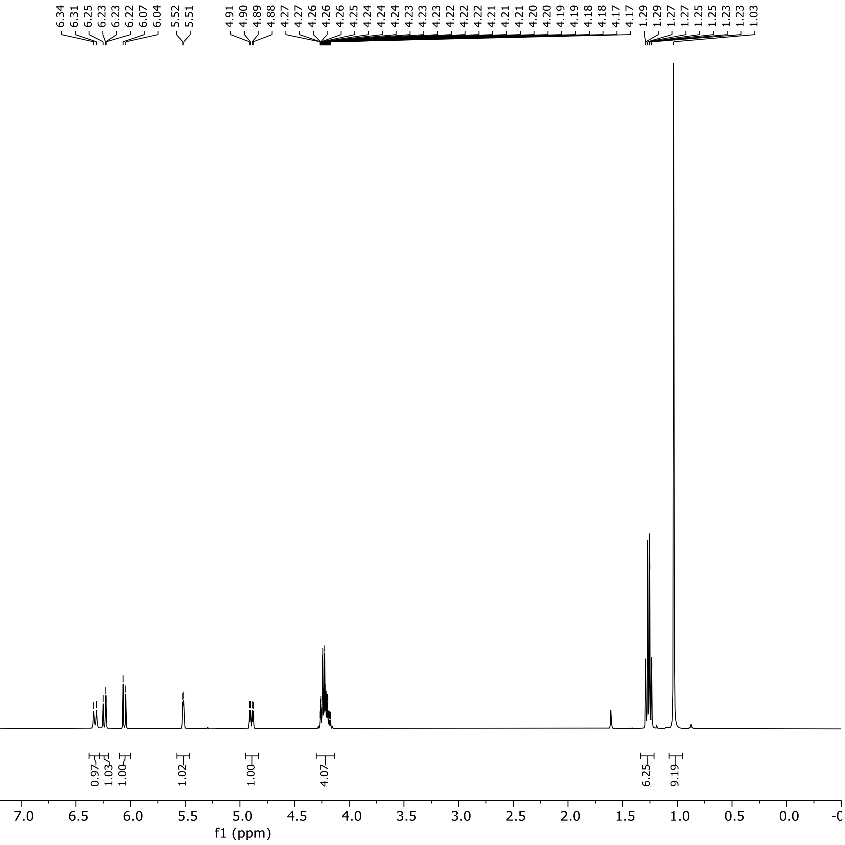

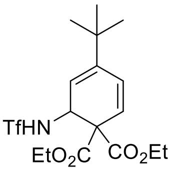

16

$^{13}C\{^1H\}$ NMR ($CDCl_3$, 126 MHz)

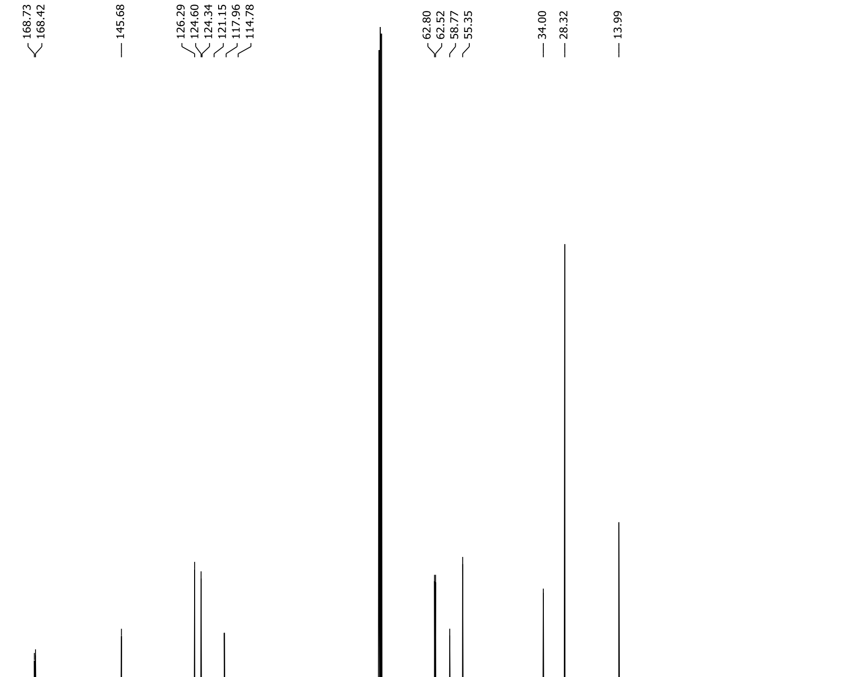

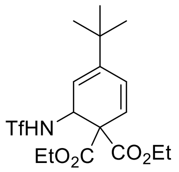

16

$^{19}F\{^{1}H\}$ NMR ($CDCl_3$, 377 MHz)

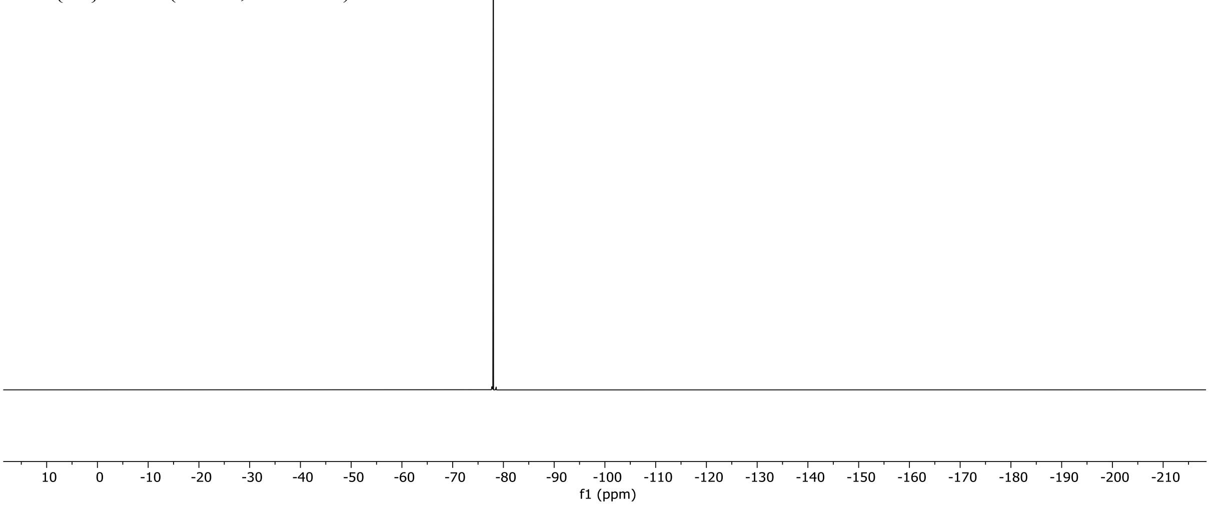

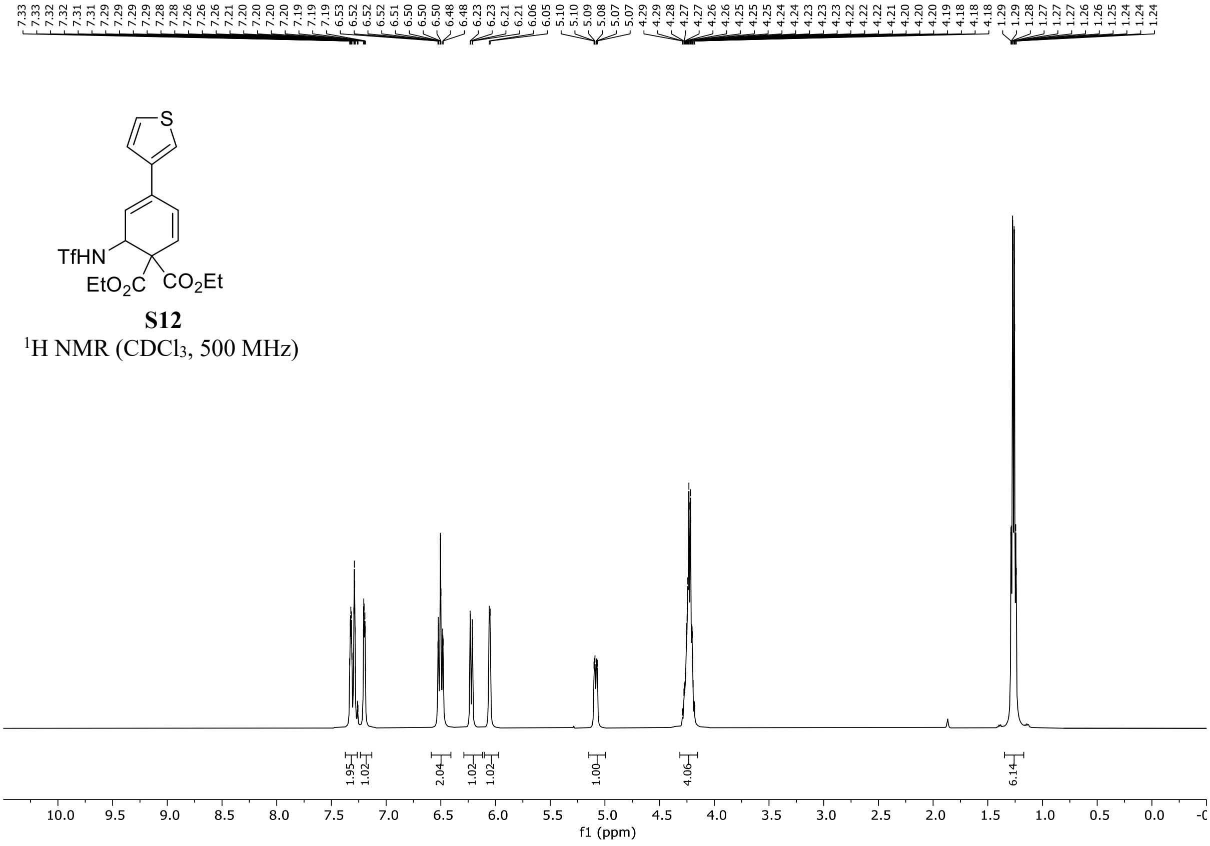

S12

^{1}H NMR ($CDCl_3$, 500 MHz)

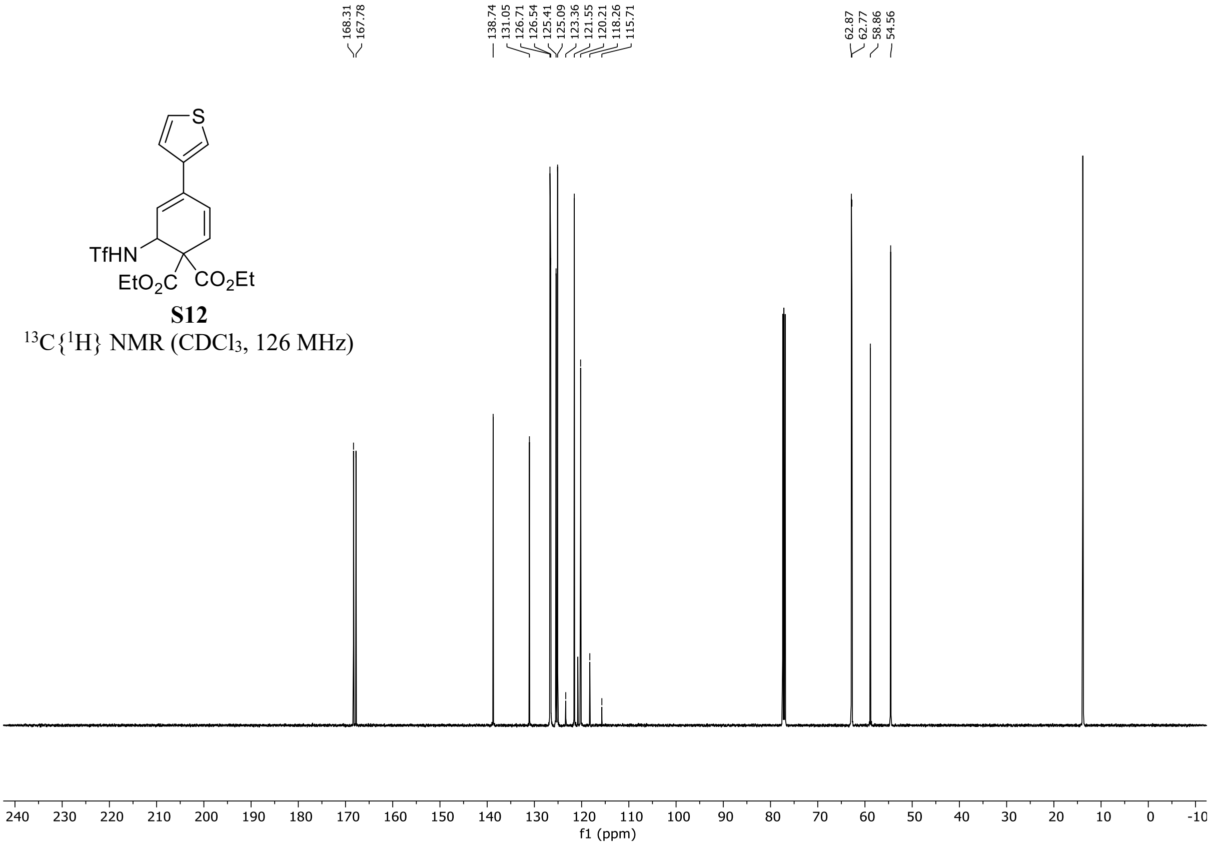

S12

$^{13}C\{^1H\}$ NMR ($CDCl_3$, 126 MHz)

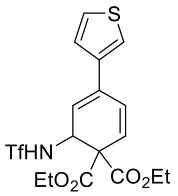

S12

$^{19}F\{^{1}H\}$ NMR ($CDCl_3$, 377 MHz)

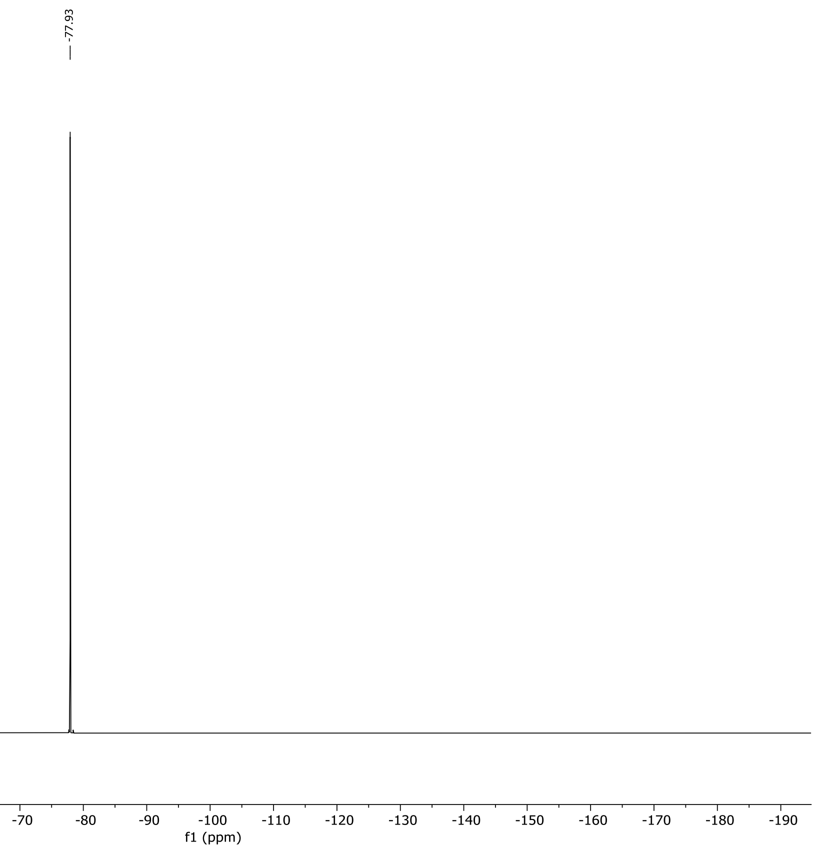

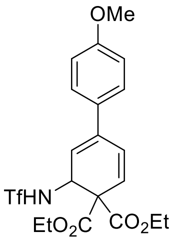

S14

^{1}H NMR (CDCl3, 500 MHz)

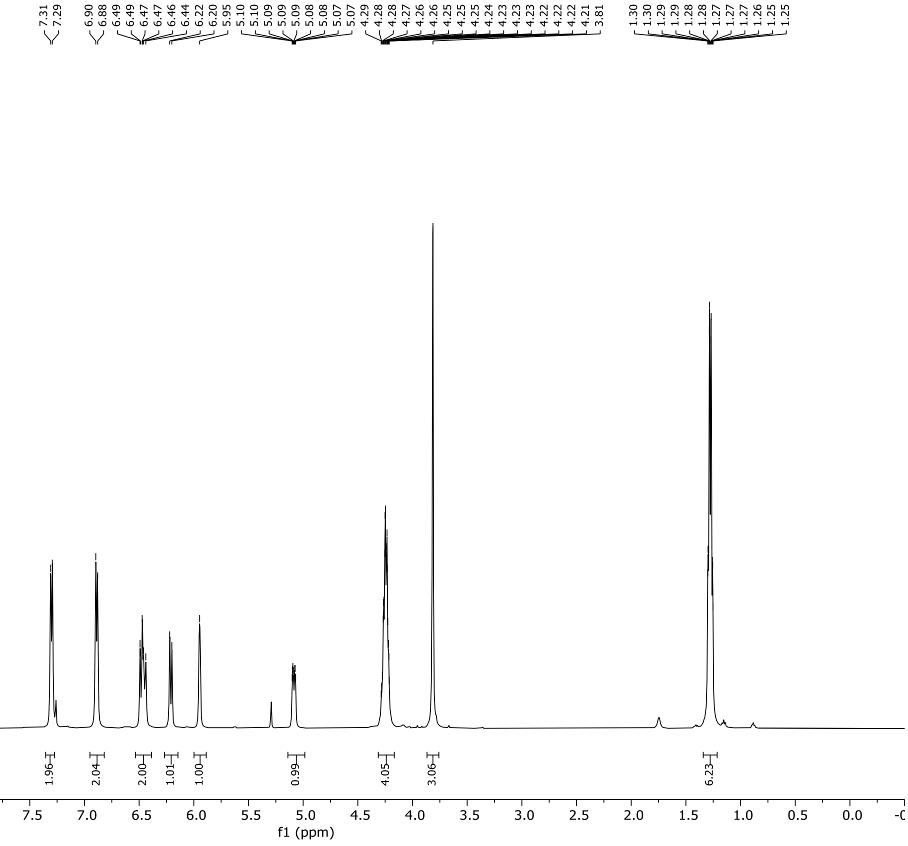

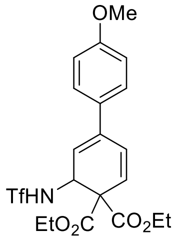

S14

$^{13}C\{^1H\}$ NMR (CDCl3, 126 MHz)

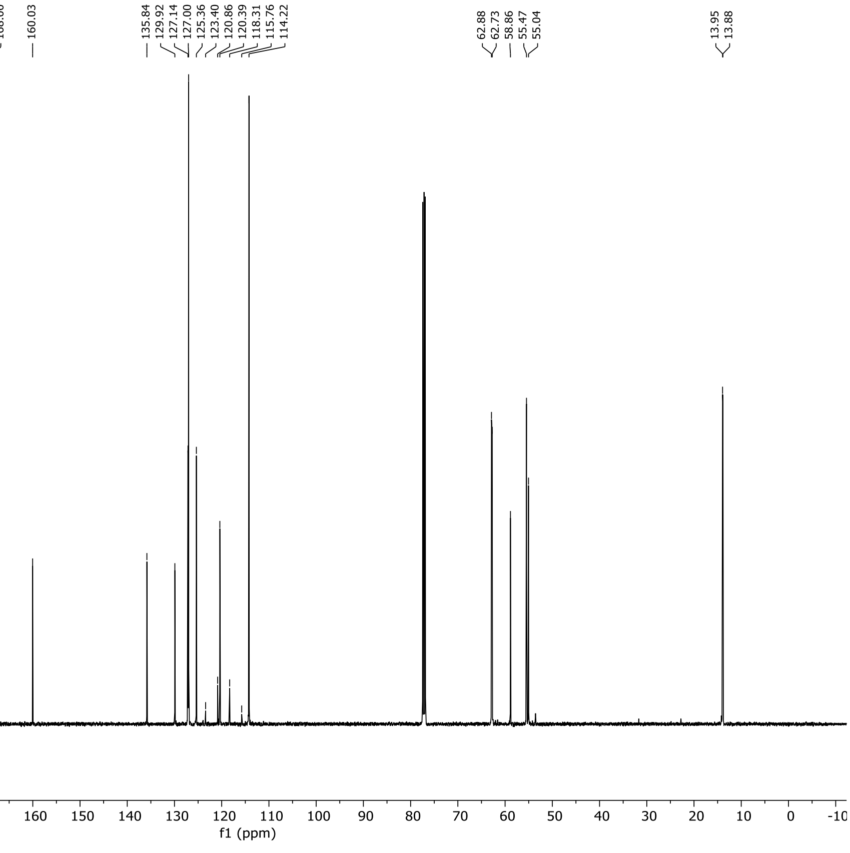

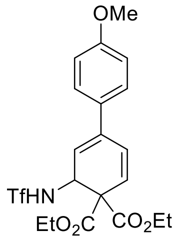

S14

$^{19}F\{^1H\}$ NMR ($CDCl_3$, 377 MHz)

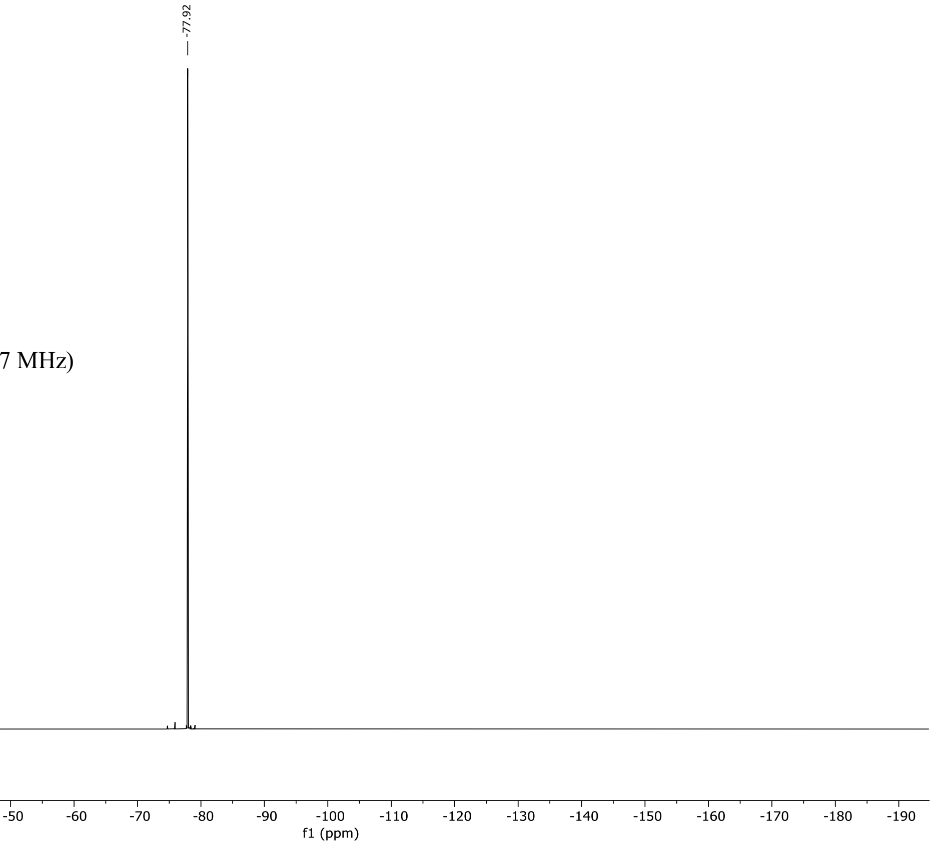

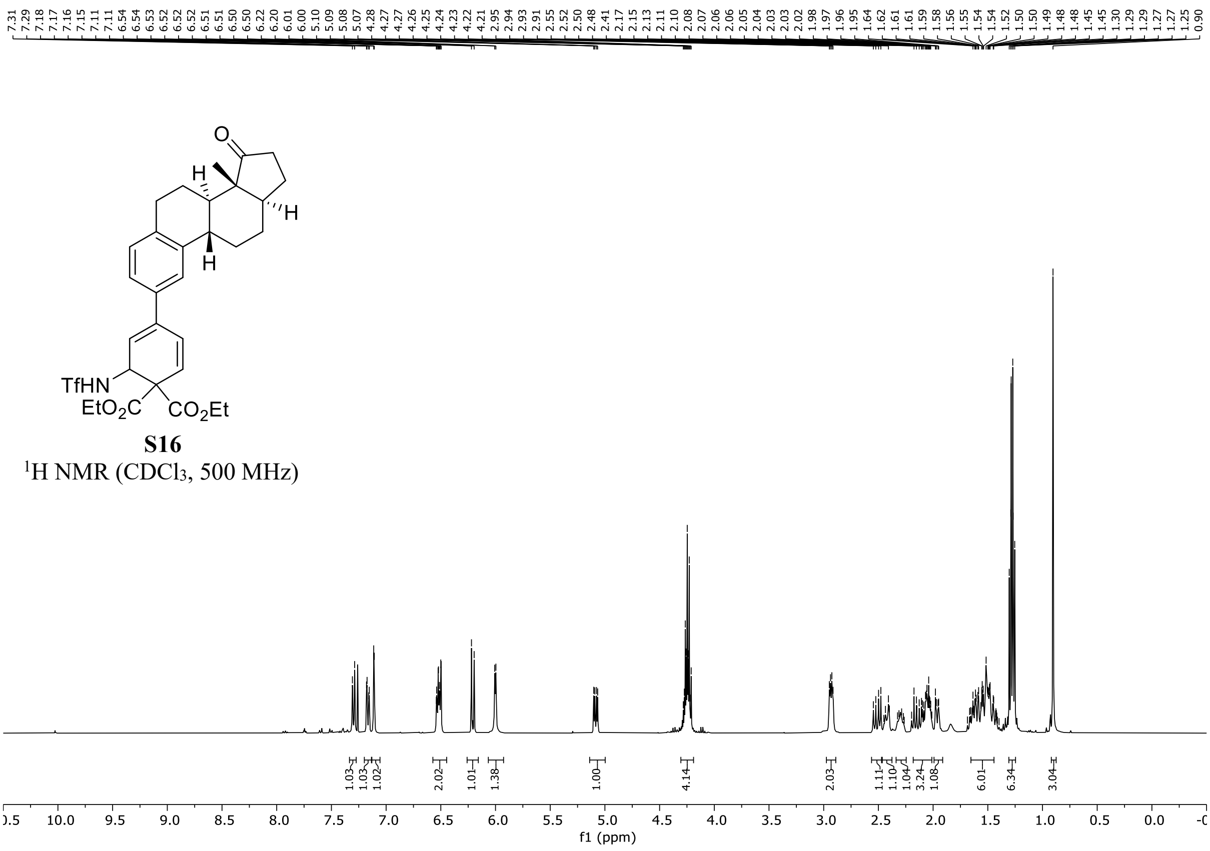

S16

^{1}H NMR ($CDCl_3$, 500 MHz)

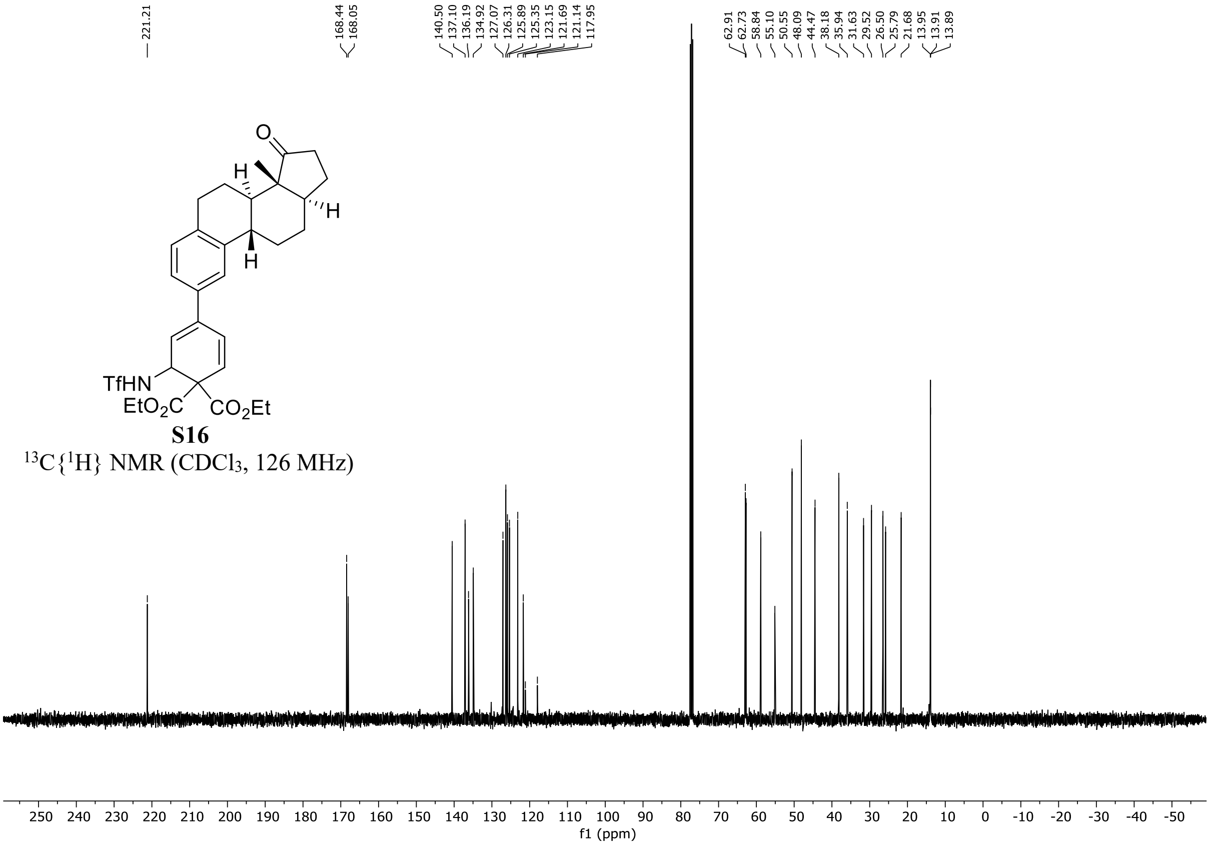

S16

$^{13}C\{^1H\}$ NMR ($CDCl_3$, 126 MHz)

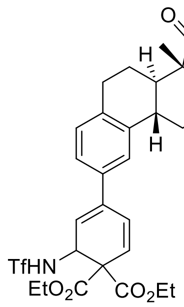

S16

$^{19}F\{^{1}H\}$ NMR ($CDCl_3$, 377 MHz)

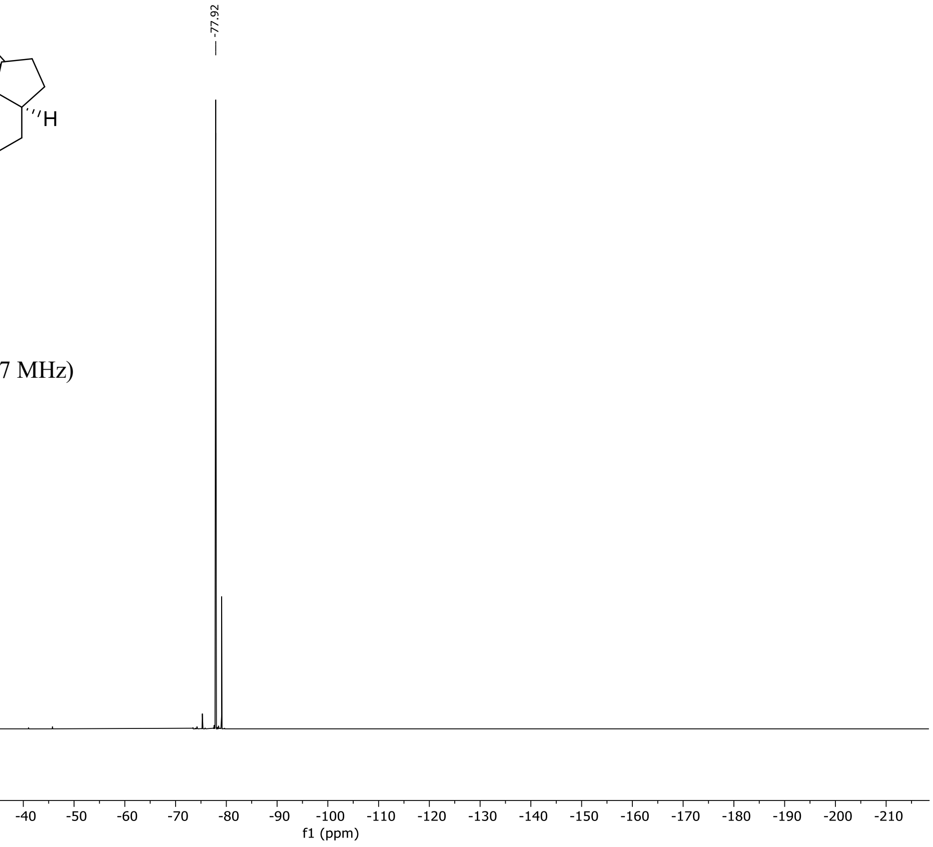

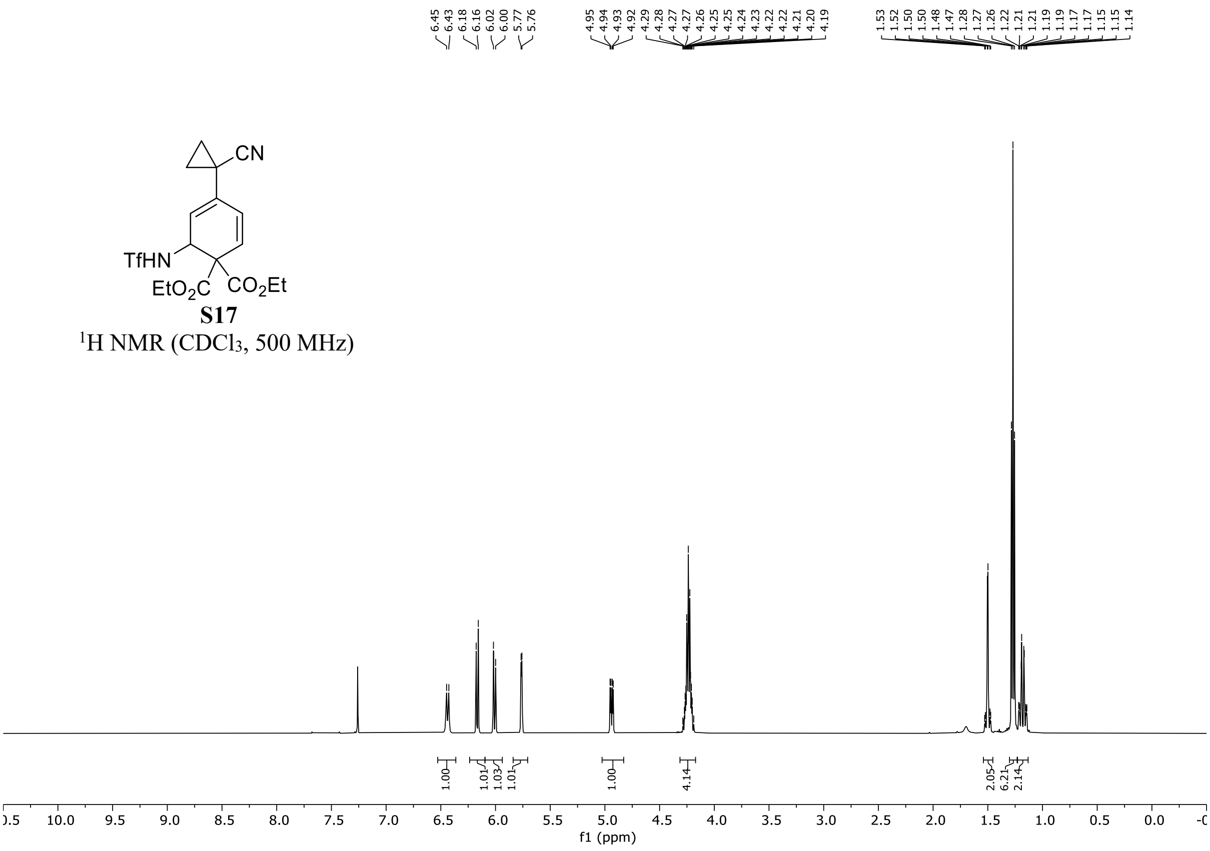

S17
^{1}H NMR ($CDCl_3$, 500 MHz)

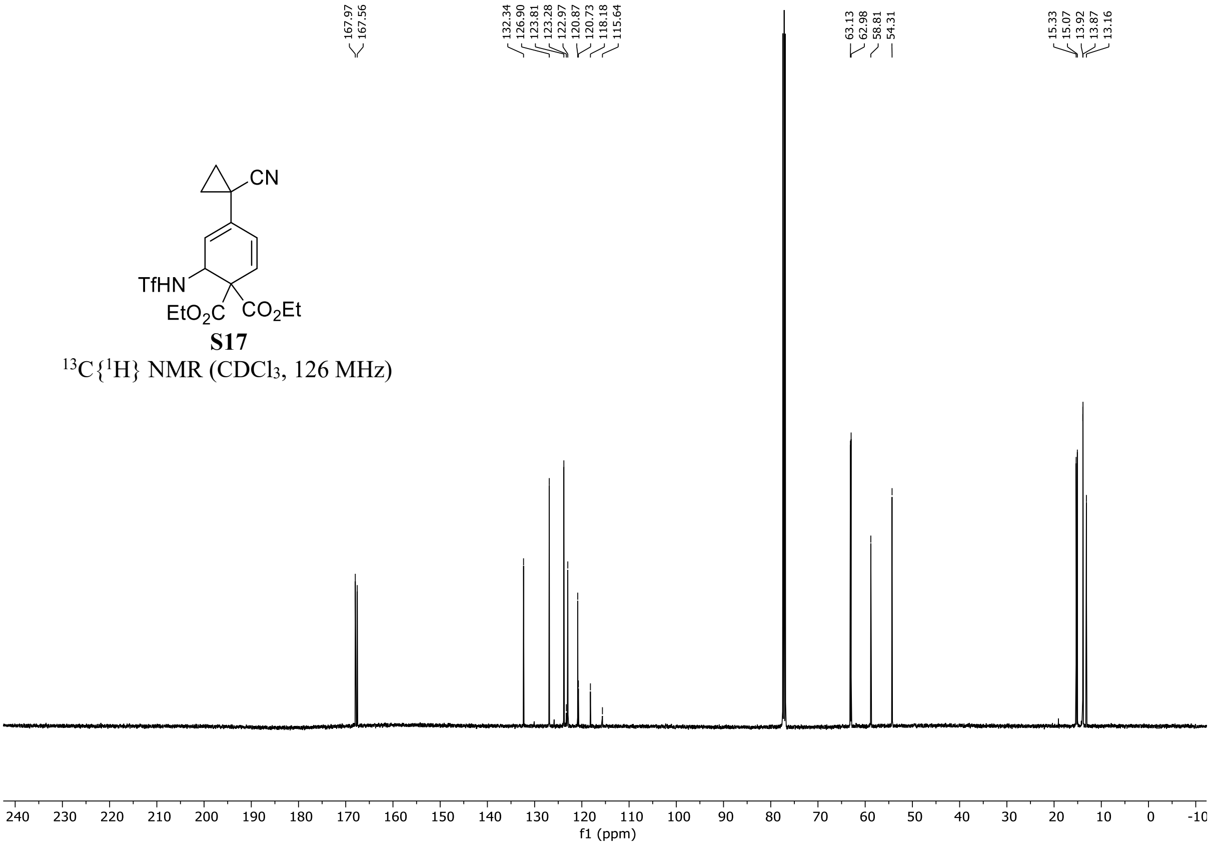

S17
$^{13}C\{^1H\}$ NMR ($CDCl_3$, 126 MHz)

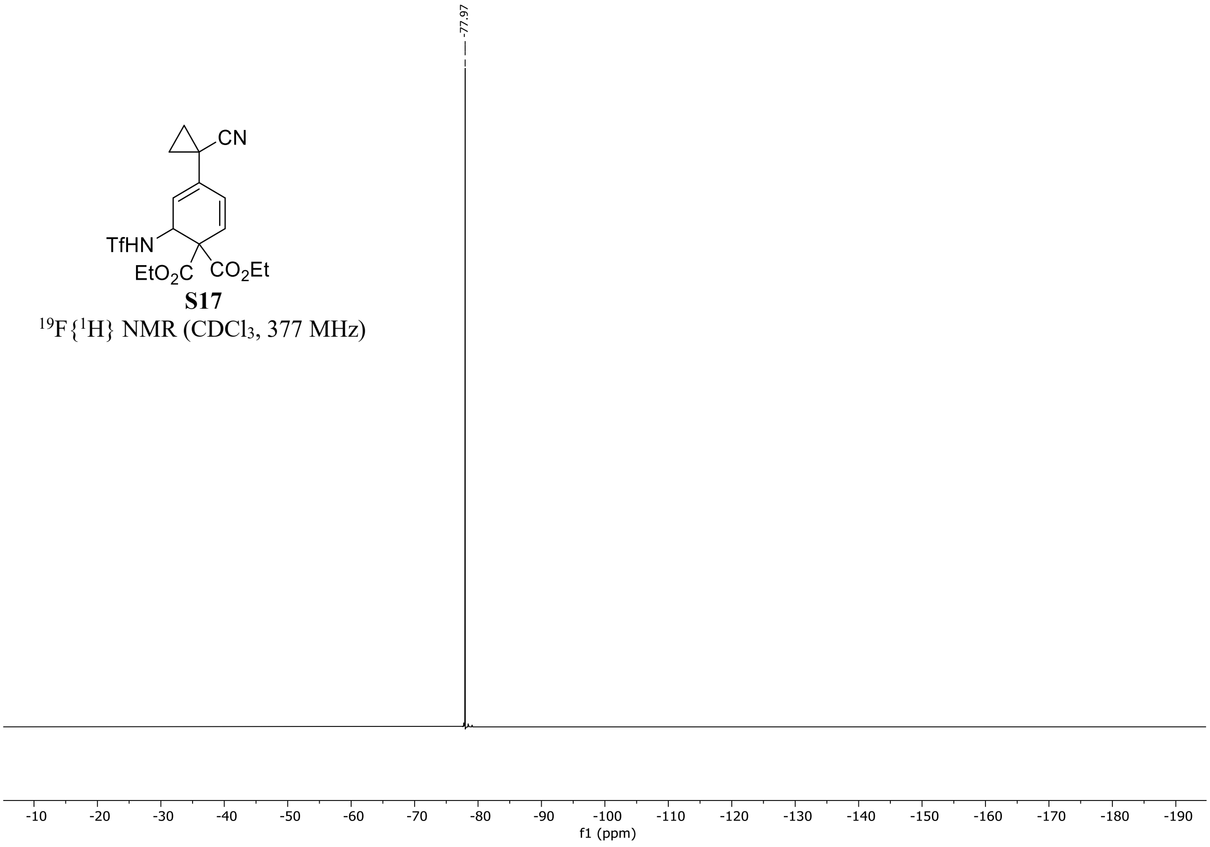

S17

$^{19}F\{^{1}H\}$ NMR ($CDCl_3$, 377 MHz)

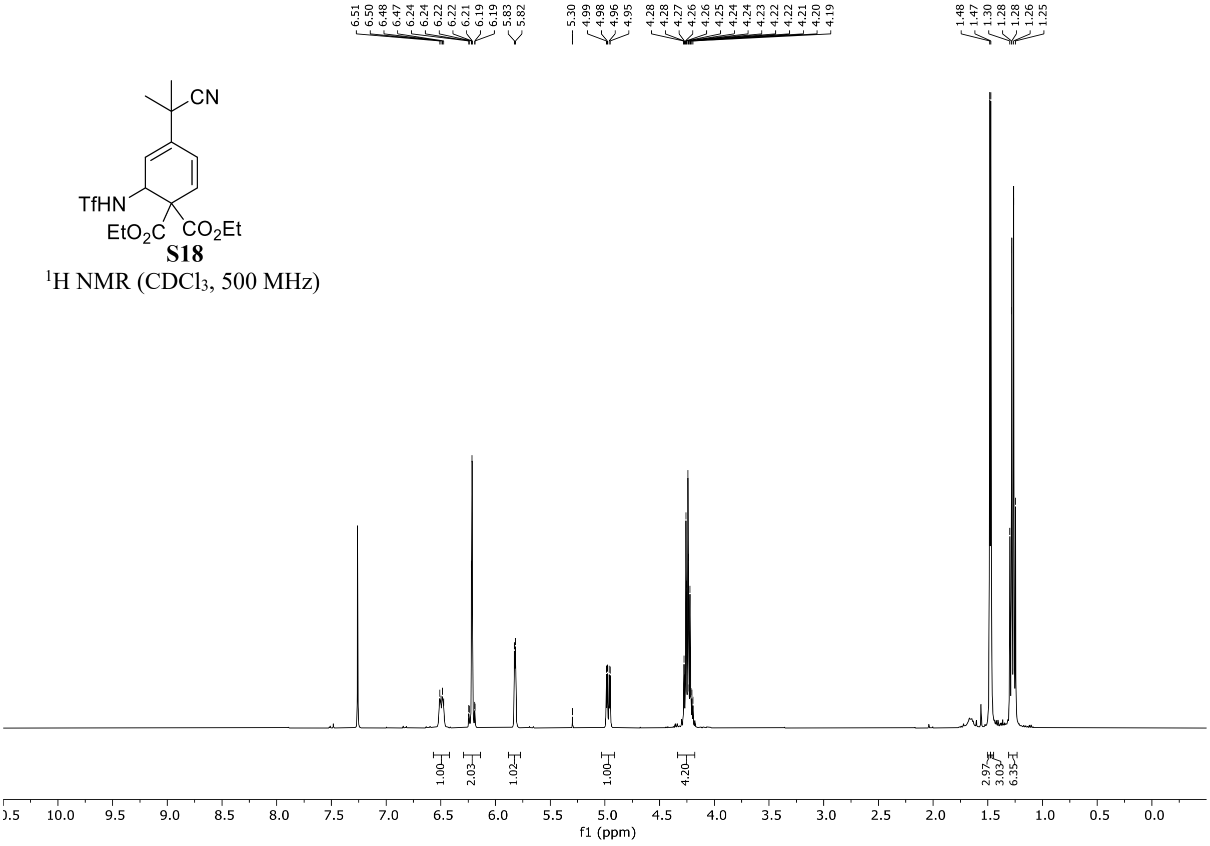
6.51 6.50 6.48 6.47 6.24 6.24 6.22 6.21 6.19 6.19 5.83 5.82
5.30 4.99 4.98 4.96 4.95
4.28 4.28 4.27 4.26 4.26 4.25 4.24 4.24 4.23 4.22 4.22 4.21 4.20 4.19
1.48 1.47 1.30 1.28 1.28 1.26 1.25
CN
TfHN
EtO2C
CO2Et
S18
1H NMR (CDCl3, 500 MHz)
1.00
2.03
1.02
1.00
4.20
2.97
3.03
6.35
f1 (ppm)

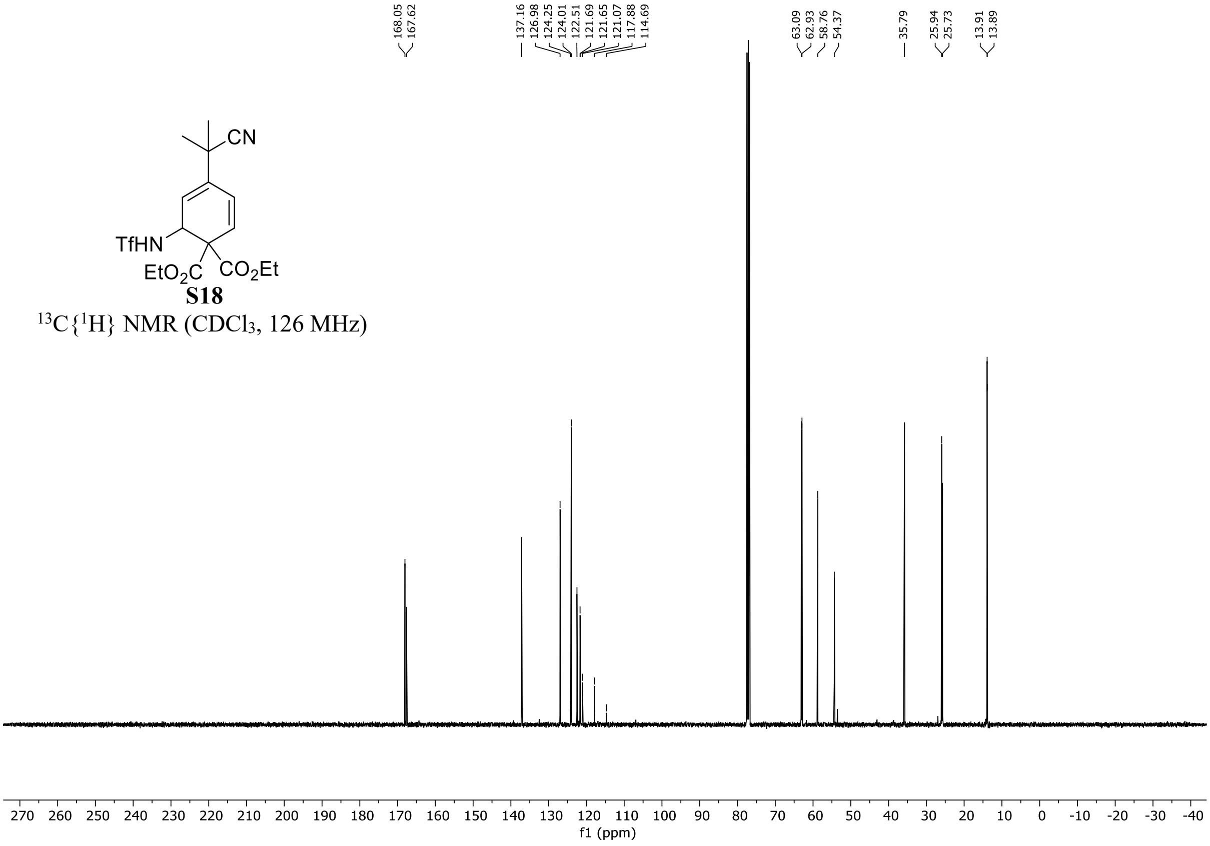
168.05 167.62
137.16 126.98 124.25 124.01 122.51 121.69 121.65 121.07 117.88 114.69
63.09 62.93 58.76 54.37
35.79
25.94 25.73
13.91 13.89
CN
TfHN
EtO2C
CO2Et
S18
13C{1H} NMR (CDCl3, 126 MHz)
f1 (ppm)

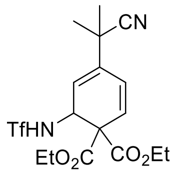

S18

$^{19}F\{^{1}H\}$ NMR ($CDCl_3$, 377 MHz)

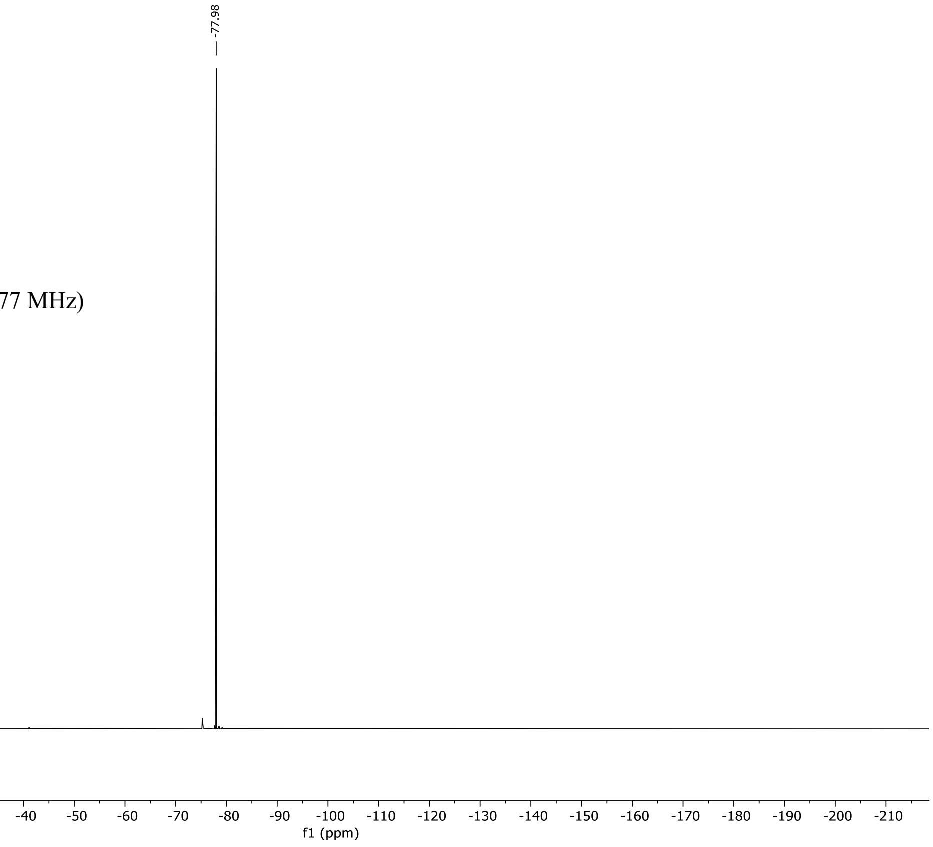

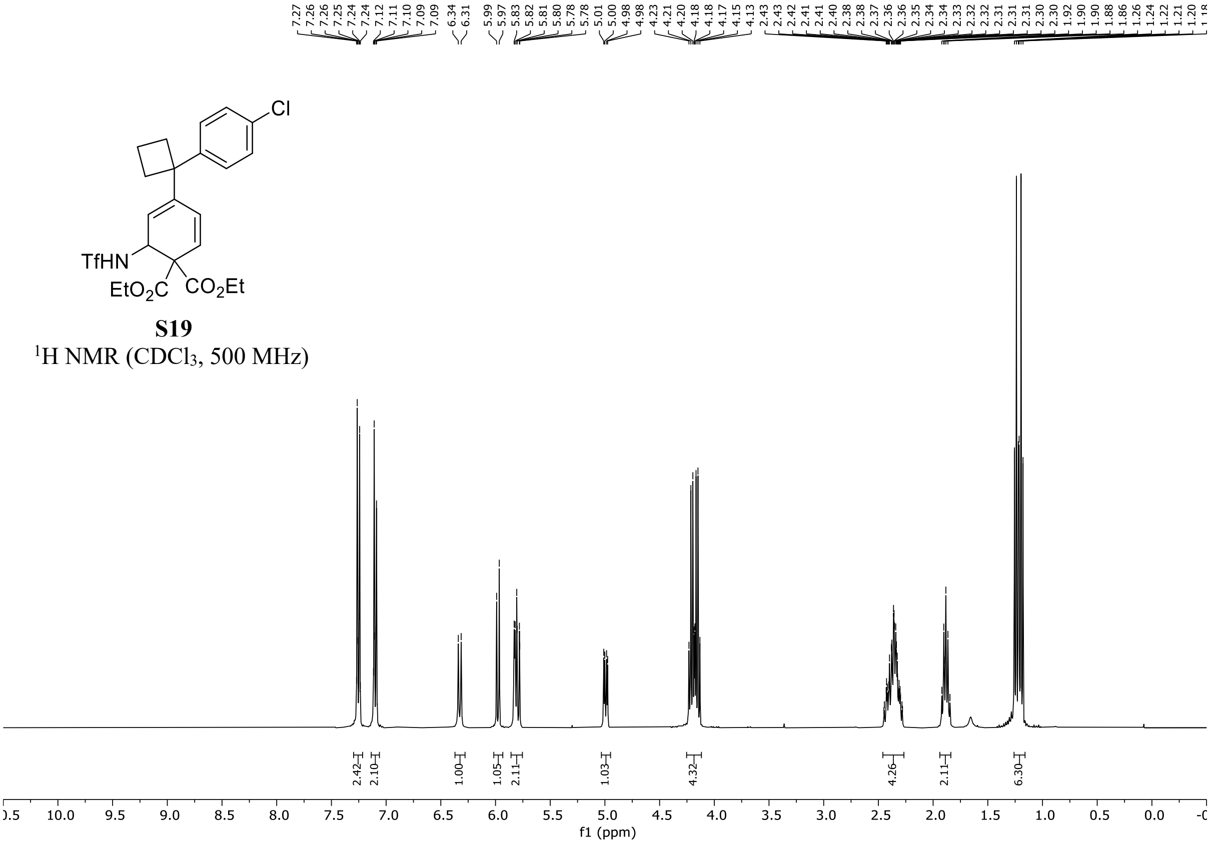

S19
^{1}H NMR ($CDCl_3$, 500 MHz)

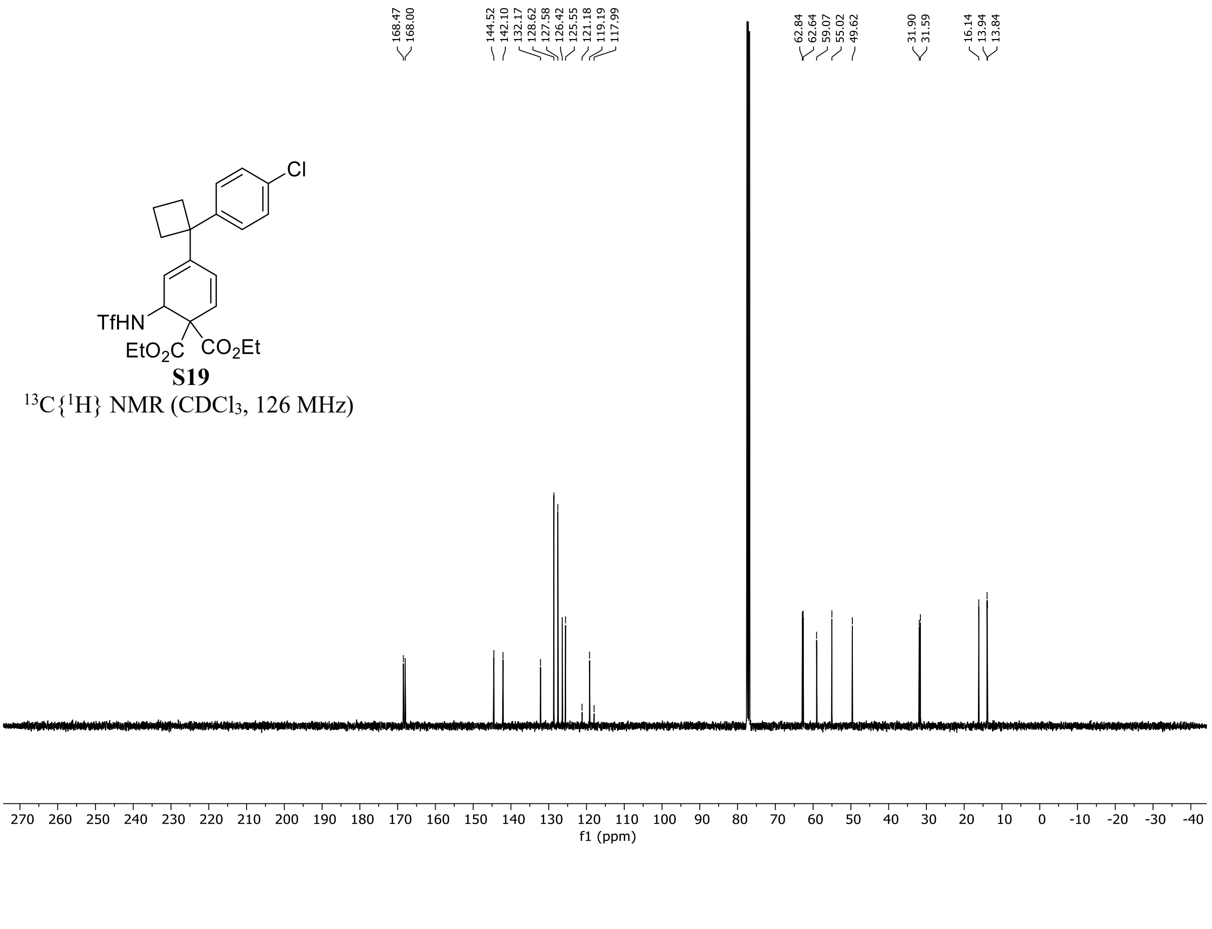

S19
$^{13}C\{^1H\}$ NMR ($CDCl_3$, 126 MHz)

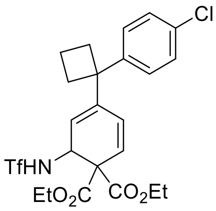

S19

$^{19}F\{^{1}H\}$ NMR ($CDCl_3$, 377 MHz)

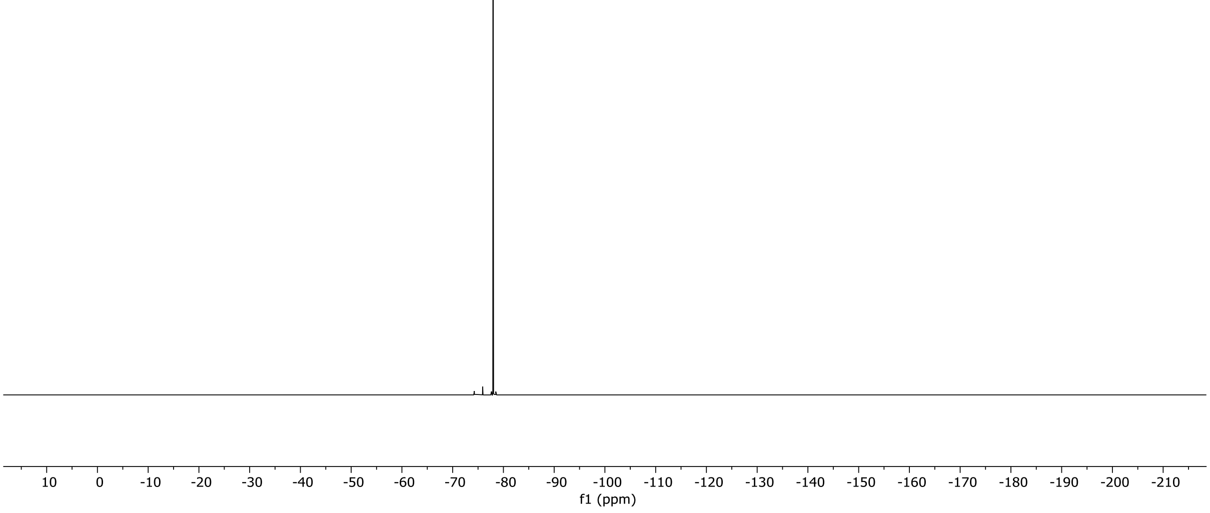

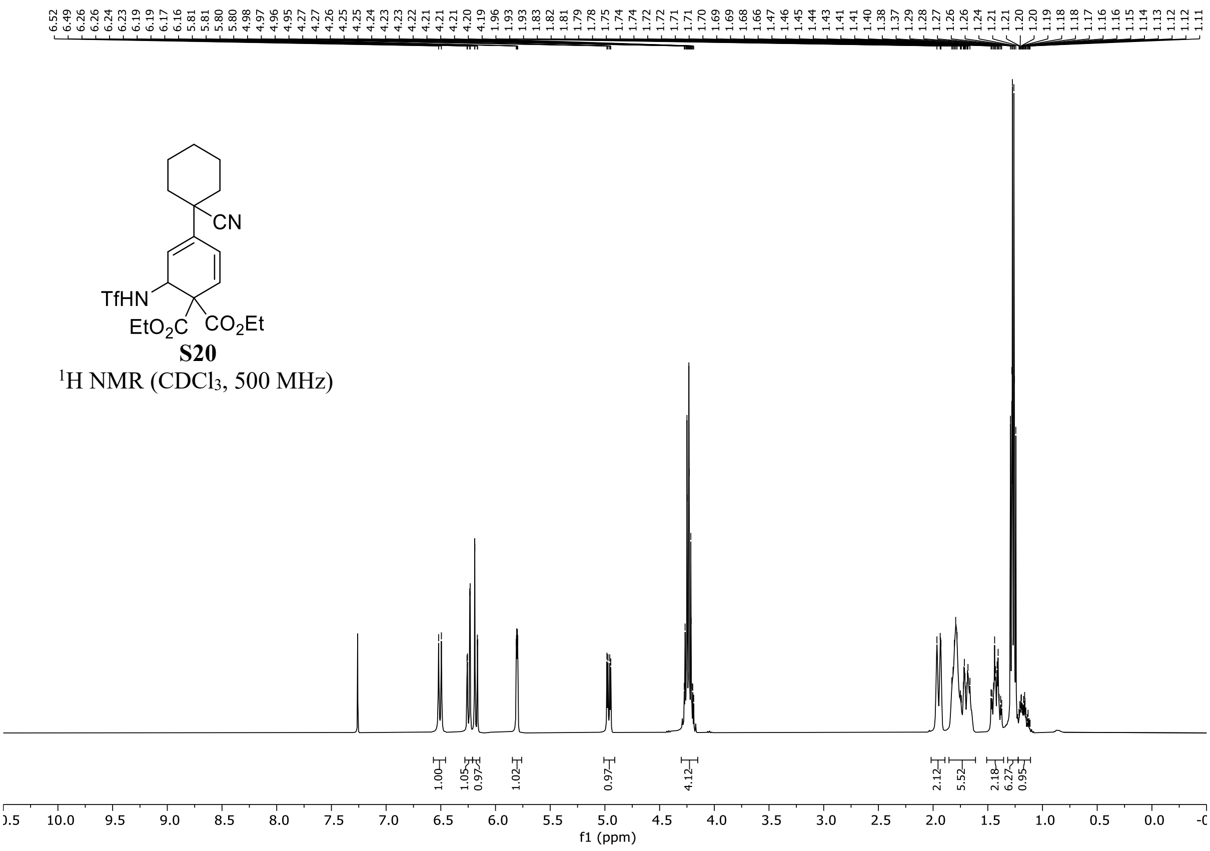

S20

^{1}H NMR ($CDCl_3$, 500 MHz)

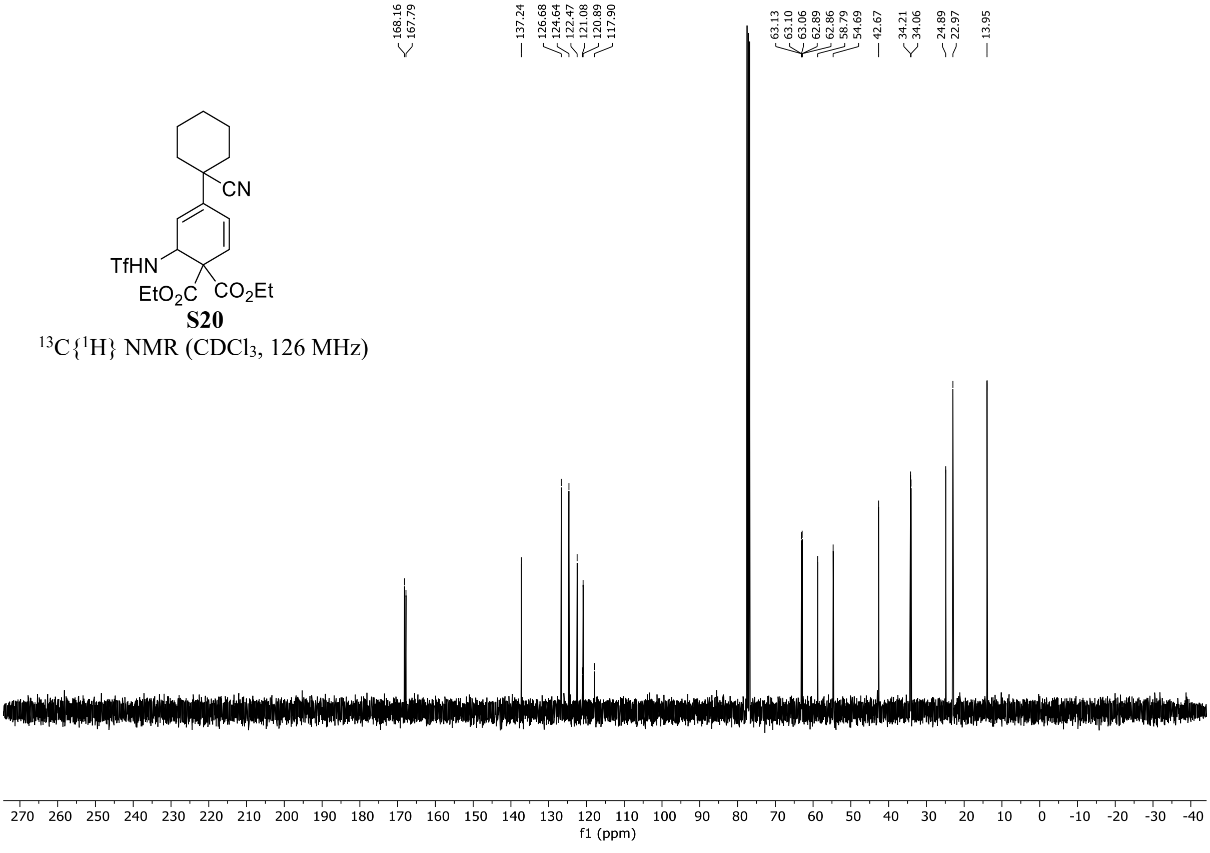

S20

^{13}C{^{1}H} NMR ($CDCl_3$, 126 MHz)

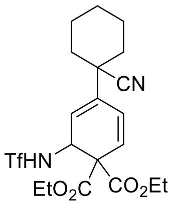

S20

$^{19}F\{^1H\}$ NMR ($CDCl_3$, 377 MHz)

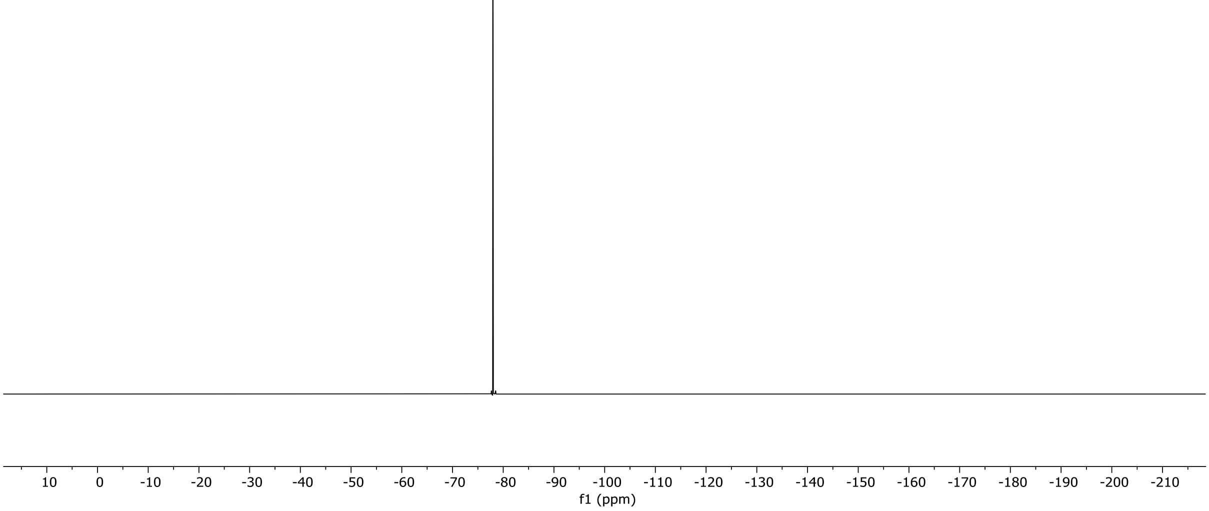

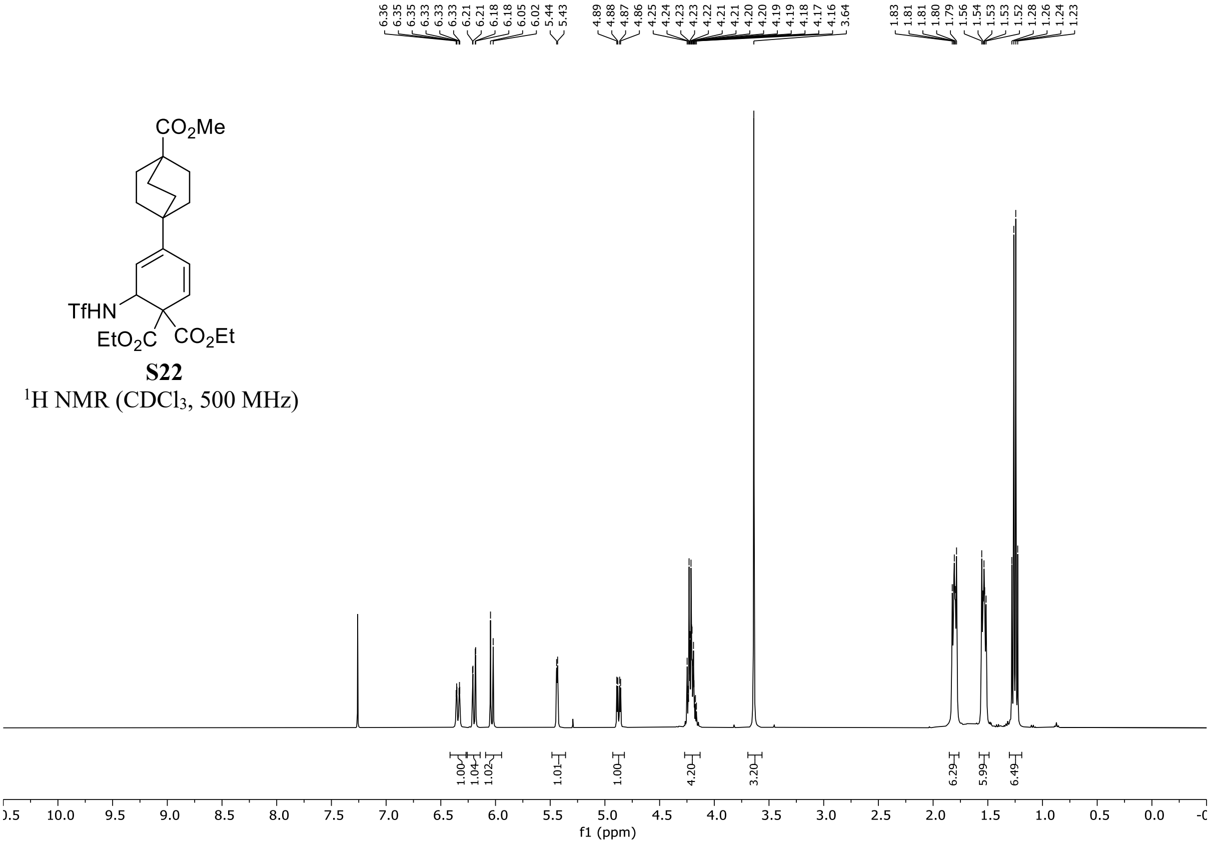

S22

^{1}H NMR ($CDCl_3$, 500 MHz)

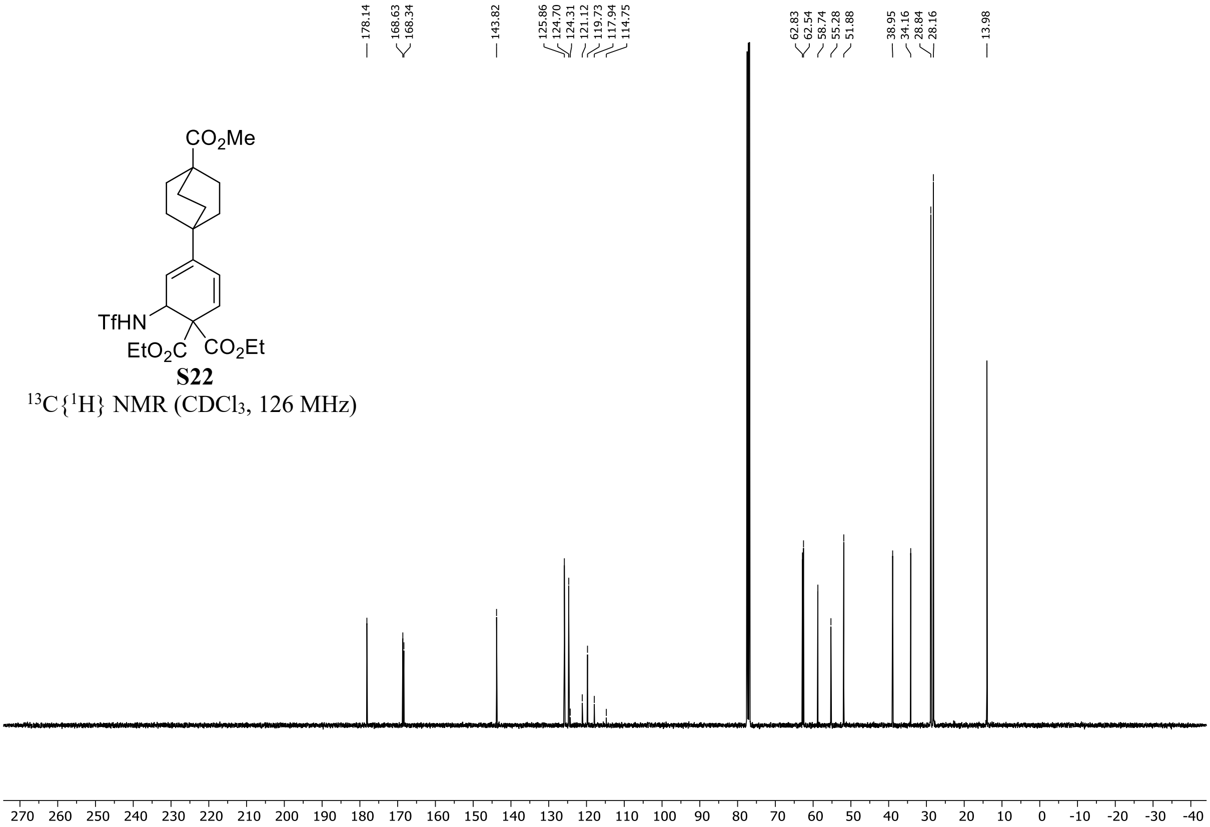

S22

$^{13}C\{^1H\}$ NMR ($CDCl_3$, 126 MHz)

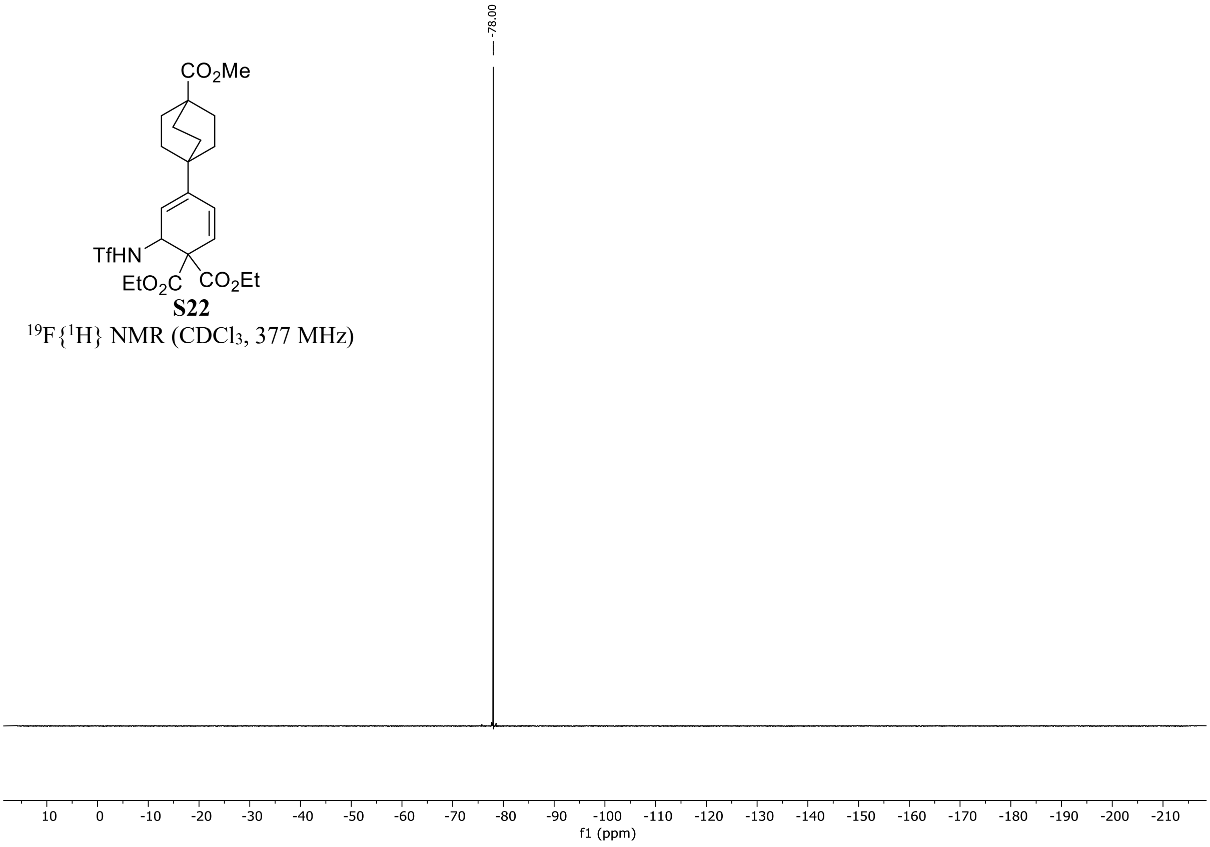

S22

$^{19}F\{^{1}H\}$ NMR ($CDCl_3$, 377 MHz)

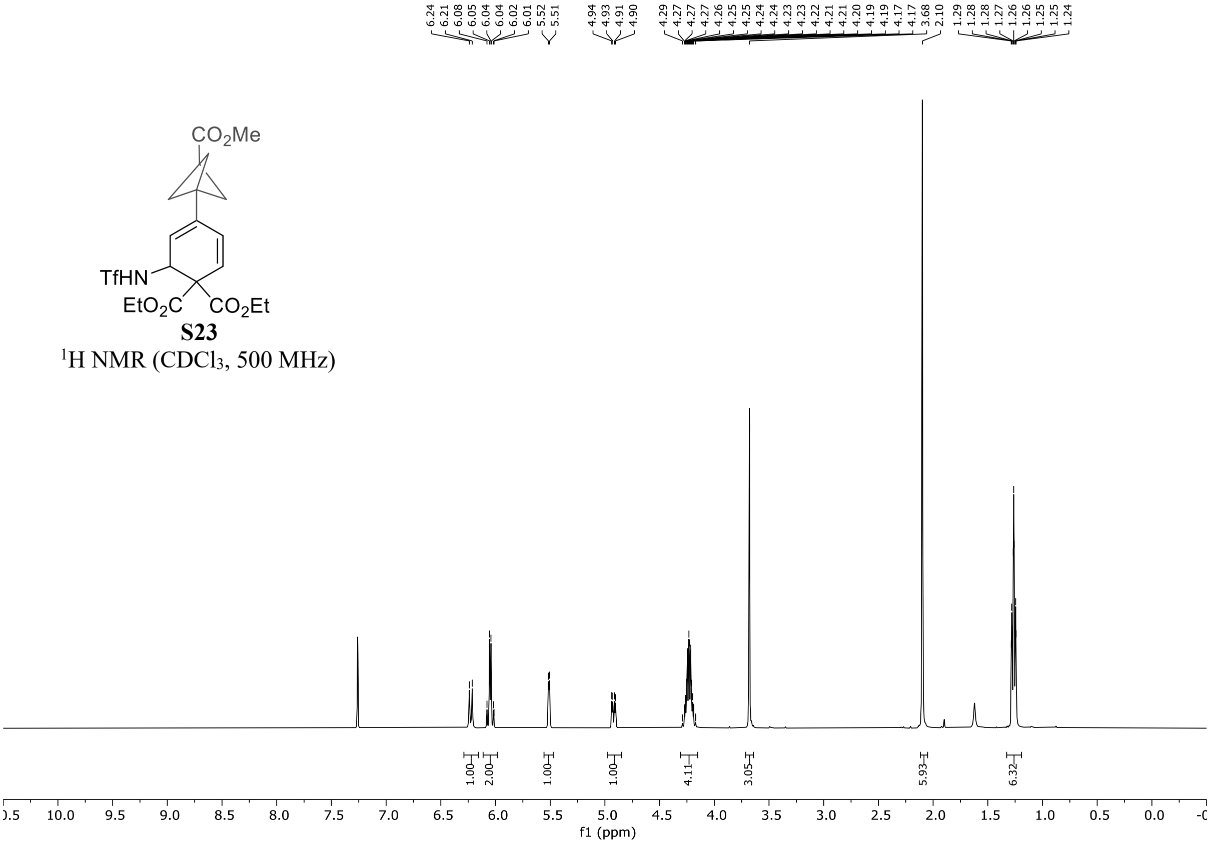

CO2Me
TfHN
EtO2C
CO2Et
S23
1H NMR (CDCl3, 500 MHz)
6.24 6.21 6.08 6.05 6.04 6.04 6.02 6.01 5.52 5.51 4.94 4.93 4.91 4.90 4.29 4.27 4.27 4.27 4.26 4.25 4.25 4.24 4.24 4.23 4.23 4.22 4.21 4.21 4.20 4.19 4.19 4.17 4.17 3.68 2.10 1.29 1.28 1.28 1.27 1.26 1.26 1.25 1.25 1.24
1.00 2.00 1.00 1.00 4.11 3.05 5.93 6.32
f1 (ppm)

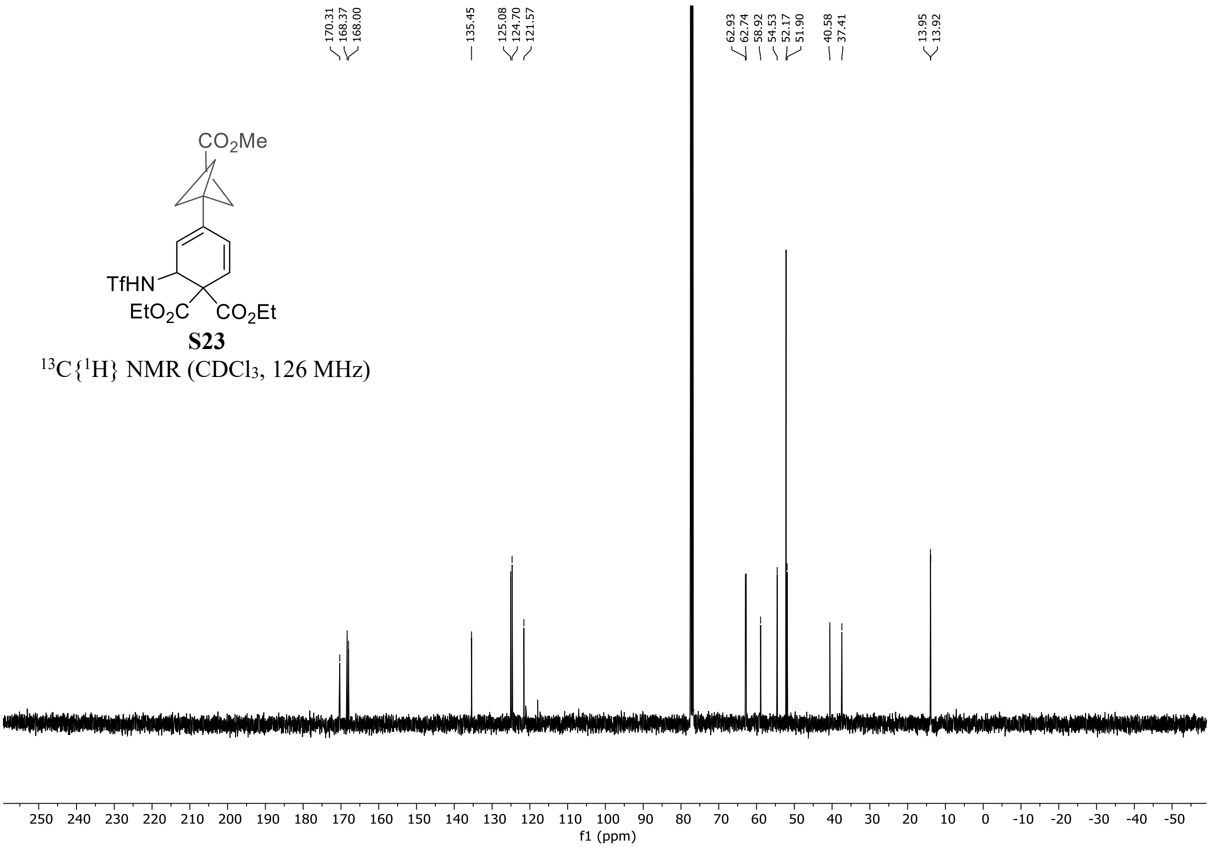

CO2Me
TfHN
EtO2C
CO2Et
S23
13C{1H} NMR (CDCl3, 126 MHz)
170.31 168.37 168.00 135.45 125.08 124.70 121.57 62.93 62.74 58.92 54.53 52.17 51.90 40.58 37.41 13.95 13.92
f1 (ppm)

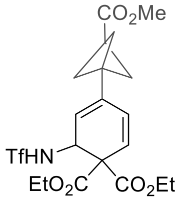

S23

$^{19}F\{^1H\}$ NMR ($CDCl_3$, 377 MHz)

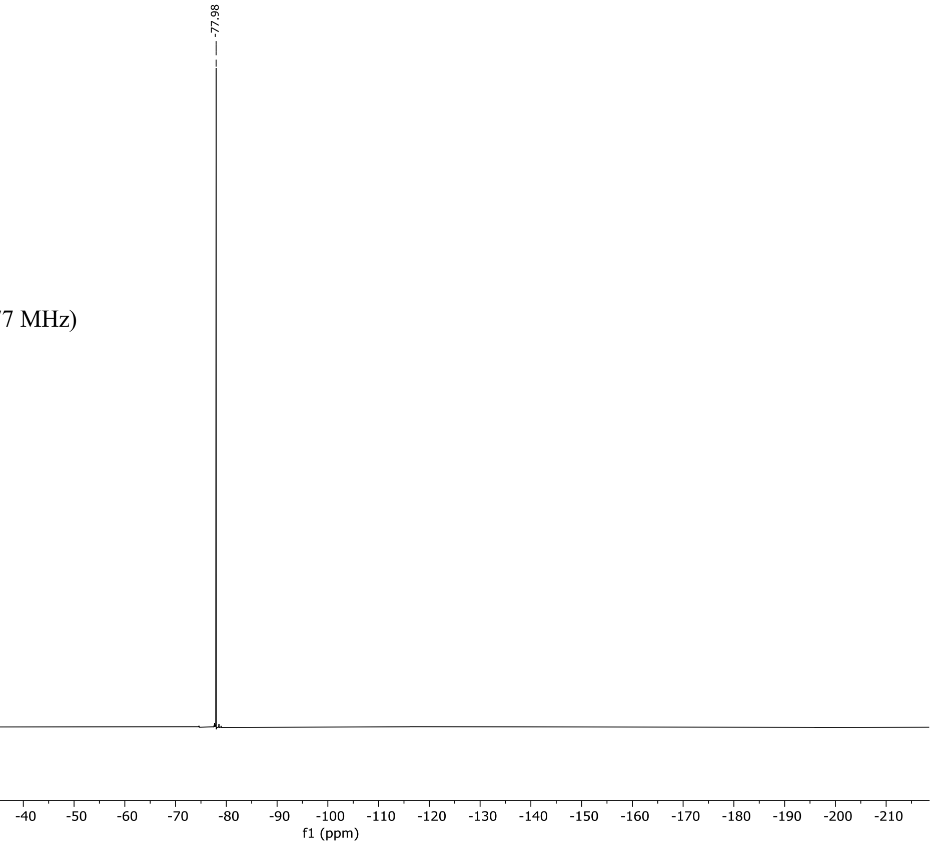

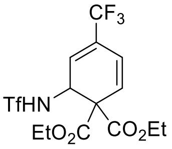

S24

^{1}H NMR ($CDCl_3$, 500 MHz)

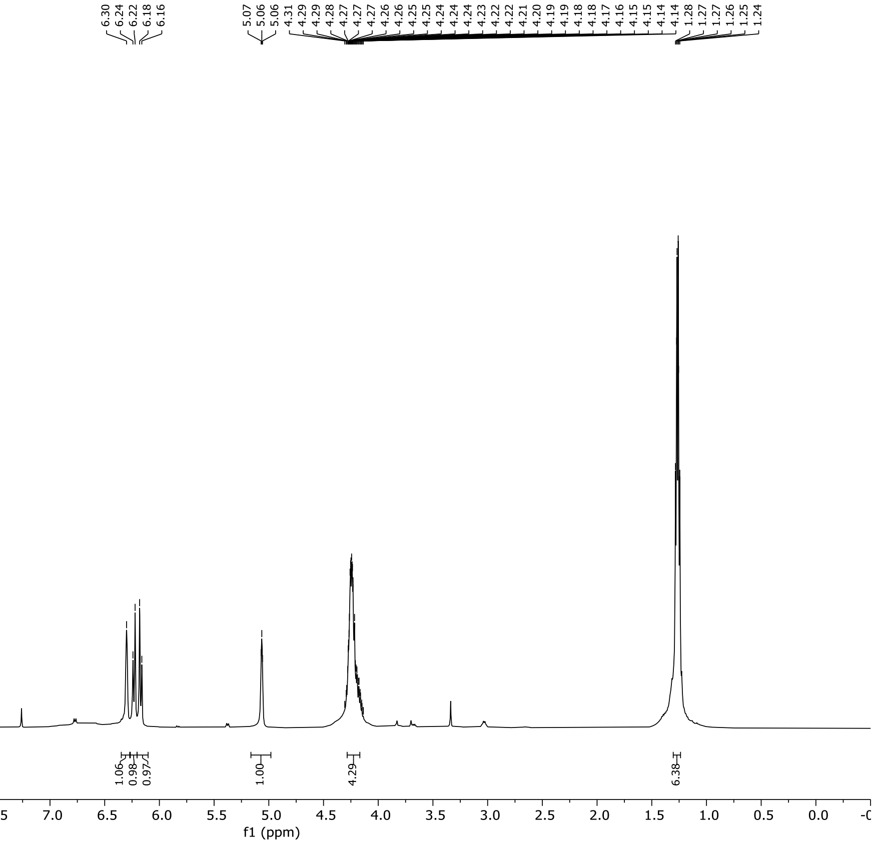

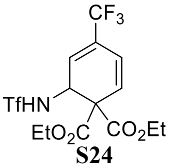

S24

$^{13}C\{^{1}H\}$ NMR ($CDCl_3$, 126 MHz)

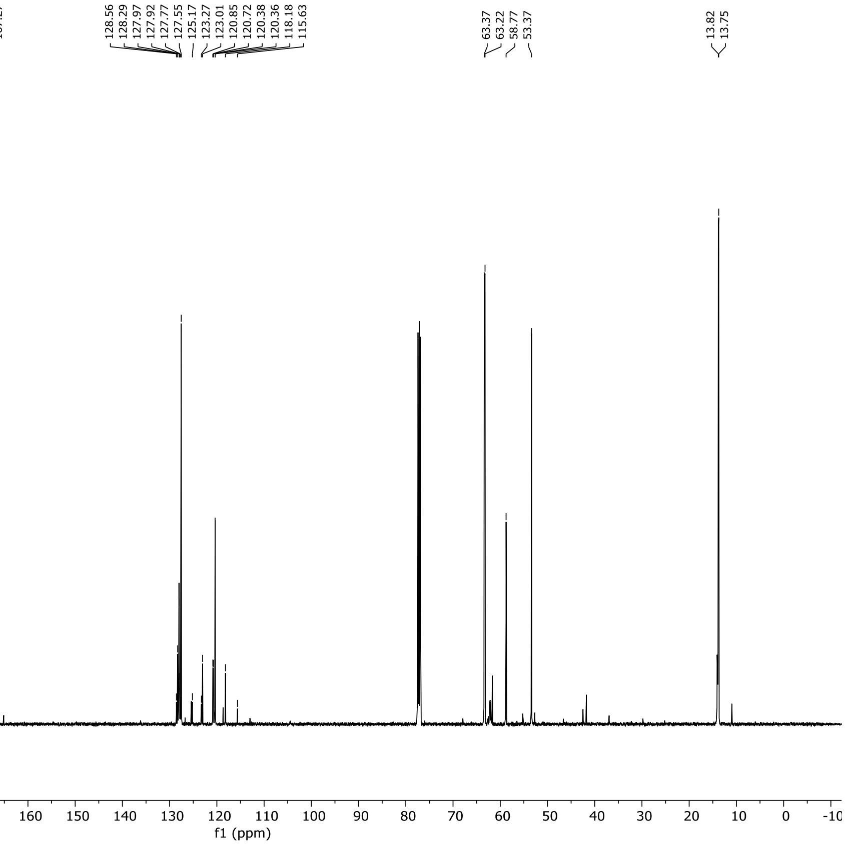

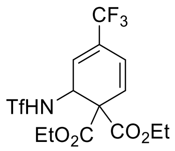

S24

$^{19}F\{^1H\}$ NMR ($CDCl_3$, 377 MHz)

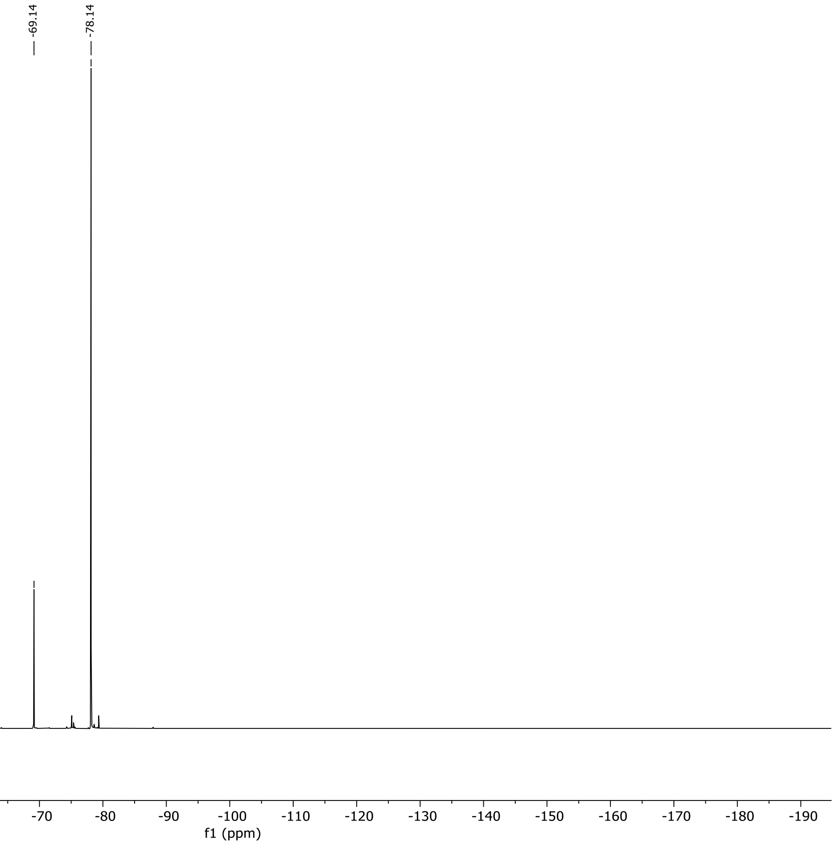

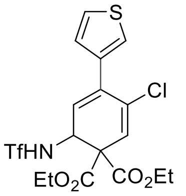

S27
^{1}H NMR ($CDCl_3$, 500 MHz)

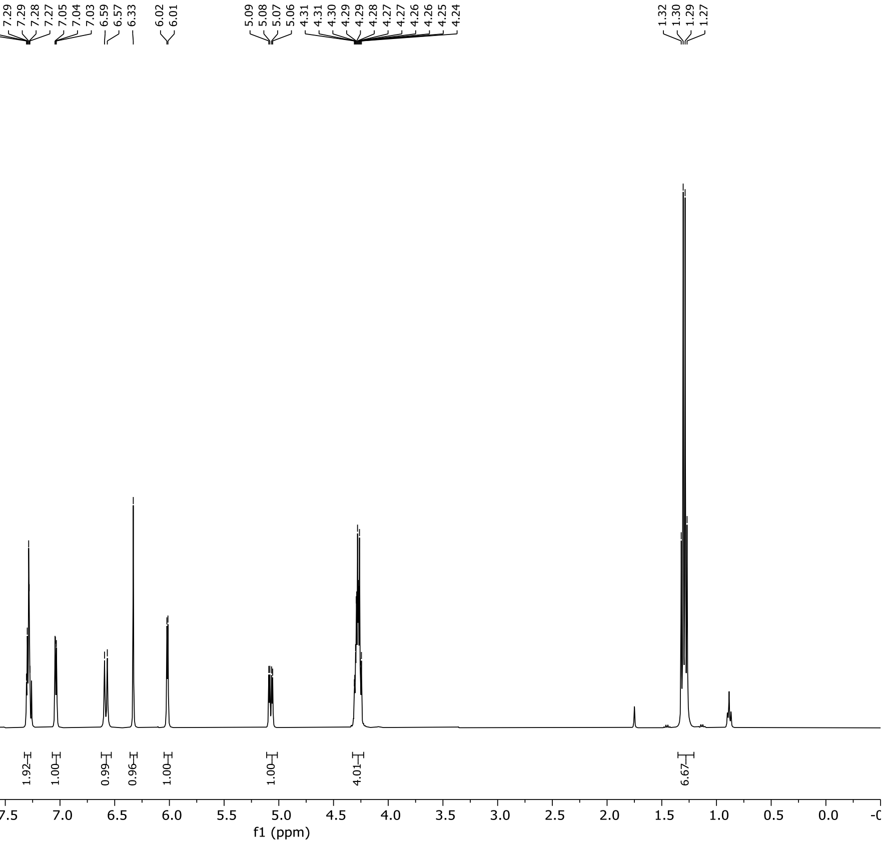

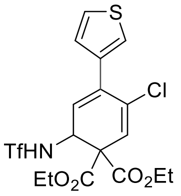

S27
^{13}C{^{1}H} NMR ($CDCl_3$, 126 MHz)

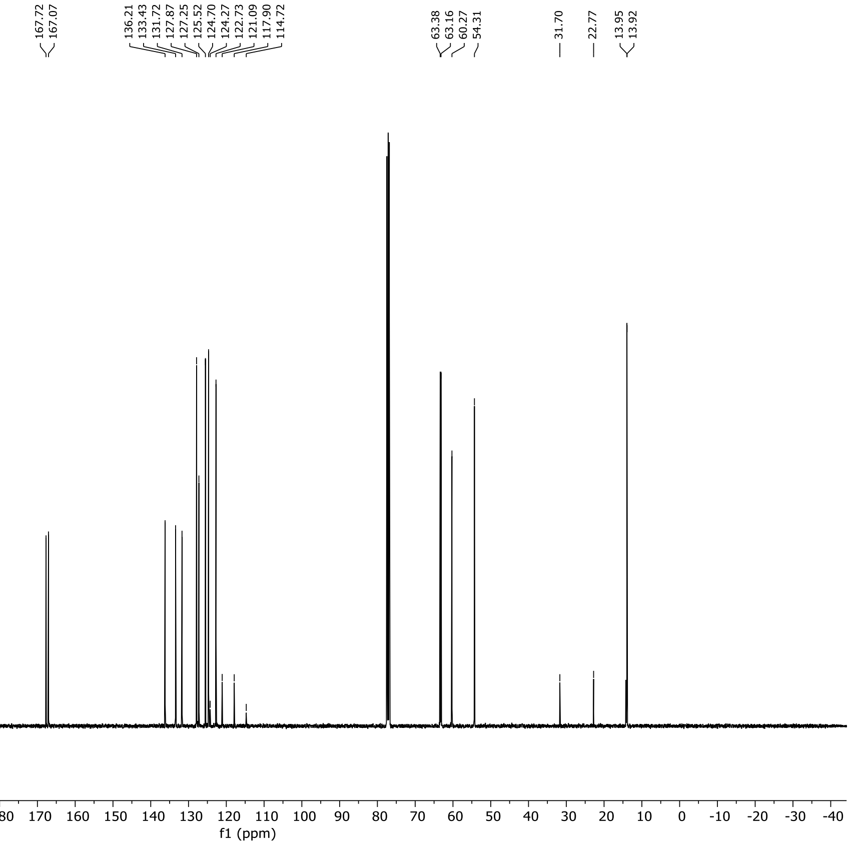

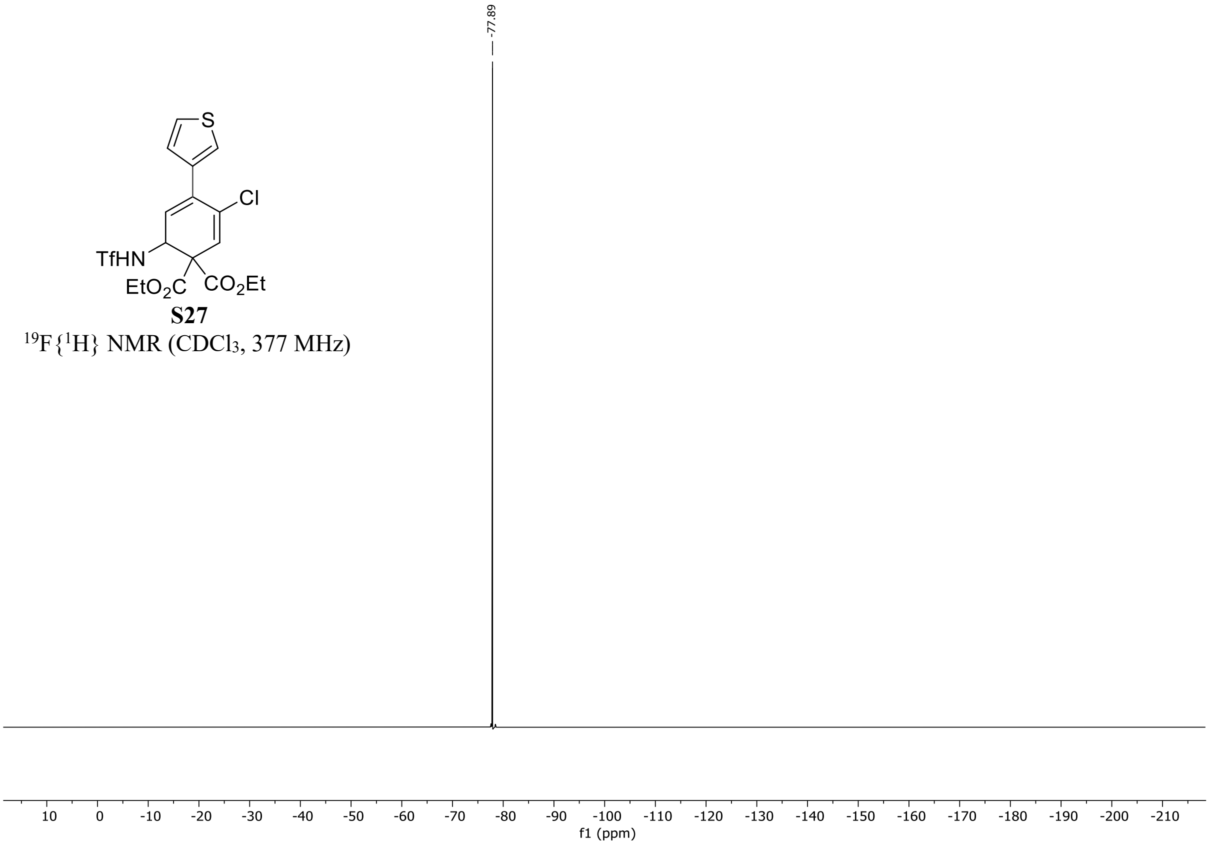

S27

$^{19}F\{^{1}H\}$ NMR ($CDCl_3$, 377 MHz)

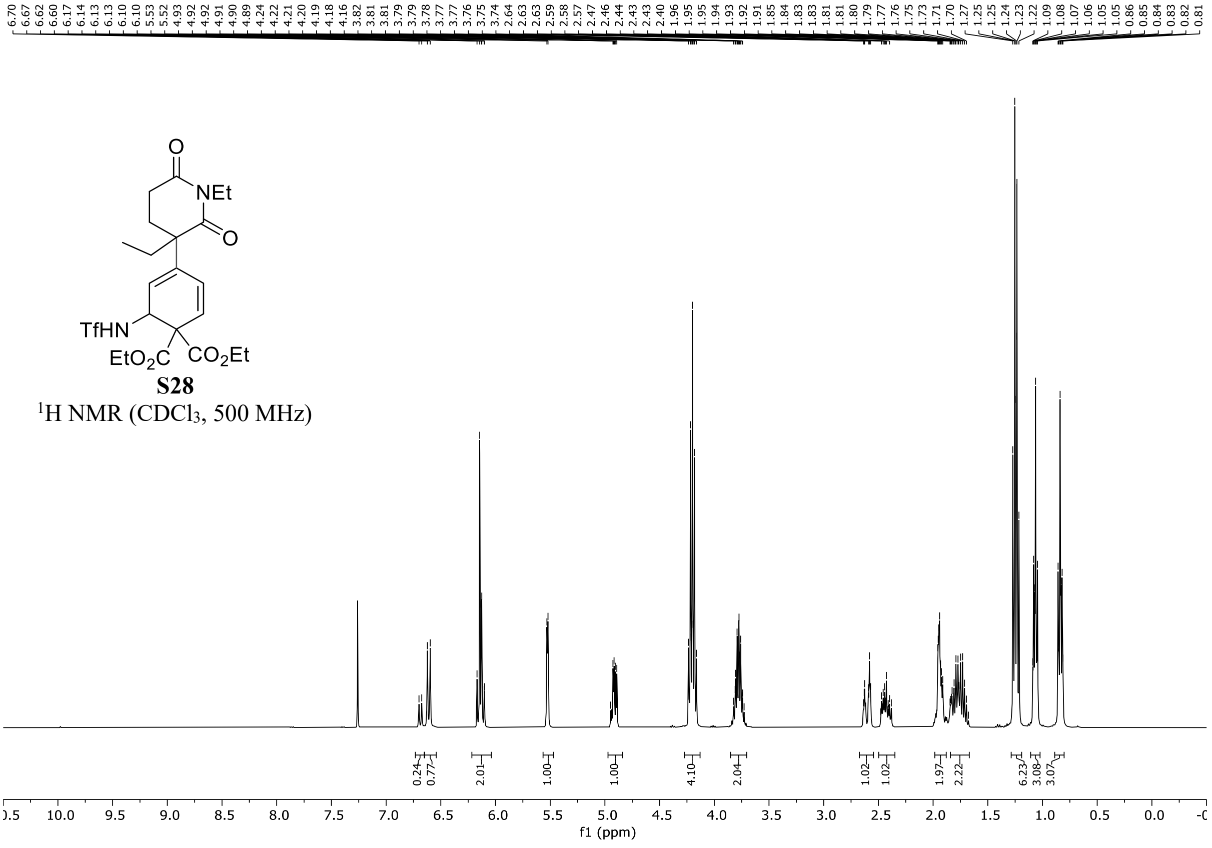

S28
^{1}H NMR ($CDCl_3$, 500 MHz)

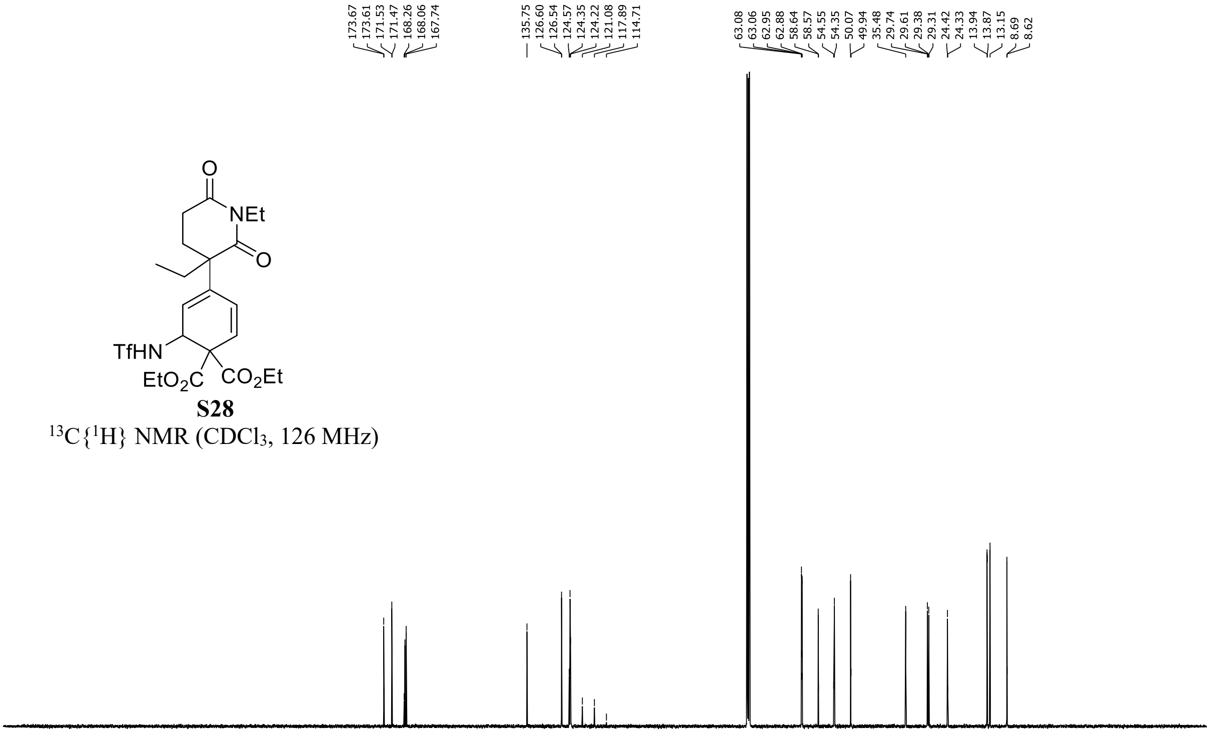

S28
^{13}C{^{1}H} NMR ($CDCl_3$, 126 MHz)

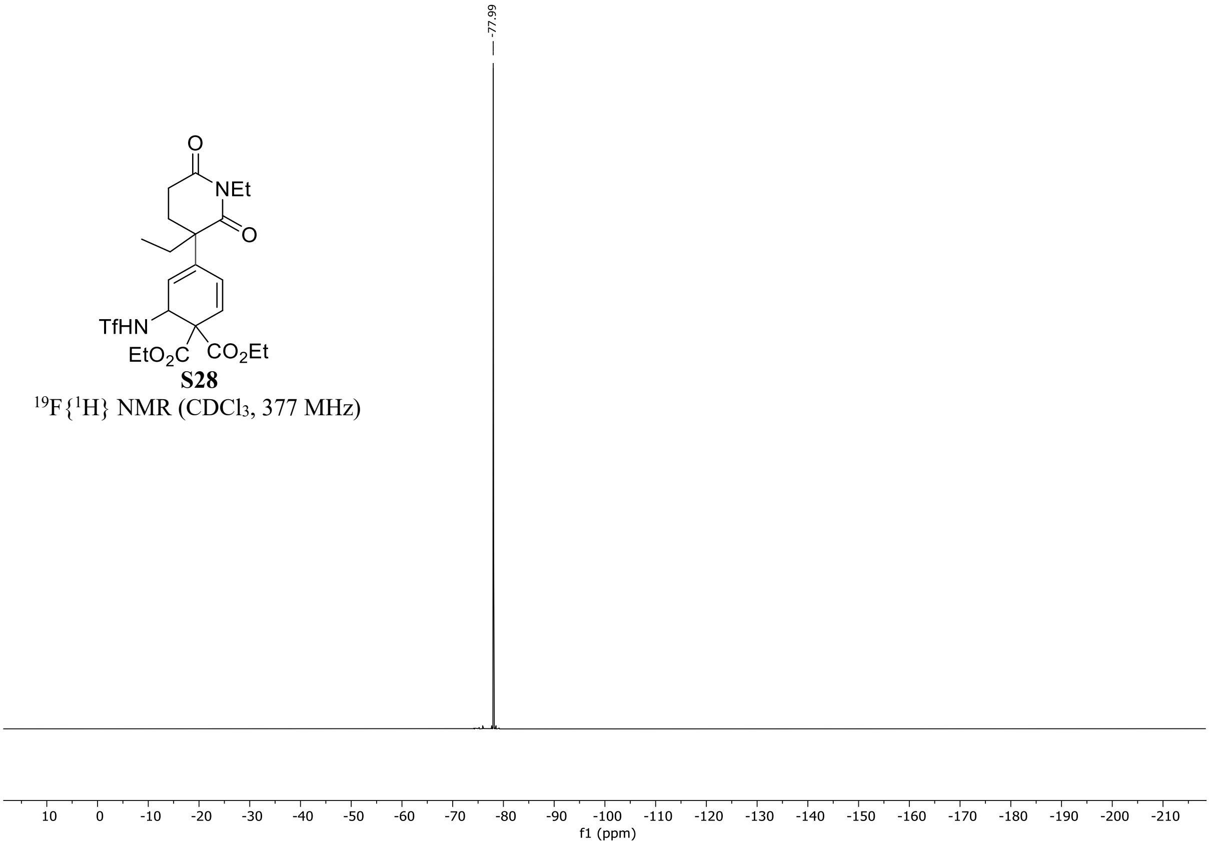

S28

$^{19}F\{^1H\}$ NMR ($CDCl_3$, 377 MHz)

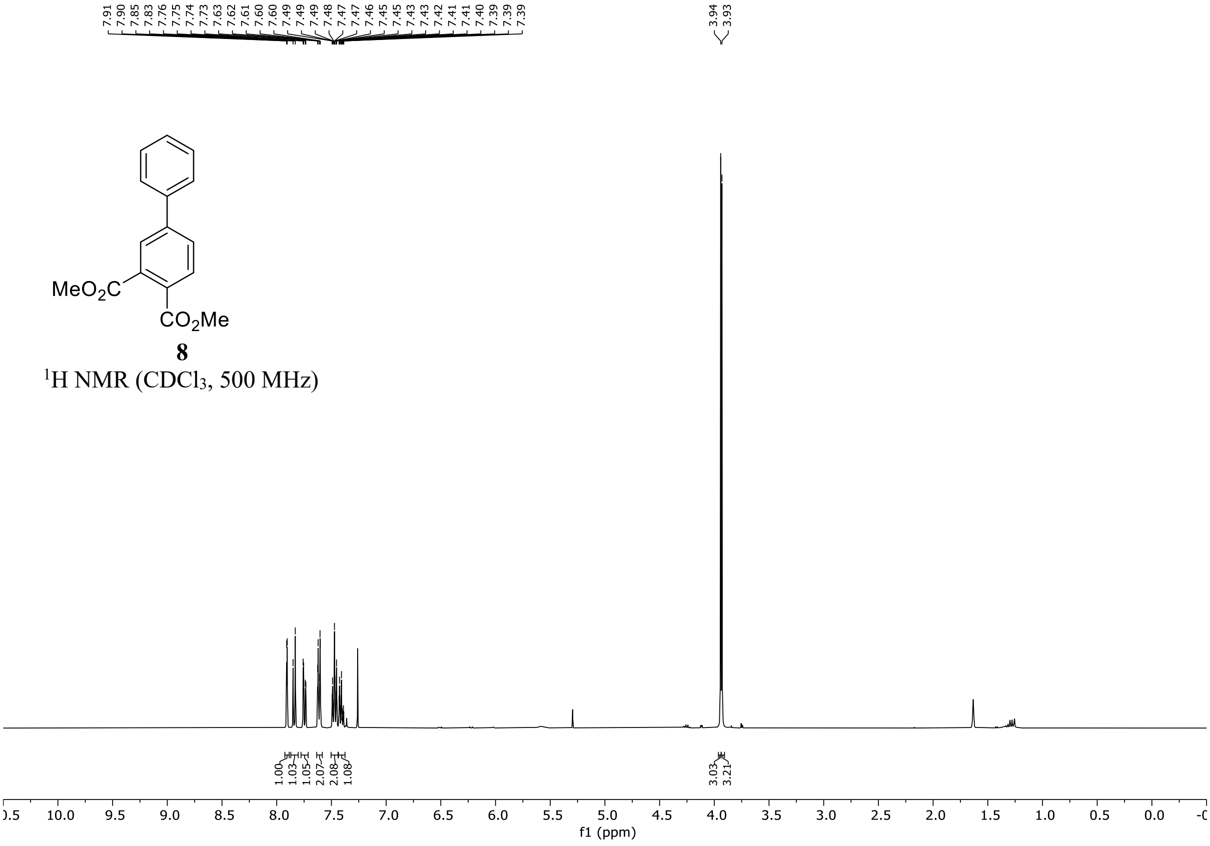

8

^{1}H NMR ($CDCl_3$, 500 MHz)

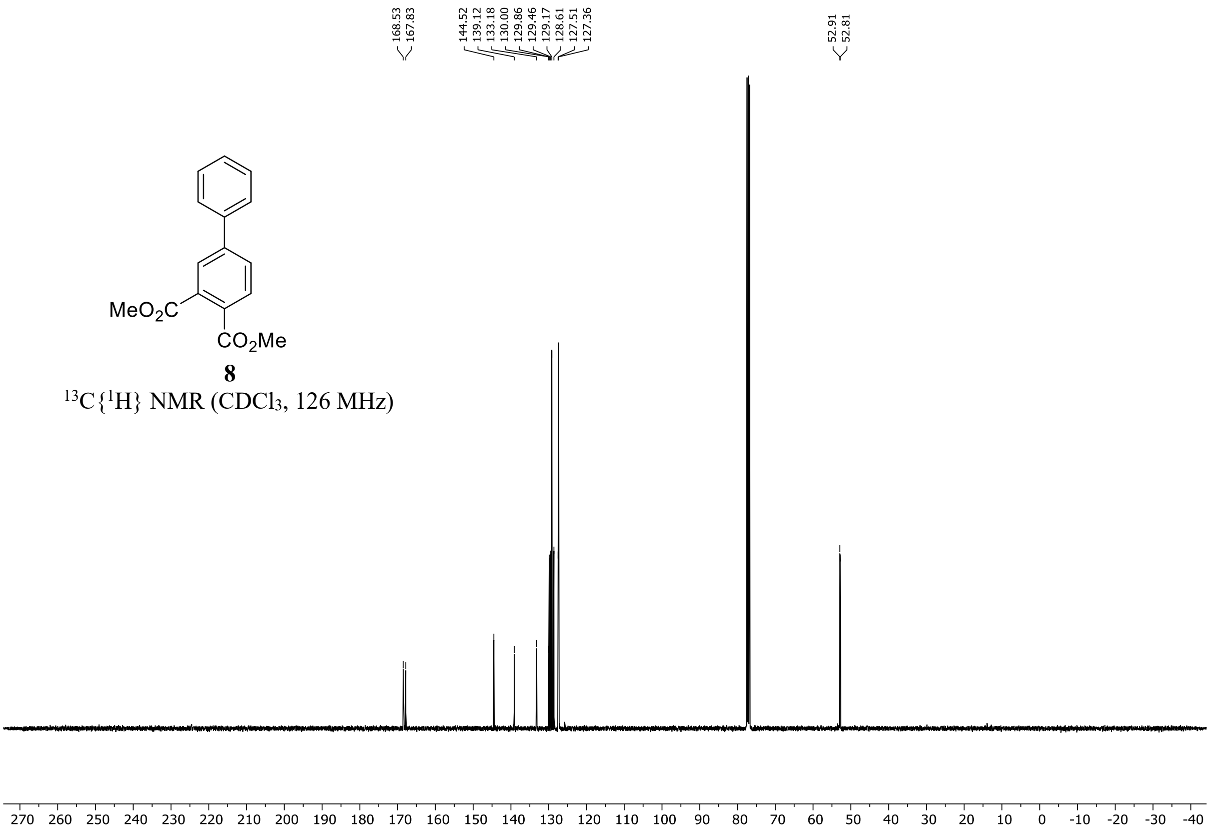

8

$^{13}C\{^{1}H\}$ NMR ($CDCl_3$, 126 MHz)

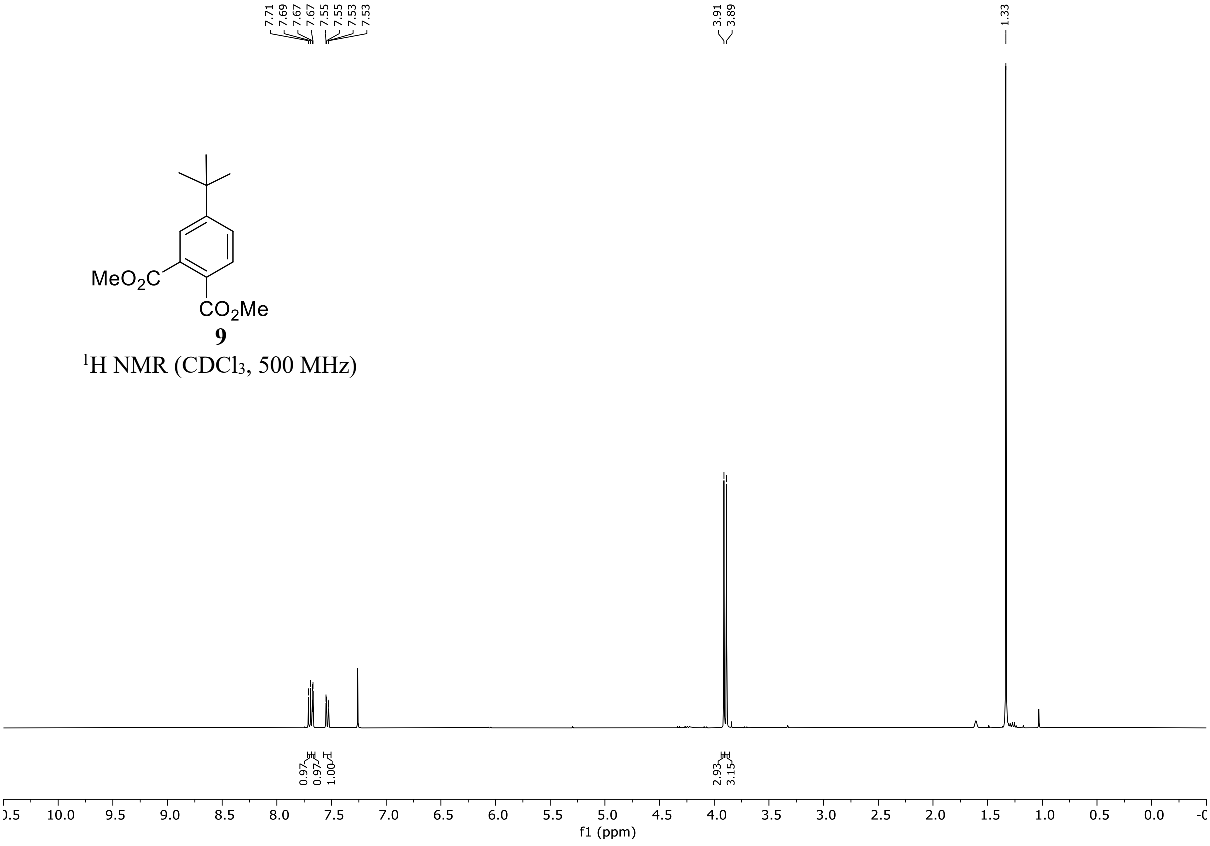

9

^{1}H NMR ($CDCl_3$, 500 MHz)

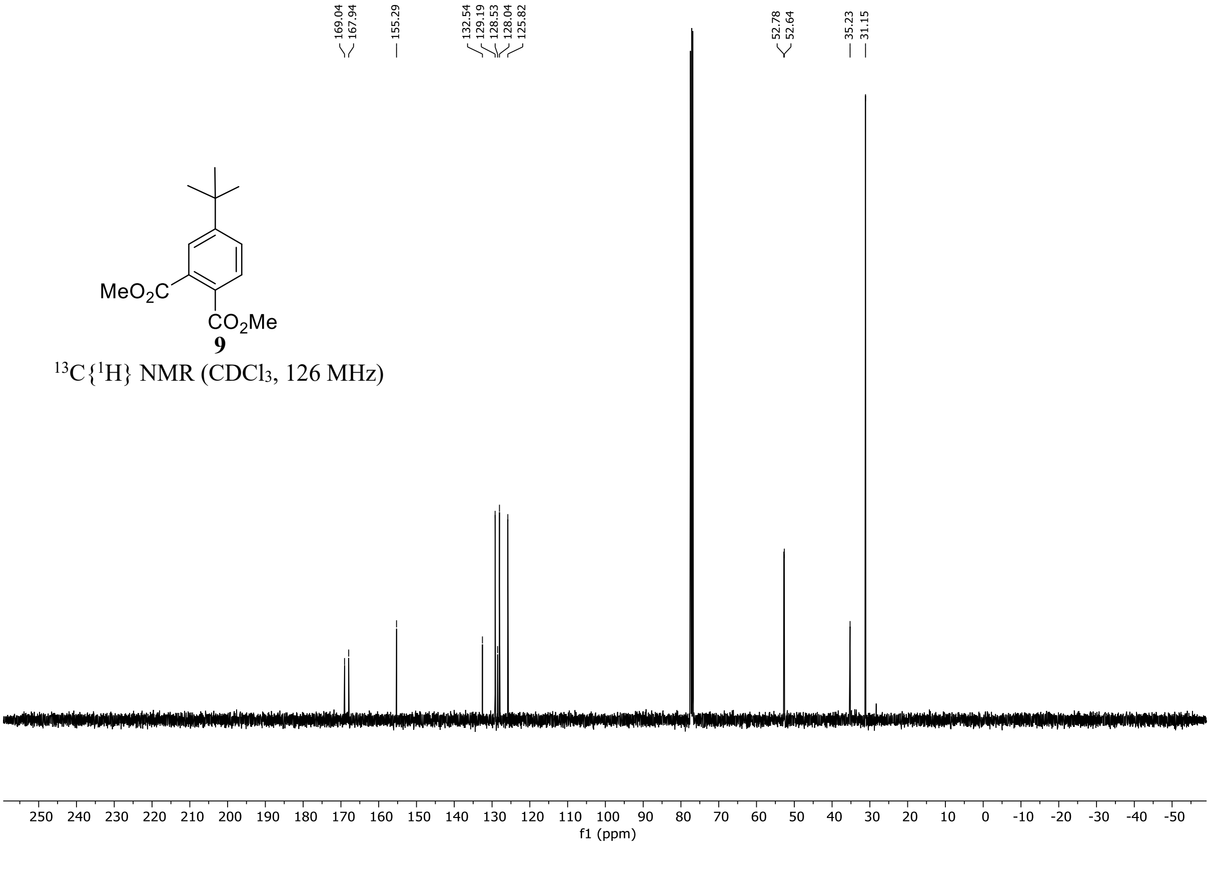

9

$^{13}C\{^{1}H\}$ NMR ($CDCl_3$, 126 MHz)

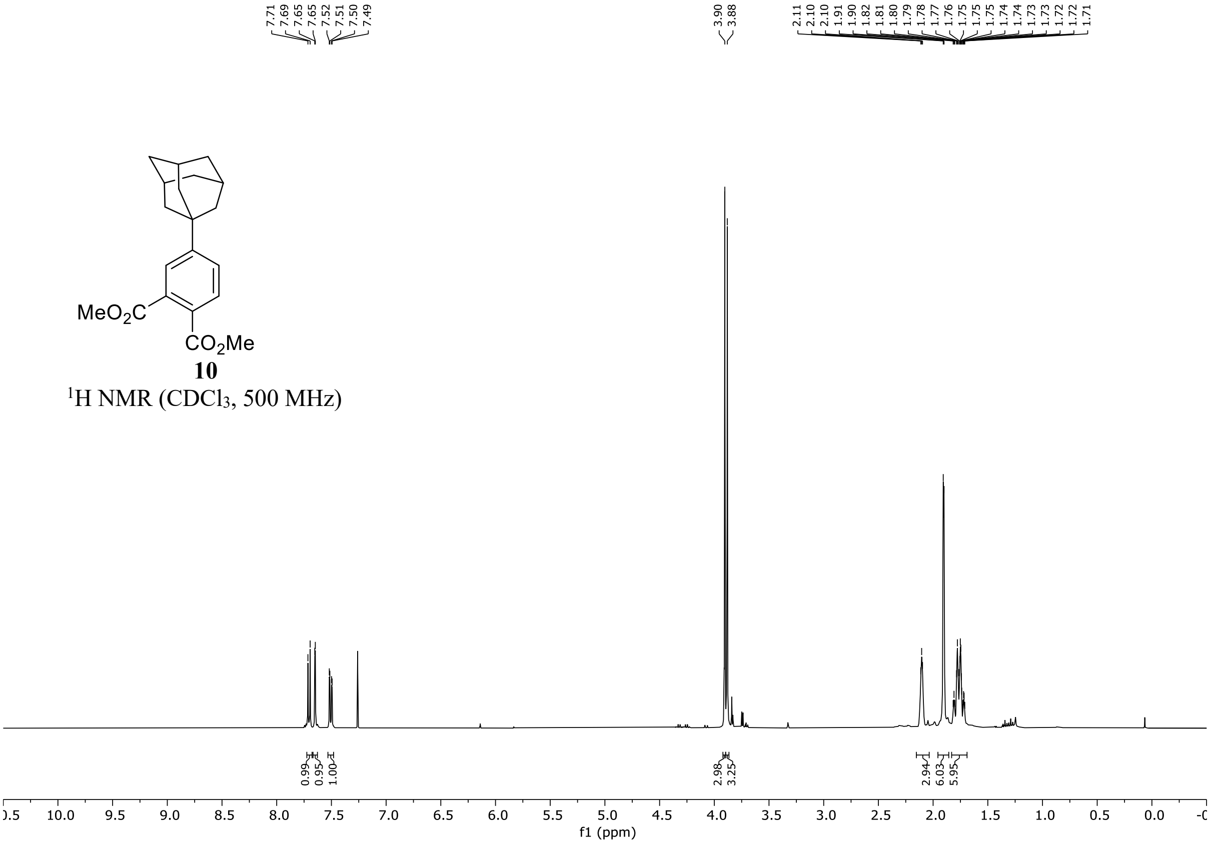
7.71
7.69
7.65
7.65
7.52
7.51
7.50
7.49
3.90
3.88
2.11
2.10
2.10
1.91
1.90
1.82
1.81
1.80
1.79
1.78
1.77
1.76
1.75
1.75
1.75
1.74
1.74
1.73
1.73
1.72
1.72
1.71
MeO2C
CO2Me
10
1H NMR (CDCl3, 500 MHz)
0.99
0.95
1.00
2.98
3.25
2.94
6.03
5.95
10.0 9.5 9.0 8.5 8.0 7.5 7.0 6.5 6.0 5.5 5.0 4.5 4.0 3.5 3.0 2.5 2.0 1.5 1.0 0.5 0.0
f1 (ppm)

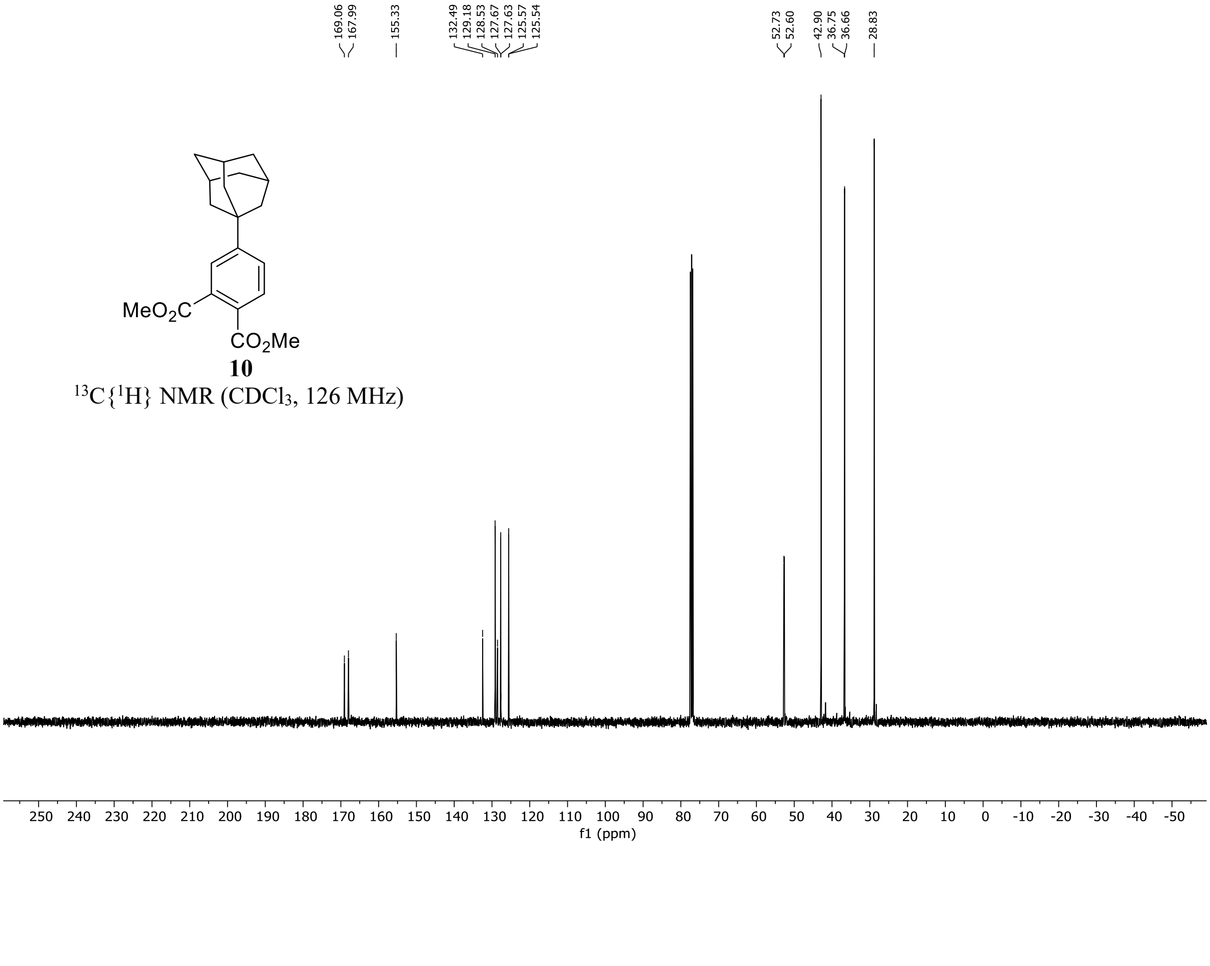
169.06
167.99
155.33
132.49
129.18
128.53
127.67
127.63
125.57
125.54
52.73
52.60
42.90
36.75
36.66
28.83
MeO2C
CO2Me
10
13C{1H} NMR (CDCl3, 126 MHz)
250 240 230 220 210 200 190 180 170 160 150 140 130 120 110 100 90 80 70 60 50 40 30 20 10 0 -10 -20 -30 -40 -50
f1 (ppm)

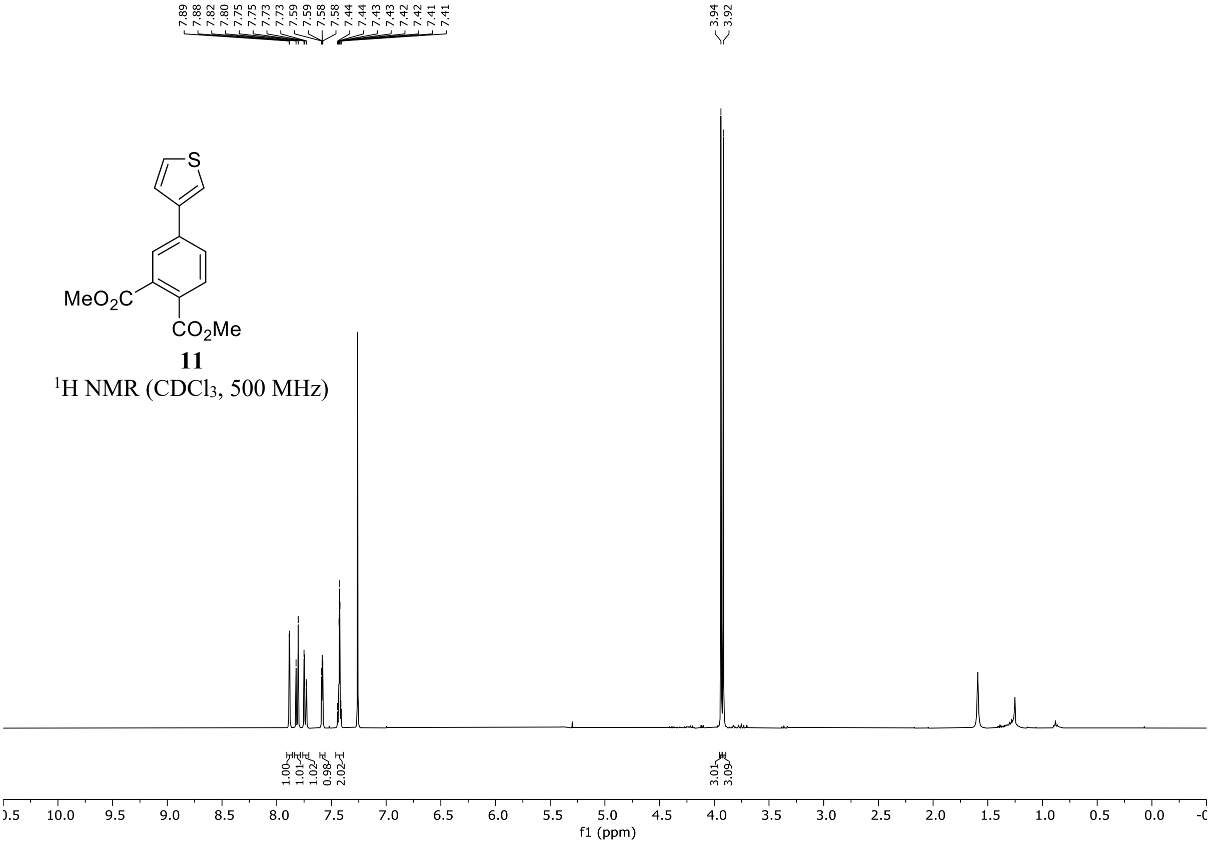

11

^{1}H NMR ($CDCl_3$, 500 MHz)

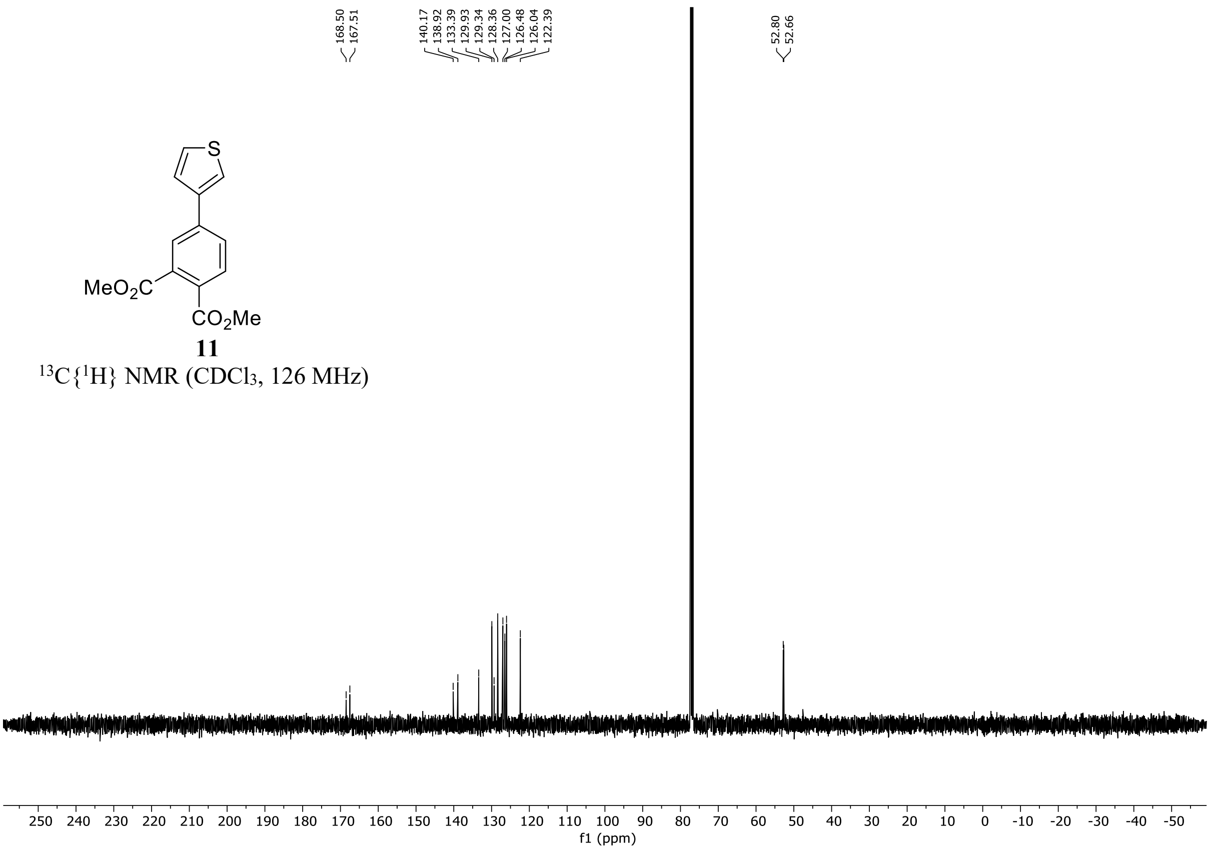

11

$^{13}C\{^{1}H\}$ NMR ($CDCl_3$, 126 MHz)

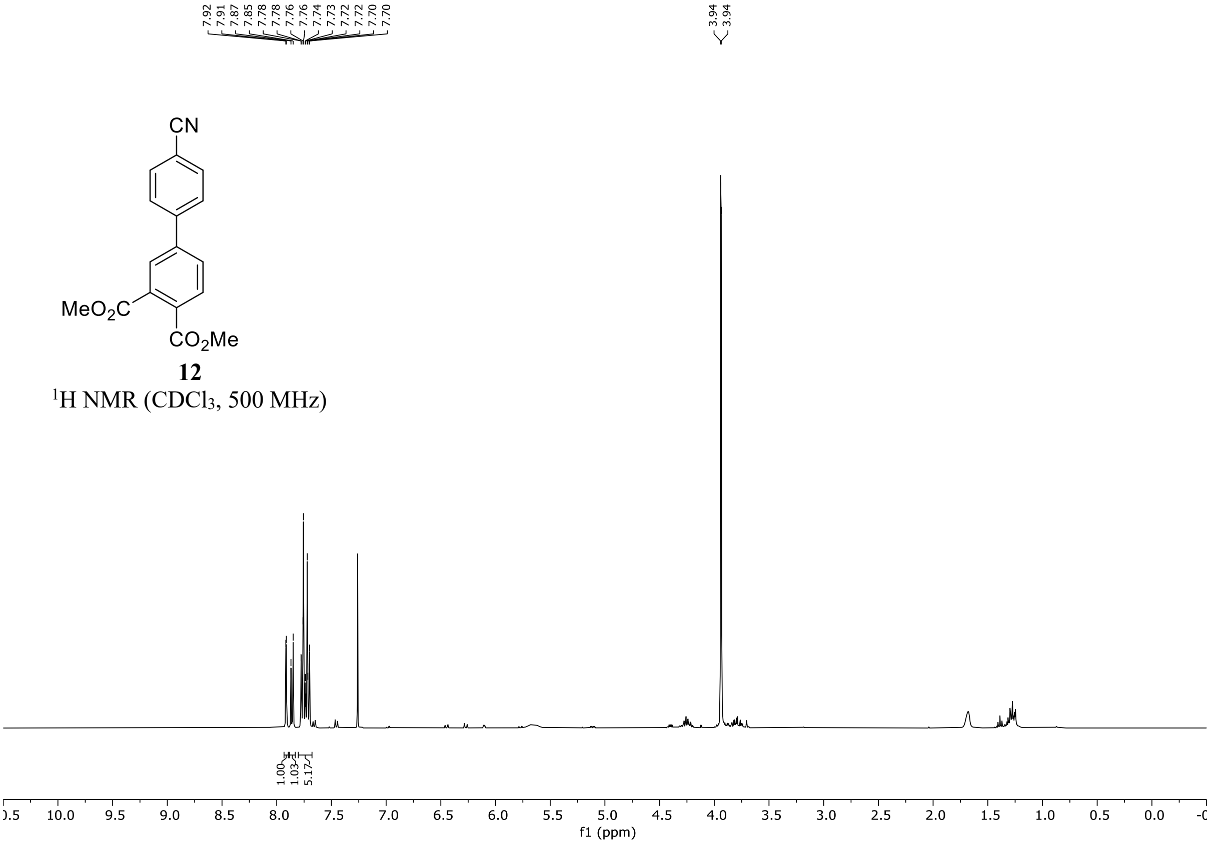

12

^{1}H NMR (CDCl3, 500 MHz)

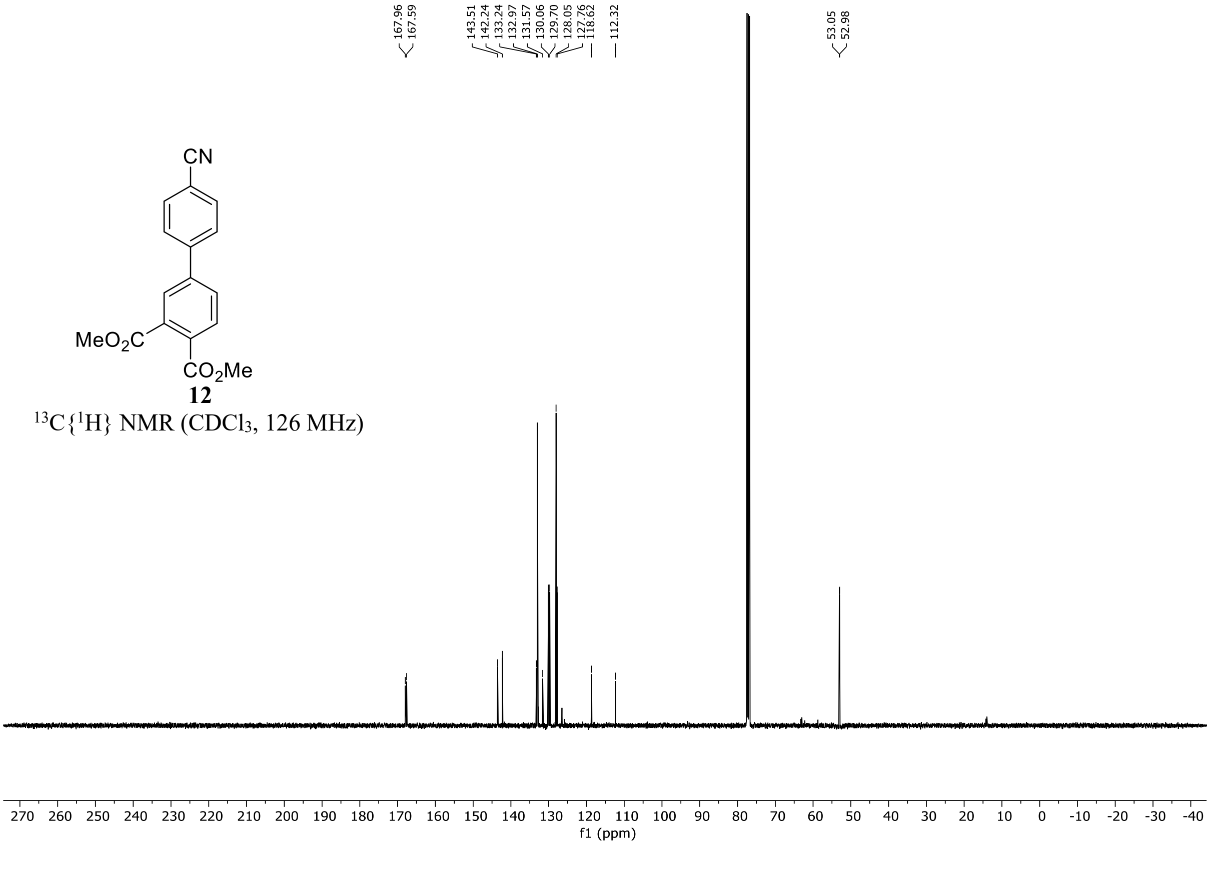

12

^{13}C{^{1}H} NMR (CDCl3, 126 MHz)

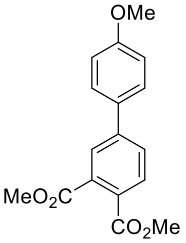

13

^{1}H NMR ($CDCl_3$, 500 MHz)

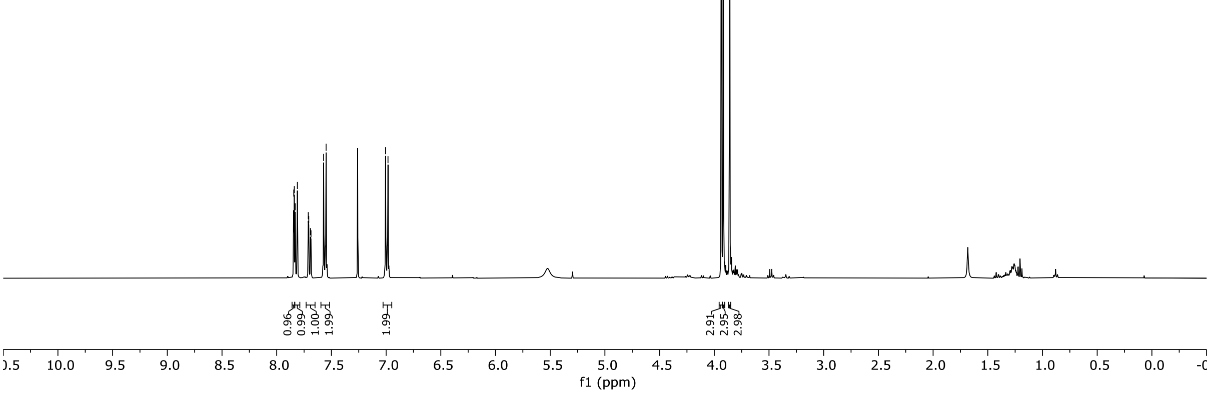

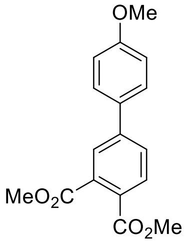

13

^{13}C{^{1}H} NMR ($CDCl_3$, 126 MHz)

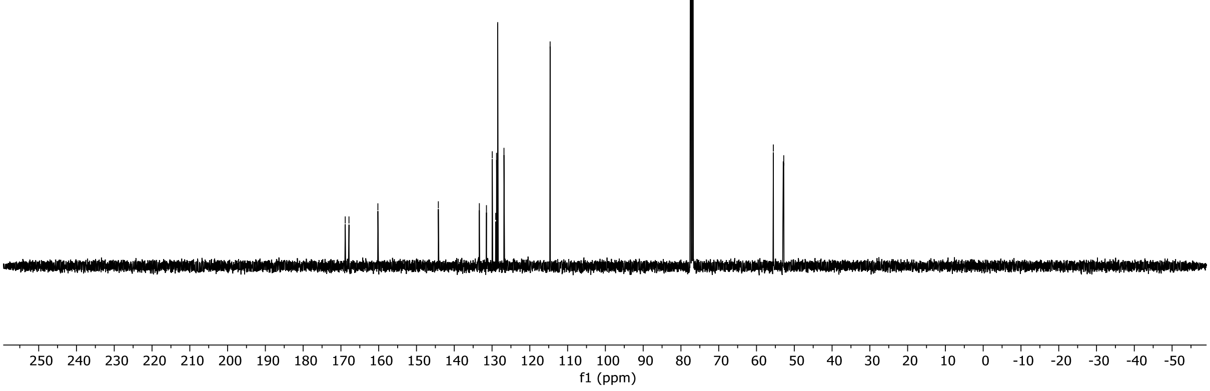

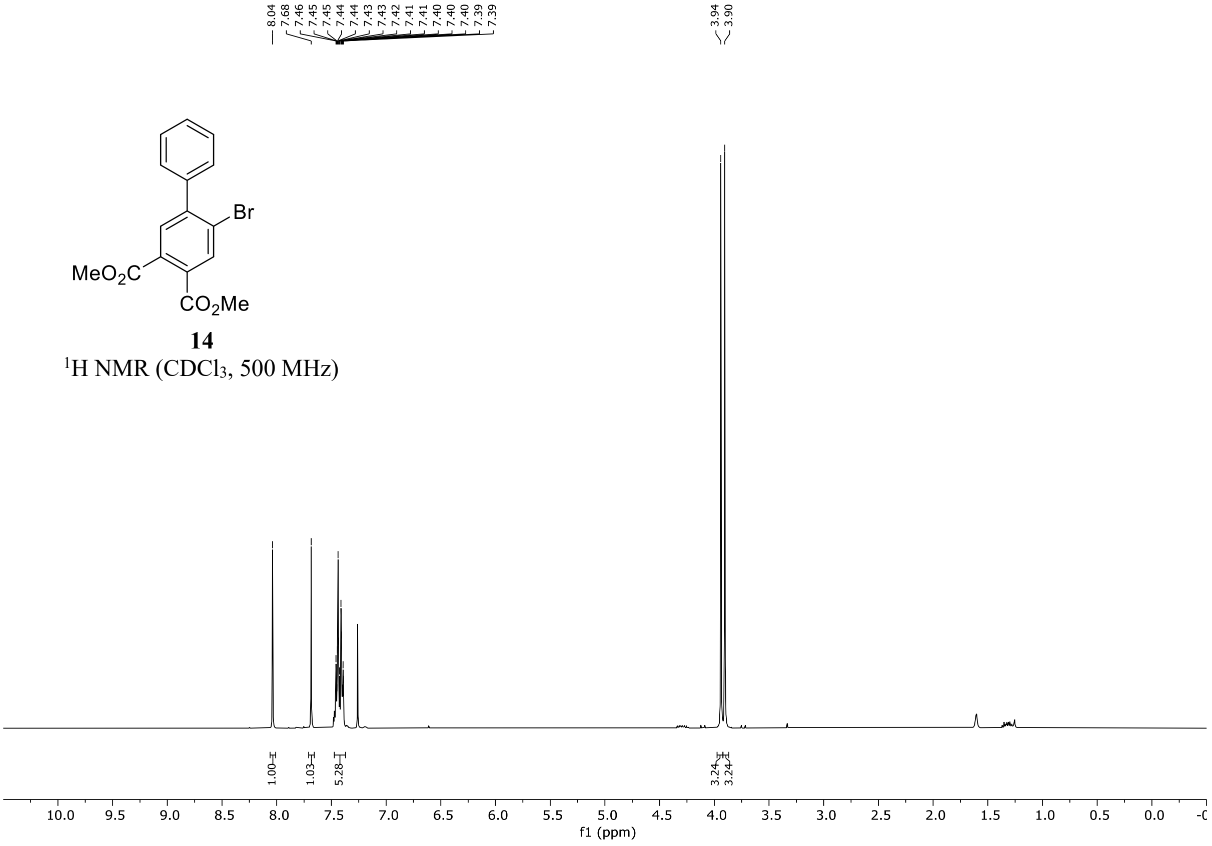
8.04
7.68
7.46
7.45
7.45
7.44
7.44
7.43
7.43
7.42
7.41
7.41
7.40
7.40
7.40
7.39
7.39
3.94
3.90
Br
MeO2C
CO2Me
14
1H NMR (CDCl3, 500 MHz)
1.00
1.03
5.28
3.24
3.24
f1 (ppm)

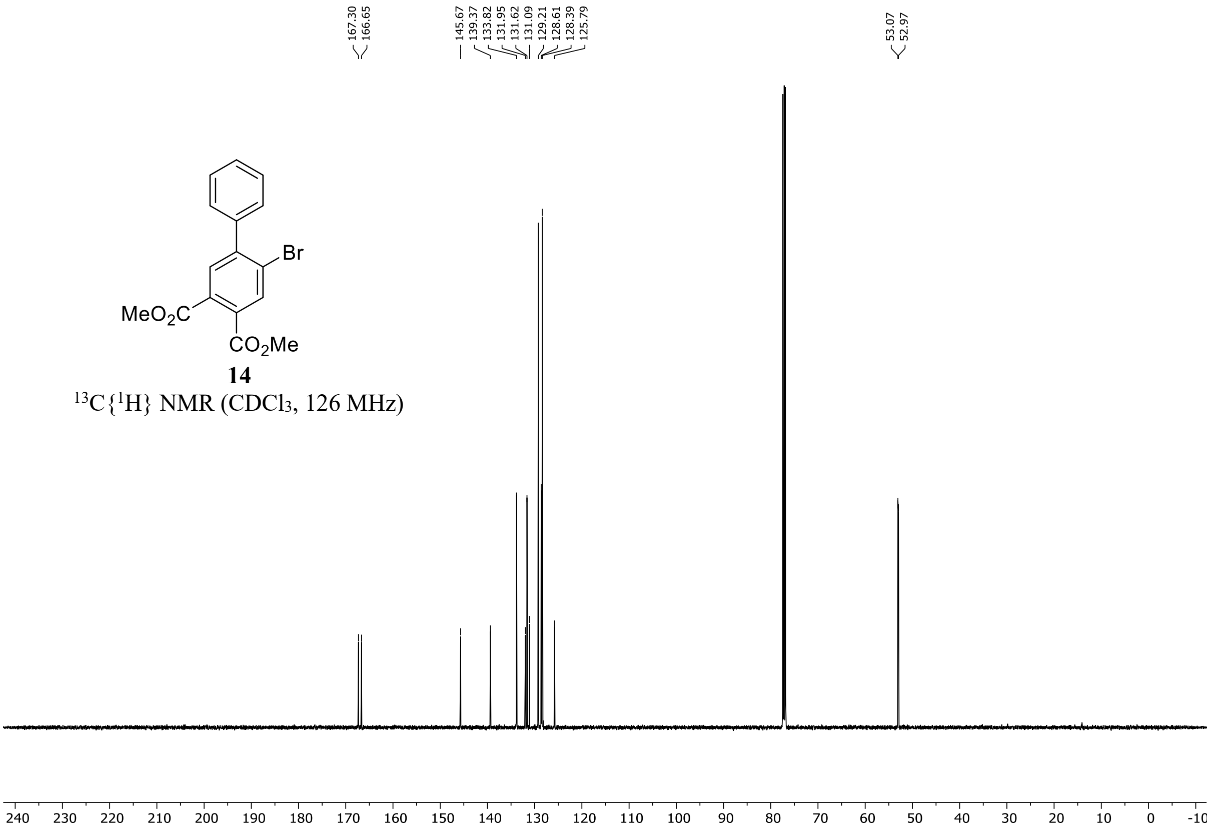
167.30
166.65
145.67
139.37
133.82
131.95
131.62
131.09
129.21
128.61
128.39
125.79
53.07
52.97
Br
MeO2C
CO2Me
14
13C{1H} NMR (CDCl3, 126 MHz)
f1 (ppm)

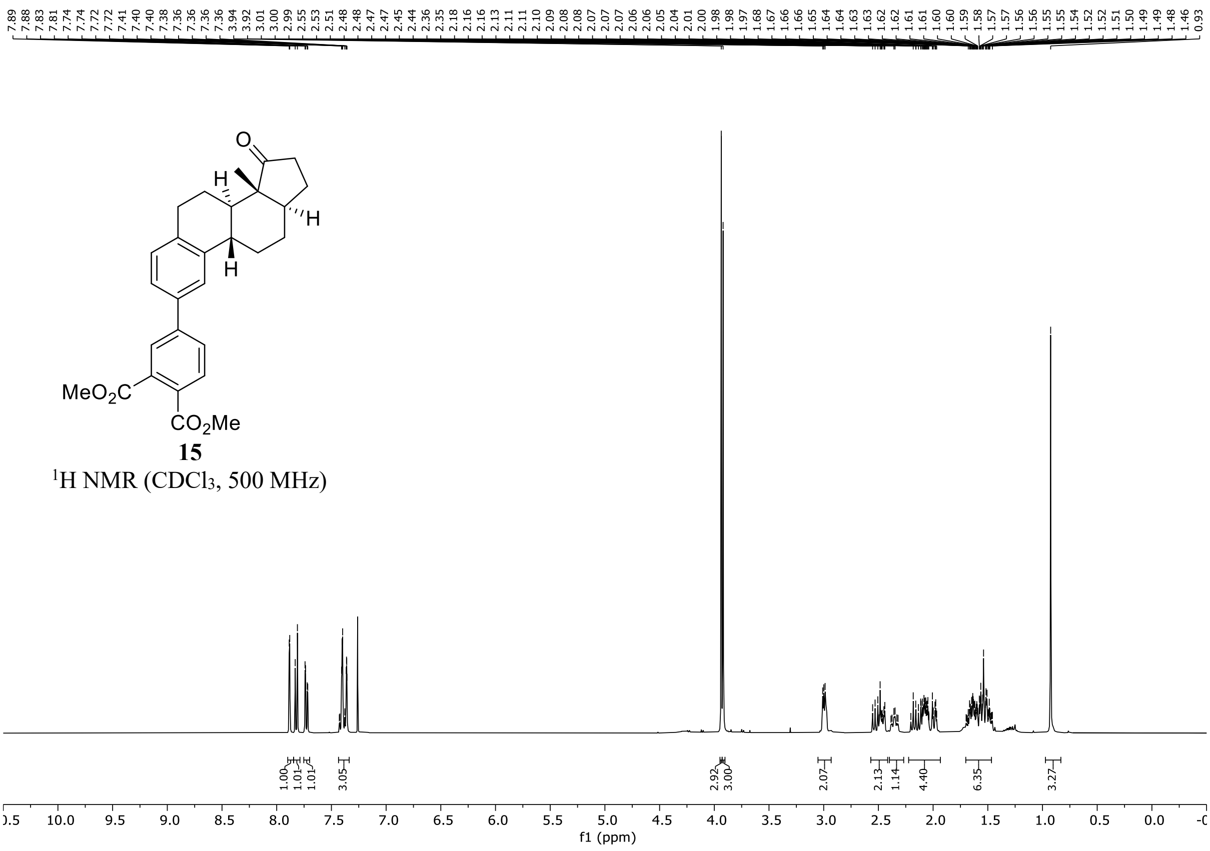

15

^{1}H NMR ($CDCl_3$, 500 MHz)

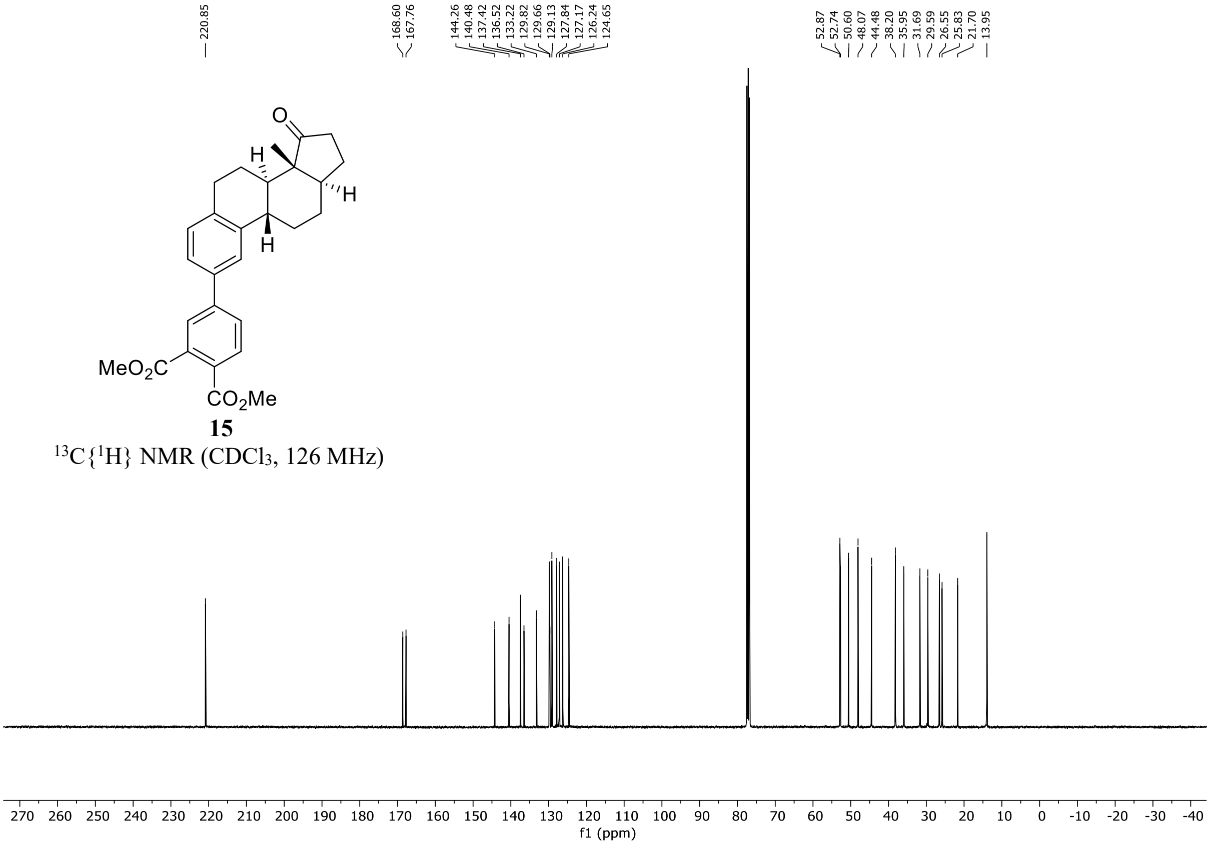

15

$^{13}C\{^{1}H\}$ NMR ($CDCl_3$, 126 MHz)

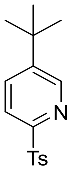

20

^{1}H NMR ($CDCl_3$, 500 MHz)

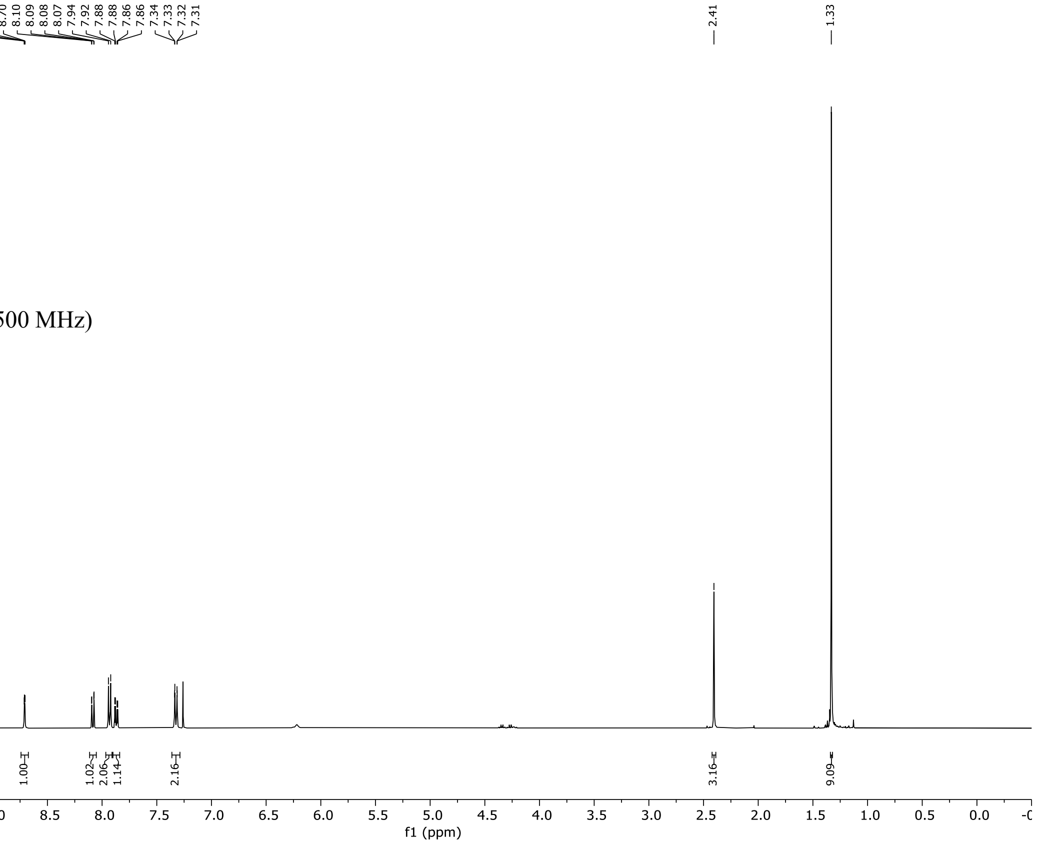

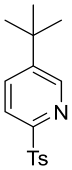

20

^{13}C{^{1}H} NMR ($CDCl_3$, 126 MHz)

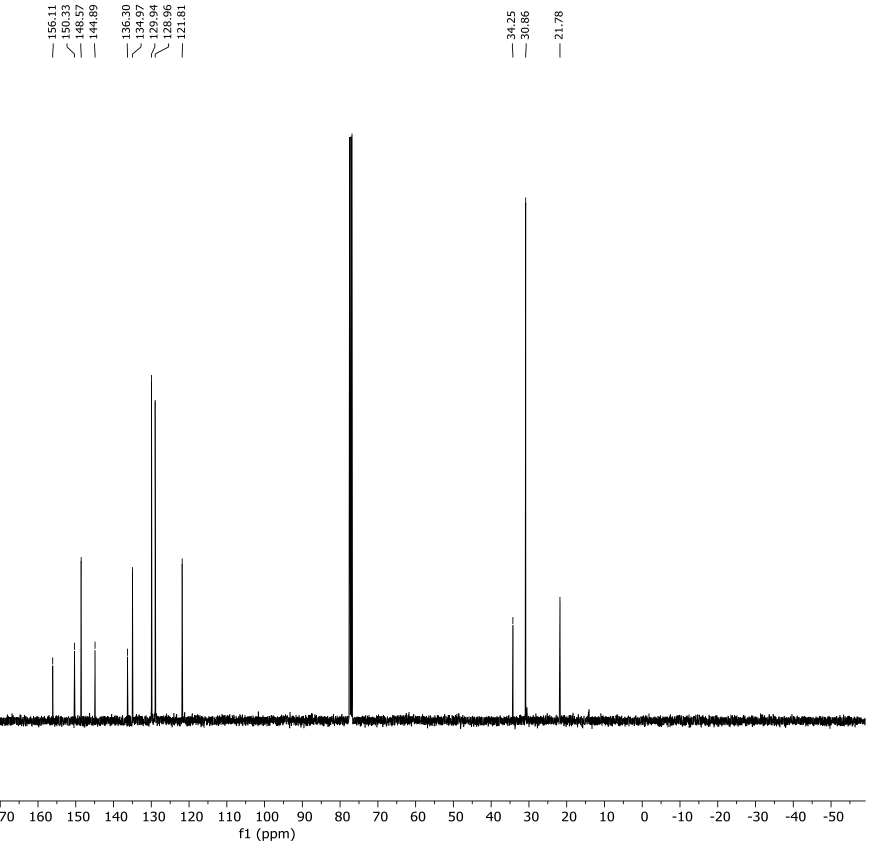

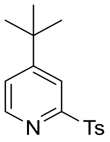

21

^{1}H NMR ($CDCl_3$, 500 MHz)

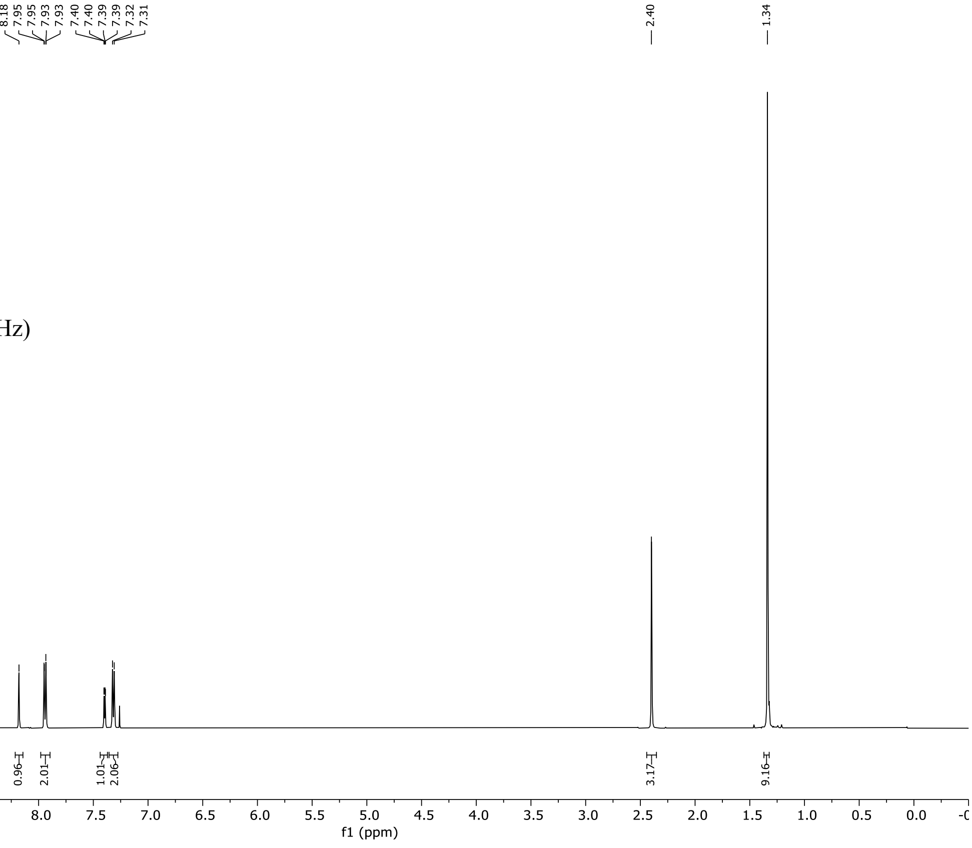

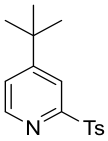

21

^{13}C{^{1}H} NMR ($CDCl_3$, 126 MHz)

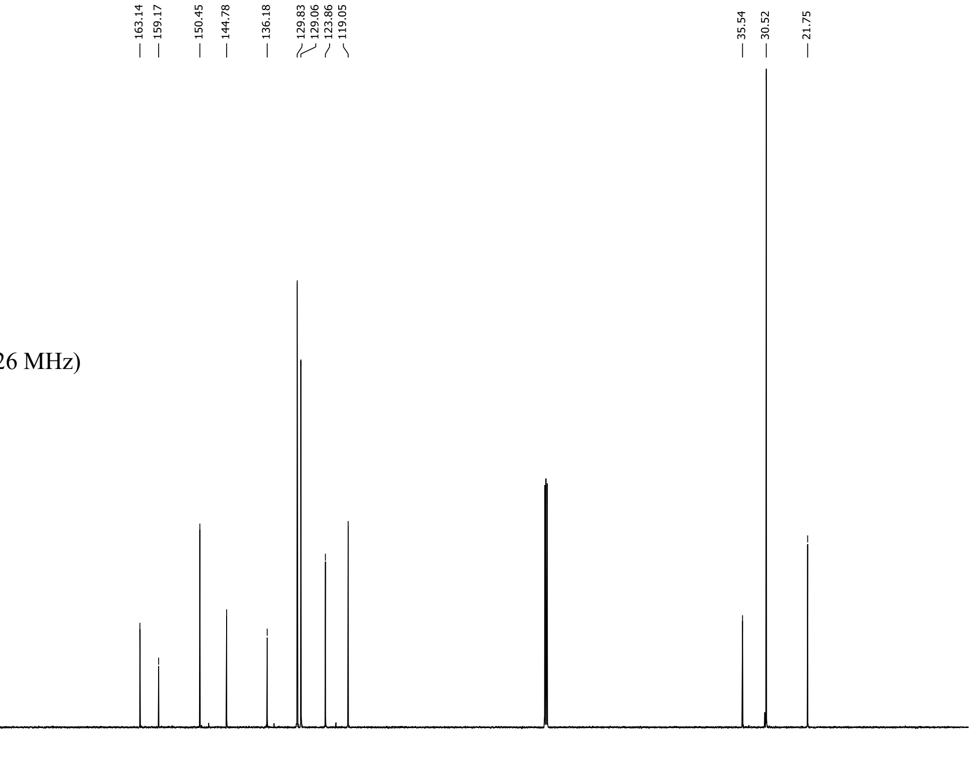

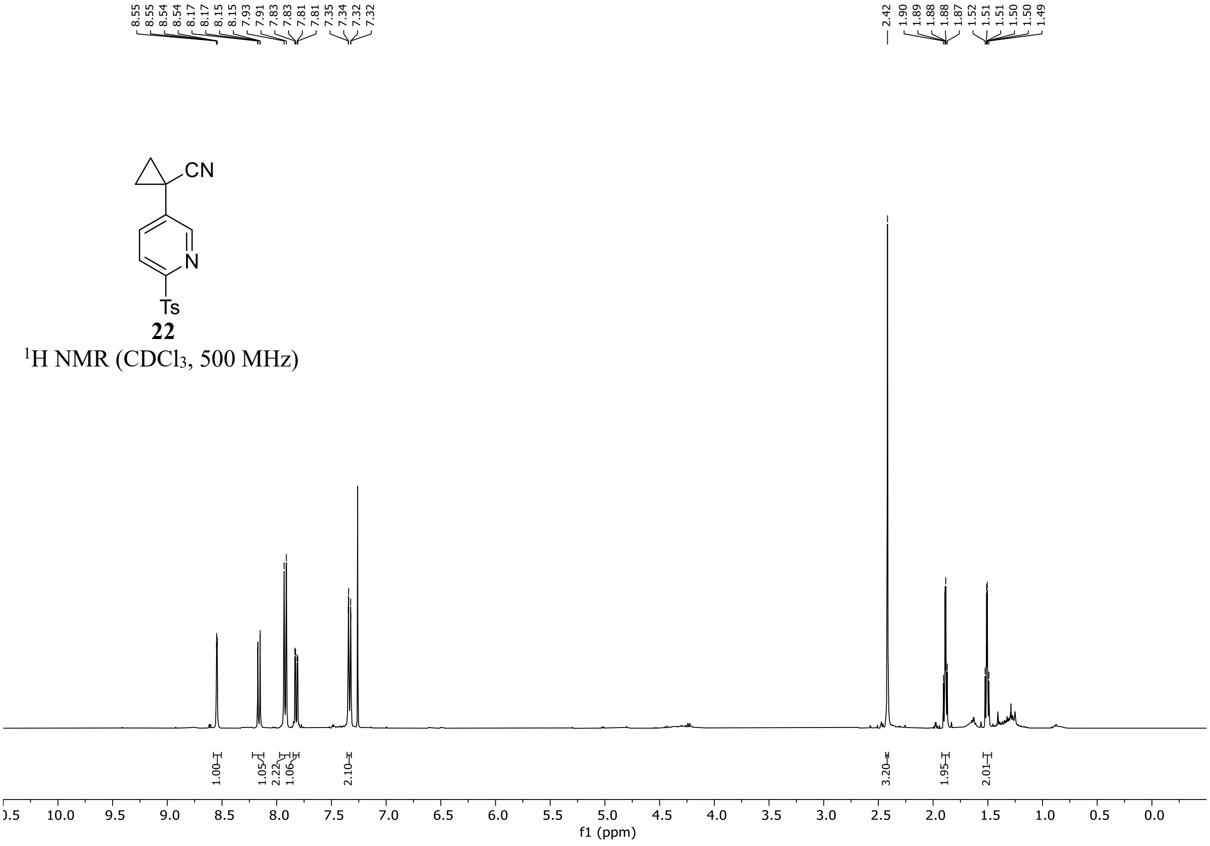
8.55 8.55 8.54 8.54 8.17 8.17 8.15 8.15 7.93 7.91 7.83 7.83 7.81 7.81 7.35 7.34 7.32 7.32
2.42 1.90 1.89 1.88 1.88 1.87 1.52 1.51 1.51 1.50 1.50 1.49
CN
N
Ts
22
1H NMR (CDCl3, 500 MHz)
1.00 1.05 2.22 1.06 2.10 3.20 1.95 2.01
f1 (ppm)

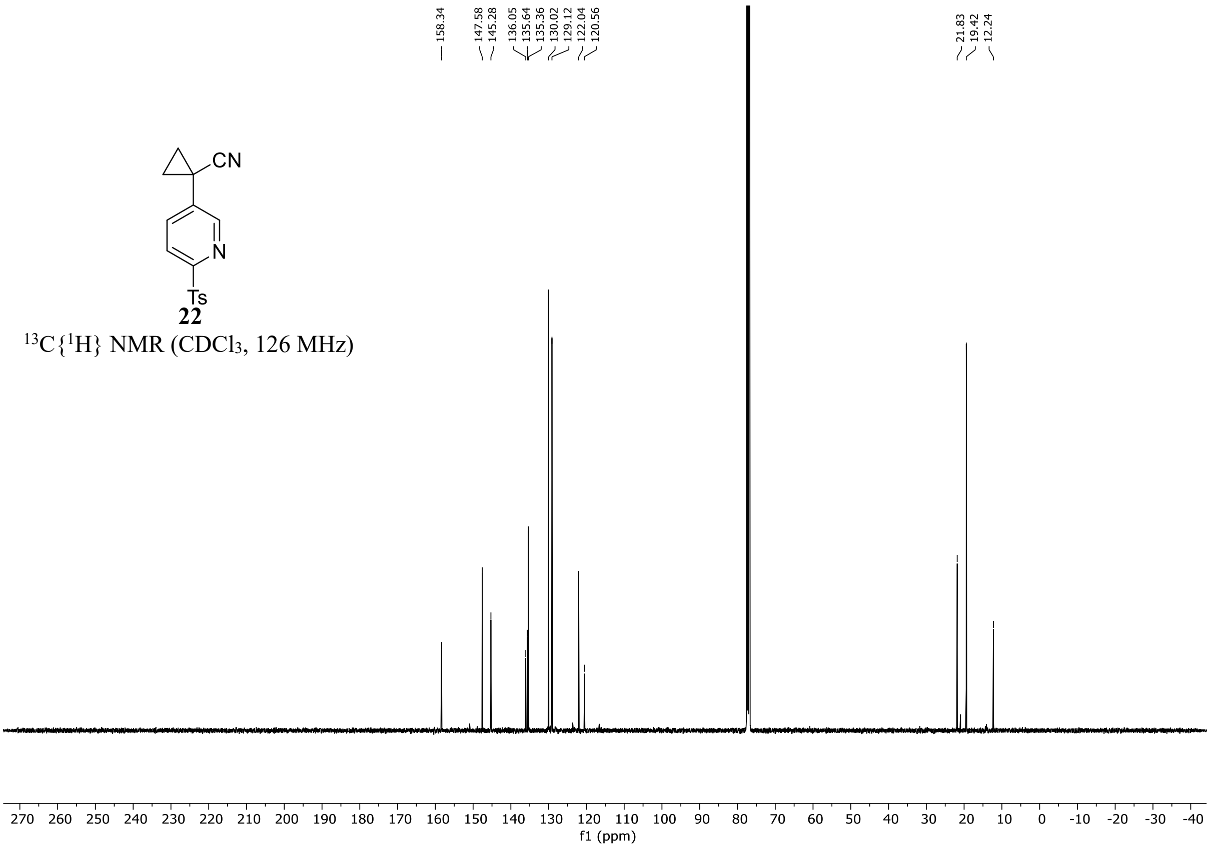
158.34 147.58 145.28 136.05 135.64 135.36 130.02 129.12 122.04 120.56
21.83 19.42 12.24
CN
N
Ts
22
13C{1H} NMR (CDCl3, 126 MHz)
f1 (ppm)

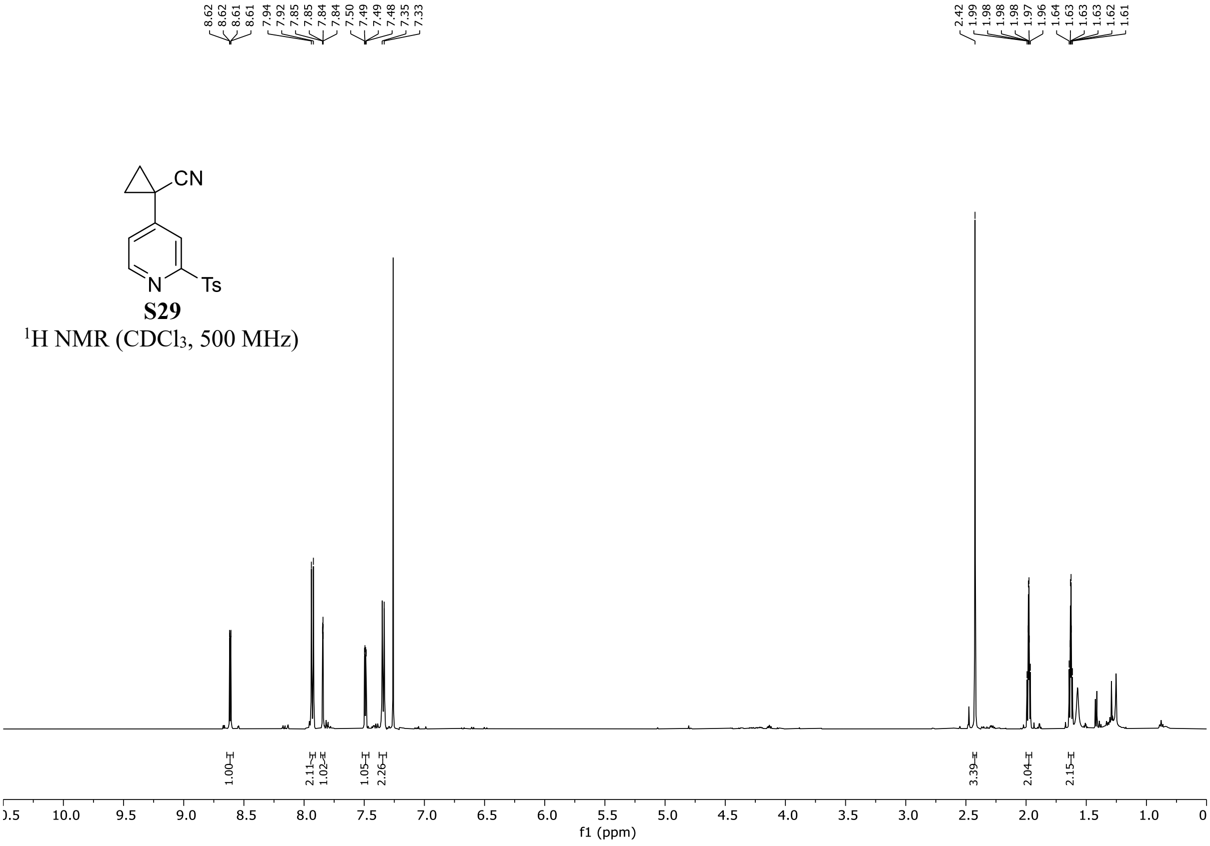
CN
N
Ts
S29
¹H NMR (CDCl₃, 500 MHz)
8.62 8.62 8.61 8.61 7.94 7.92 7.85 7.85 7.84 7.84 7.50 7.49 7.49 7.48 7.35 7.33
2.42 1.99 1.98 1.98 1.98 1.97 1.96 1.64 1.63 1.63 1.63 1.62 1.61
1.00 2.11 1.02 1.05 2.26 3.39 2.04 2.15
f1 (ppm)

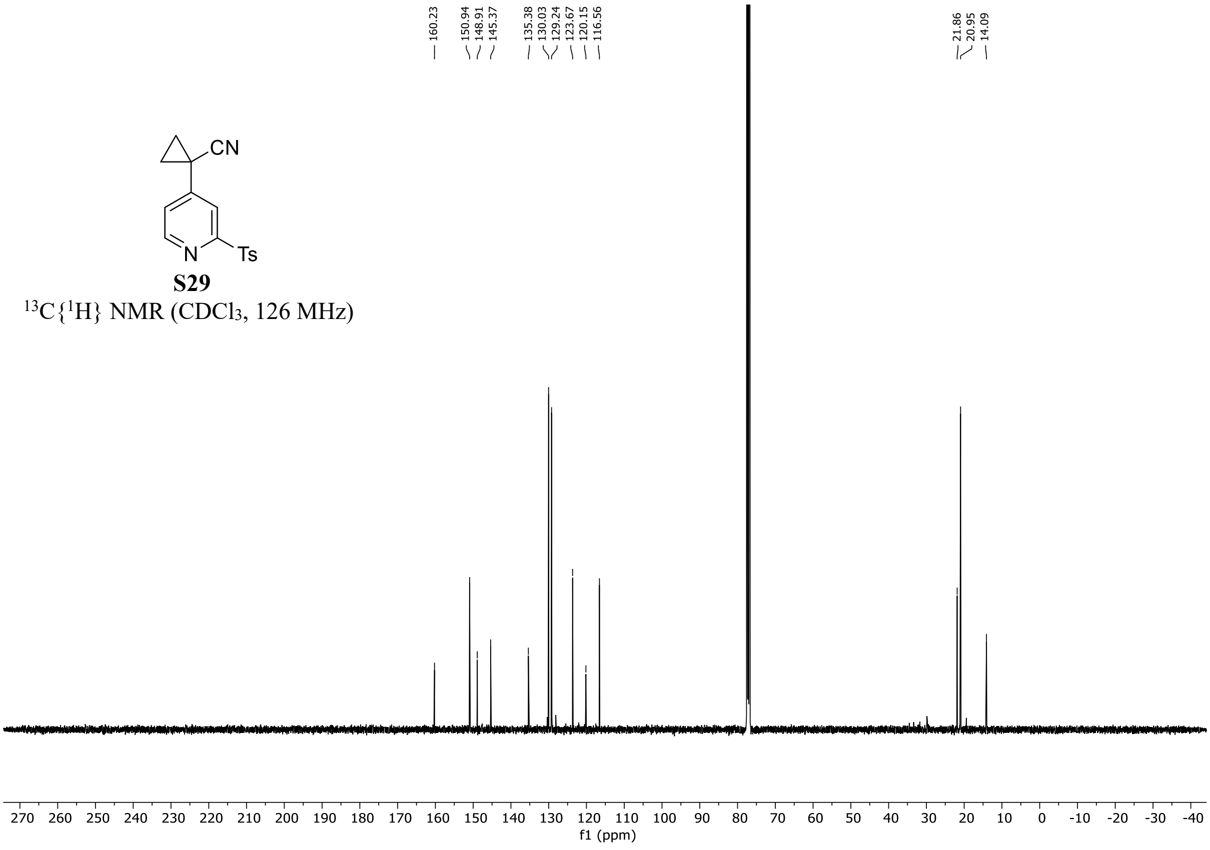
CN
N
Ts
S29
¹³C{¹H} NMR (CDCl₃, 126 MHz)
160.23 150.94 148.91 145.37 135.38 130.03 129.24 123.67 120.15 116.56
21.86 20.95 14.09
f1 (ppm)

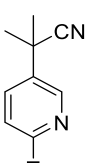

23

^{1}H NMR (CDCl$_3$, 500 MHz)

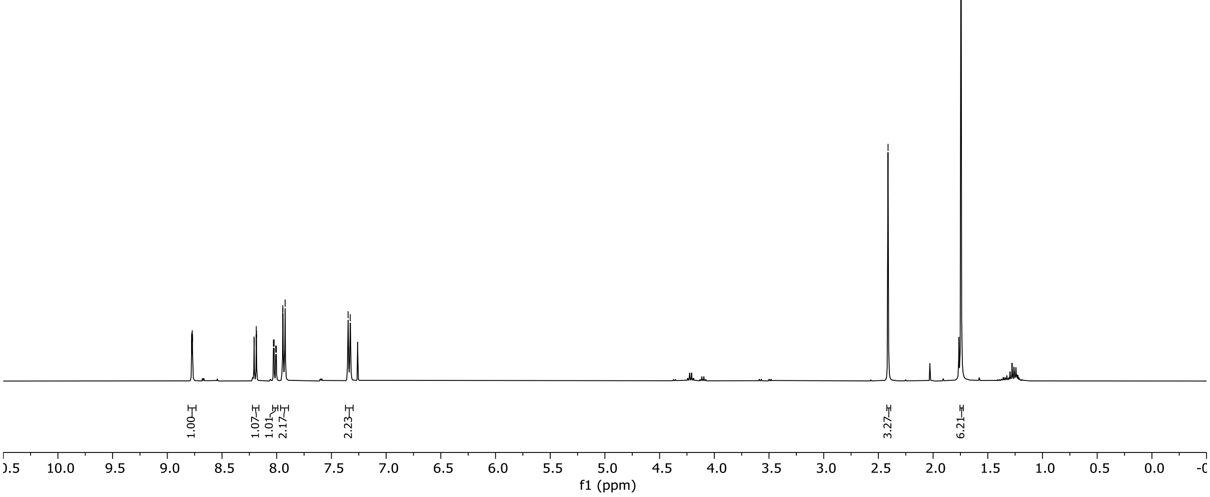

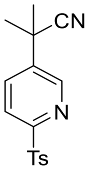

23

^{13}C{^{1}H} NMR (CDCl$_3$, 126 MHz)

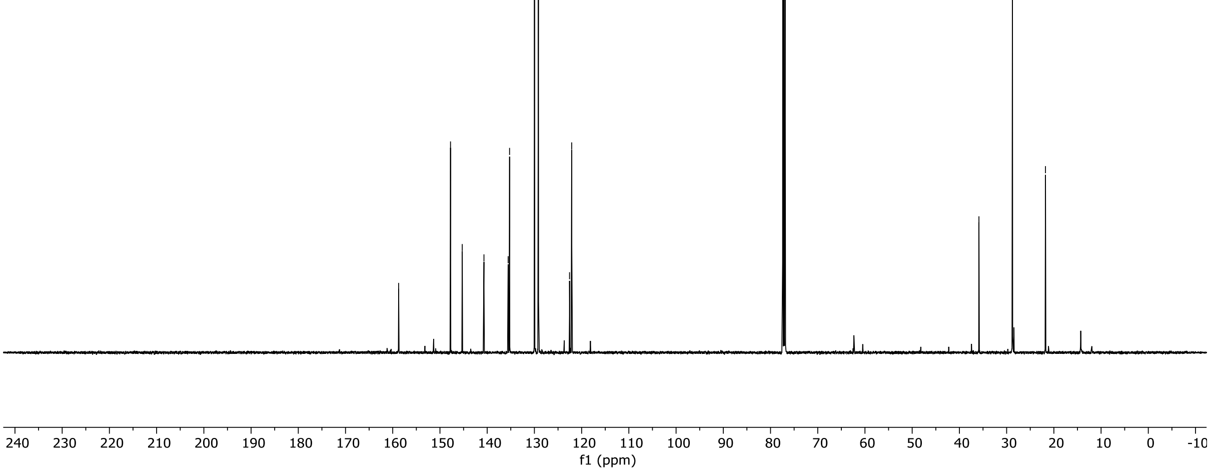

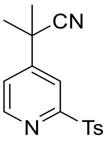

S30

^{1}H NMR (CDCl$_3$, 500 MHz)

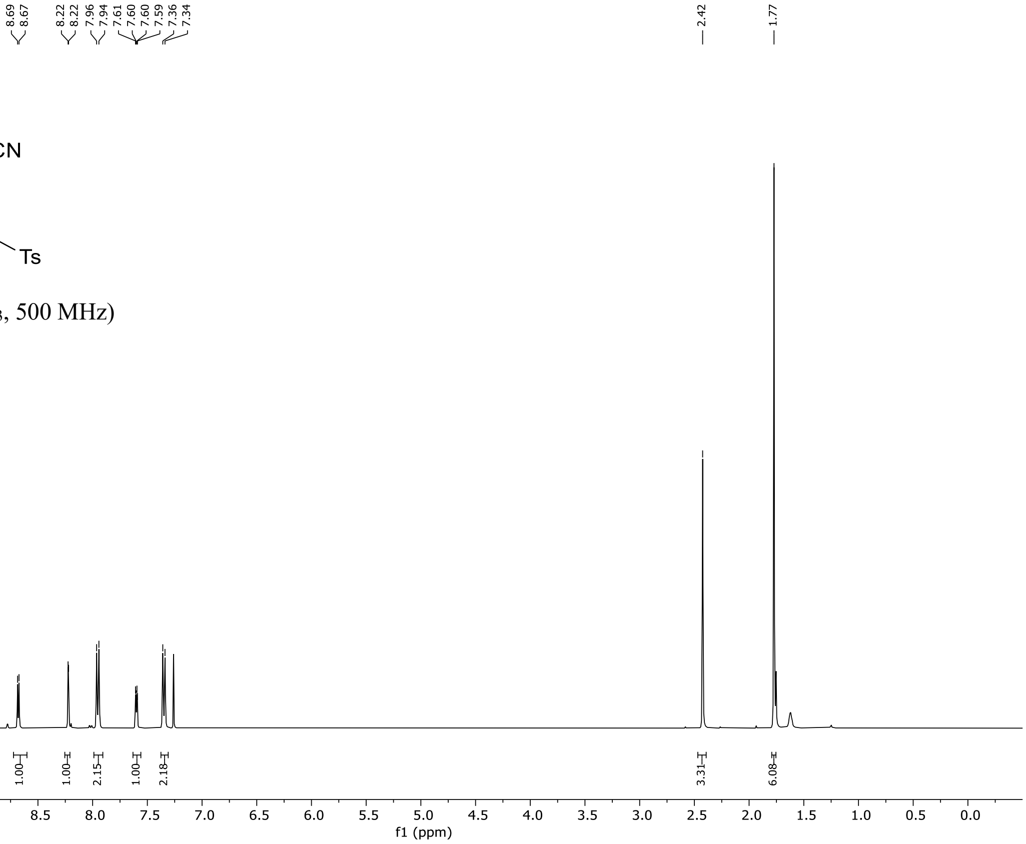

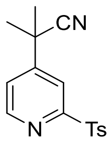

S30

^{13}C{^{1}H} NMR (CDCl$_3$, 126 MHz)

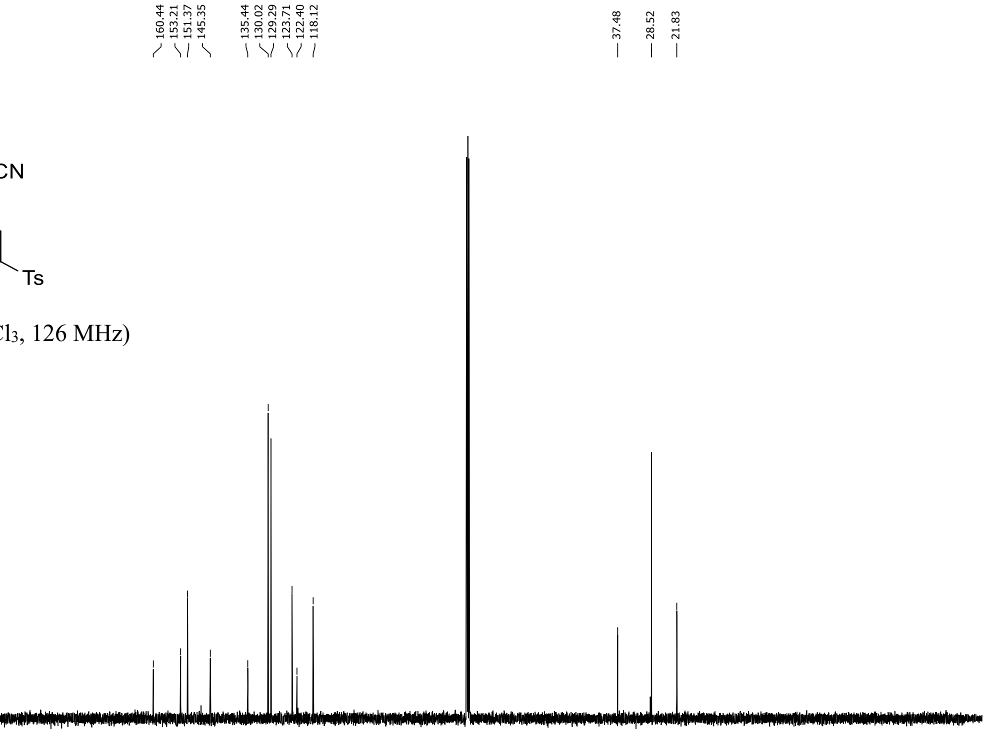

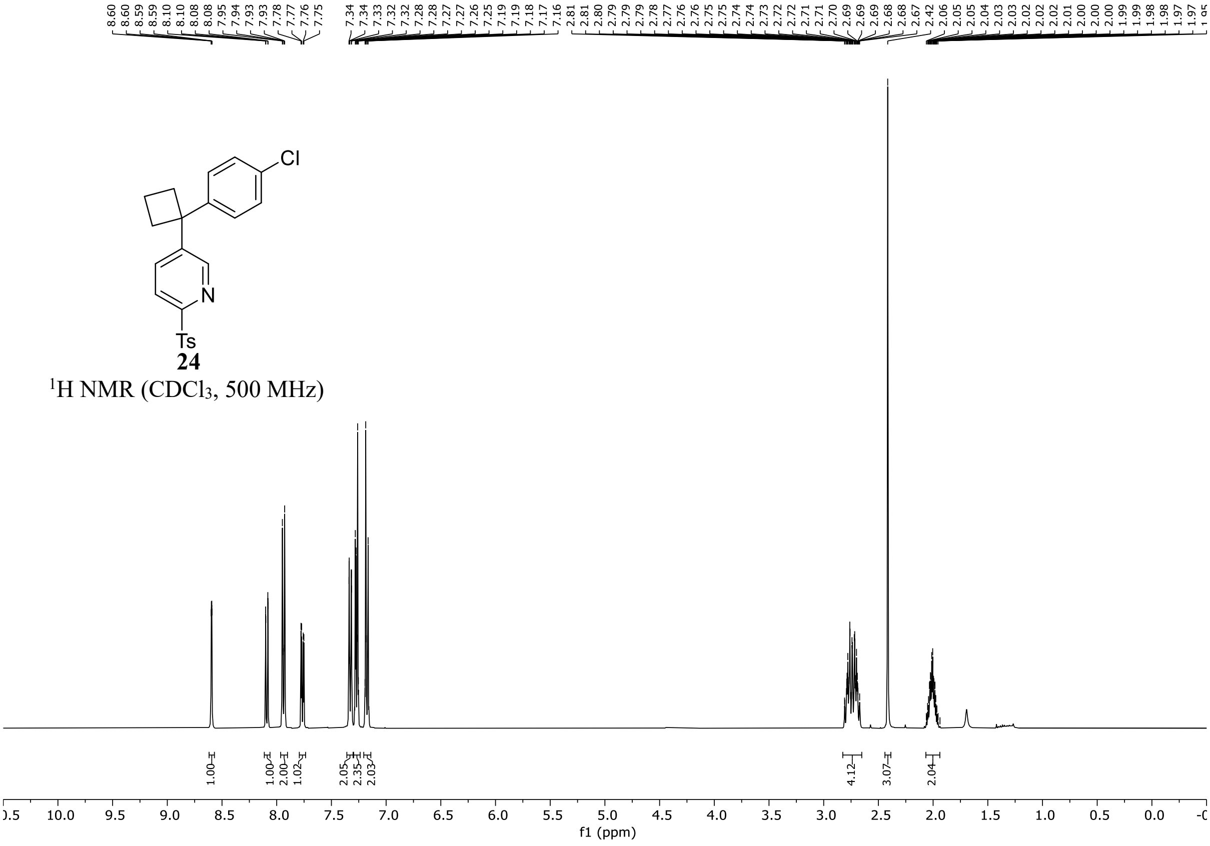

24

^{1}H NMR ($CDCl_3$, 500 MHz)

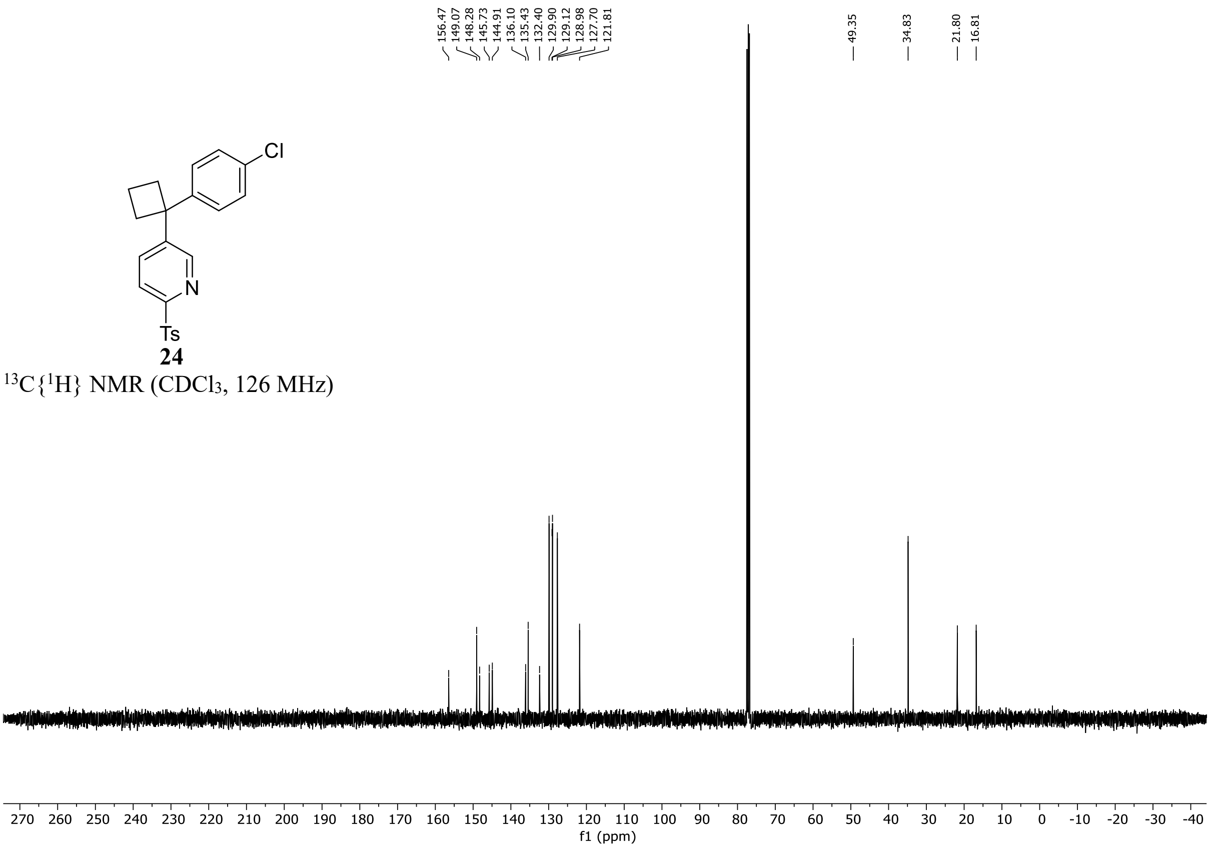

24

$^{13}C\{^1H\}$ NMR ($CDCl_3$, 126 MHz)

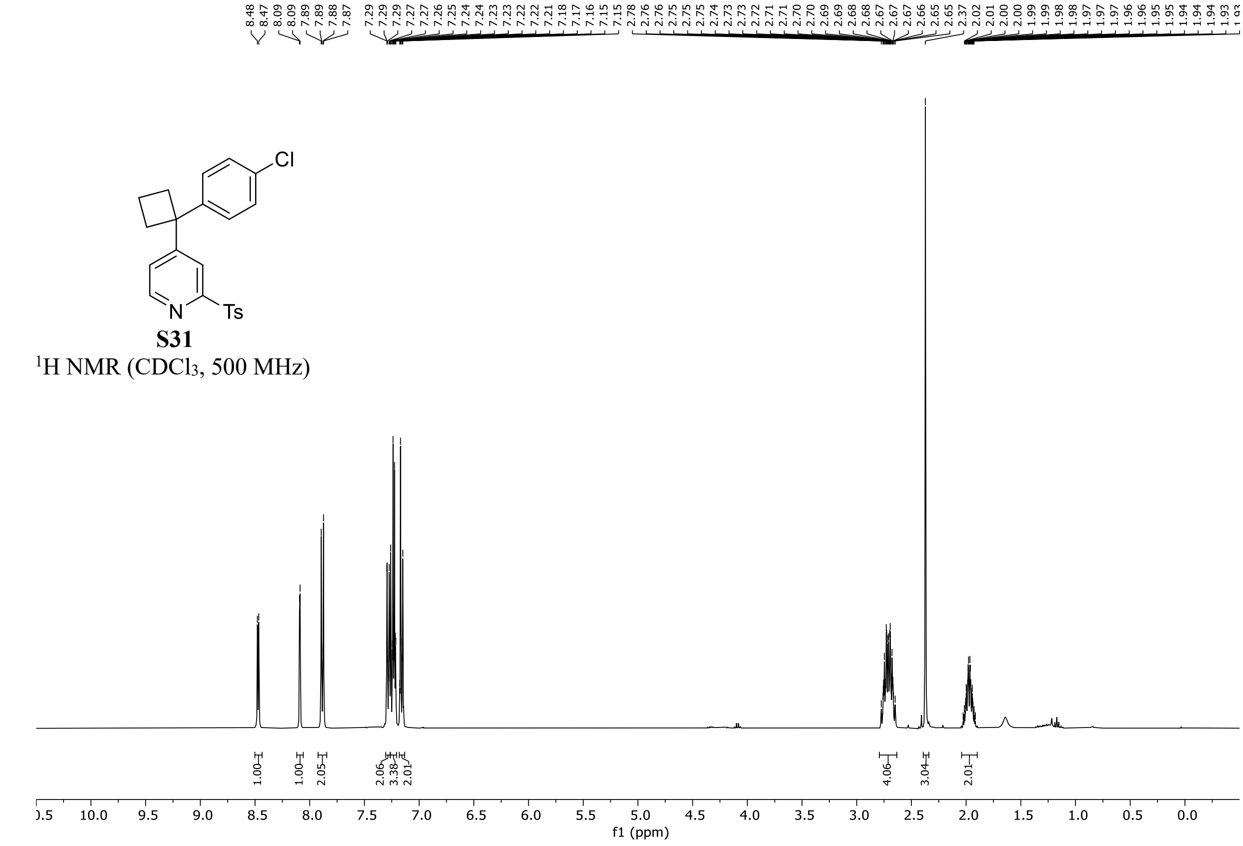

Cl
N
Ts
S31
1H NMR (CDCl3, 500 MHz)
f1 (ppm)

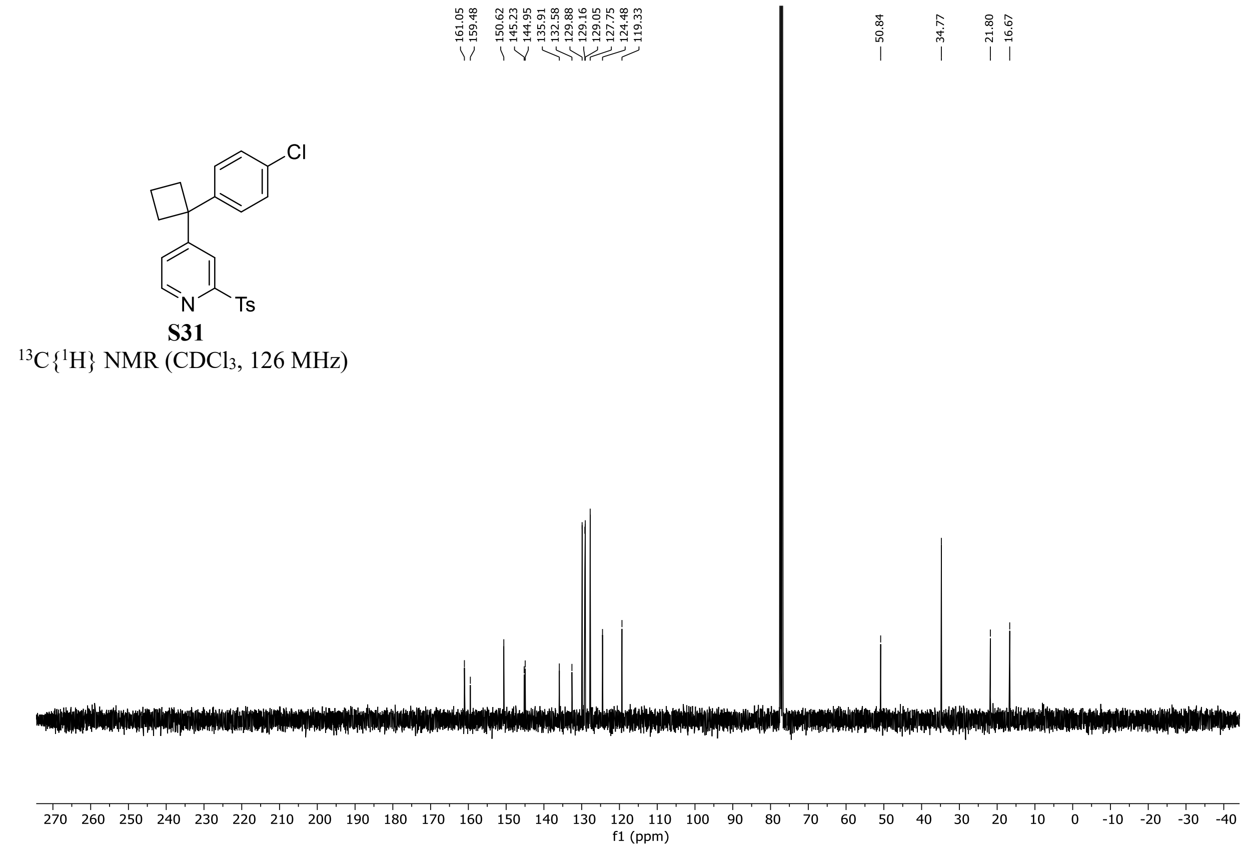

161.05
159.48
150.62
145.23
144.95
135.91
132.58
129.88
129.16
129.05
127.75
124.48
119.33
50.84
34.77
21.80
16.67
Cl
N
Ts
S31
13C{1H} NMR (CDCl3, 126 MHz)
f1 (ppm)

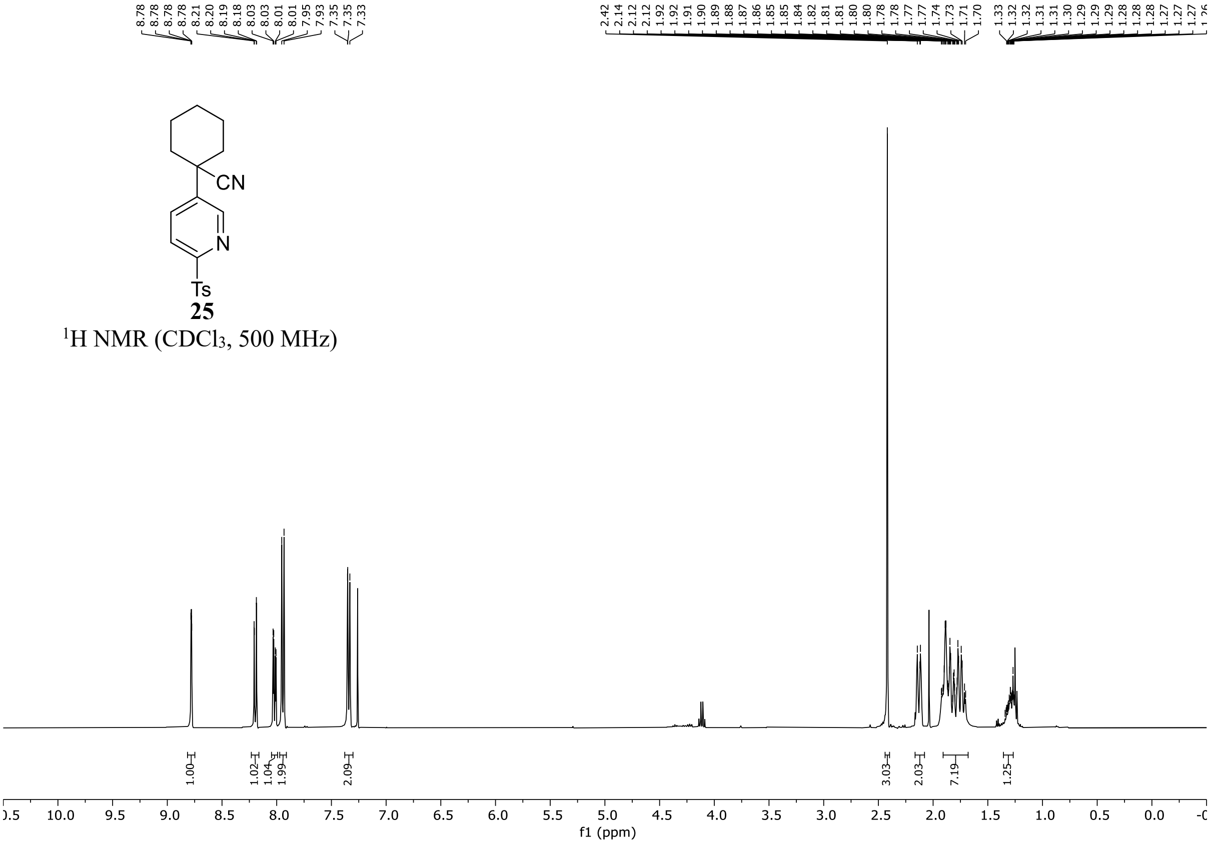
8.78 8.78 8.78 8.78 8.21 8.20 8.19 8.18 8.03 8.03 8.01 8.01 7.95 7.93 7.35 7.35 7.33
2.42 2.14 2.12 2.12 1.92 1.92 1.91 1.90 1.89 1.88 1.87 1.86 1.85 1.85 1.84 1.82 1.81 1.81 1.80 1.80 1.78 1.78 1.77 1.77 1.74 1.73 1.71 1.70
1.33 1.32 1.31 1.30 1.29 1.29 1.29 1.28 1.28 1.27 1.27
CN
N
Ts
25
1H NMR (CDCl3, 500 MHz)
1.00
1.02
1.04
1.99
2.09
3.03
2.03
7.19
1.25
10.0 9.5 9.0 8.5 8.0 7.5 7.0 6.5 6.0 5.5 5.0 4.5 4.0 3.5 3.0 2.5 2.0 1.5 1.0 0.5 0.0
f1 (ppm)

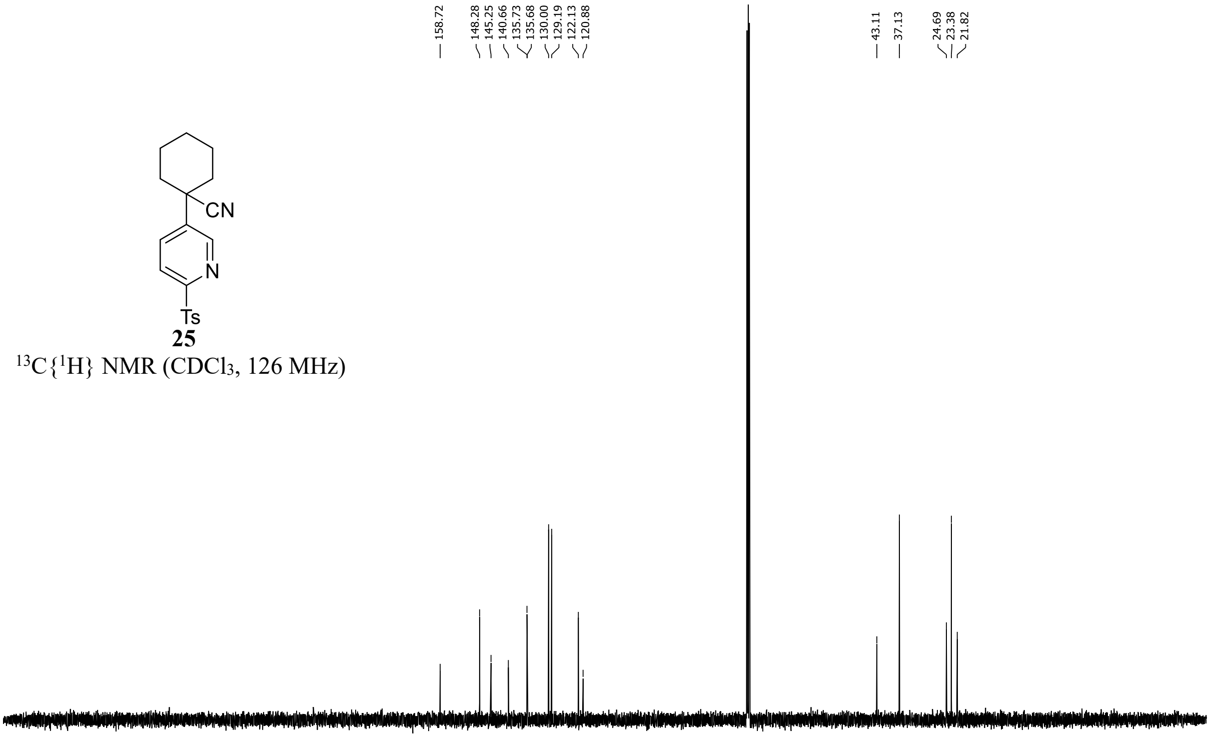
158.72
148.28 145.25 140.66 135.73 135.68 130.00 129.19 122.13 120.88
43.11
37.13
24.69 23.38 21.82
CN
N
Ts
25
13C{1H} NMR (CDCl3, 126 MHz)
270 260 250 240 230 220 210 200 190 180 170 160 150 140 130 120 110 100 90 80 70 60 50 40 30 20 10 0 -10 -20 -30 -40
f1 (ppm)

26

^{1}H NMR ($CDCl_3$, 500 MHz)

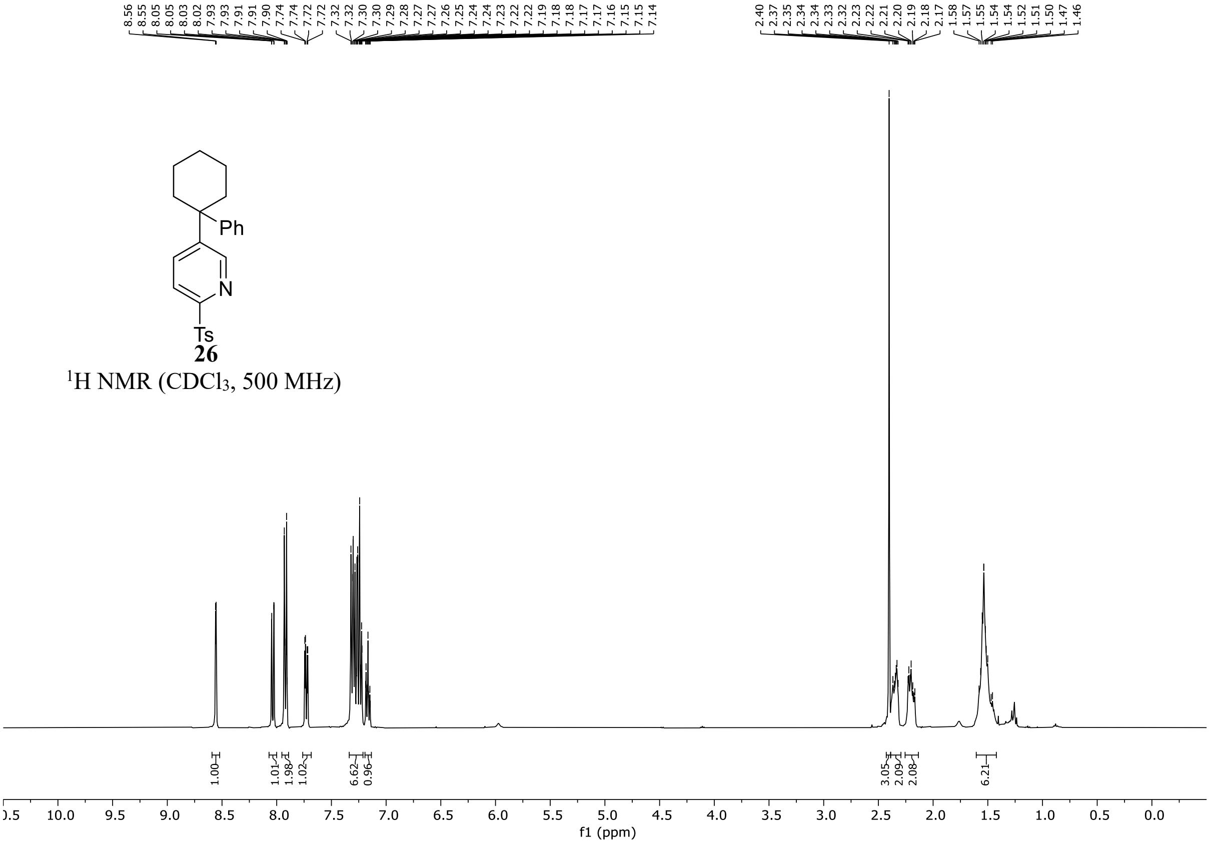

26

^{13}C{^{1}H} NMR ($CDCl_3$, 126 MHz)

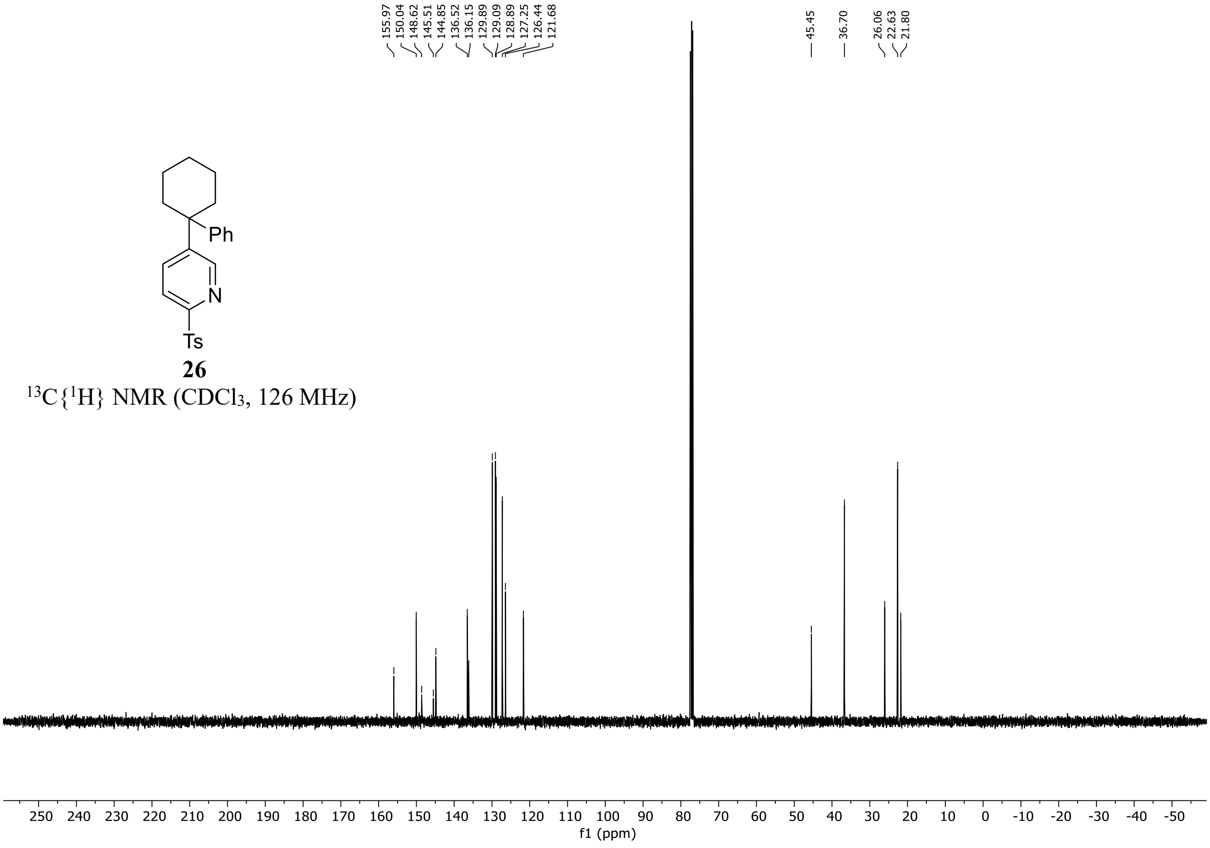

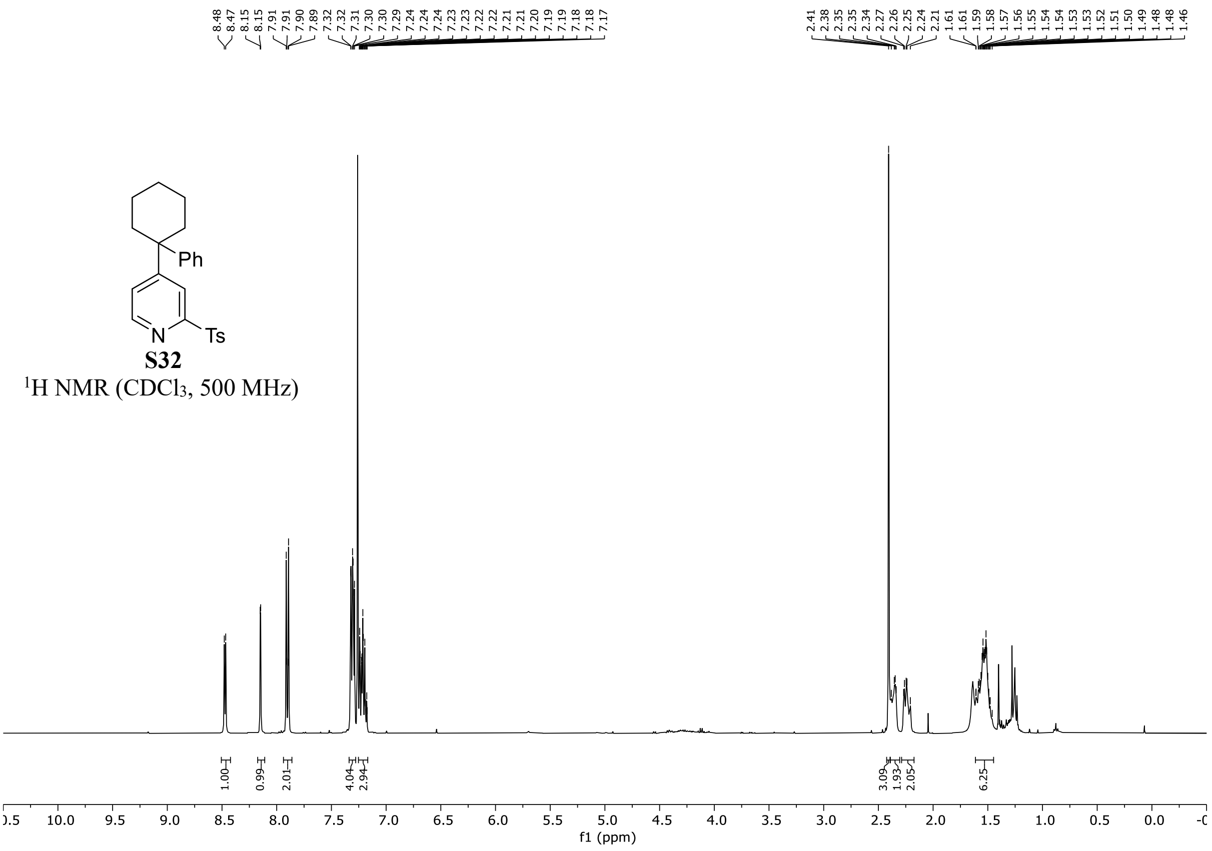

S32

^{1}H NMR ($CDCl_3$, 500 MHz)

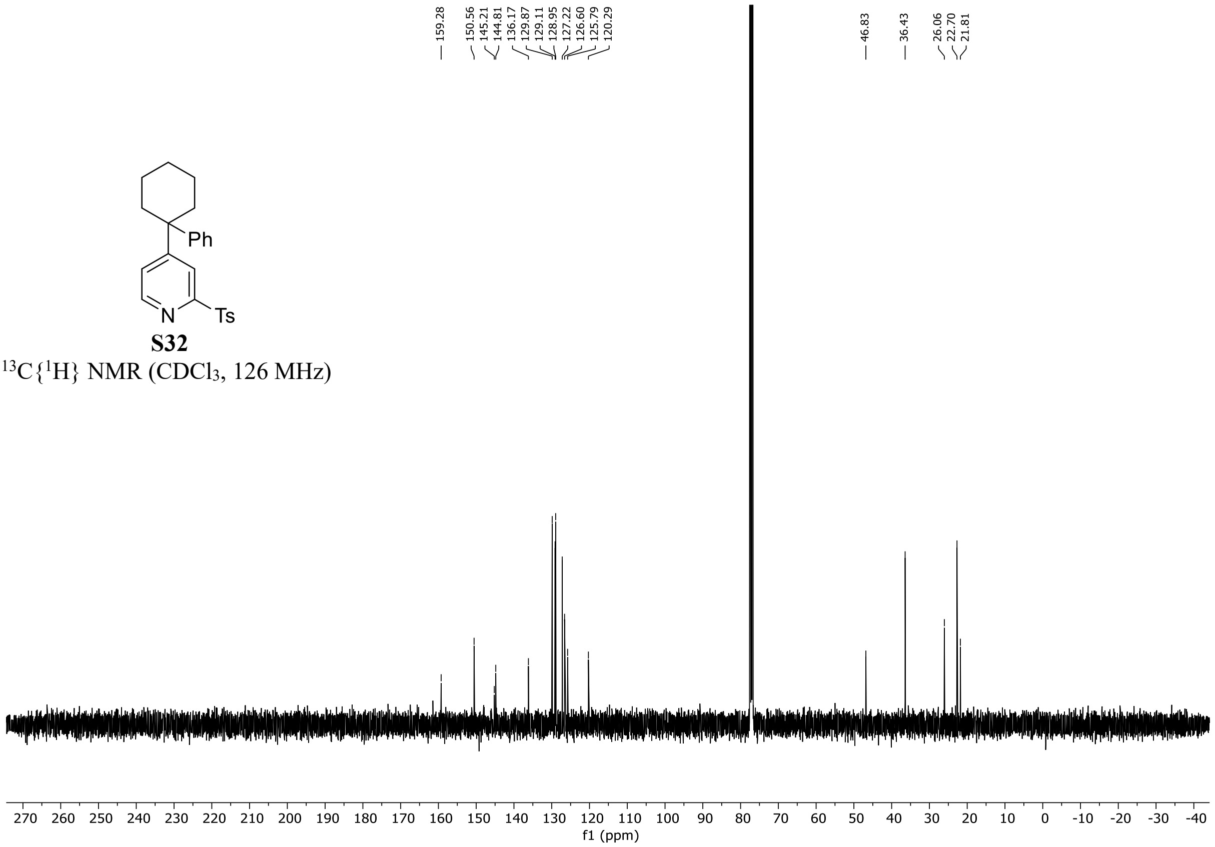

S32

$^{13}C\{^{1}H\}$ NMR ($CDCl_3$, 126 MHz)

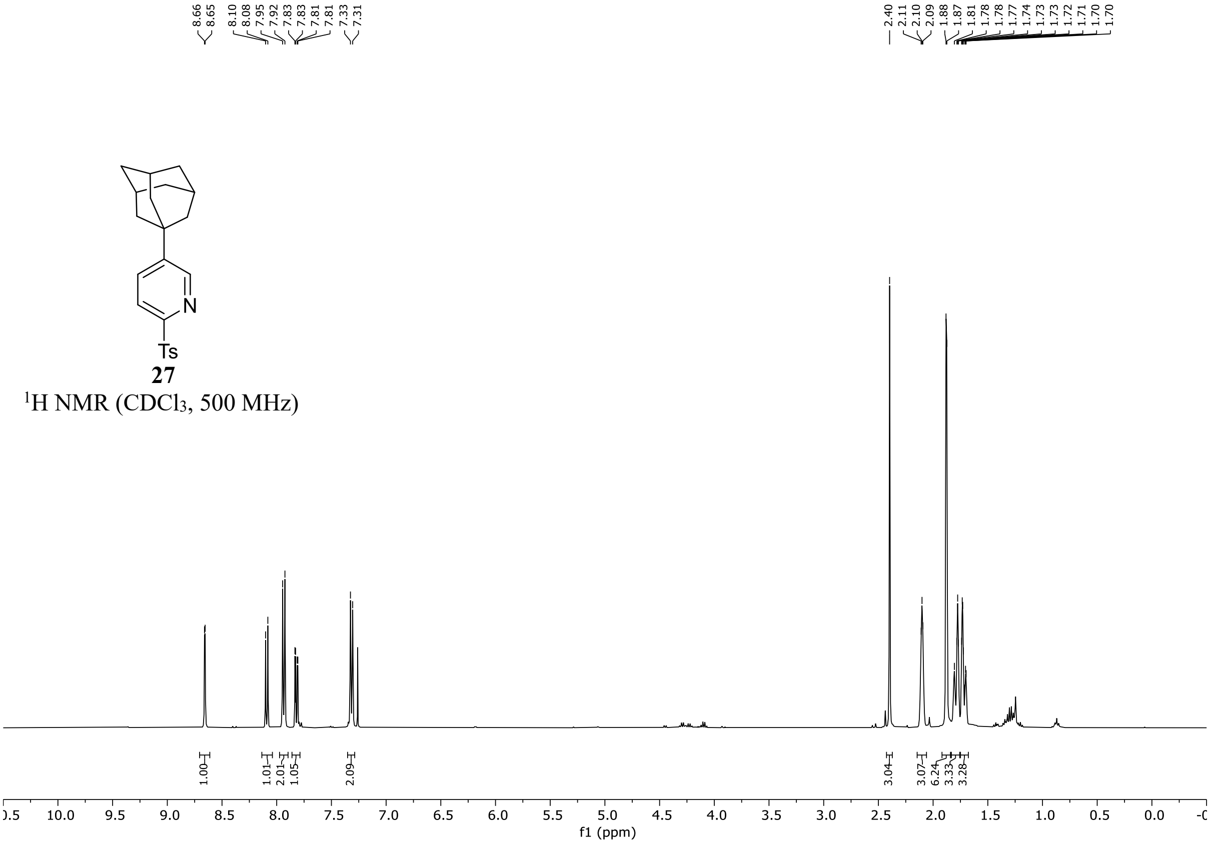
8.66
8.65
8.10
8.08
7.95
7.92
7.83
7.83
7.81
7.81
7.33
7.31
2.40
2.11
2.10
2.09
1.88
1.87
1.81
1.78
1.77
1.74
1.73
1.72
1.71
1.70
1.70
N
Ts
27
¹H NMR (CDCl₃, 500 MHz)
1.00
1.01
2.01
1.05
2.09
3.04
3.07
6.24
3.33
3.28
f1 (ppm)

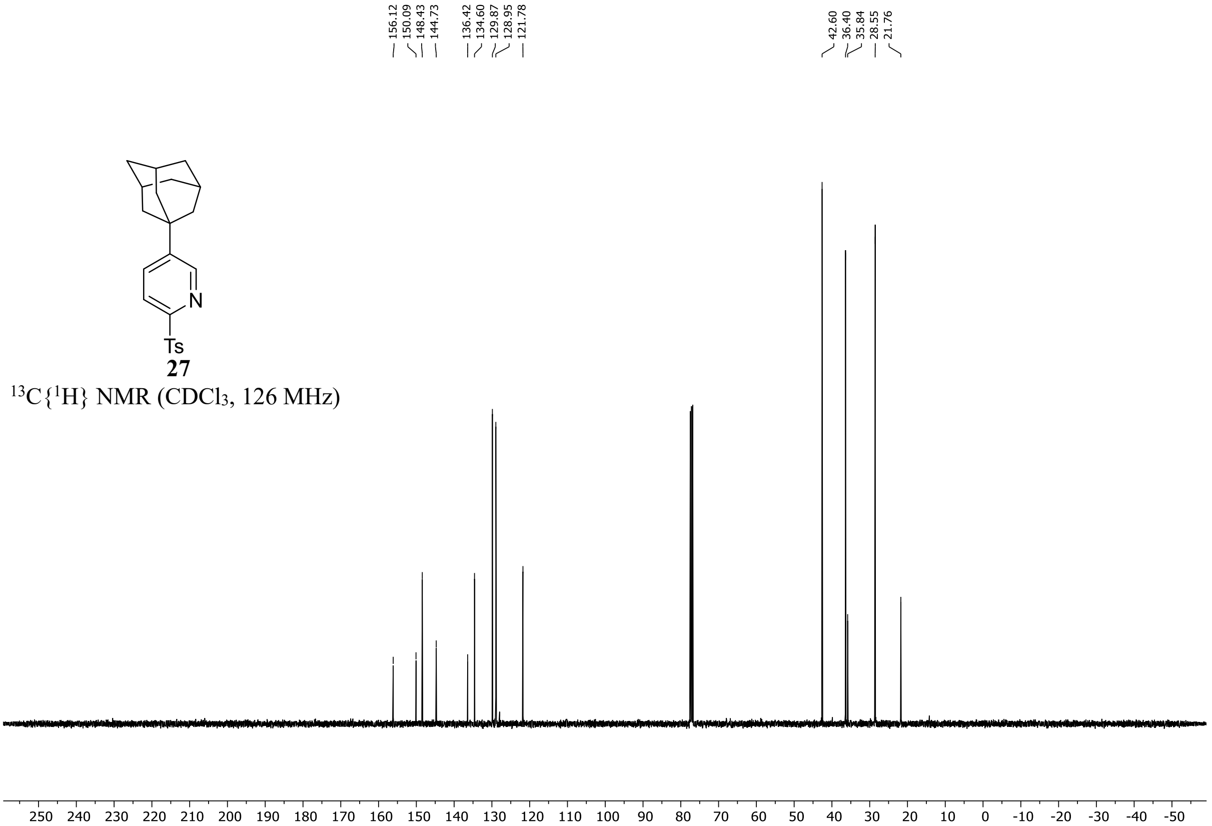
156.12
150.09
148.43
144.73
136.42
134.60
129.87
128.95
121.78
42.60
36.40
35.84
28.55
21.76
N
Ts
27
¹³C{¹H} NMR (CDCl₃, 126 MHz)
f1 (ppm)

S33
^{1}H NMR (CDCl$_3$, 500 MHz)

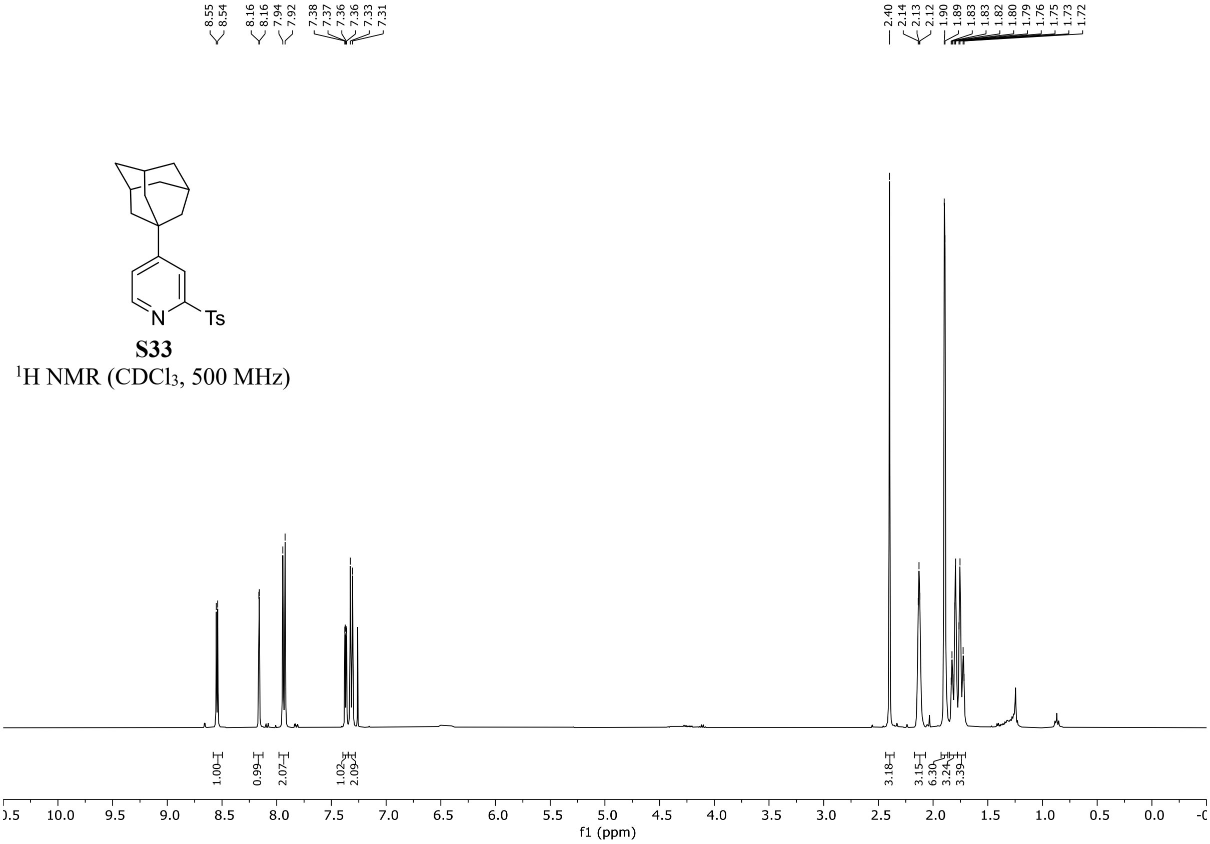

S33
^{13}C{^{1}H} NMR (CDCl$_3$, 126 MHz)

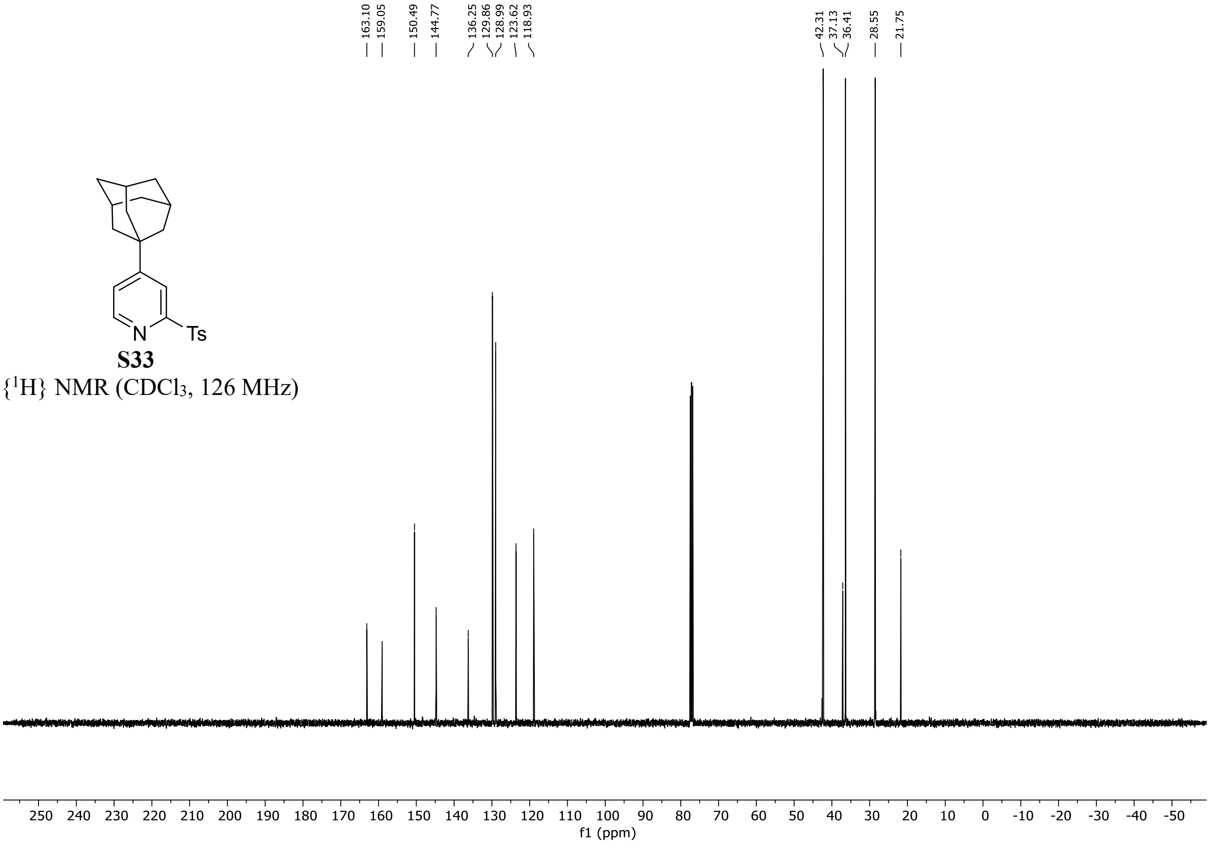

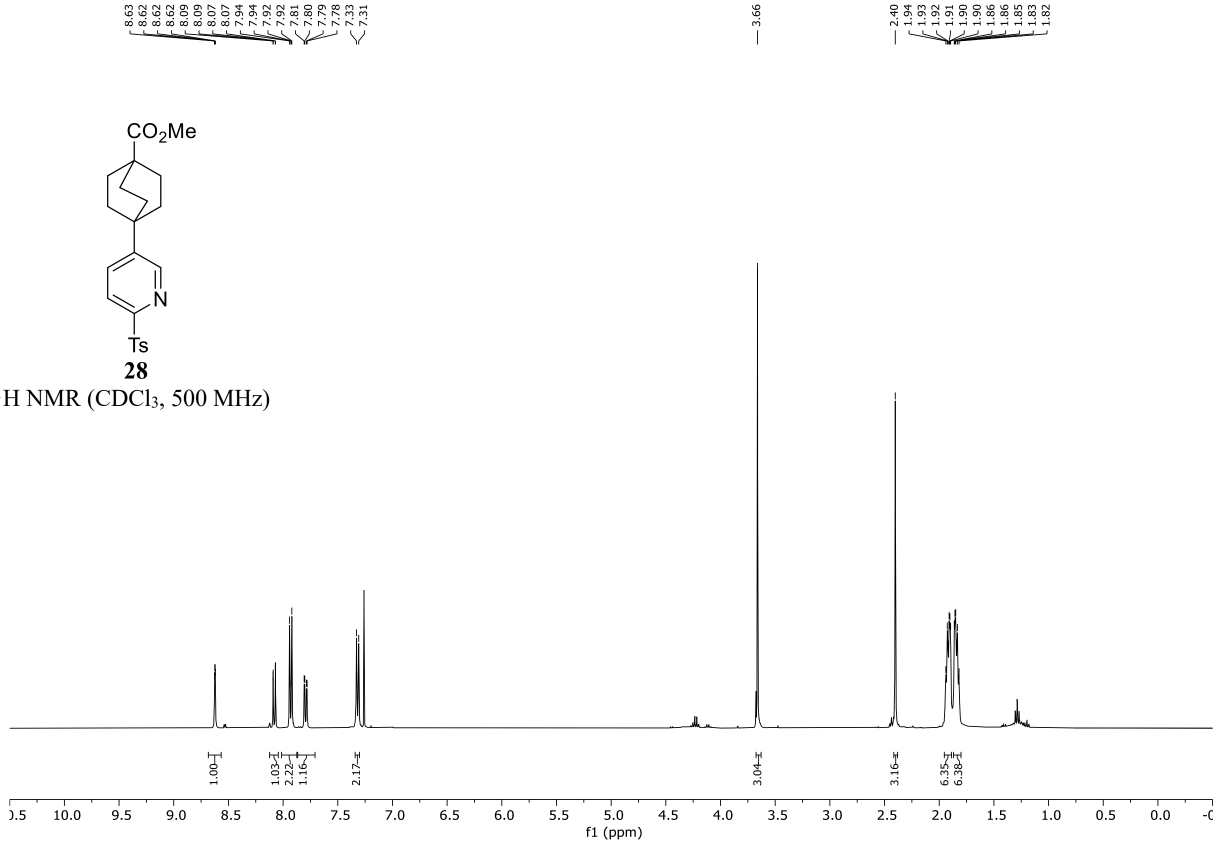
8.63
8.62
8.62
8.09
8.09
8.07
8.07
7.94
7.94
7.92
7.92
7.81
7.80
7.79
7.78
7.33
7.31
3.66
2.40
1.94
1.93
1.92
1.91
1.91
1.90
1.90
1.86
1.86
1.85
1.83
1.82
CO2Me
N
Ts
28
1H NMR (CDCl3, 500 MHz)
1.00
1.03
2.22
1.16
2.17
3.04
3.16
6.35
6.38
f1 (ppm)

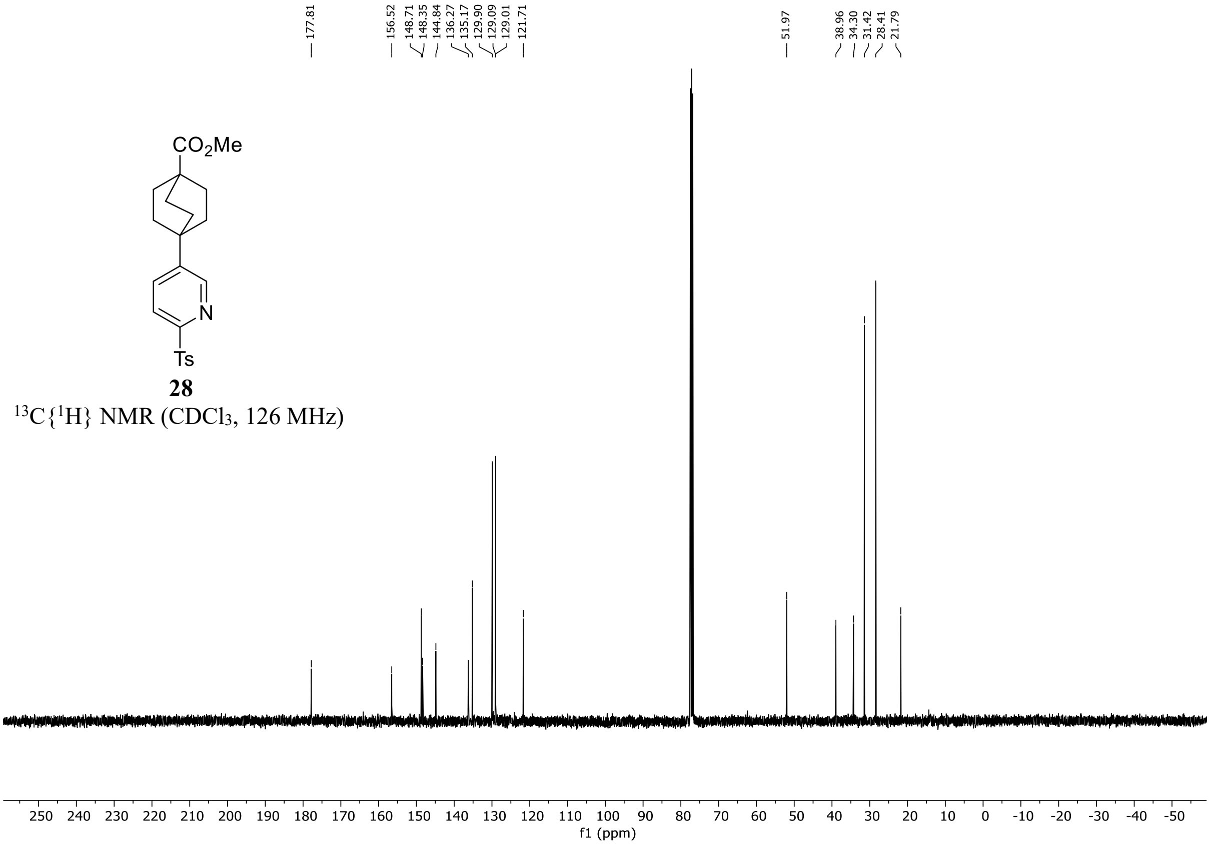
177.81
156.52
148.71
148.35
144.84
136.27
135.17
129.90
129.09
129.01
121.71
51.97
38.96
34.30
31.42
28.41
21.79
CO2Me
N
Ts
28
13C{1H} NMR (CDCl3, 126 MHz)
f1 (ppm)

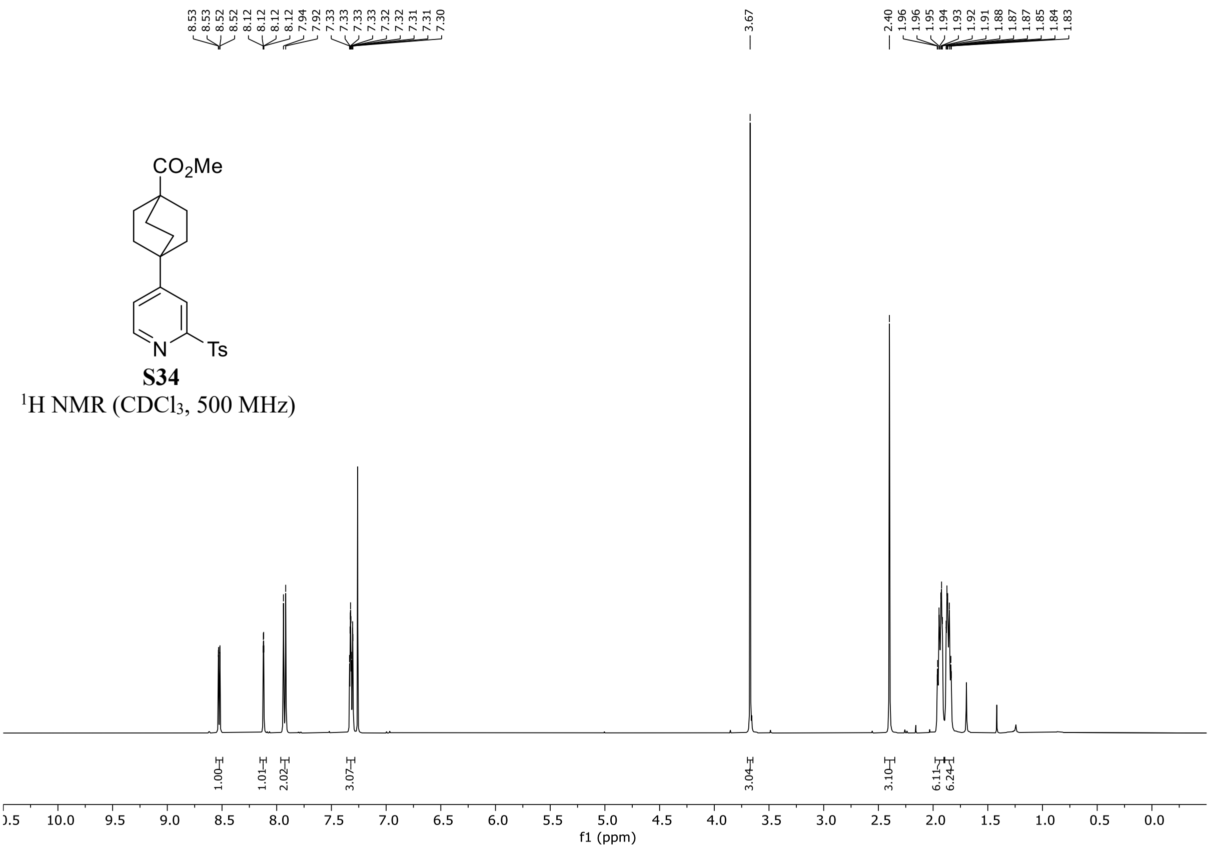
8.53 8.53 8.52 8.52 8.12 8.12 8.12 8.12 7.94 7.92 7.33 7.33 7.33 7.33 7.32 7.32 7.31 7.31 7.30
3.67
2.40 1.96 1.96 1.95 1.94 1.93 1.92 1.91 1.88 1.87 1.87 1.85 1.84 1.83
CO2Me
N
Ts
S34
1H NMR (CDCl3, 500 MHz)
1.00 1.01 2.02 3.07 3.04 3.10 6.11 6.24
f1 (ppm)

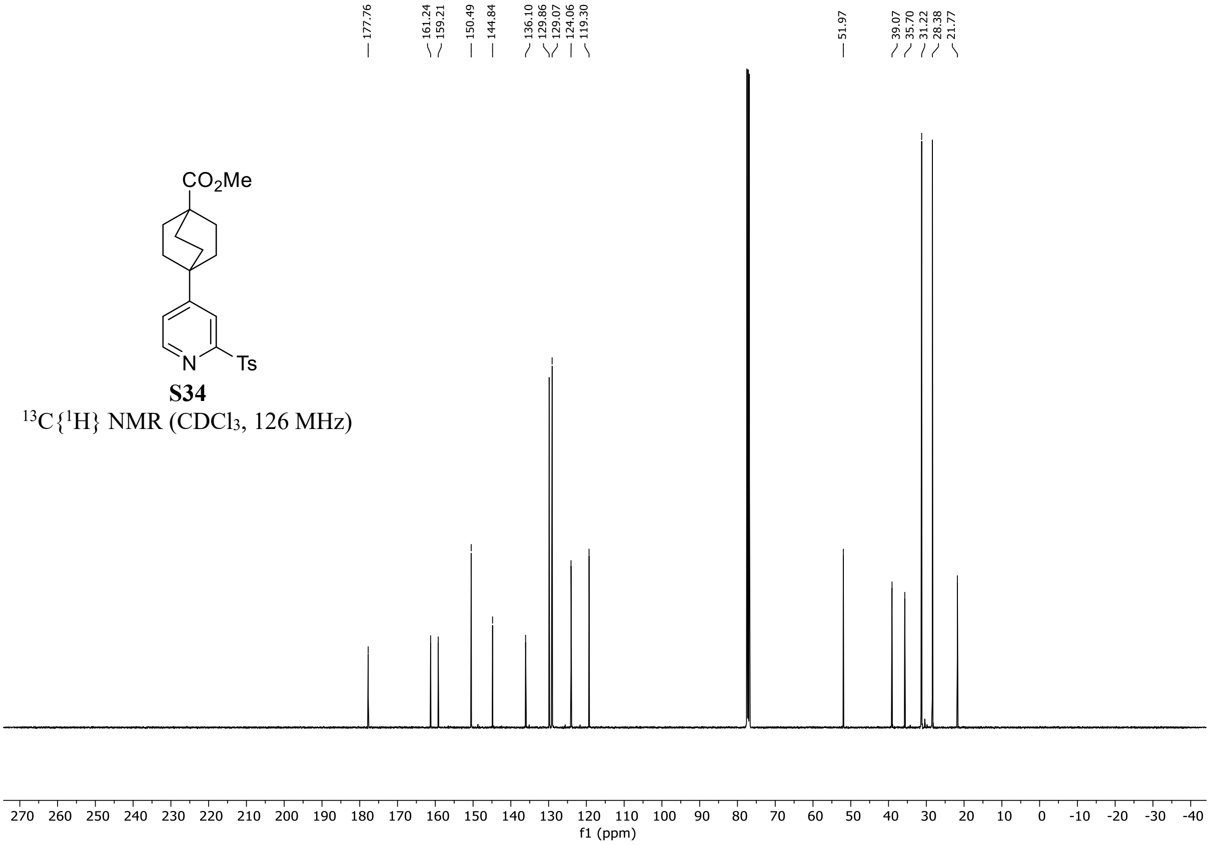
177.76
161.24 159.21 150.49 144.84 136.10 129.86 129.07 124.06 119.30
51.97
39.07 35.70 31.22 28.38 21.77
CO2Me
N
Ts
S34
13C{1H} NMR (CDCl3, 126 MHz)
f1 (ppm)

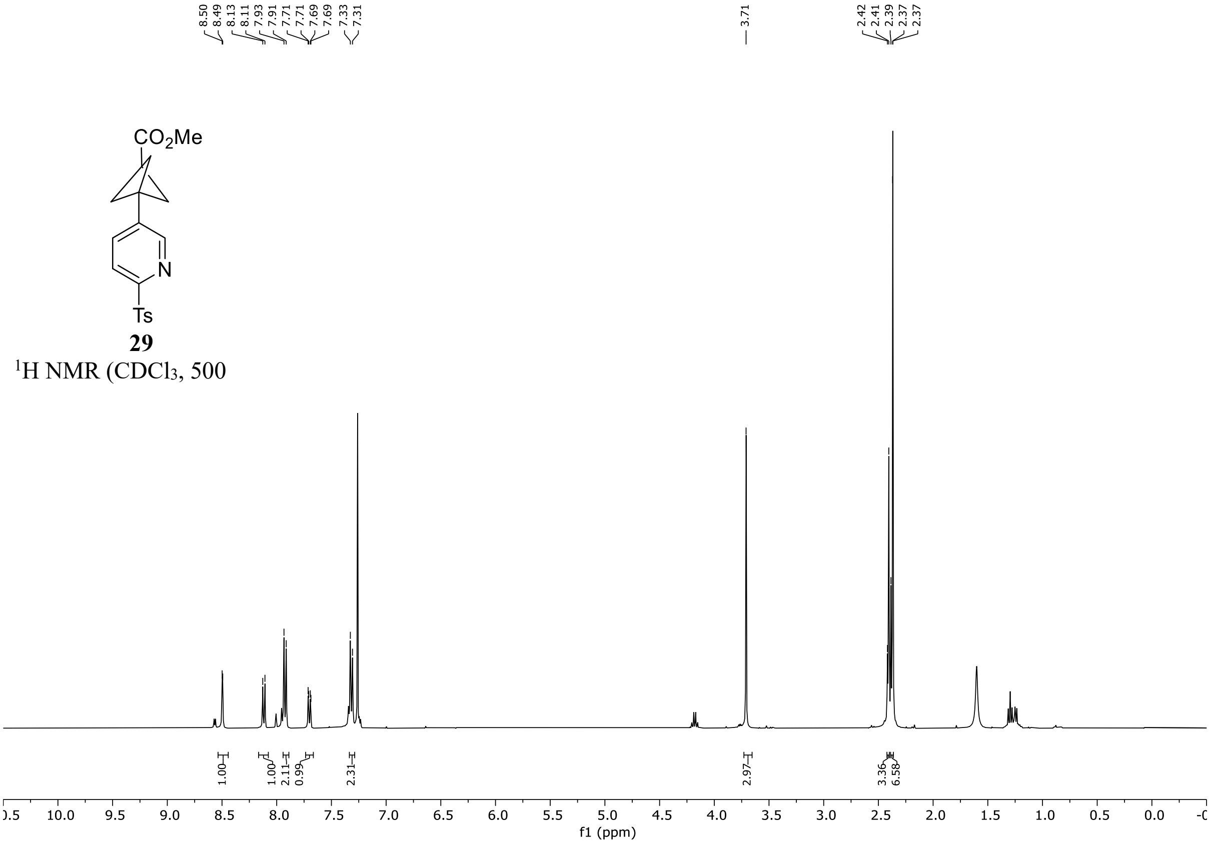

29

^{1}H NMR ($CDCl_3$, 500

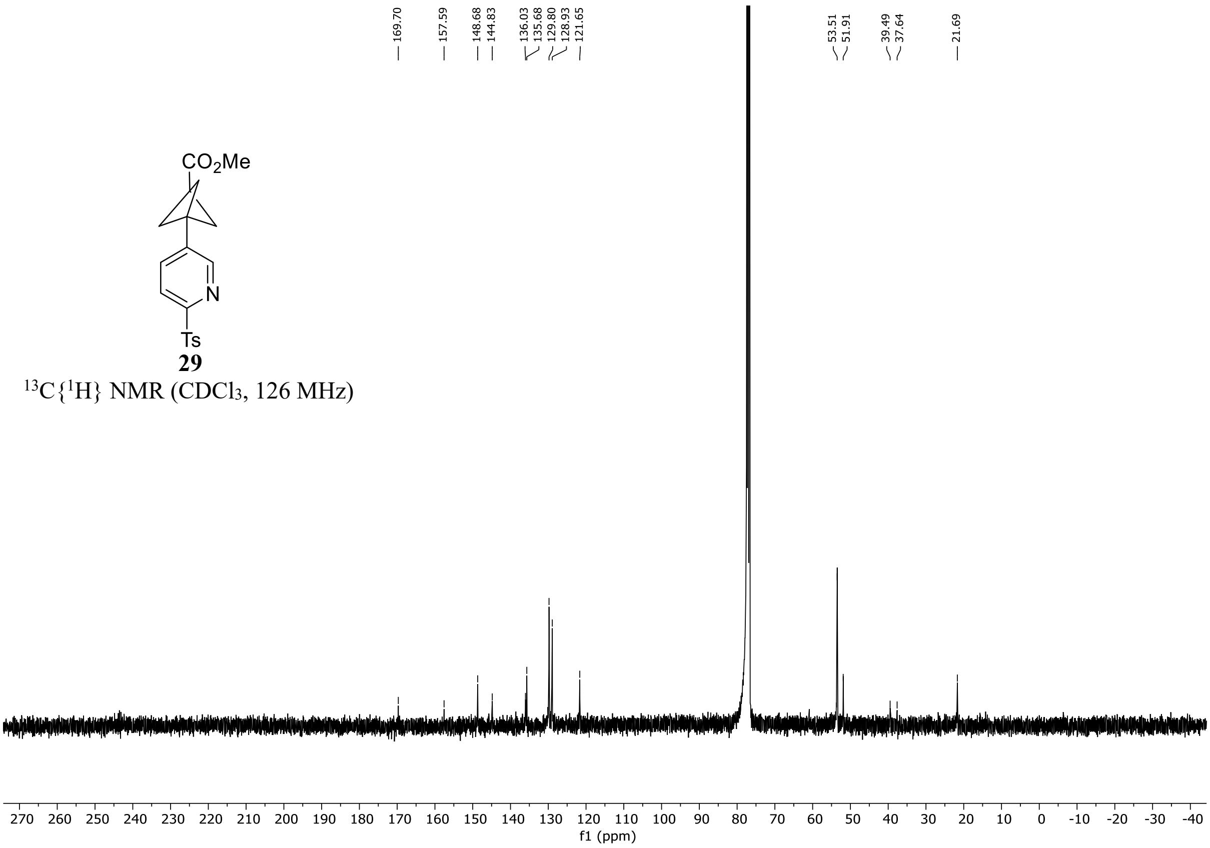

29

$^{13}C\{^{1}H\}$ NMR ($CDCl_3$, 126 MHz)

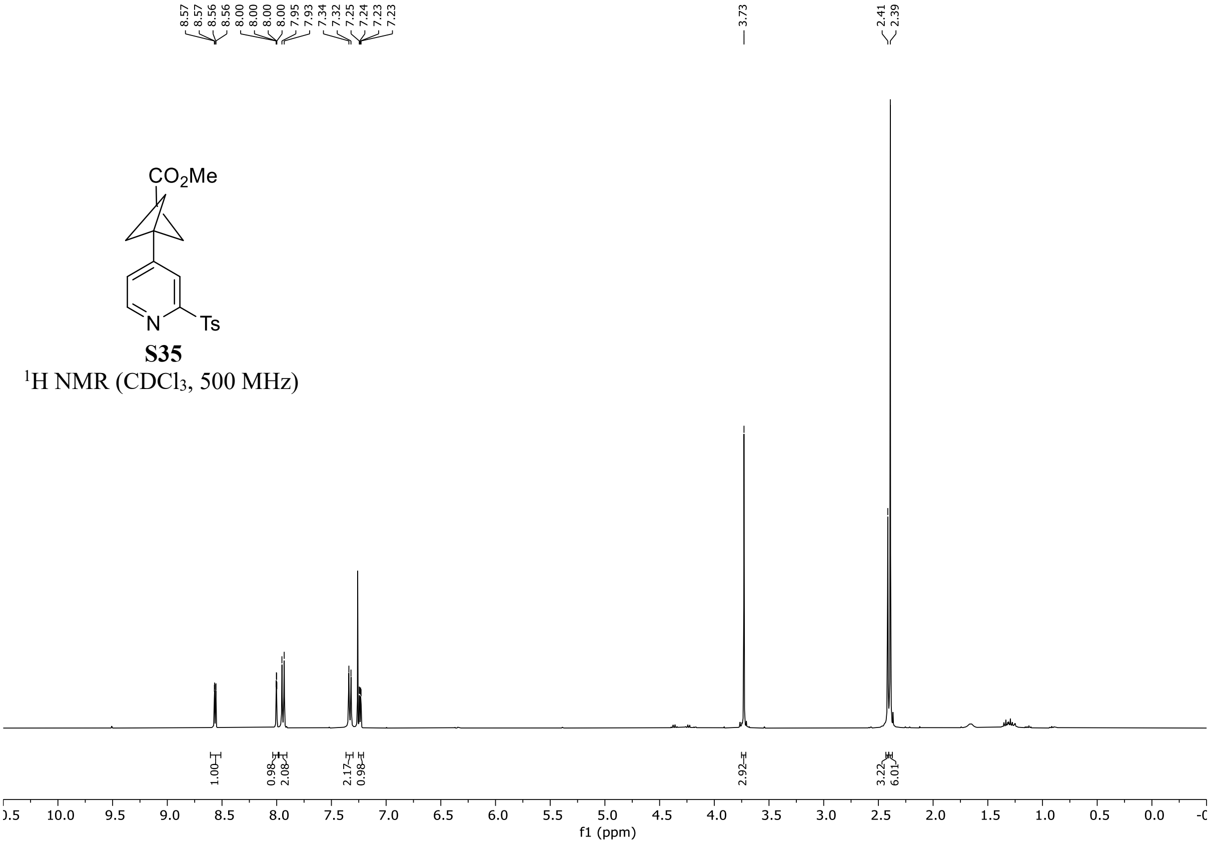

S35
^{1}H NMR ($CDCl_3$, 500 MHz)

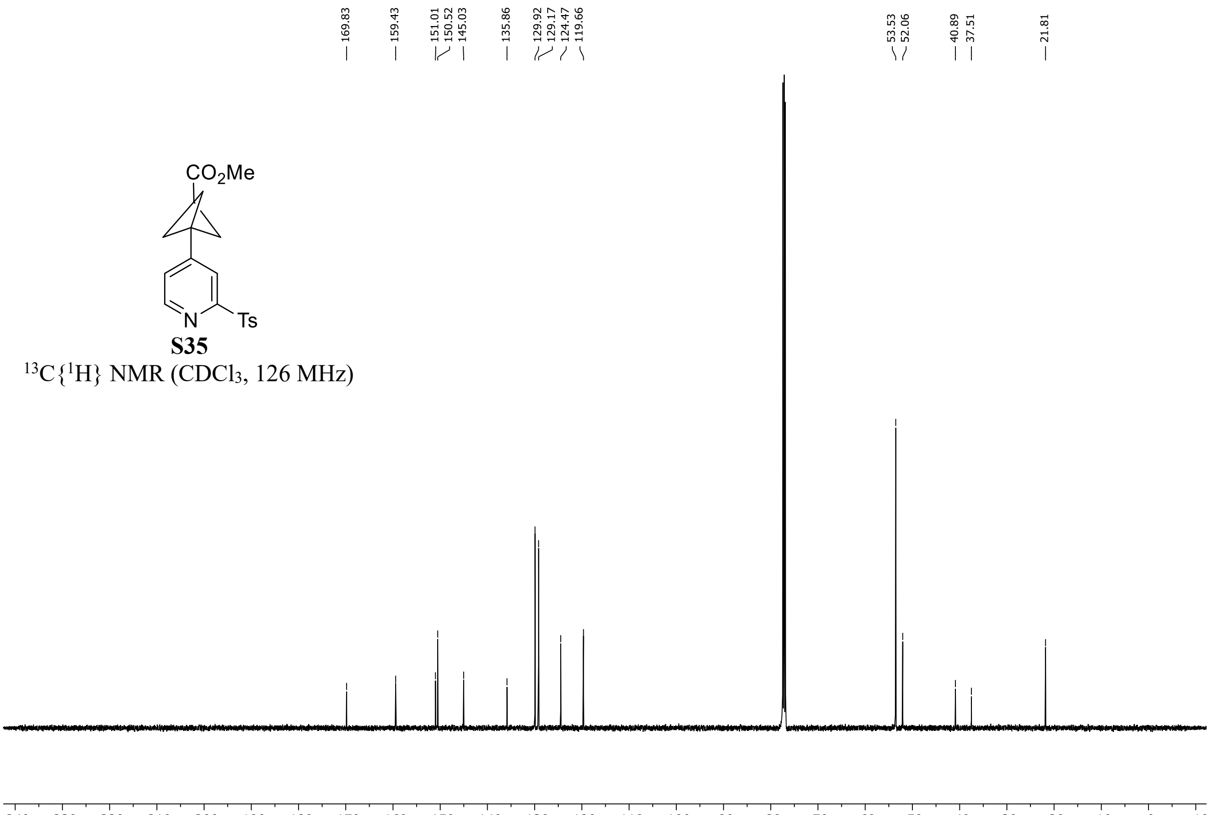

S35
$^{13}C\{^{1}H\}$ NMR ($CDCl_3$, 126 MHz)

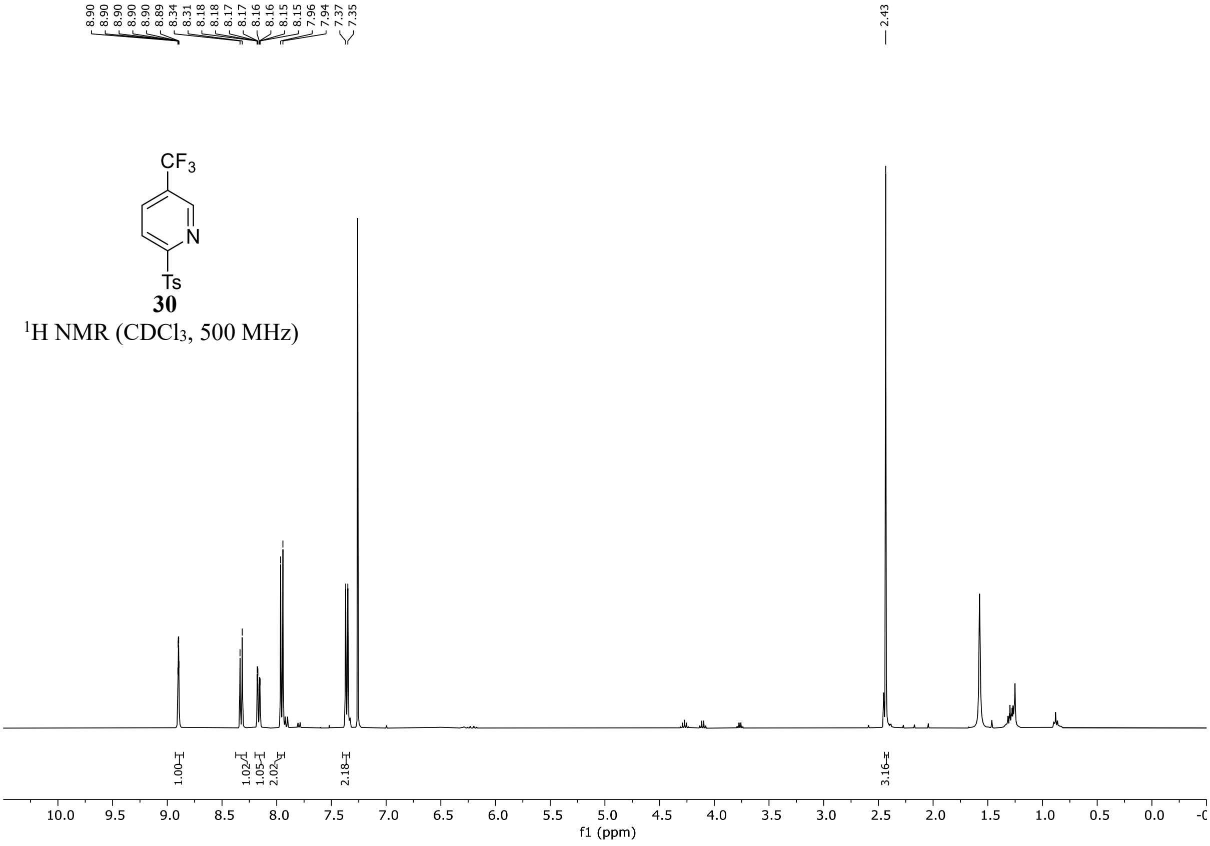

30

^{1}H NMR ($CDCl_3$, 500 MHz)

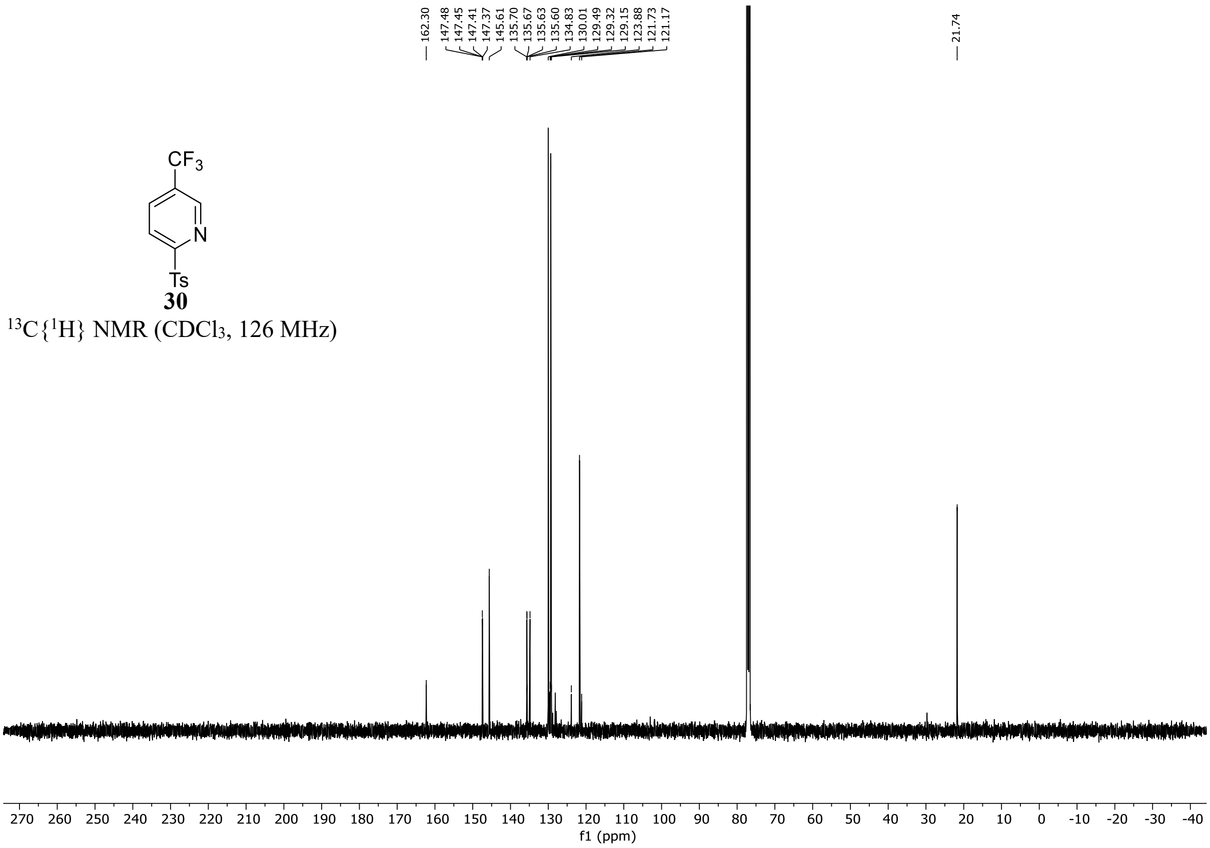

30

$^{13}C\{^{1}H\}$ NMR ($CDCl_3$, 126 MHz)

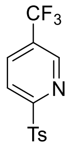

30

$^{19}F\{^{1}H\}$ NMR ($CDCl_3$, 377 MHz)

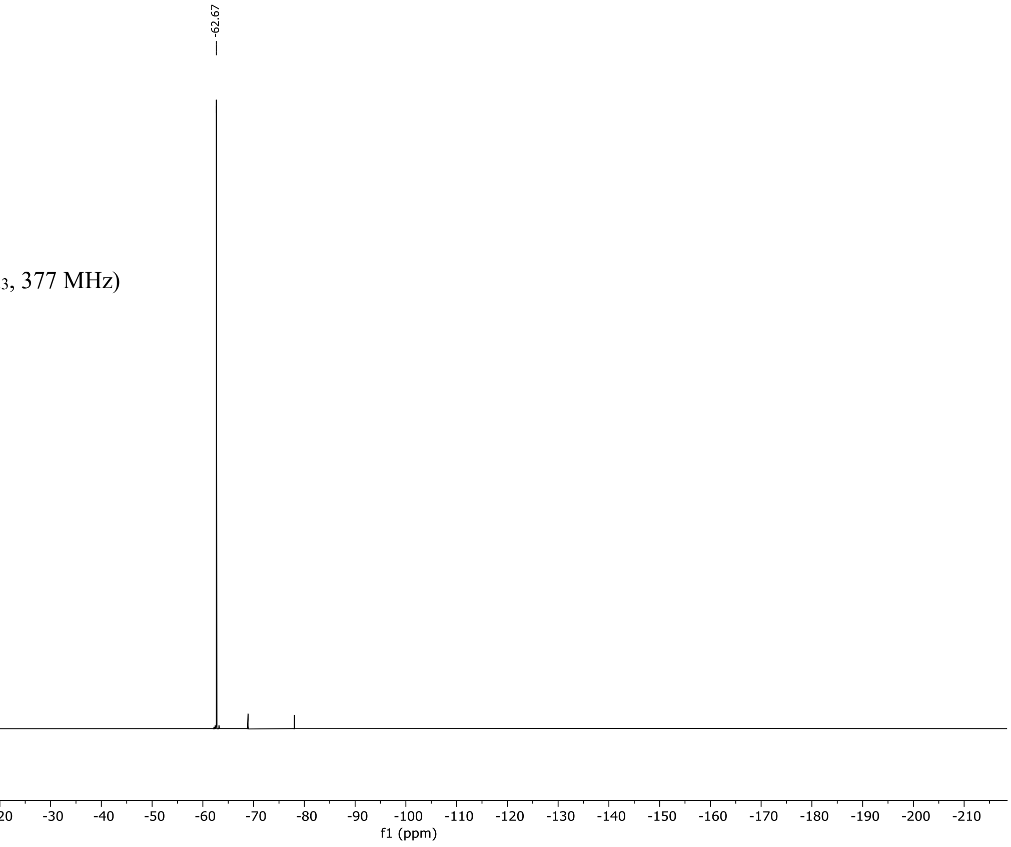

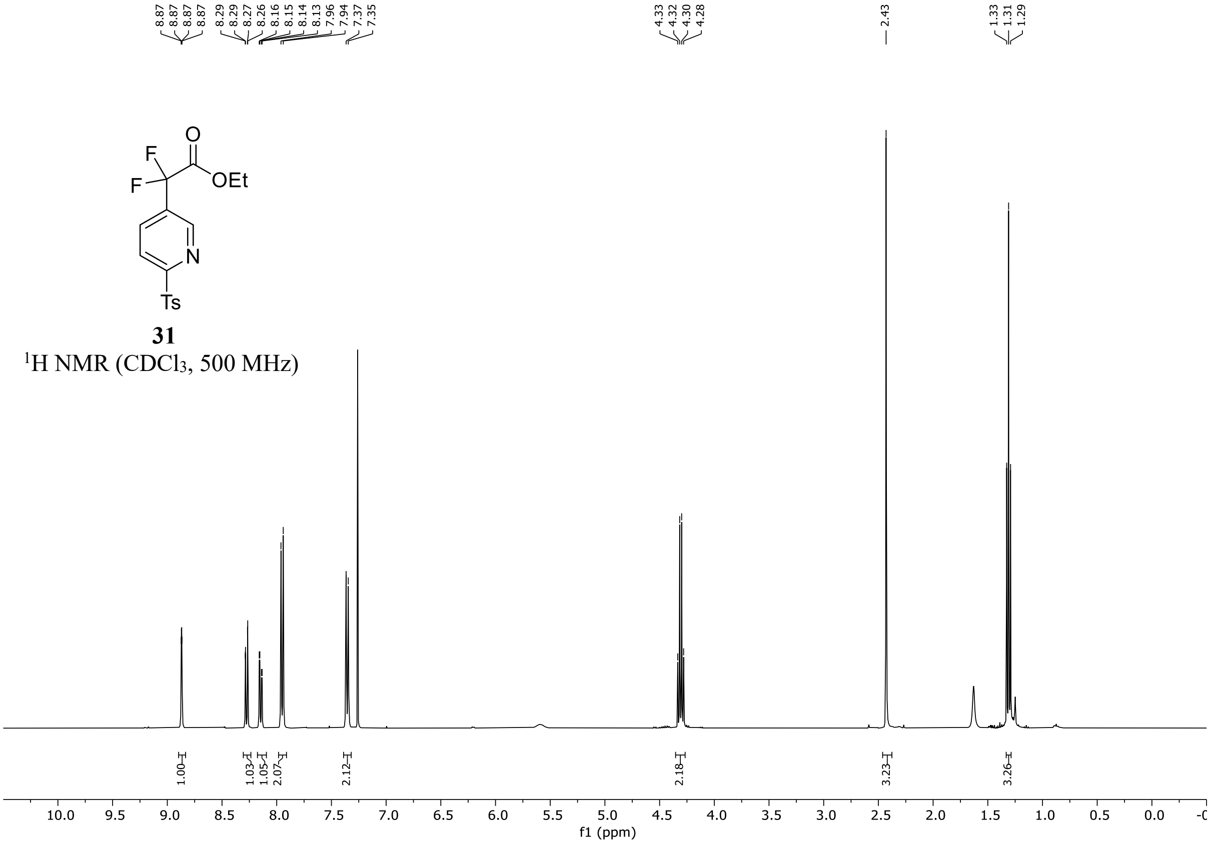

8.87
8.87
8.87
8.87
8.29
8.29
8.27
8.26
8.16
8.15
8.14
8.13
7.96
7.94
7.37
7.35
4.33
4.32
4.30
4.28
2.43
1.33
1.31
1.29
OEt
N
Ts
31
^{1}H NMR ($CDCl_3$, 500 MHz)
1.00
1.03
1.05
2.07
2.12
2.18
3.23
3.26
f1 (ppm)

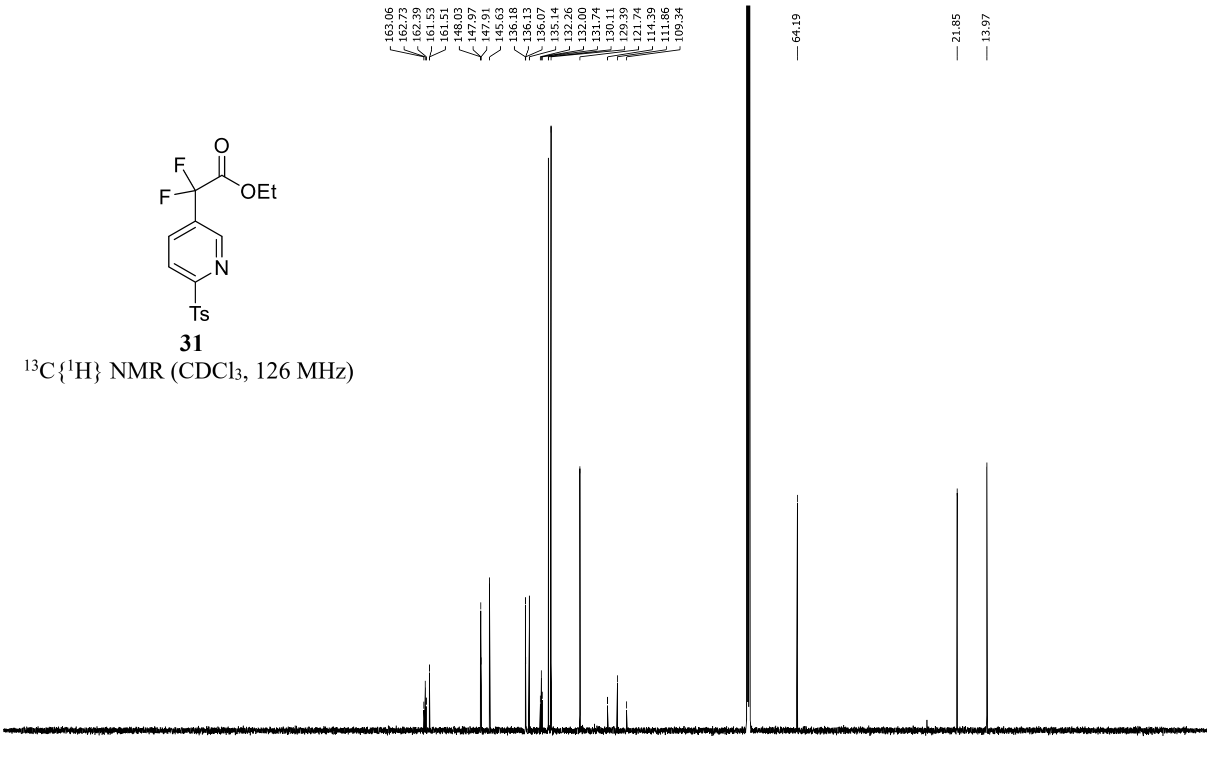

163.06
162.73
162.39
161.53
161.51
148.03
147.97
147.91
145.63
136.18
136.13
136.07
135.14
132.26
132.00
131.74
130.11
129.39
121.74
114.39
111.86
109.34
64.19
21.85
13.97
OEt
N
Ts
31
^{13}C{^{1}H} NMR ($CDCl_3$, 126 MHz)
f1 (ppm)

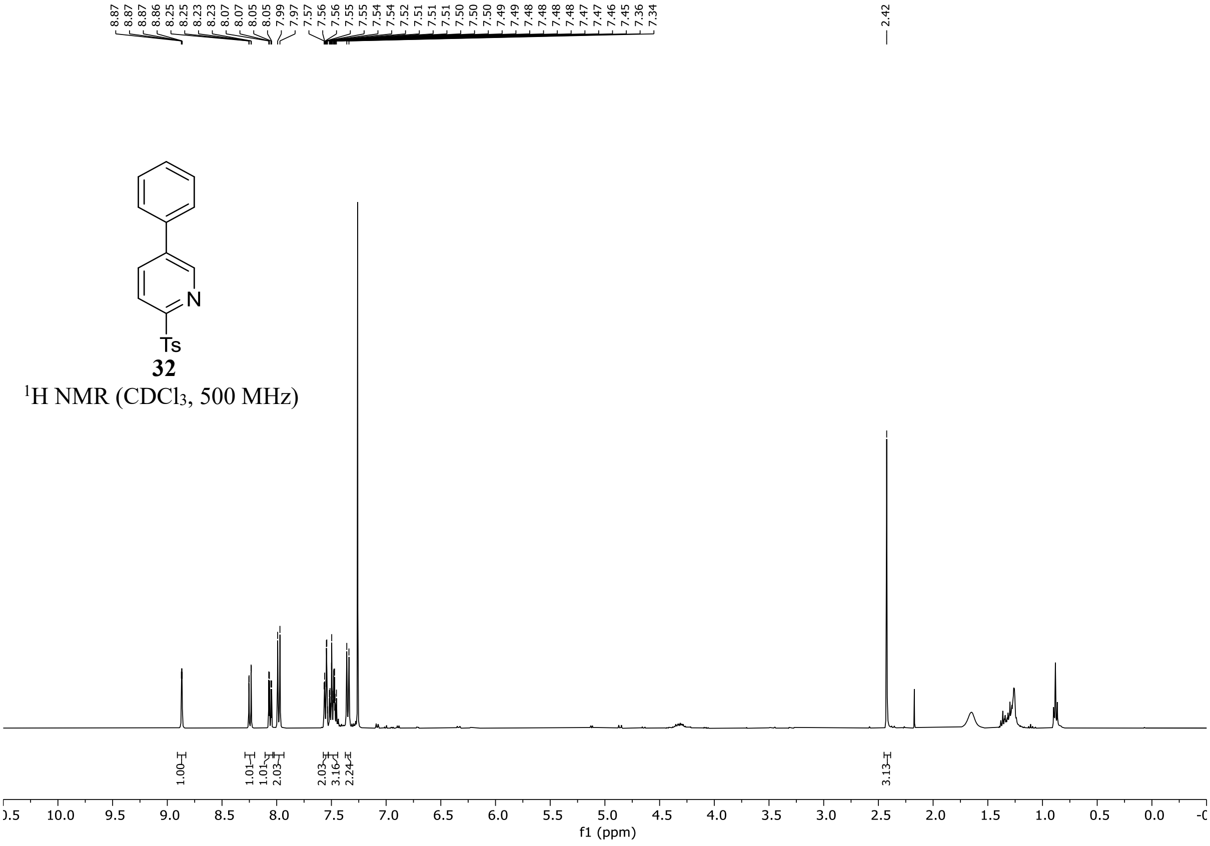

32

^{1}H NMR ($CDCl_3$, 500 MHz)

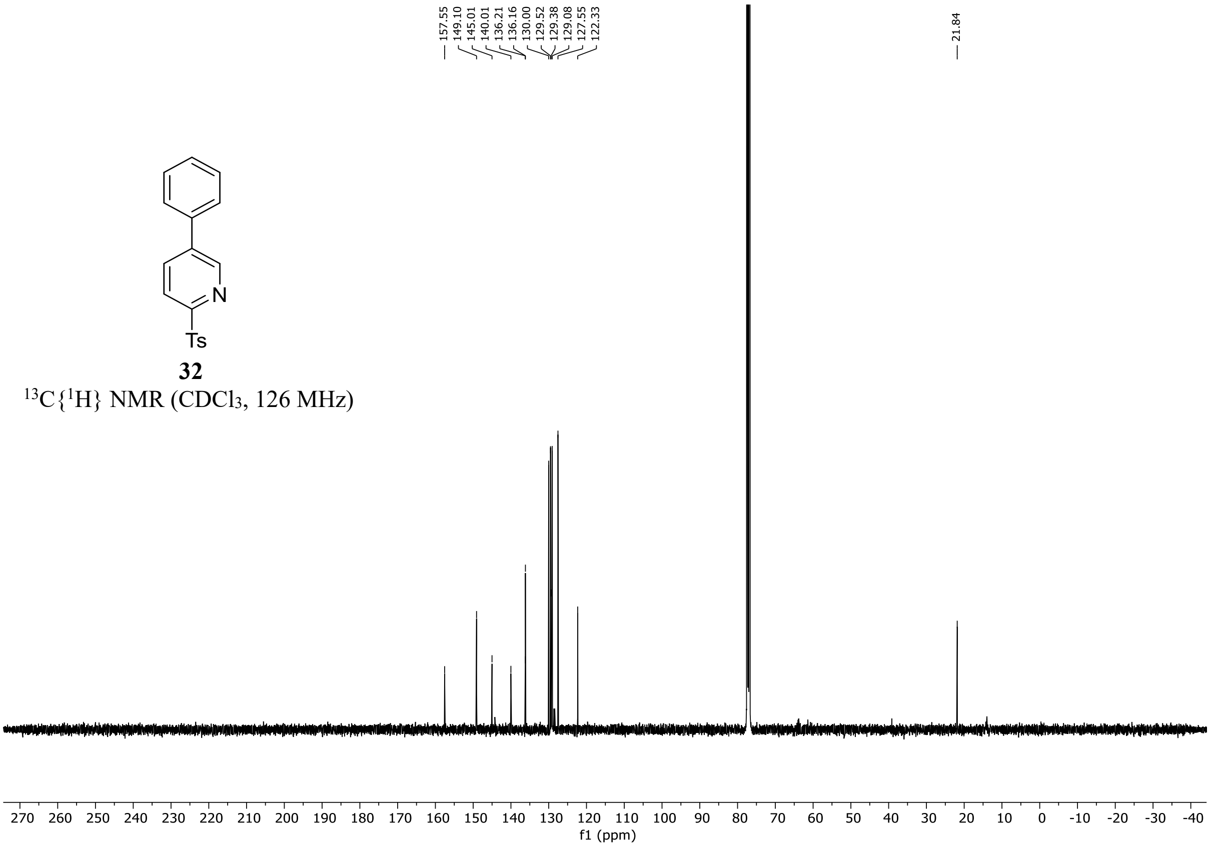

32

^{13}C{^{1}H} NMR ($CDCl_3$, 126 MHz)

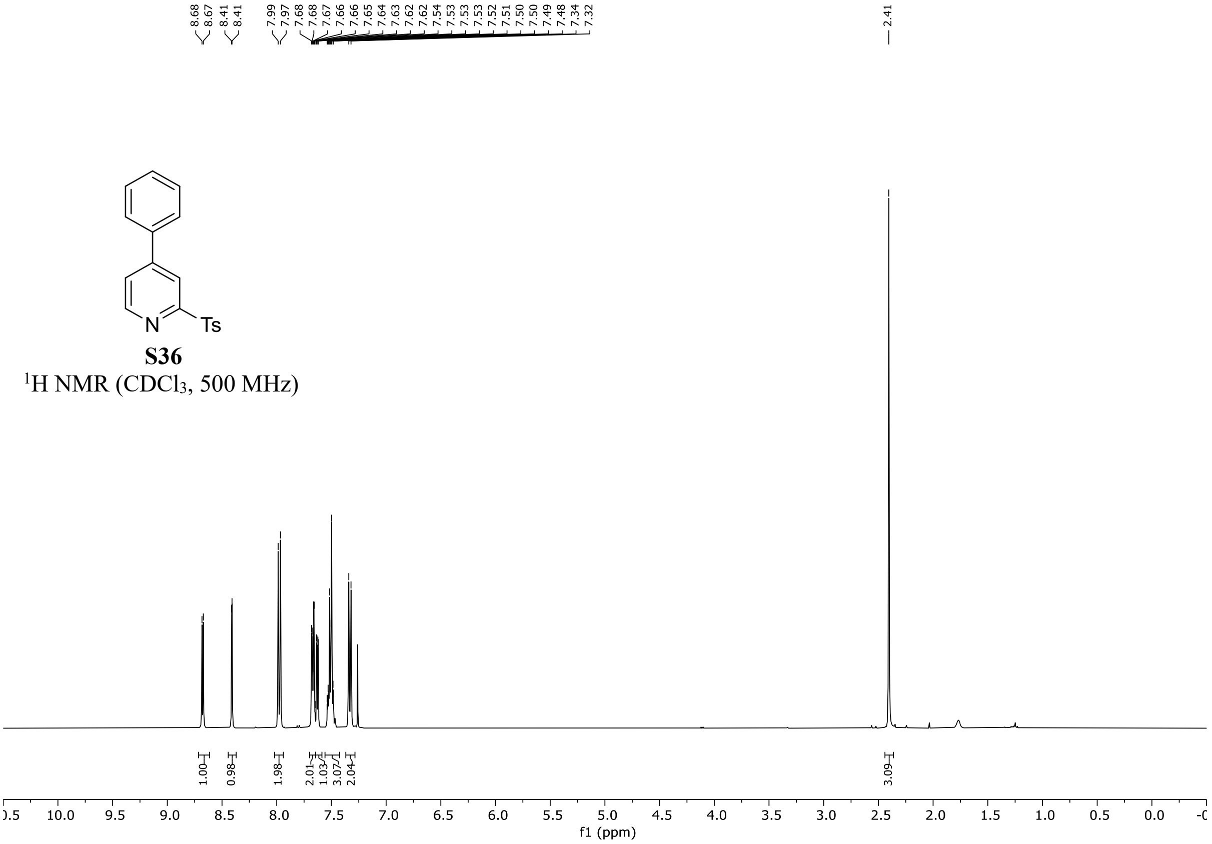

S36
^{1}H NMR ($CDCl_3$, 500 MHz)

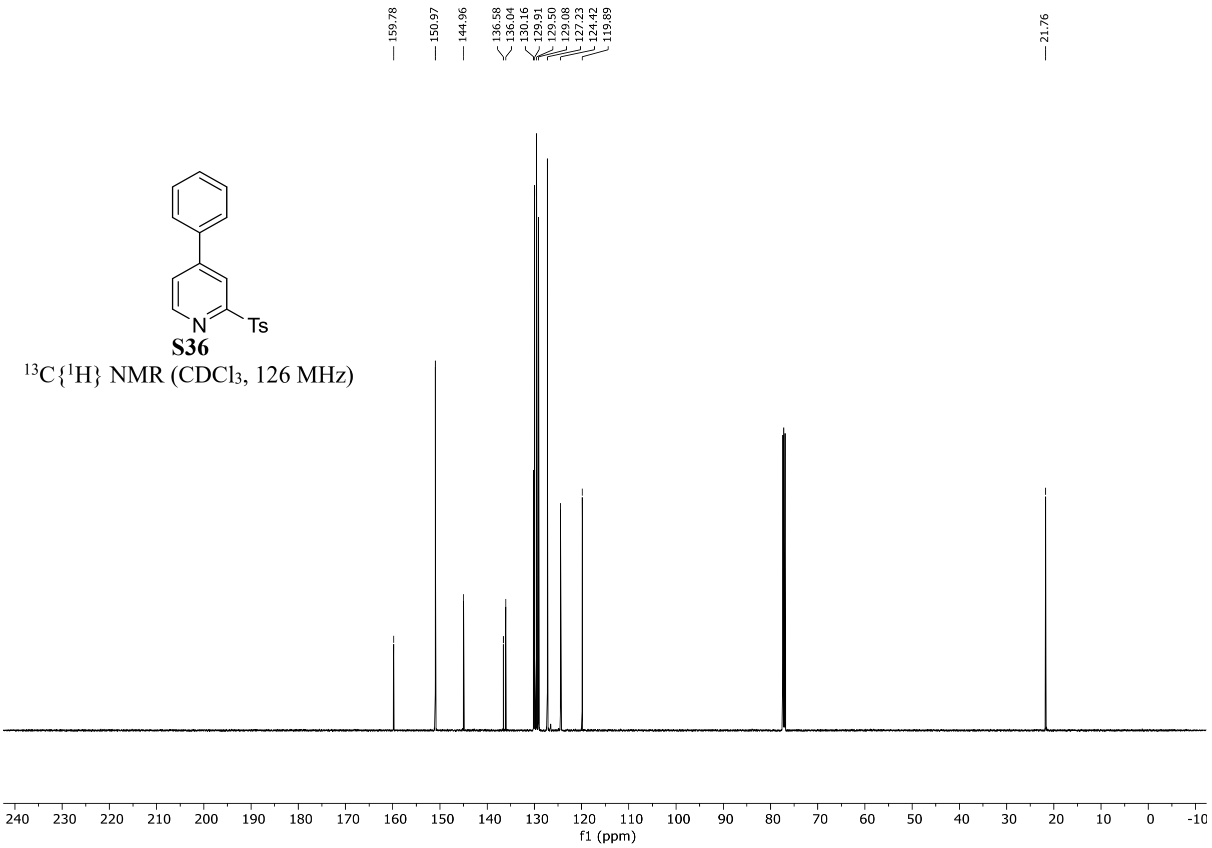

S36
$^{13}C\{^{1}H\}$ NMR ($CDCl_3$, 126 MHz)

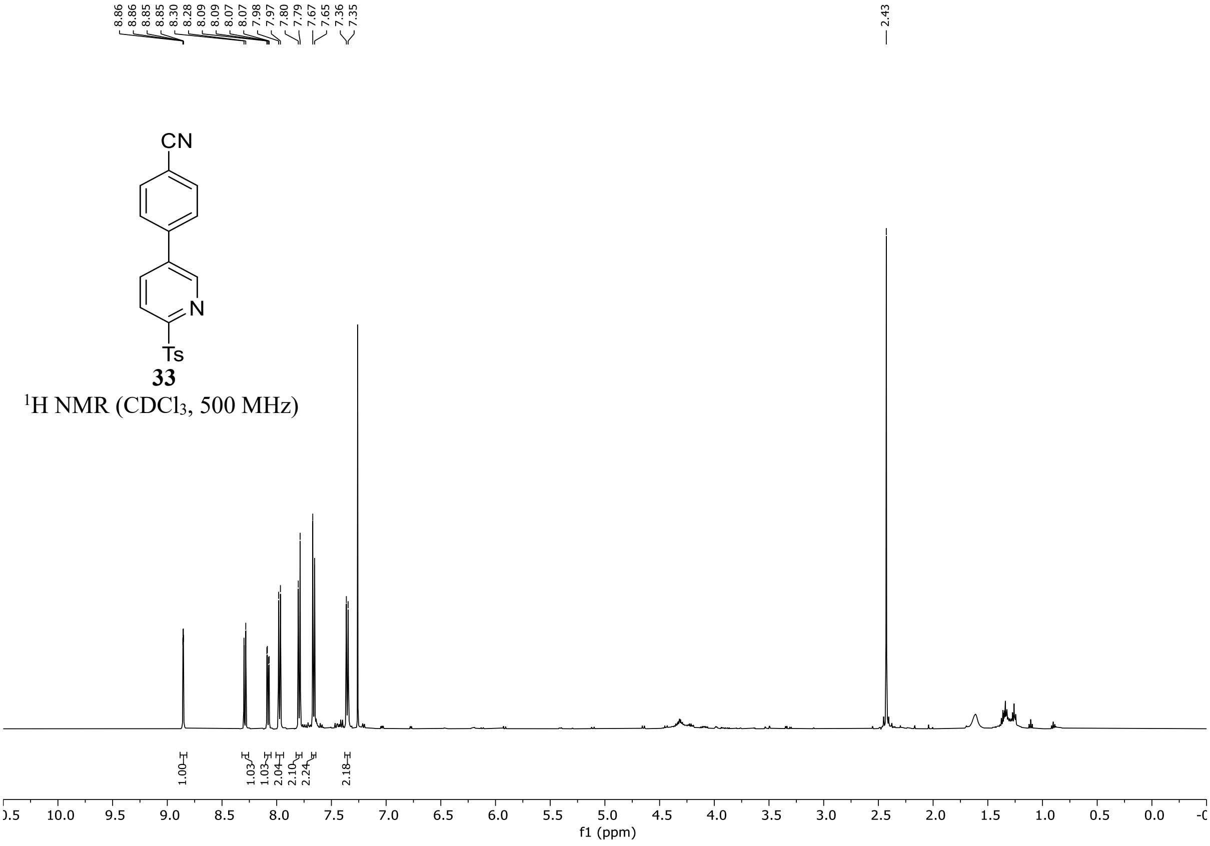
8.86 8.86 8.85 8.85 8.30 8.28 8.09 8.09 8.07 8.07 7.98 7.97 7.80 7.79 7.67 7.65 7.36 7.35
2.43
CN
N
Ts
33
^{1}H NMR (CDCl$_3$, 500 MHz)
1.00 1.03 1.03 2.04 2.10 2.24 2.18
f1 (ppm)

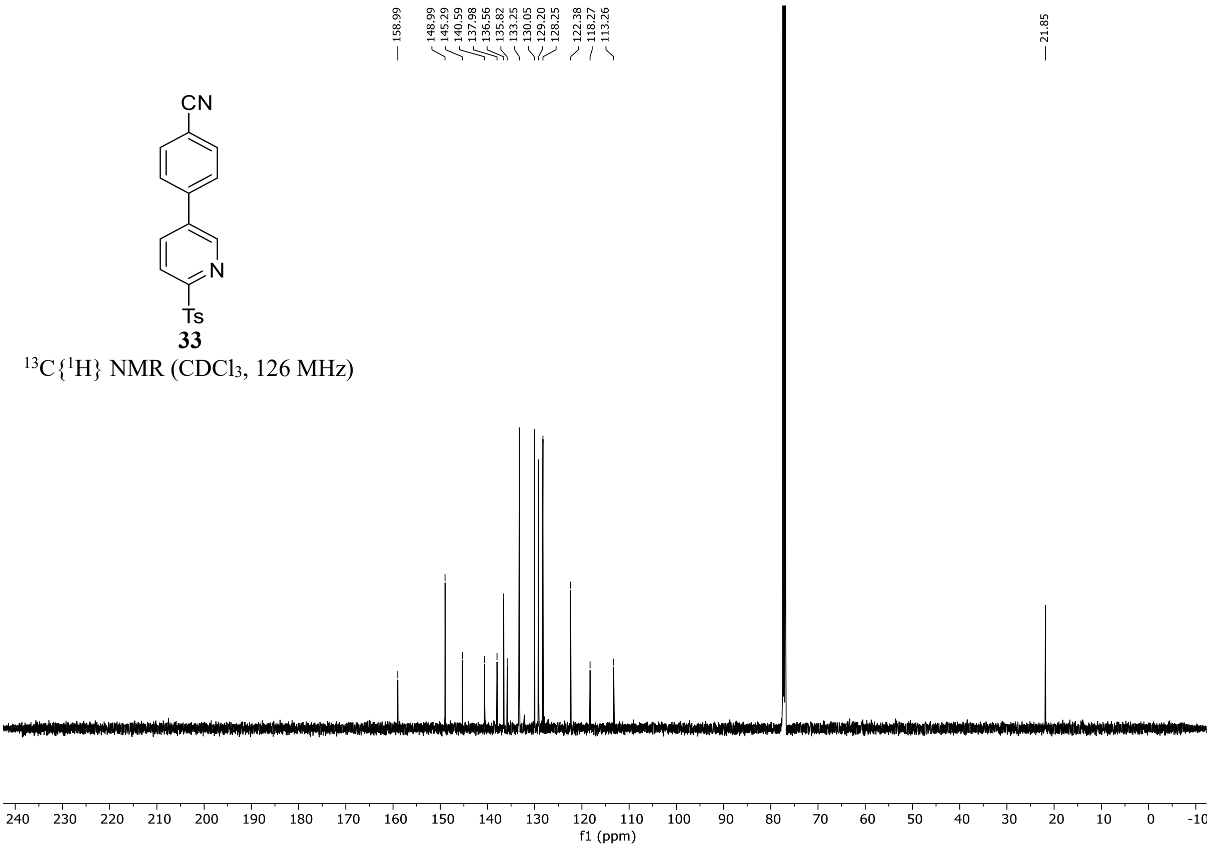
158.99
148.99 145.29 140.59 137.98 136.56 135.82 133.25 130.05 129.20 128.25 122.38 118.27 113.26
21.85
CN
N
Ts
33
^{13}C{^{1}H} NMR (CDCl$_3$, 126 MHz)
f1 (ppm)

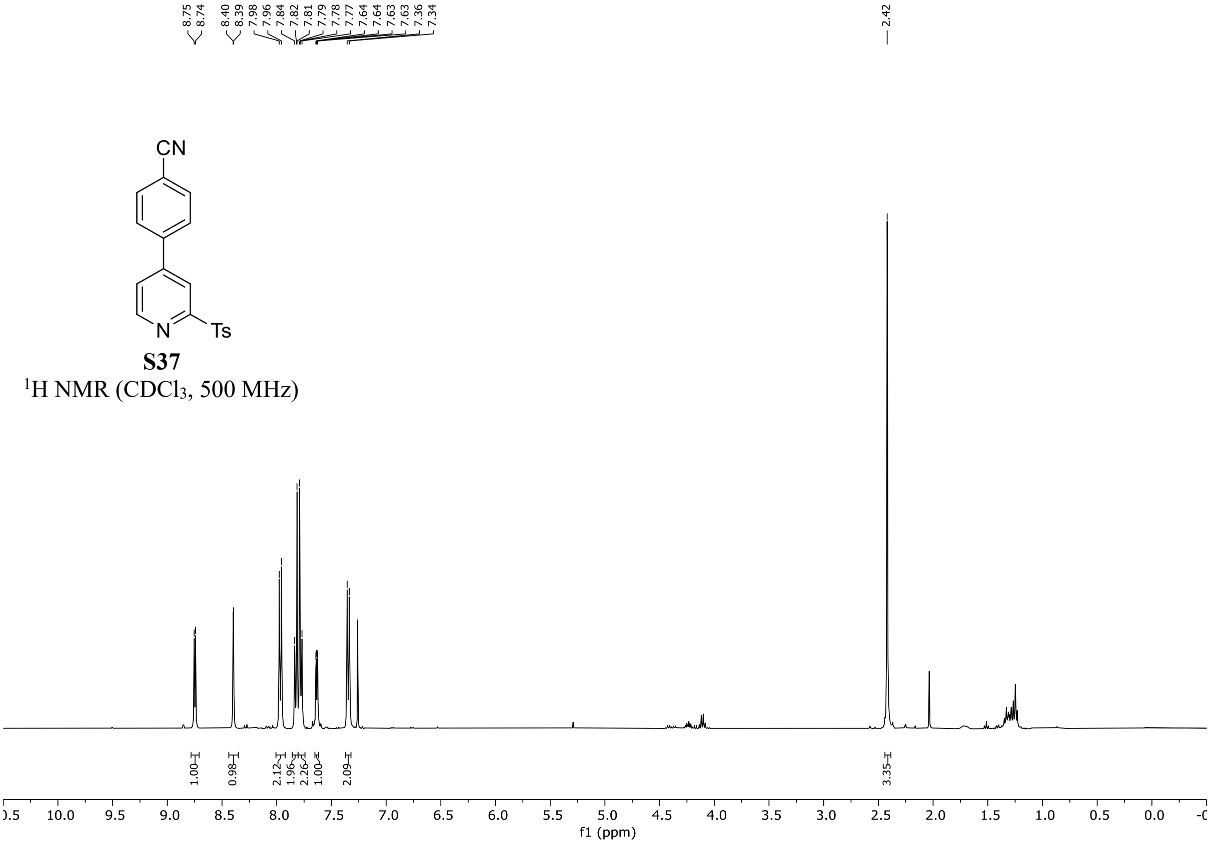
8.75
8.74
8.40
8.39
7.98
7.96
7.84
7.82
7.81
7.79
7.78
7.77
7.64
7.64
7.63
7.63
7.36
7.34
2.42
CN
N
Ts
S37
1H NMR (CDCl3, 500 MHz)
1.00
0.98
2.12
1.96
2.26
1.00
2.09
3.35
f1 (ppm)

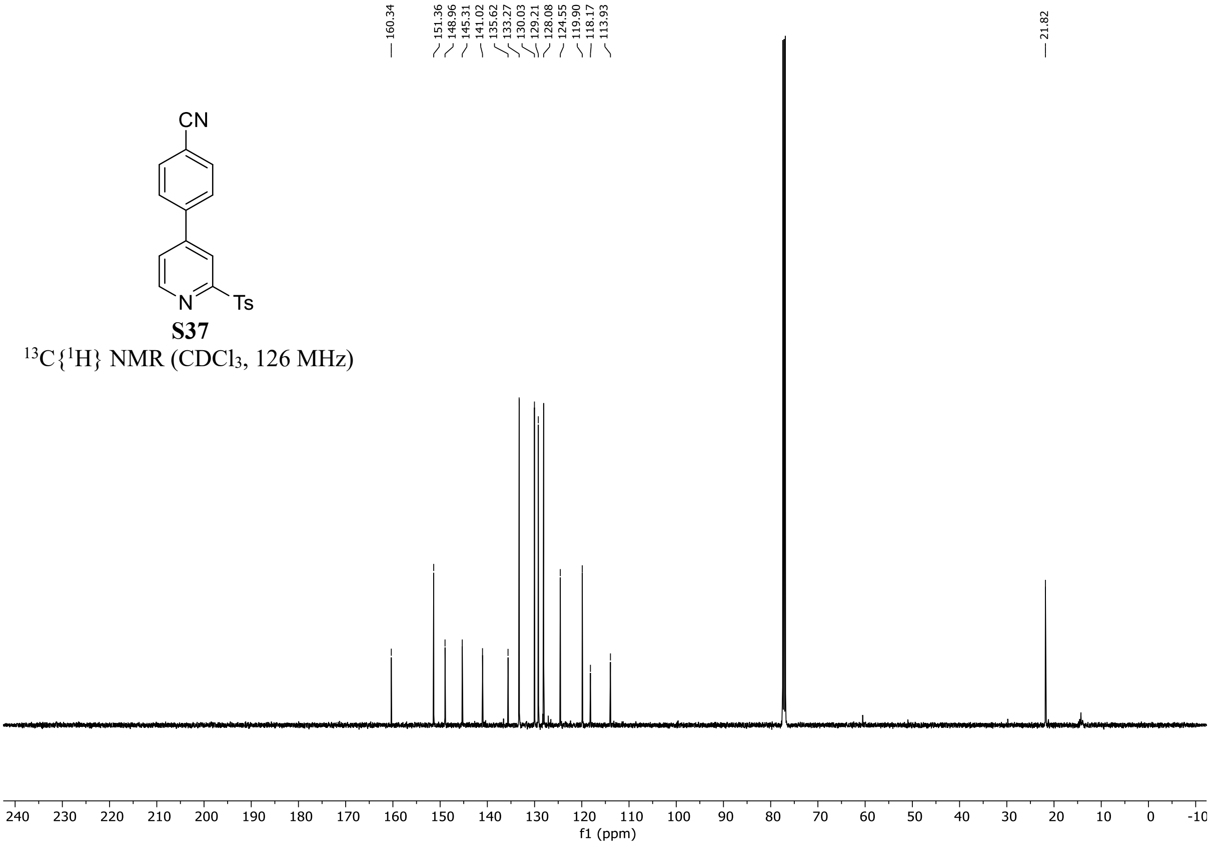
160.34
151.36
148.96
145.31
141.02
135.62
133.27
130.03
129.21
128.08
124.55
119.90
118.17
113.93
21.82
CN
N
Ts
S37
13C{1H} NMR (CDCl3, 126 MHz)
f1 (ppm)

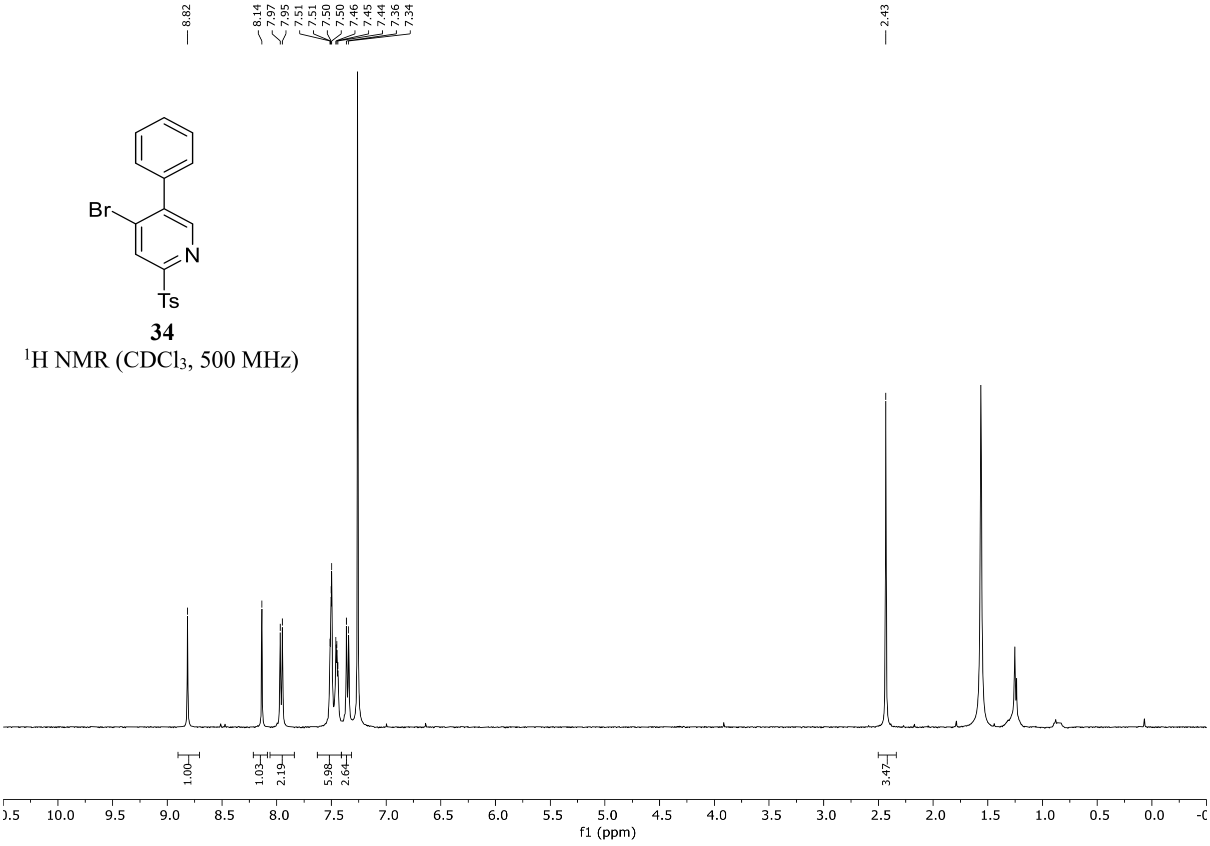

34

^{1}H NMR ($CDCl_3$, 500 MHz)

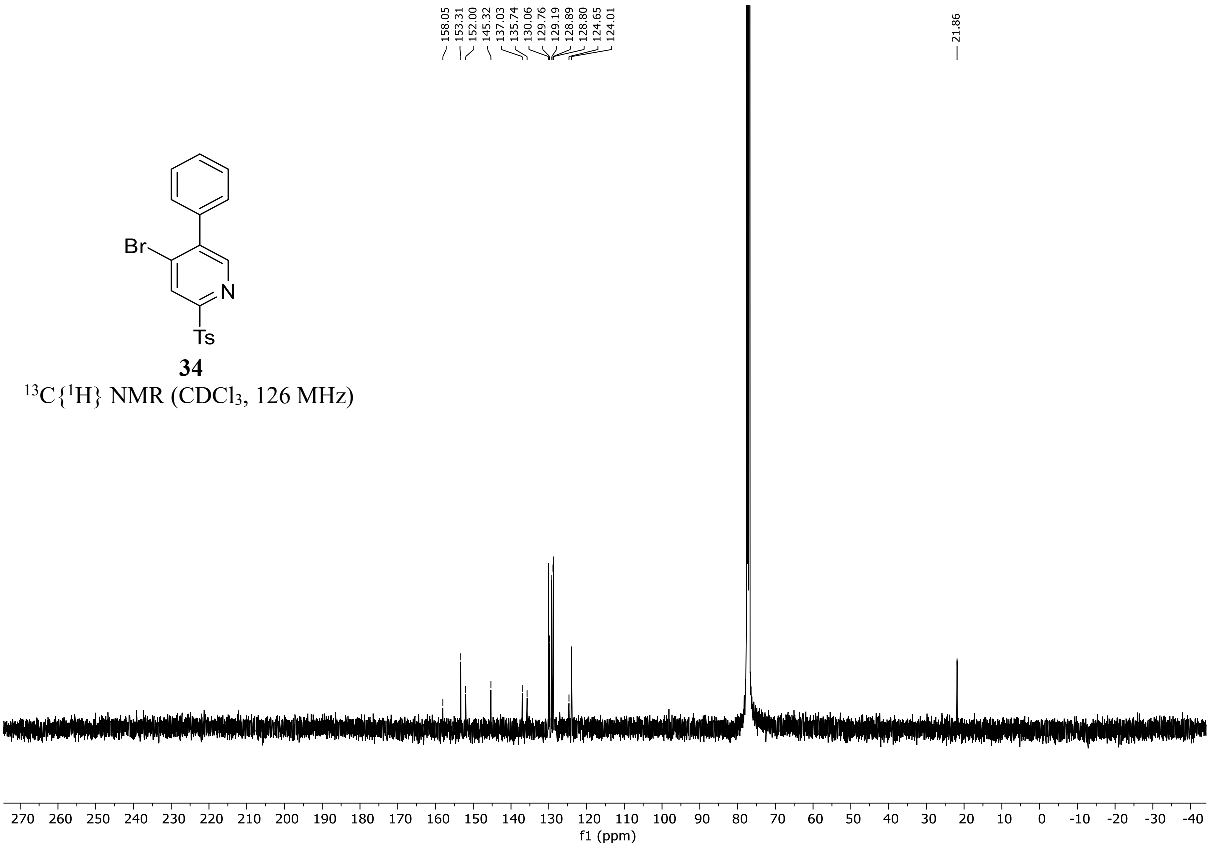

34

$^{13}C\{^{1}H\}$ NMR ($CDCl_3$, 126 MHz)

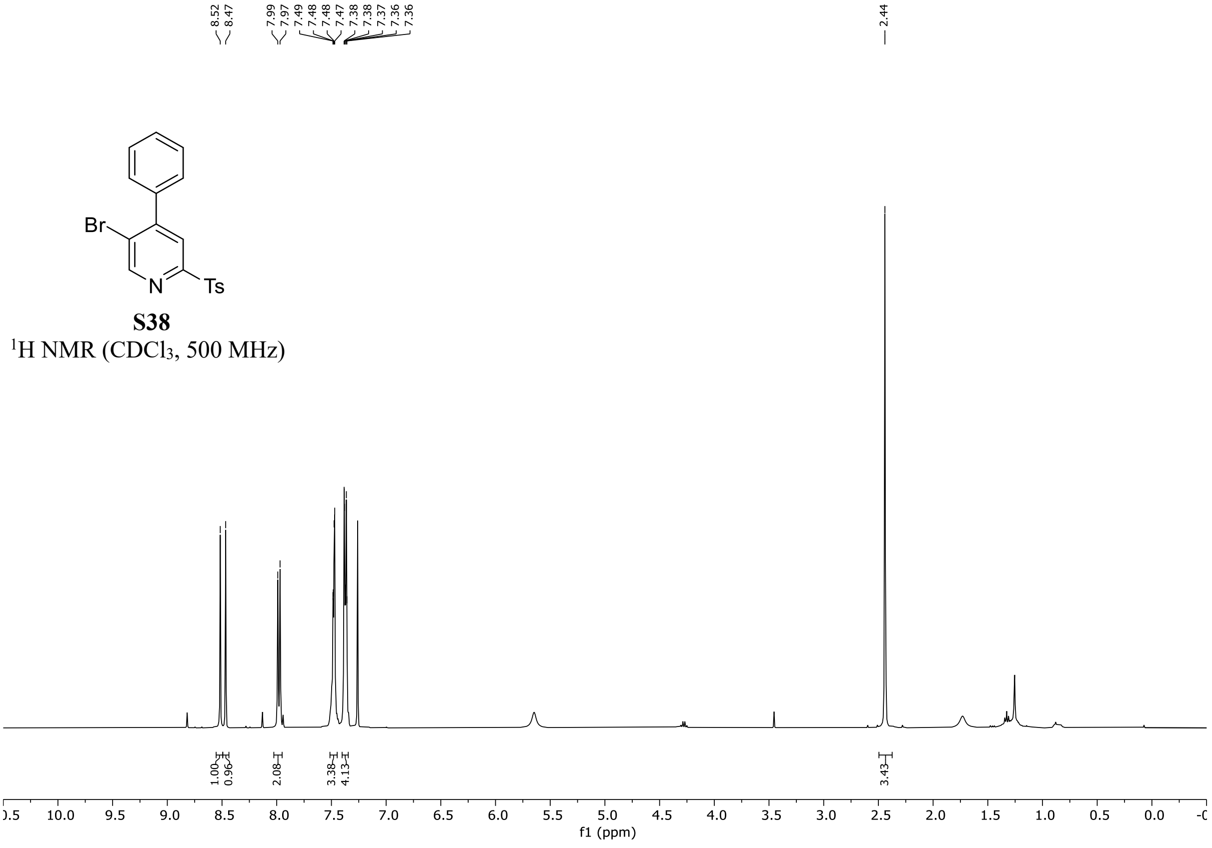
8.52
8.47
7.99
7.97
7.49
7.48
7.48
7.47
7.38
7.38
7.37
7.36
7.36
2.44
Br
N
Ts
S38
1H NMR (CDCl3, 500 MHz)
1.00
0.96
2.08
3.38
4.13
3.43
f1 (ppm)

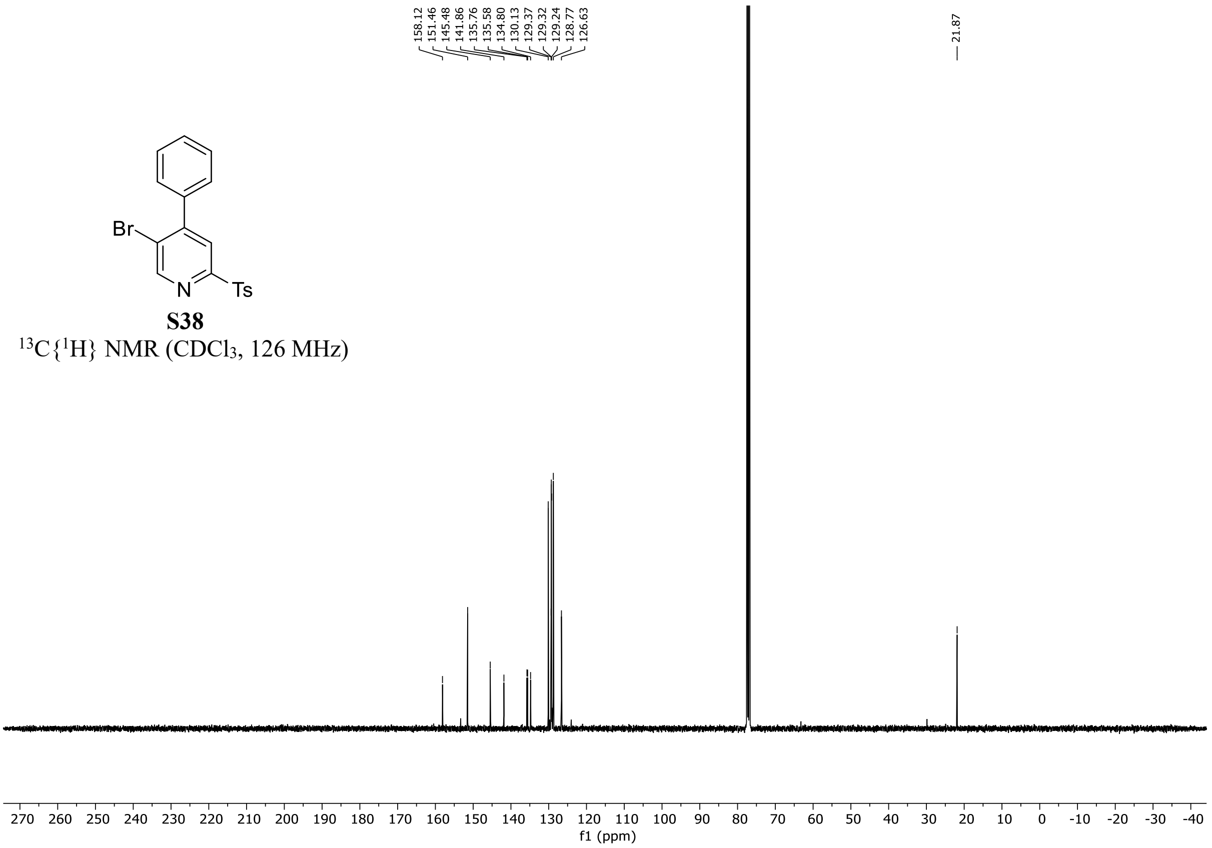
158.12
151.46
145.48
141.86
135.76
135.58
134.80
130.13
129.37
129.32
129.24
128.77
126.63
21.87
Br
N
Ts
S38
13C{1H} NMR (CDCl3, 126 MHz)
f1 (ppm)

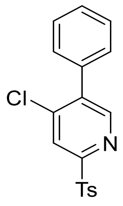

35

^{1}H NMR ($CDCl_3$, 500 MHz)

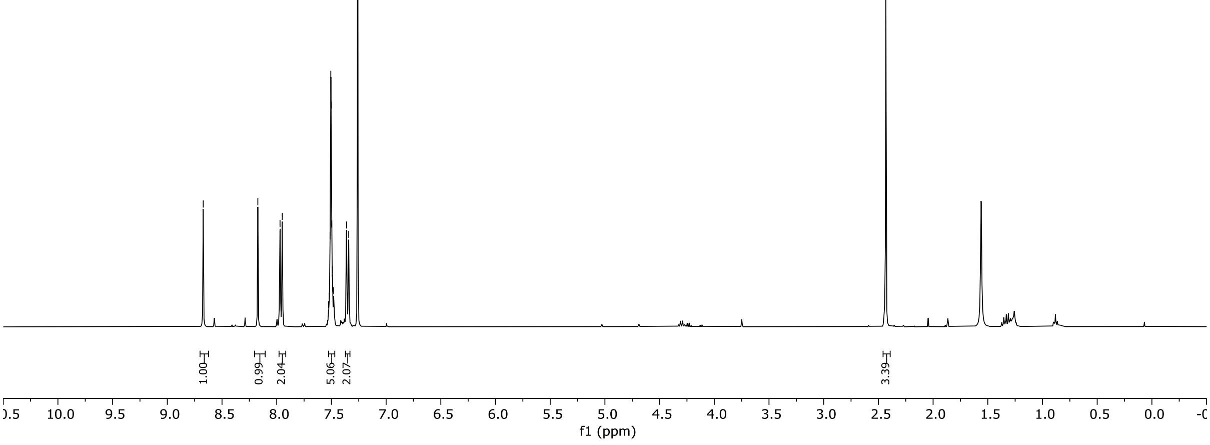

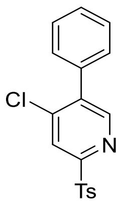

35

$^{13}C\{^1H\}$ NMR ($CDCl_3$, 126 MHz)

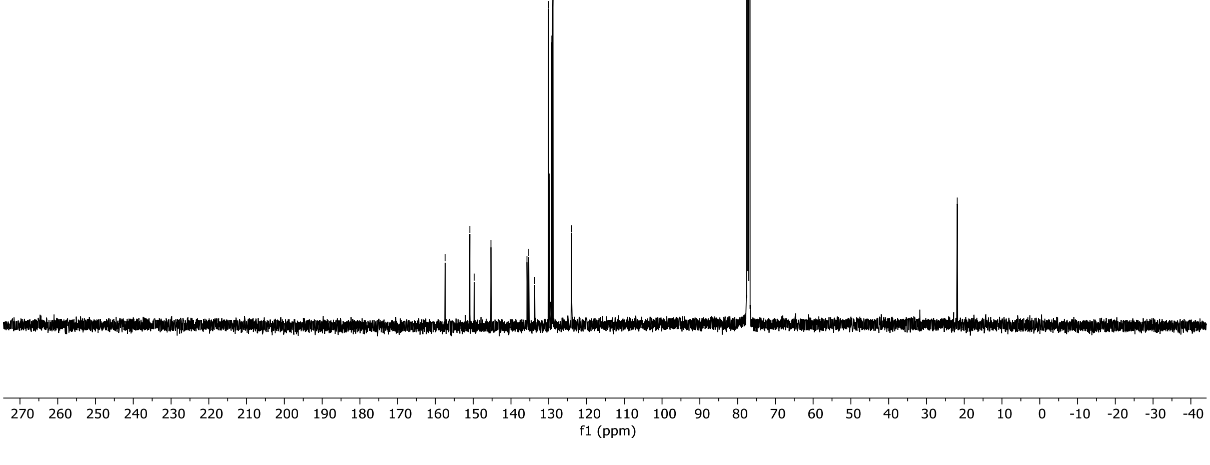

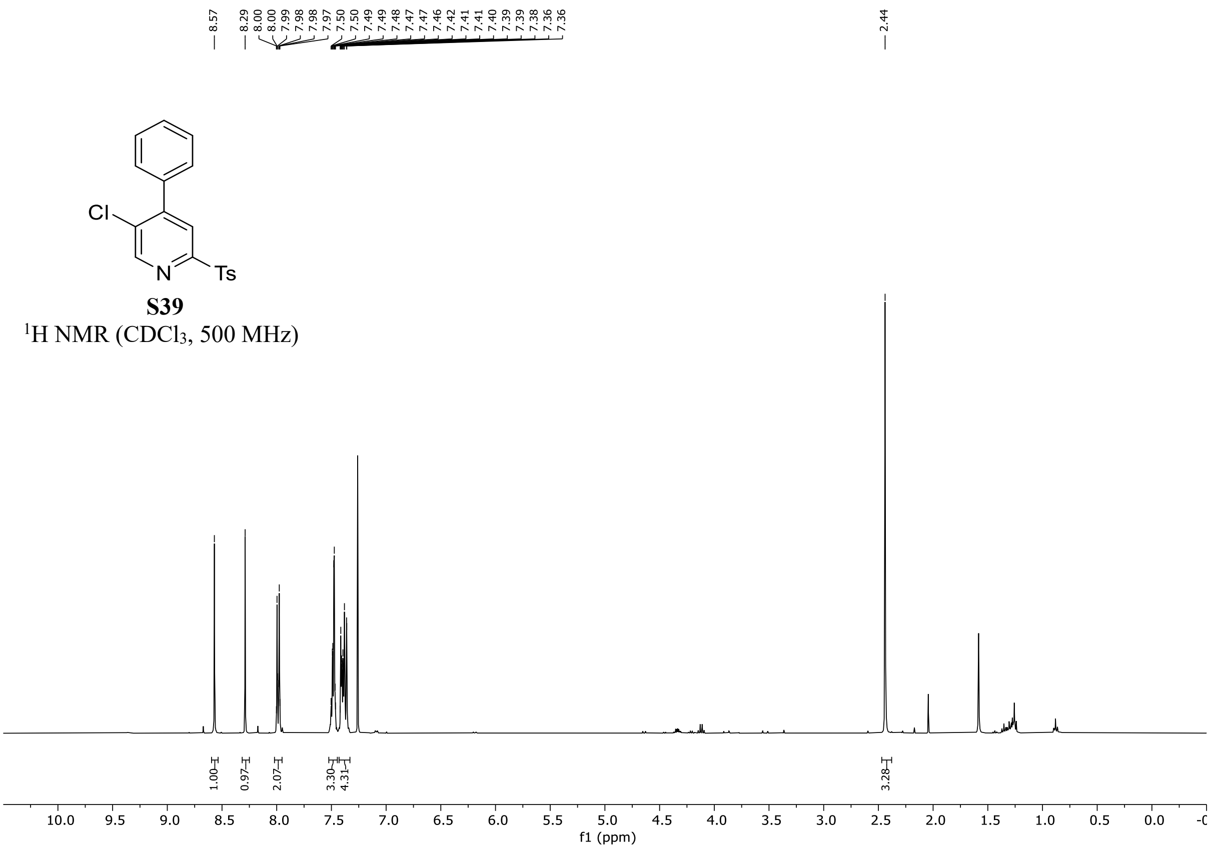

S39

^{1}H NMR ($CDCl_3$, 500 MHz)

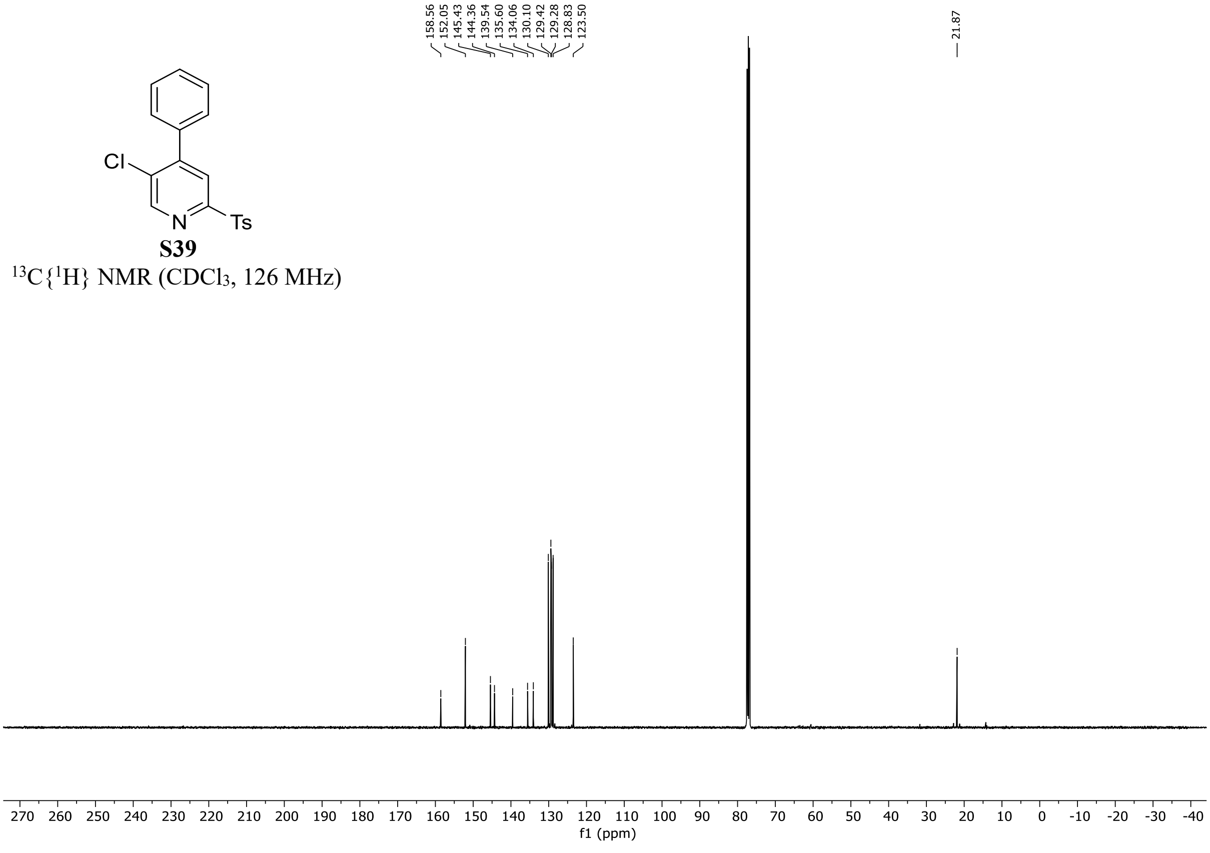

S39

$^{13}C\{^{1}H\}$ NMR ($CDCl_3$, 126 MHz)

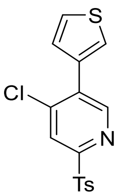

36
^{1}H NMR ($CDCl_3$, 500 MHz)

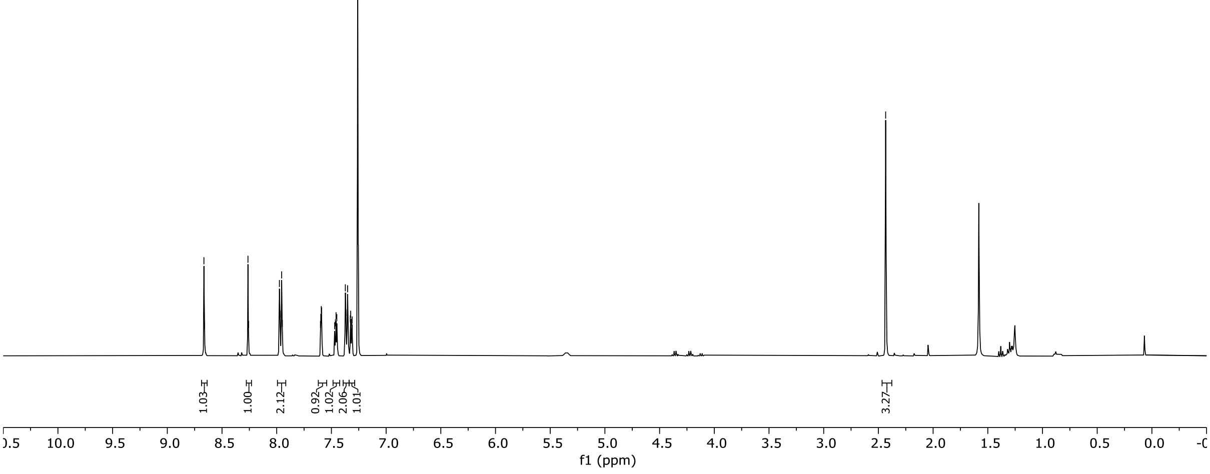

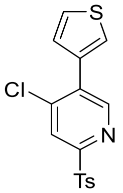

36
$^{13}C\{^{1}H\}$ NMR ($CDCl_3$, 126 MHz)

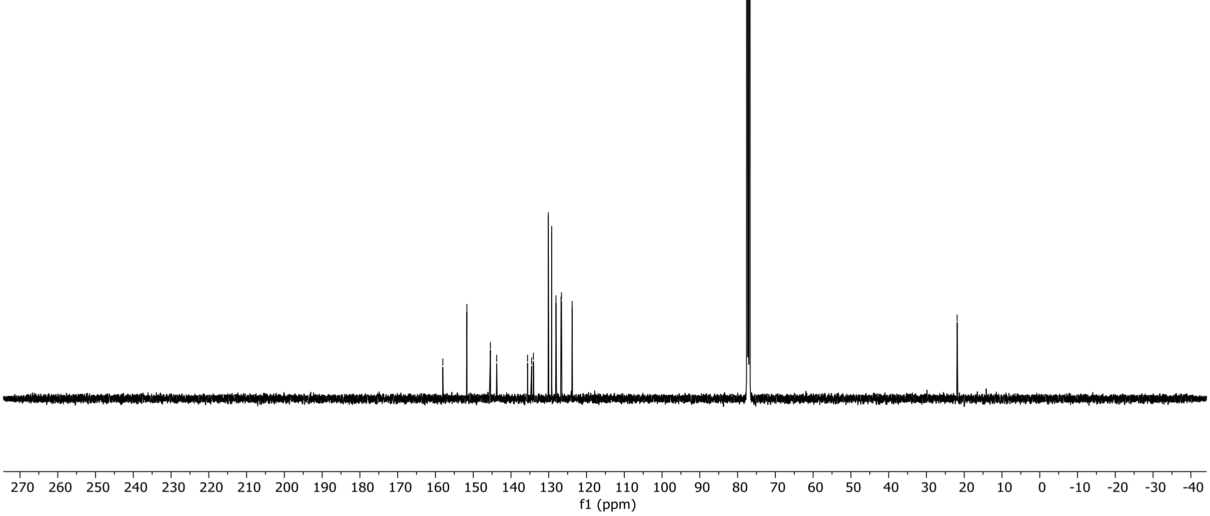

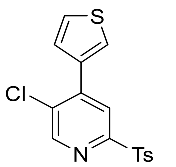

S40

^{1}H NMR ($CDCl_3$, 500 MHz)

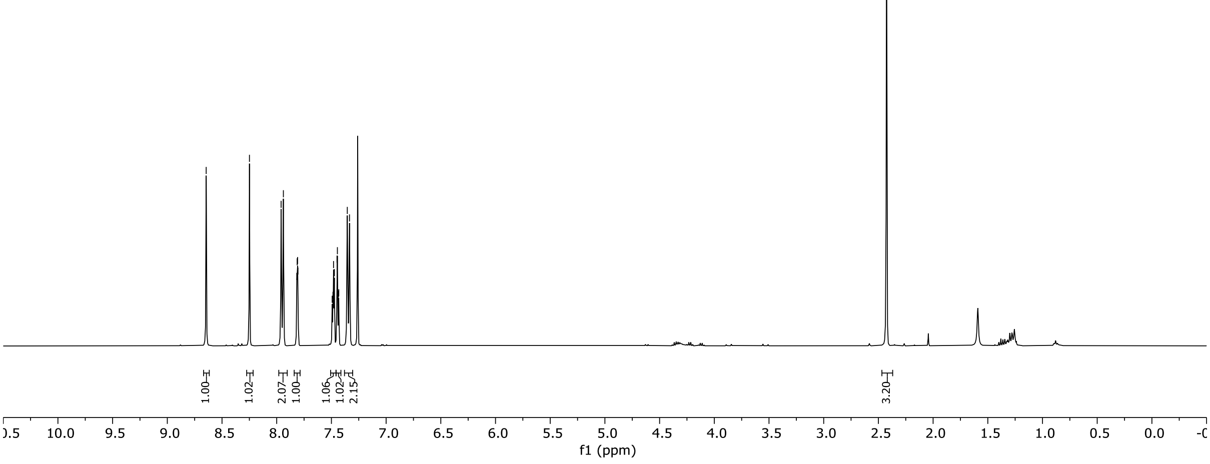

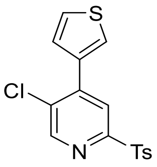

S40

^{13}C{^{1}H} NMR ($CDCl_3$, 126 MHz)

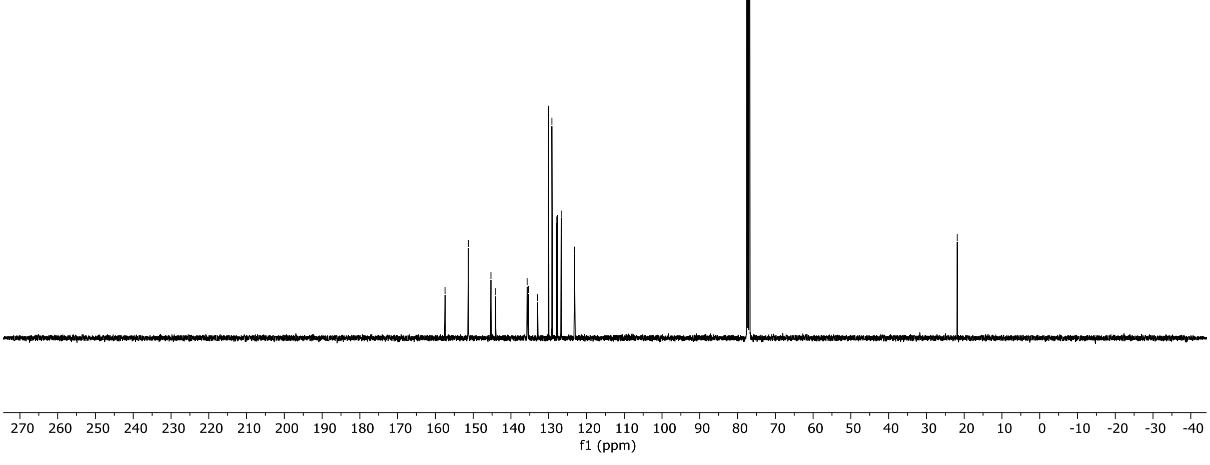

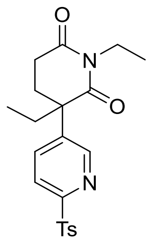

38

^{1}H NMR ($CDCl_3$, 500 MHz)

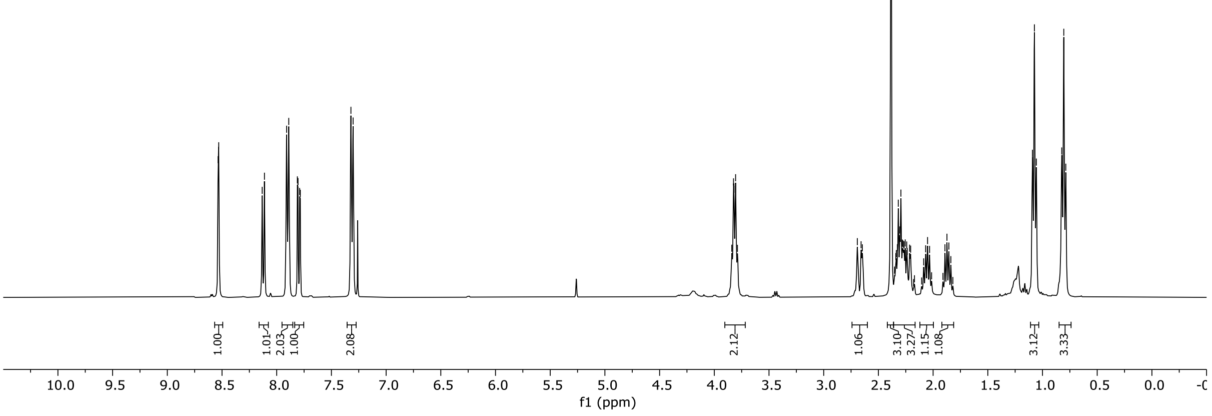

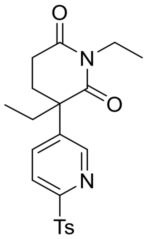

38

$^{13}C\{^1H\}$ NMR ($CDCl_3$, 126 MHz)

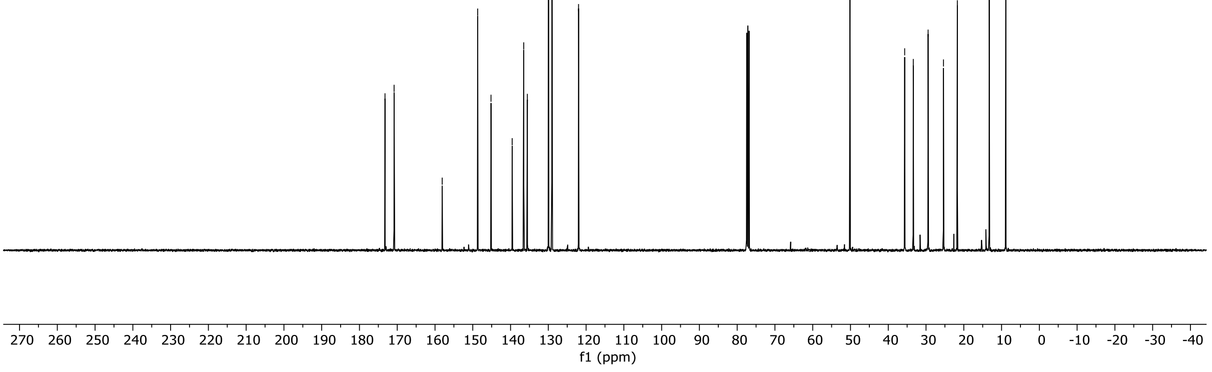

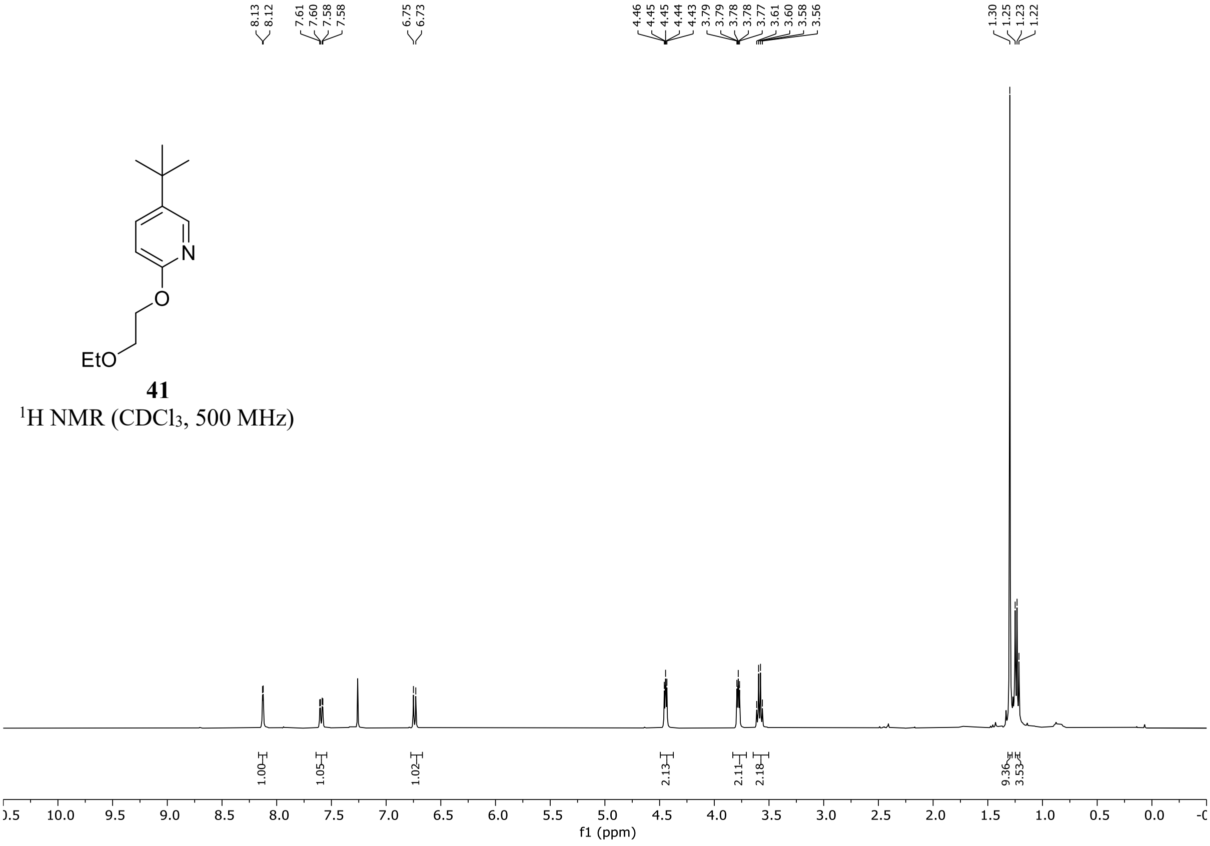

41

^{1}H NMR ($CDCl_3$, 500 MHz)

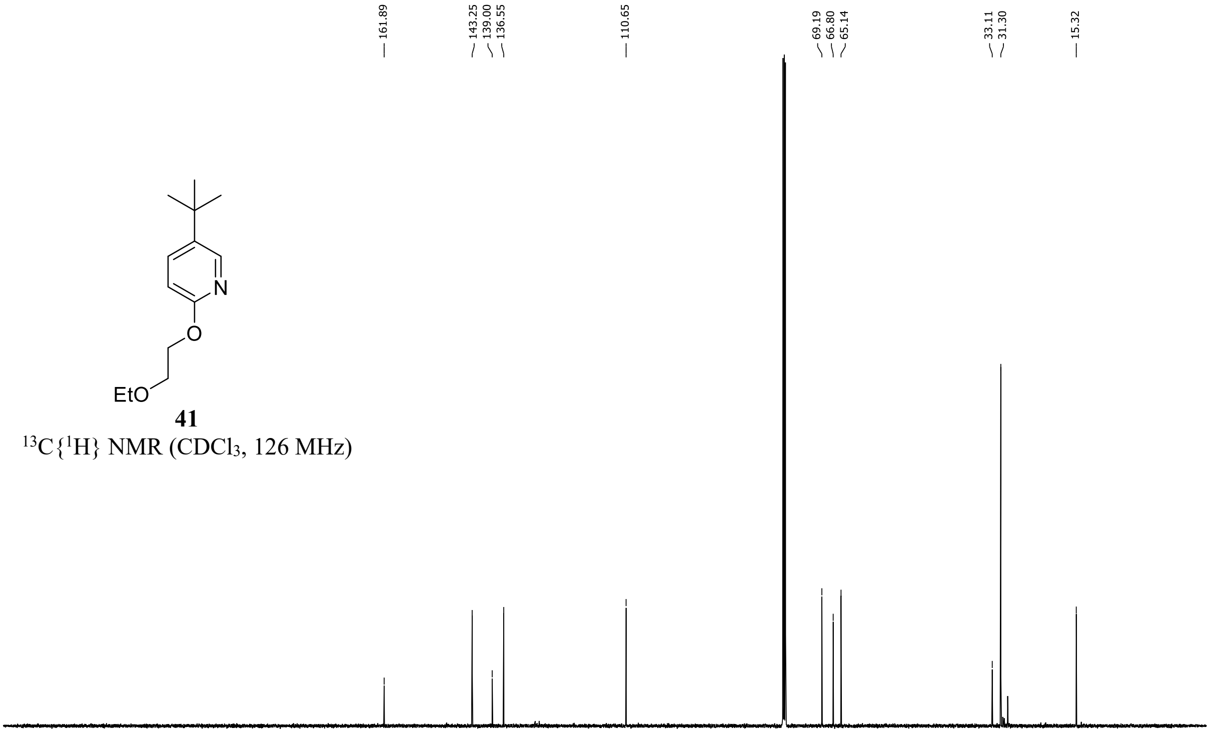

41

$^{13}C\{^1H\}$ NMR ($CDCl_3$, 126 MHz)

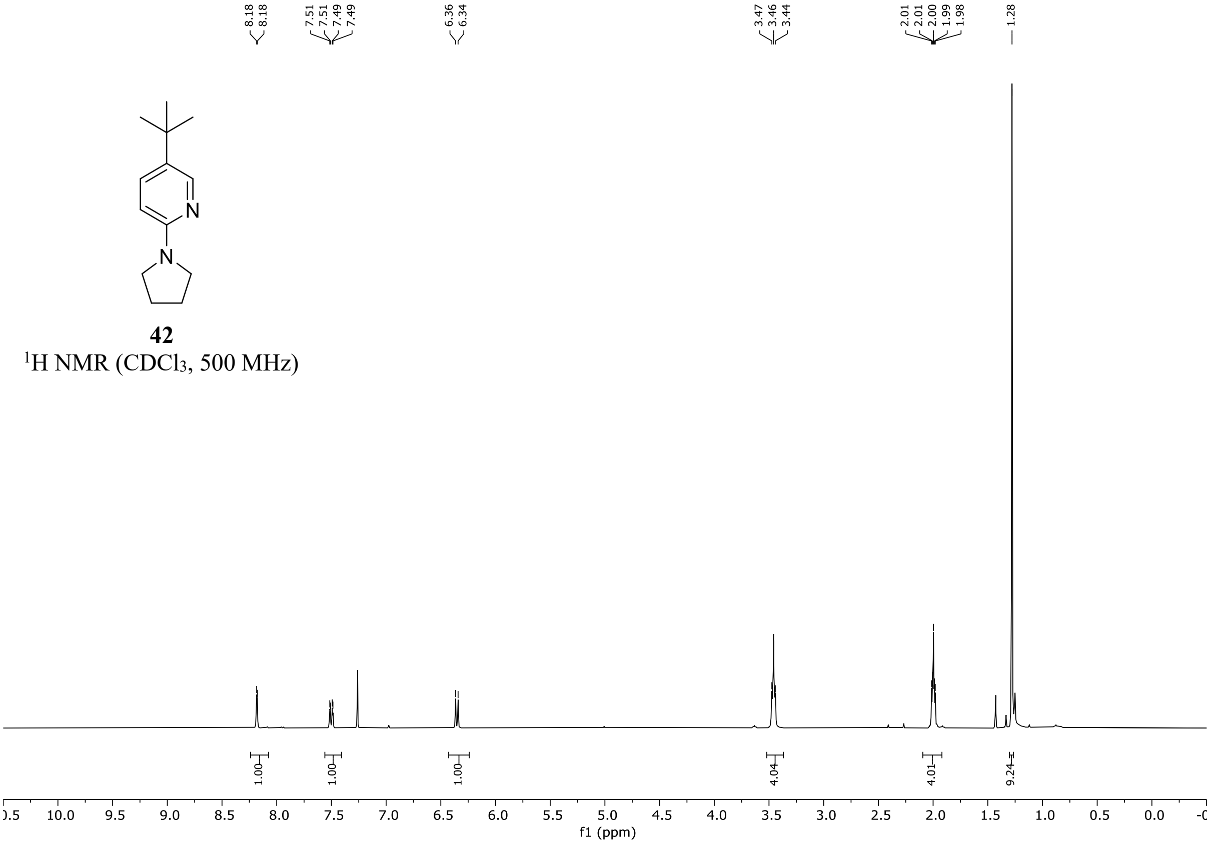

42

^1H NMR ($CDCl_3$, 500 MHz)

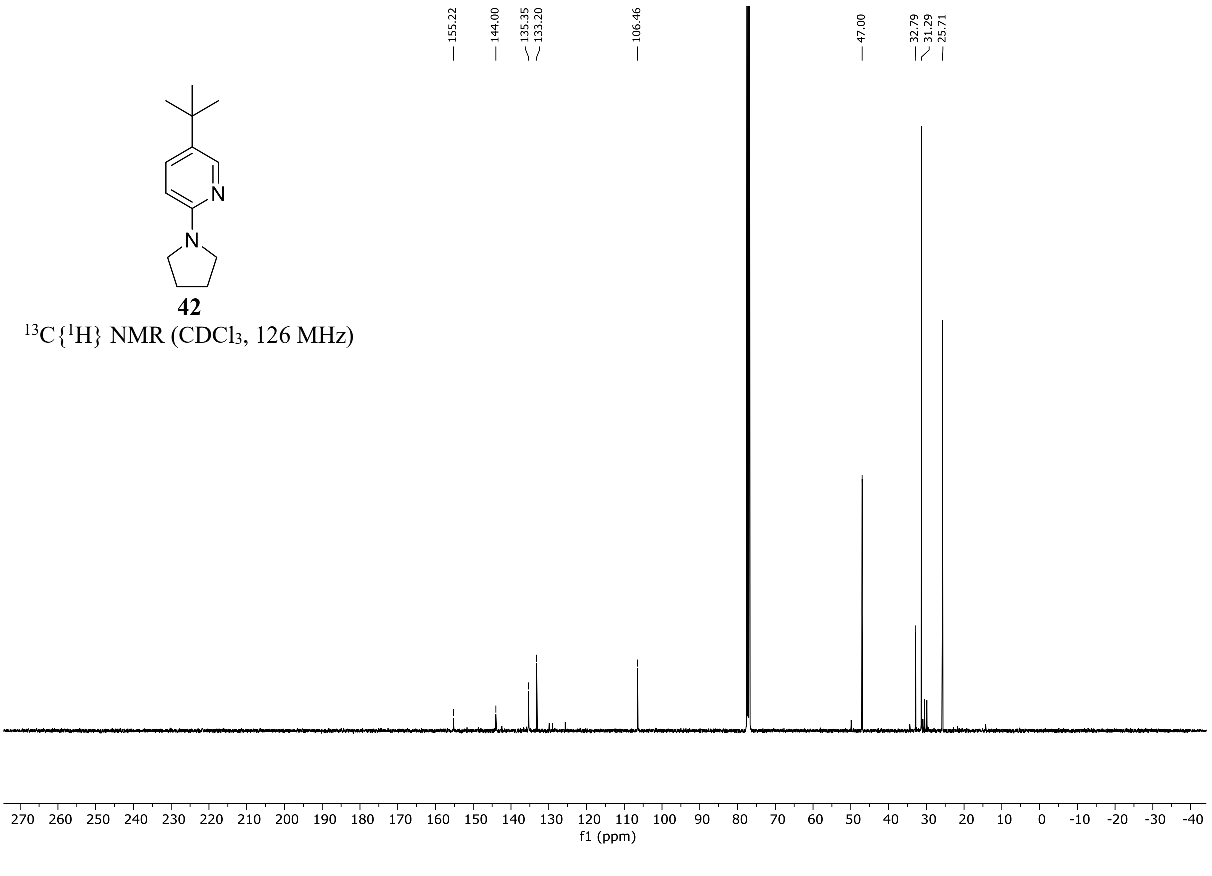

42

$^{13}C\{^1H\}$ NMR ($CDCl_3$, 126 MHz)

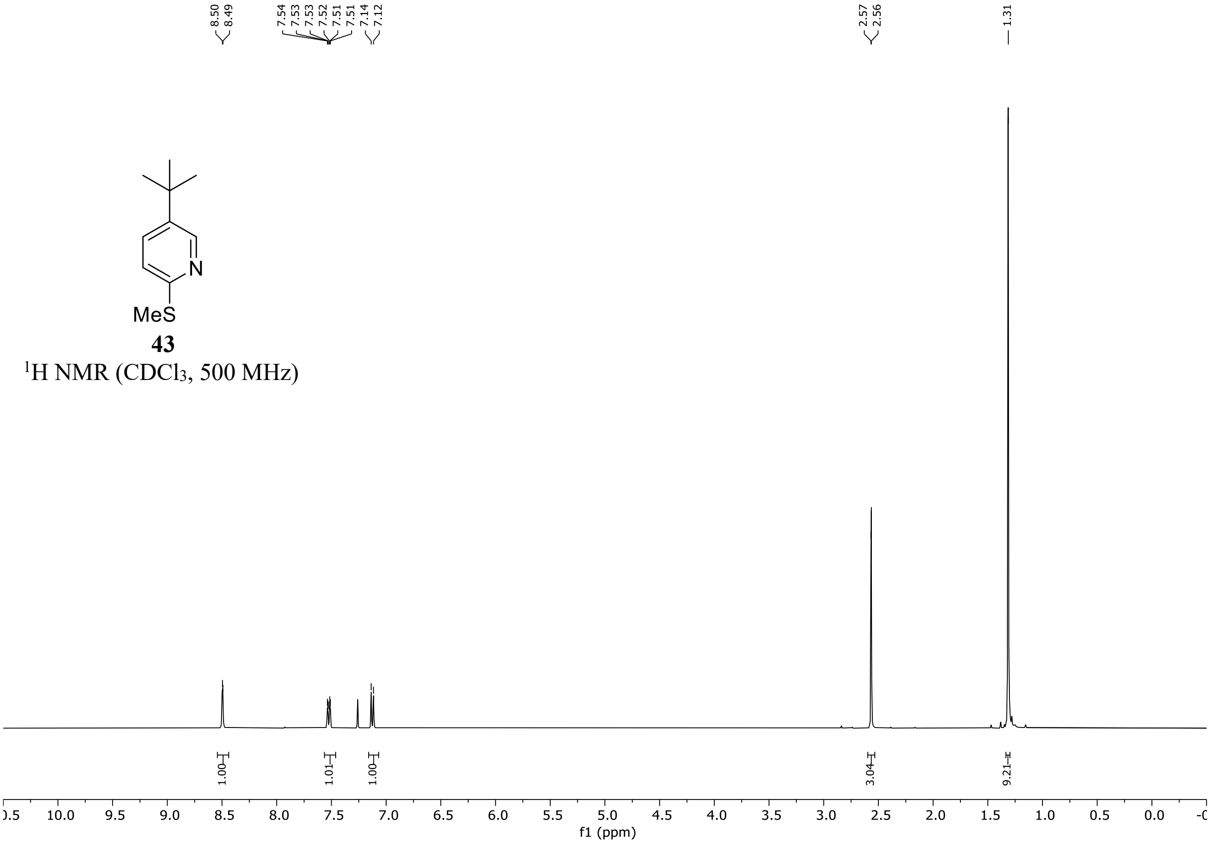

43

^{1}H NMR ($CDCl_3$, 500 MHz)

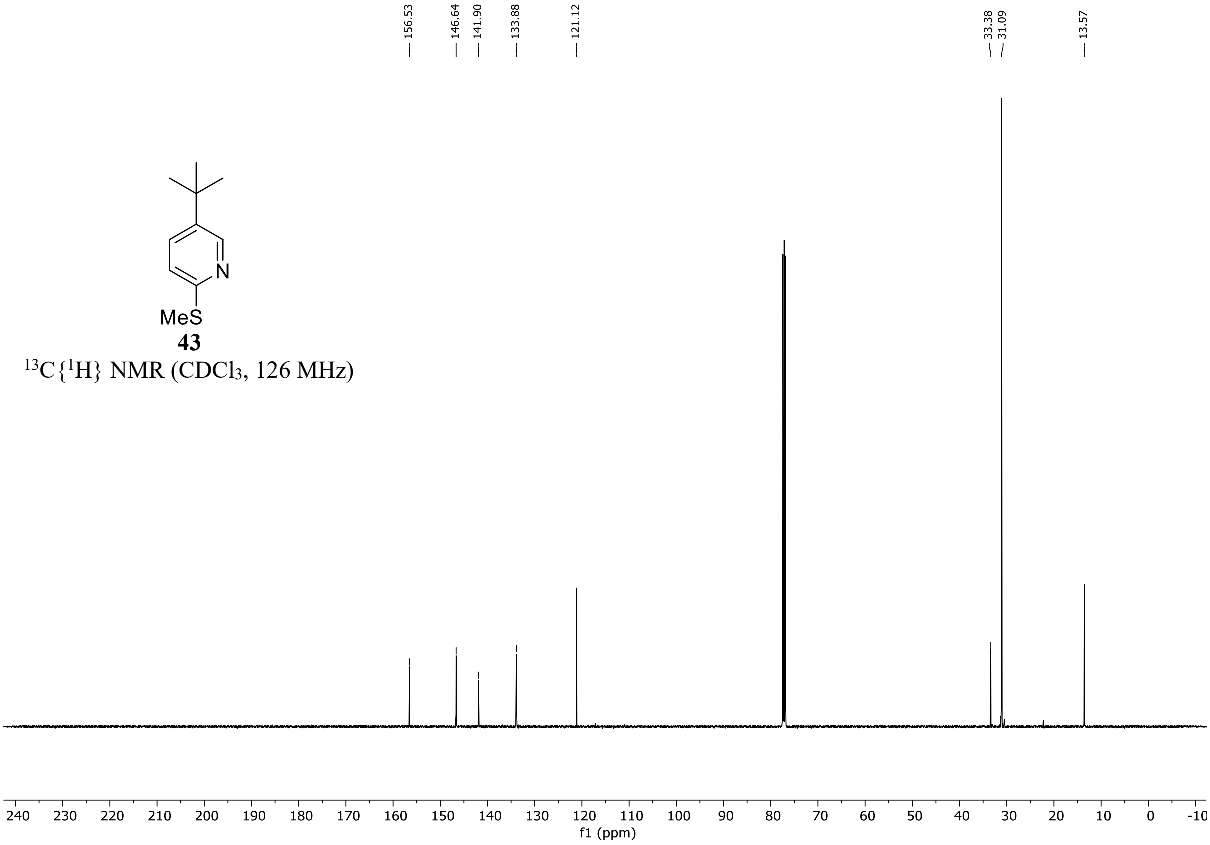

43

^{13}C{^{1}H} NMR ($CDCl_3$, 126 MHz)

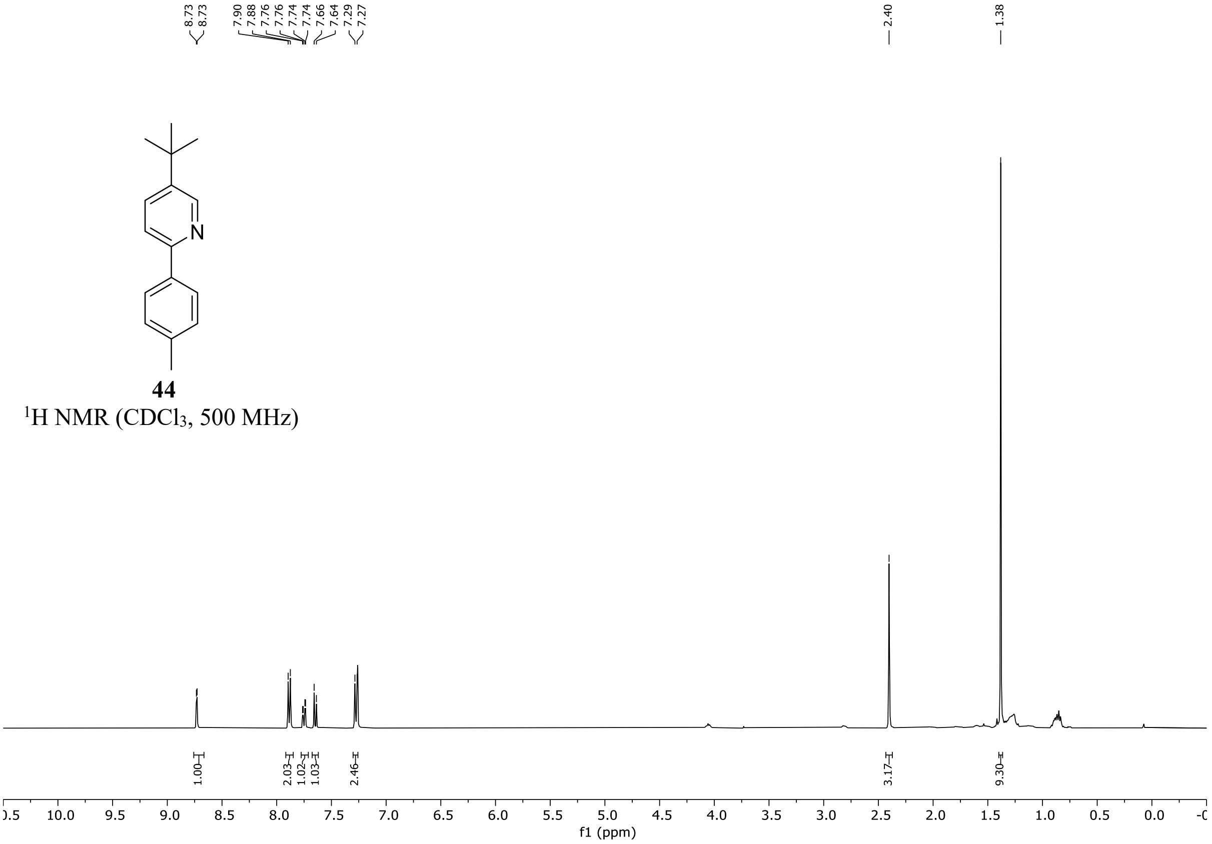

44
^{1}H NMR ($CDCl_3$, 500 MHz)

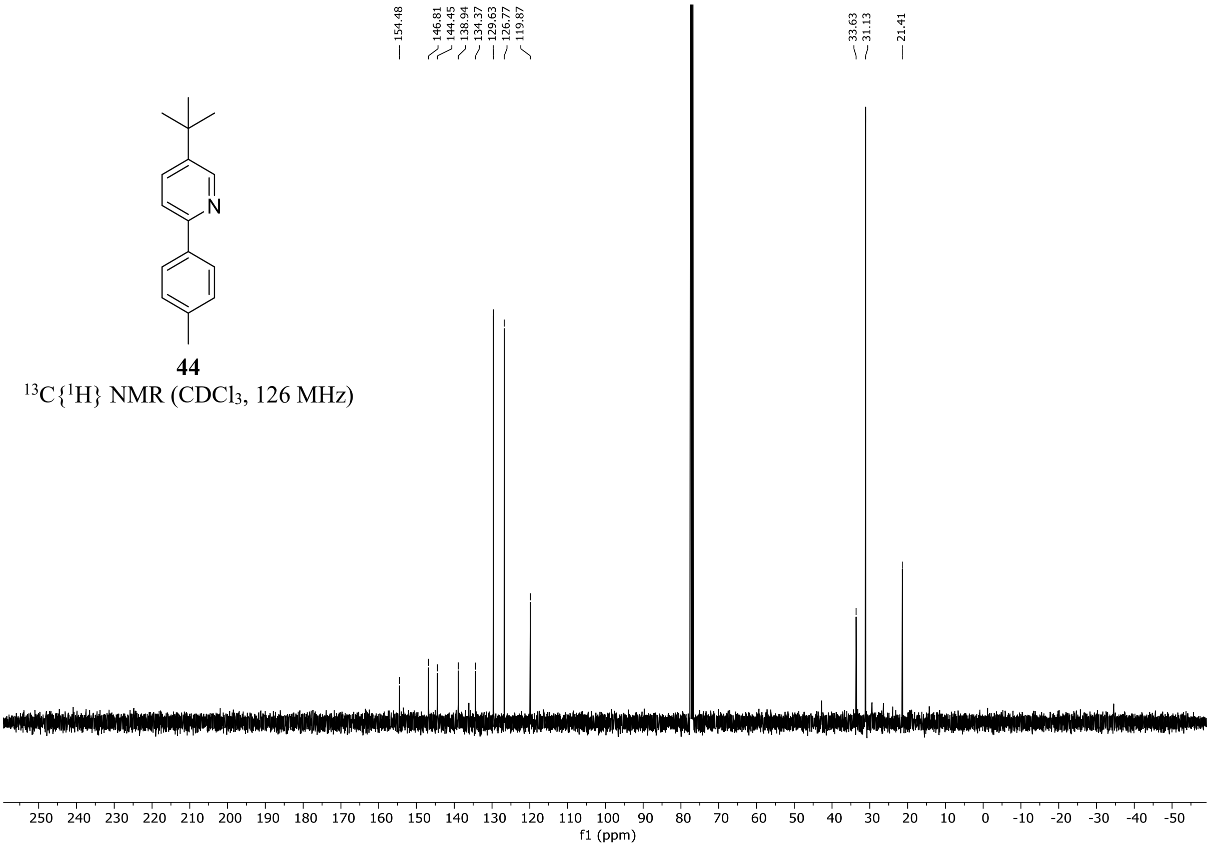

44
$^{13}C\{^{1}H\}$ NMR ($CDCl_3$, 126 MHz)

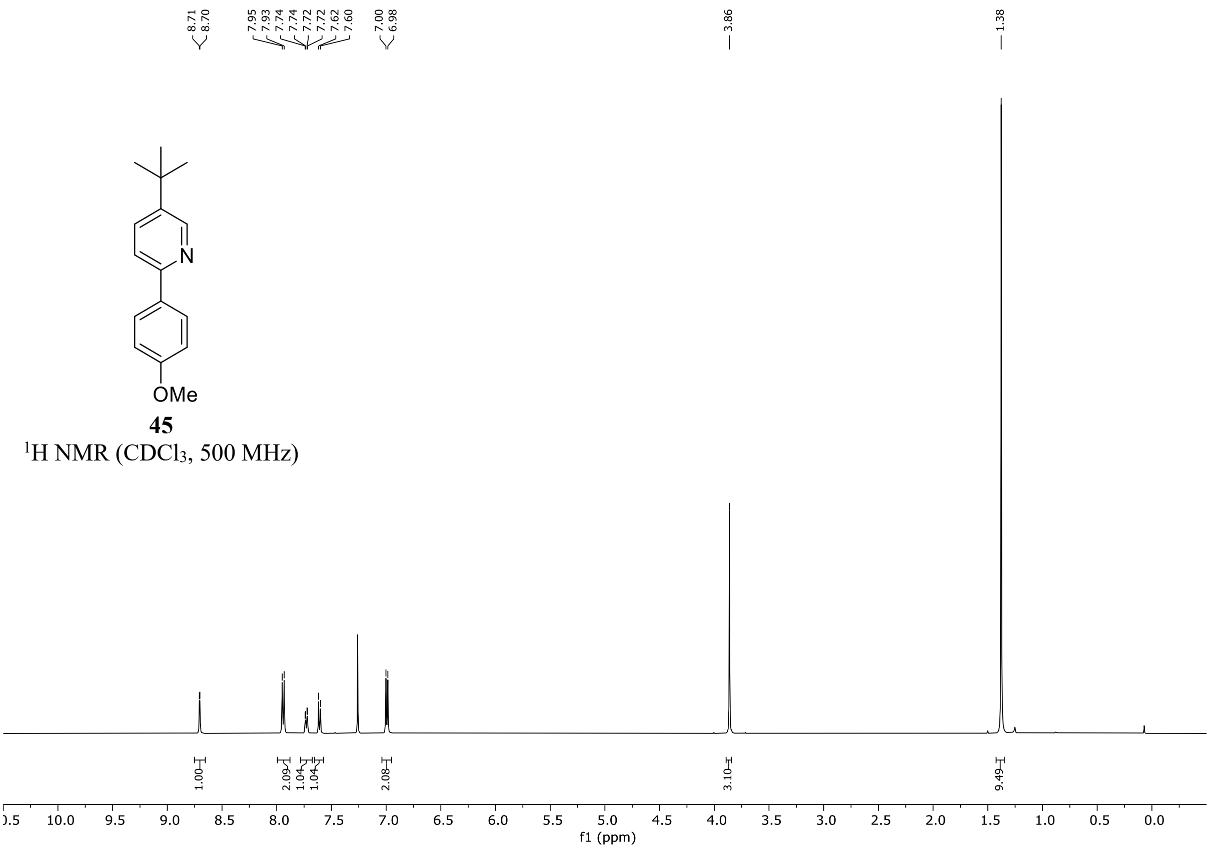
8.71
8.70
7.95
7.93
7.74
7.74
7.72
7.72
7.62
7.60
7.00
6.98
3.86
1.38
N
OMe
45
1H NMR (CDCl3, 500 MHz)
1.00
2.09
1.04
1.04
2.08
3.10
9.49
f1 (ppm)

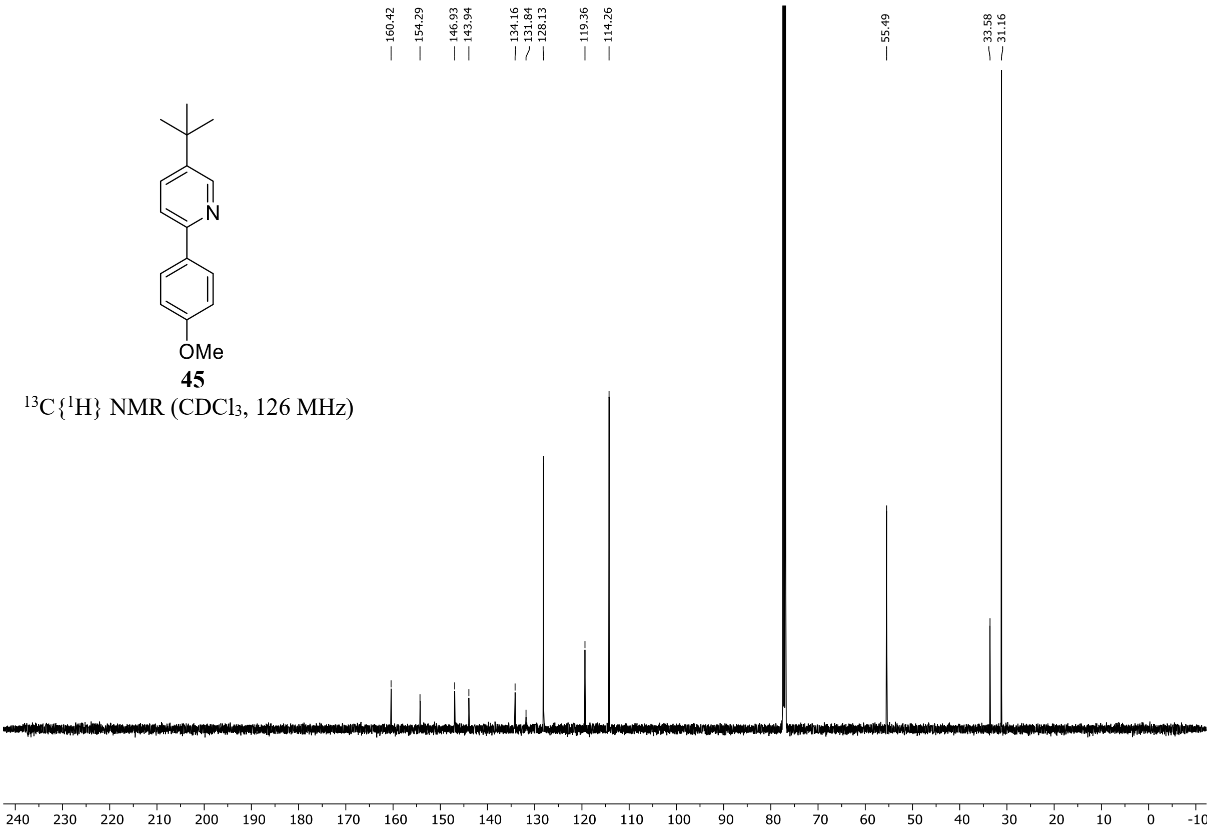
160.42
154.29
146.93
143.94
134.16
131.84
128.13
119.36
114.26
55.49
33.58
31.16
N
OMe
45
13C{1H} NMR (CDCl3, 126 MHz)
f1 (ppm)

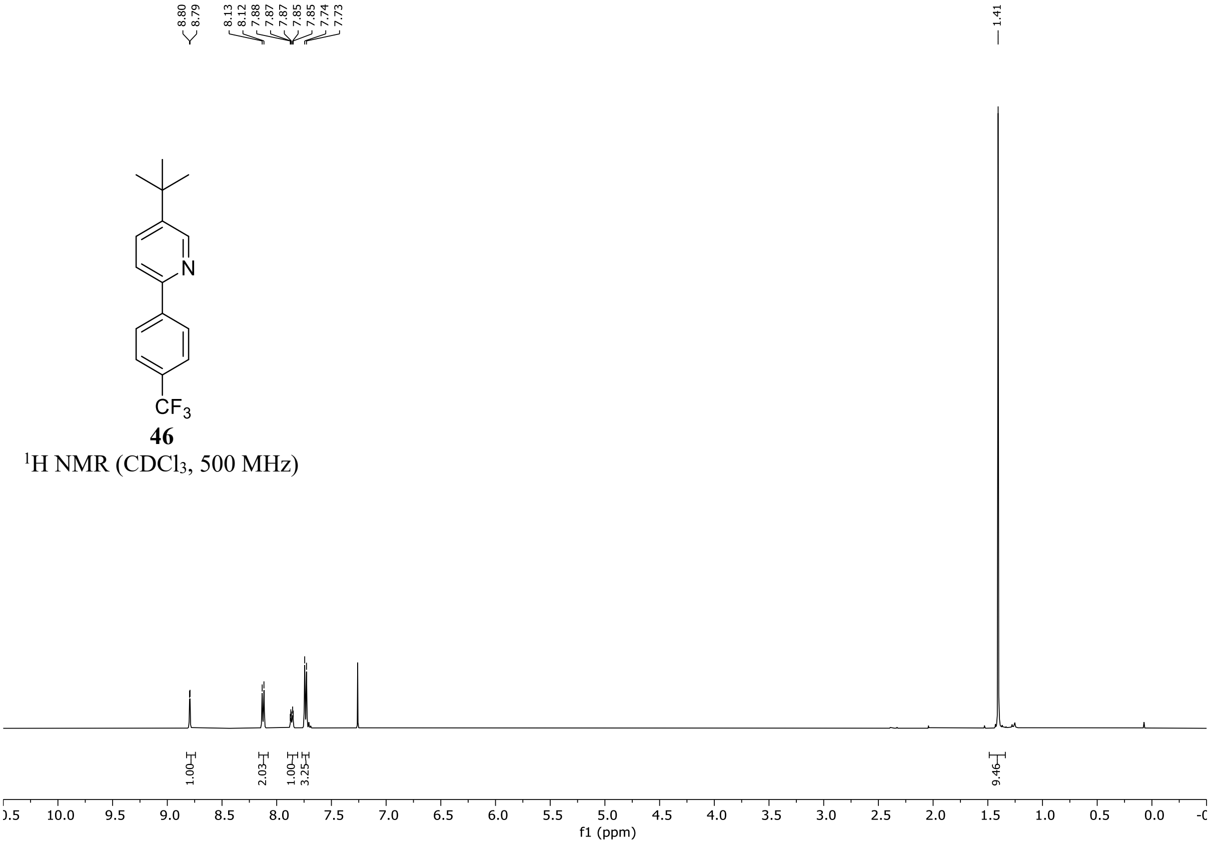
8.80
8.79
8.13
8.12
7.88
7.87
7.87
7.85
7.85
7.74
7.73
1.41
CF3
46
1H NMR (CDCl3, 500 MHz)
1.00
2.03
1.00
3.25
9.46
f1 (ppm)

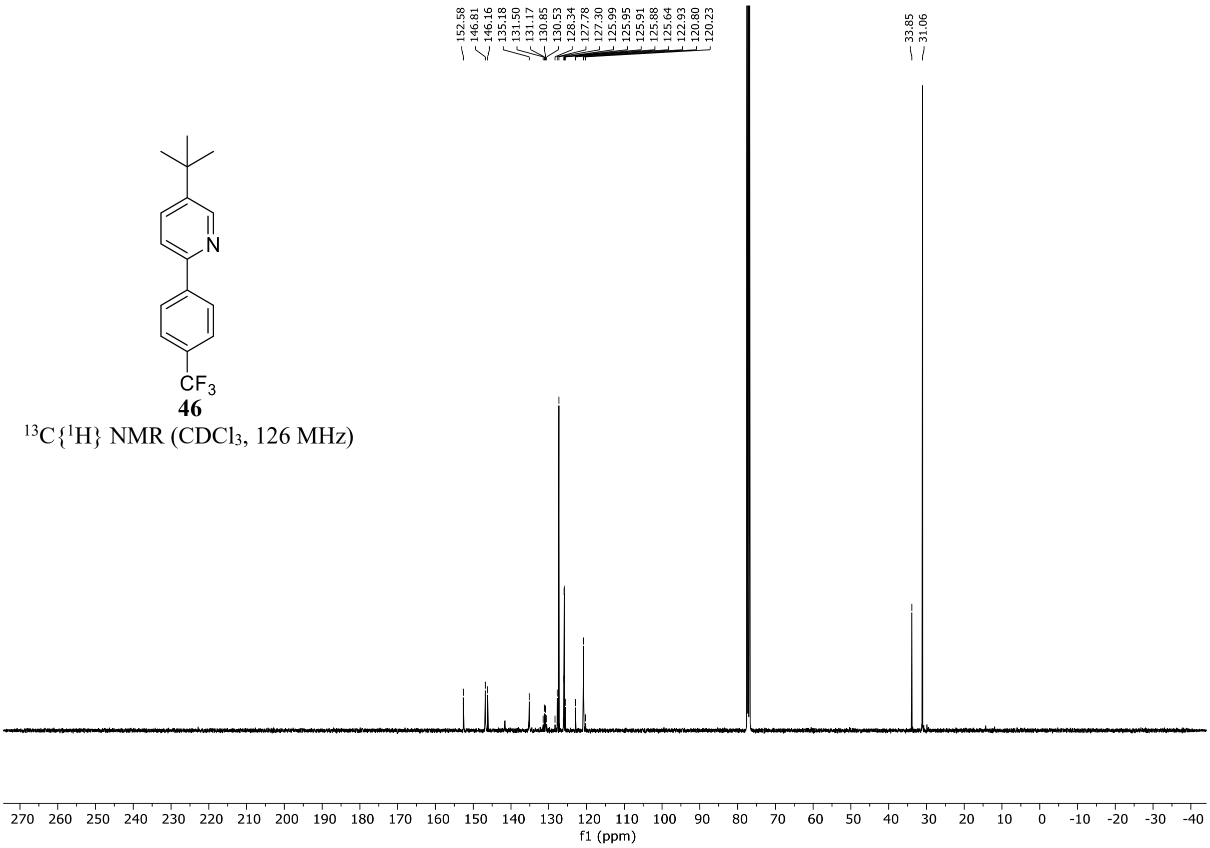
152.58
146.81
146.16
135.18
131.50
131.17
130.85
130.53
128.34
127.78
127.30
125.99
125.95
125.91
125.88
125.64
122.93
120.80
120.23
33.85
31.06
CF3
46
13C{1H} NMR (CDCl3, 126 MHz)
f1 (ppm)

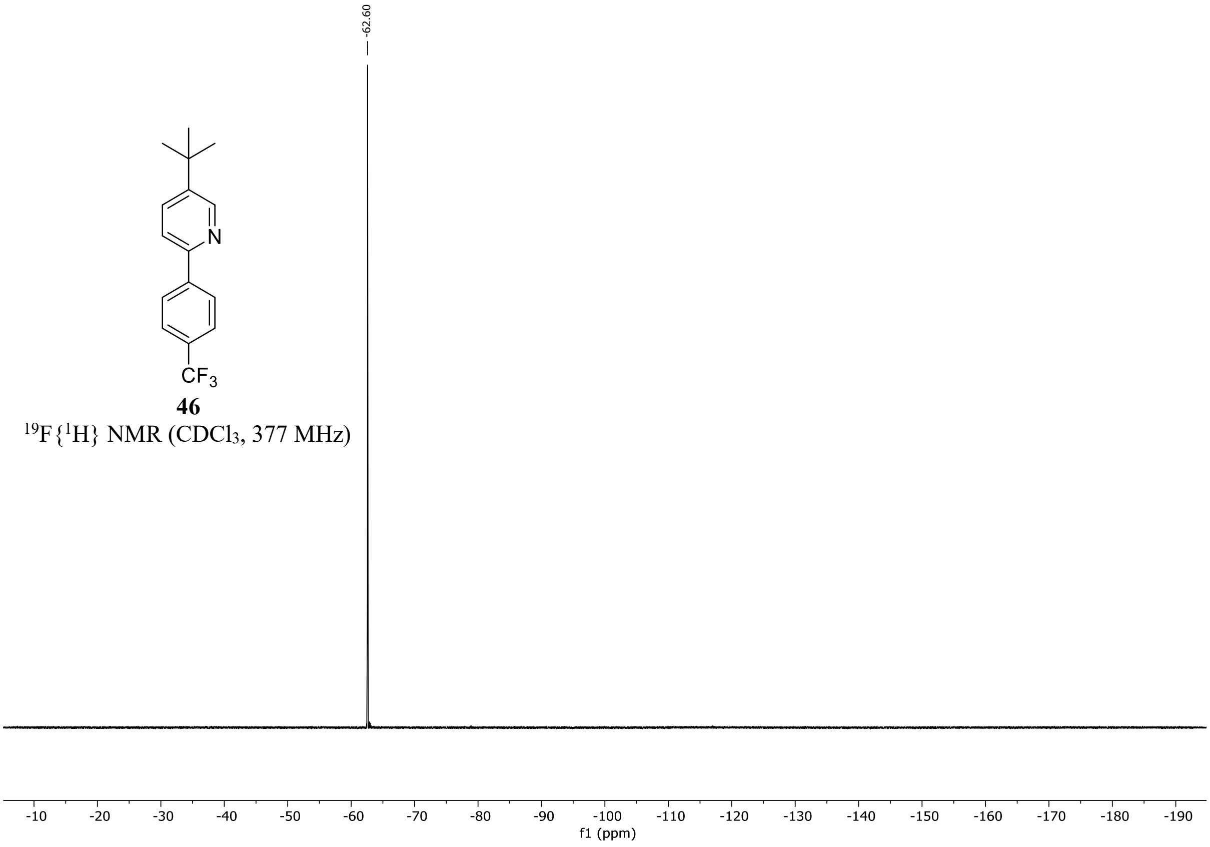

-62.60
N
CF3
46
$^{19}F\{^{1}H\}$ NMR ($CDCl_3$, 377 MHz)
-10
-20
-30
-40
-50
-60
-70
-80
-90
-100
-110
-120
-130
-140
-150
-160
-170
-180
-190
f1 (ppm)

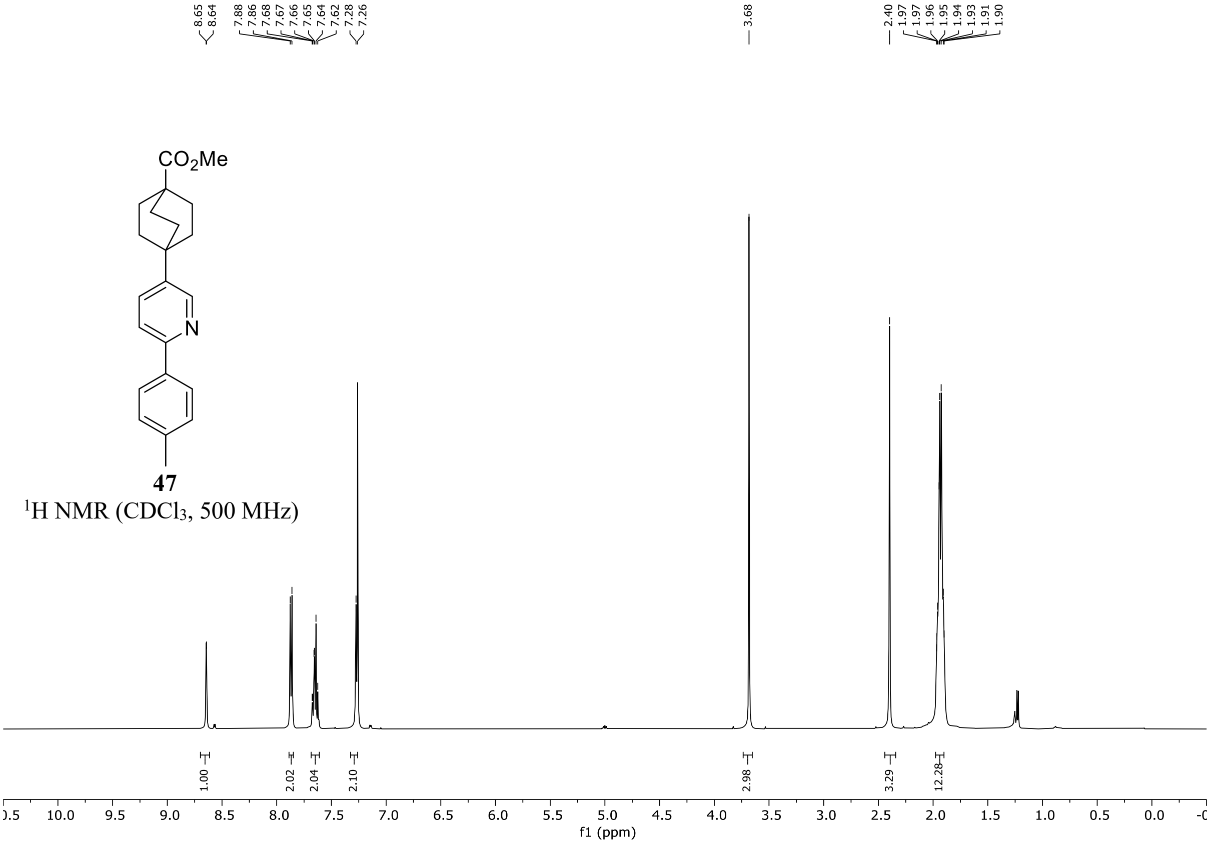

47

^{1}H NMR ($CDCl_3$, 500 MHz)

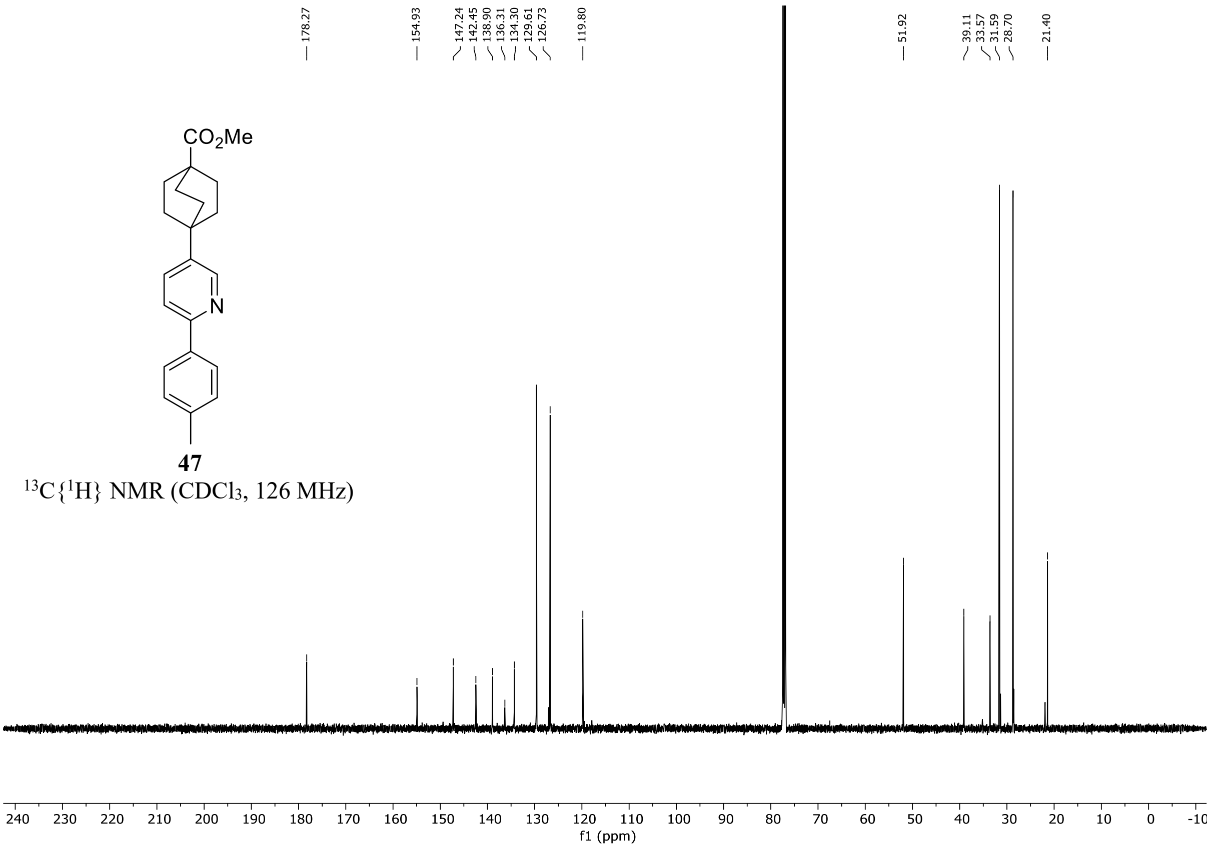

47

$^{13}C\{^{1}H\}$ NMR ($CDCl_3$, 126 MHz)

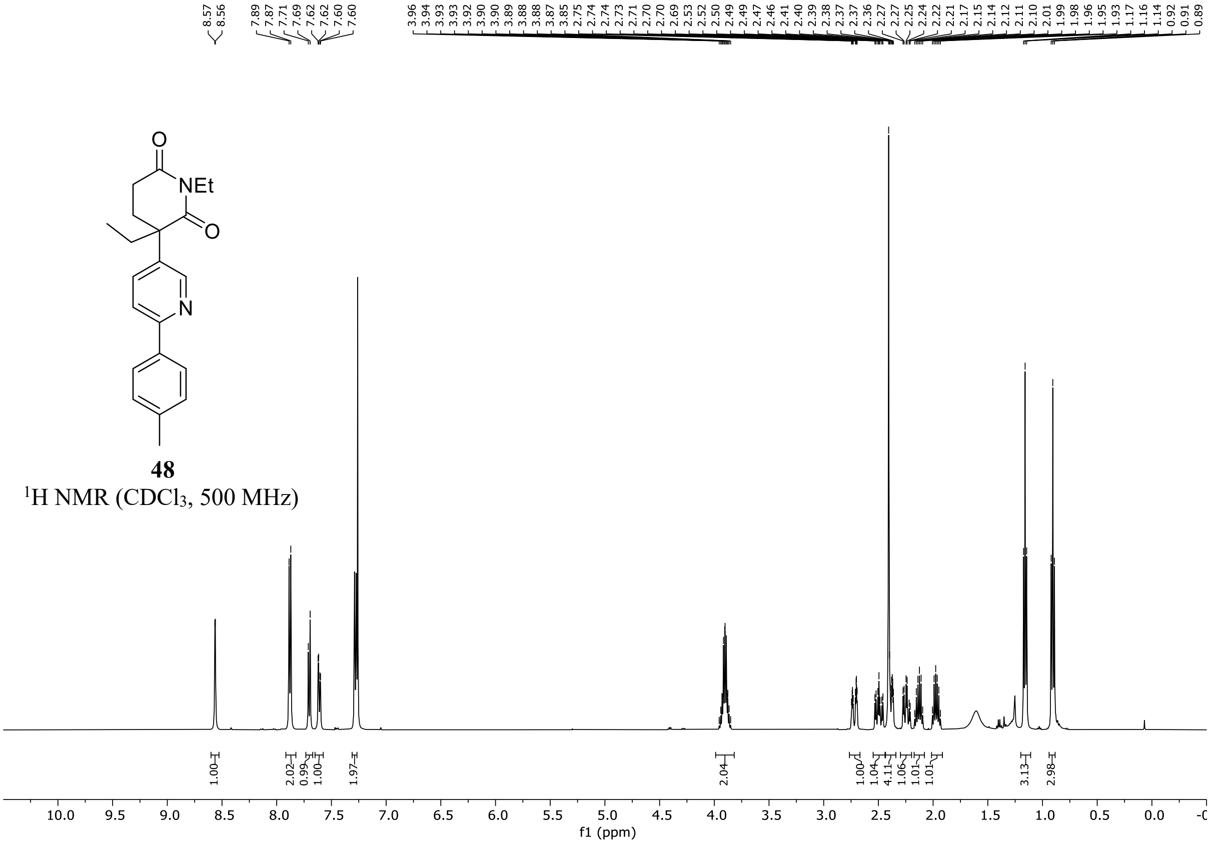

48
^{1}H NMR ($CDCl_3$, 500 MHz)

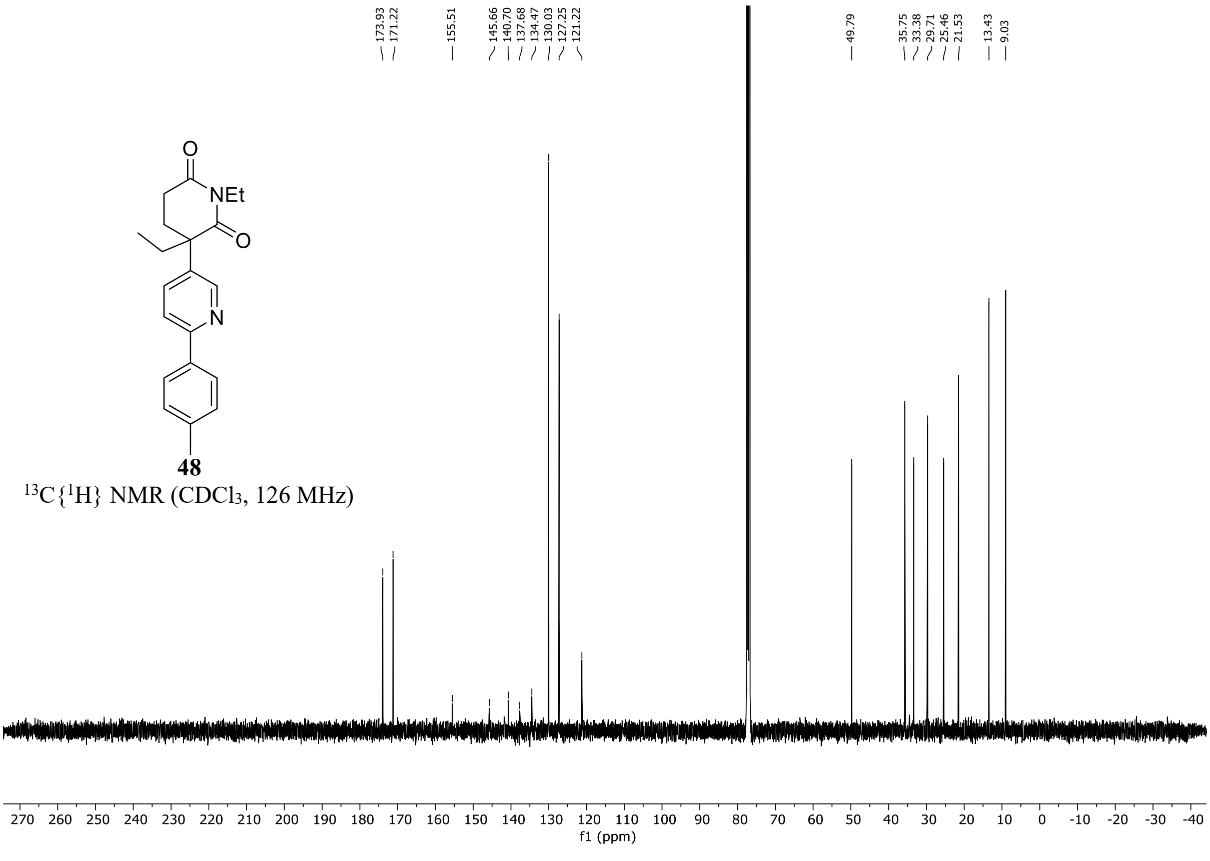

48
$^{13}C\{^1H\}$ NMR ($CDCl_3$, 126 MHz)

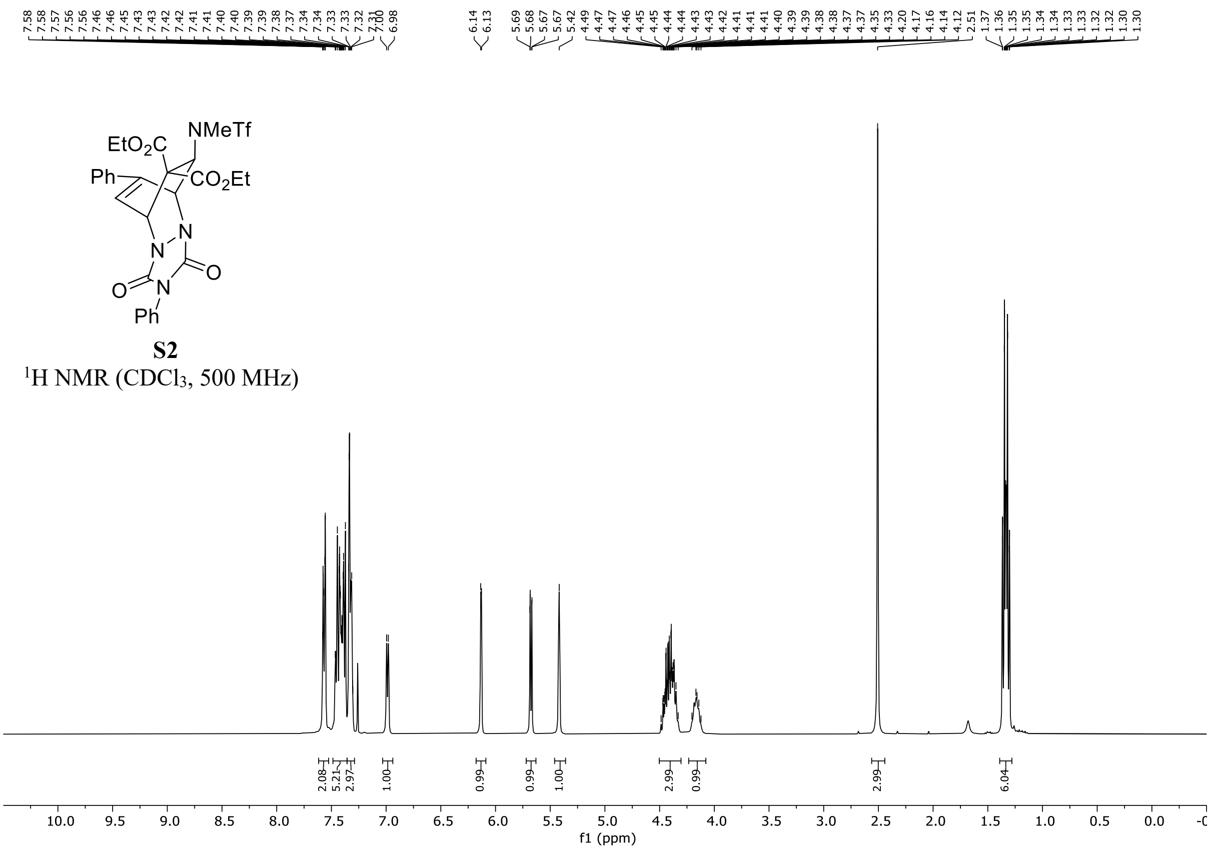

S2

^{1}H NMR ($CDCl_3$, 500 MHz)

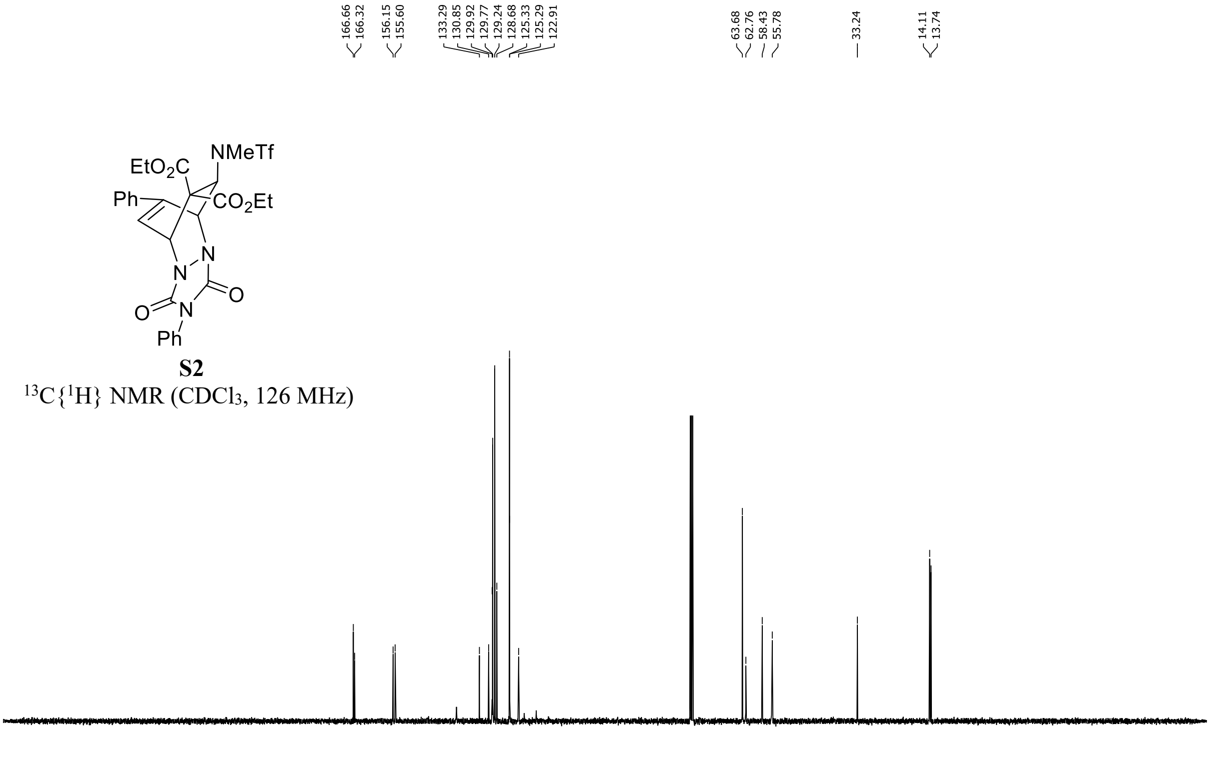

S2

$^{13}C\{^{1}H\}$ NMR ($CDCl_3$, 126 MHz)

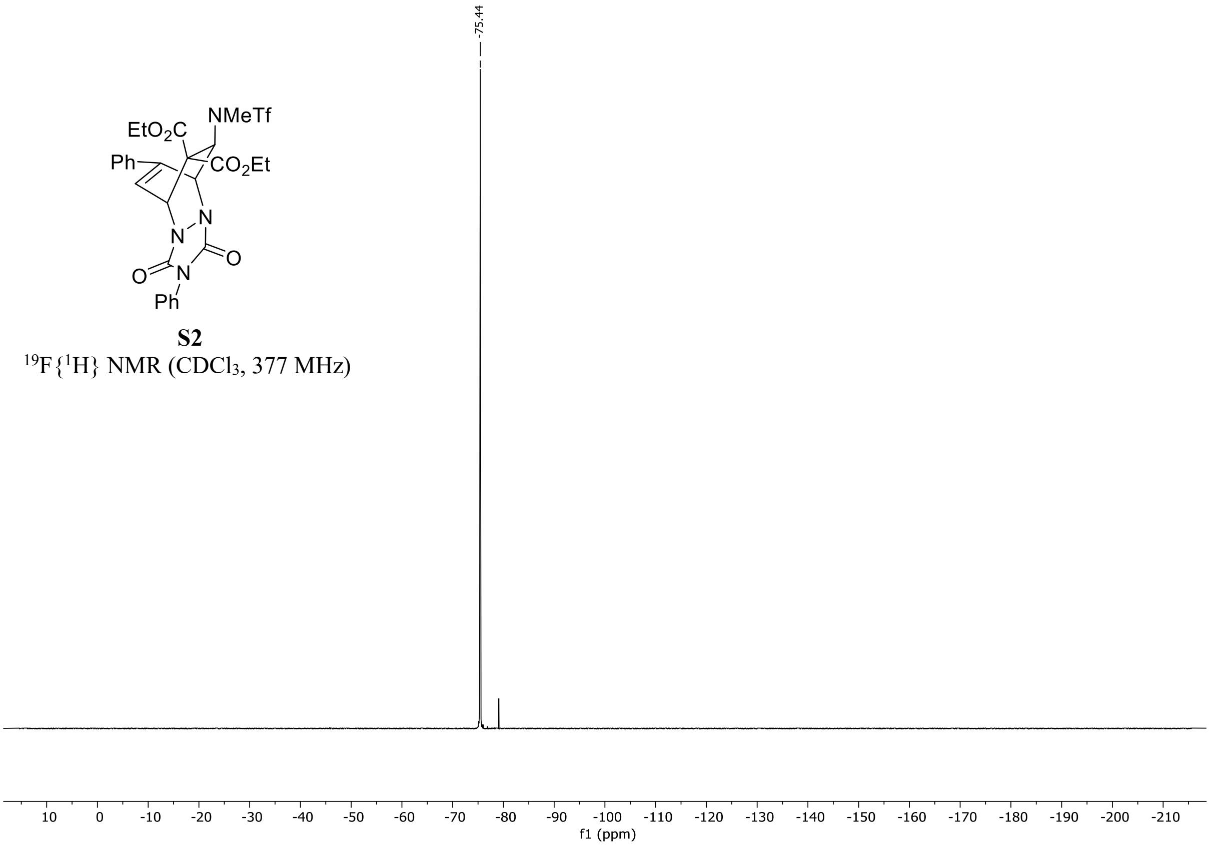

S2

$^{19}F\{^1H\}$ NMR ($CDCl_3$, 377 MHz)

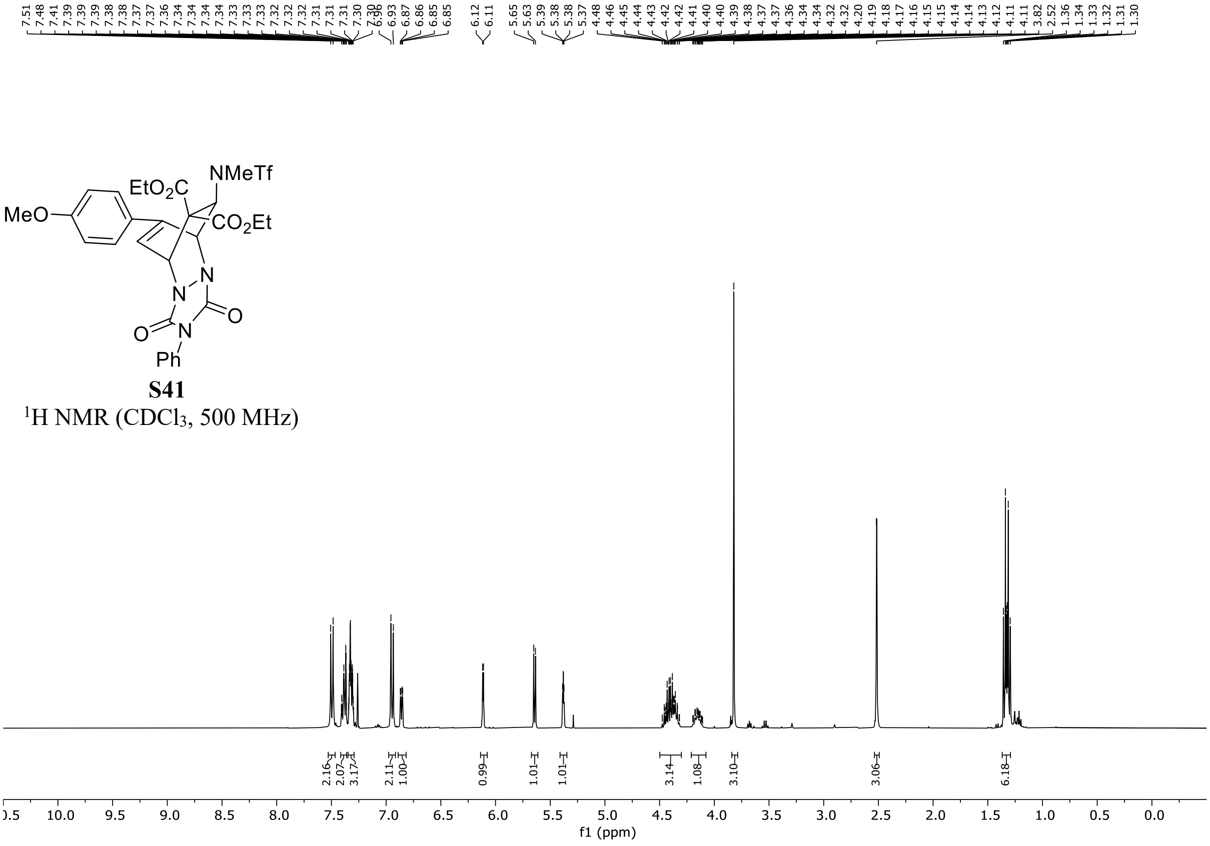

S41

^{1}H NMR (CDCl$_3$, 500 MHz)

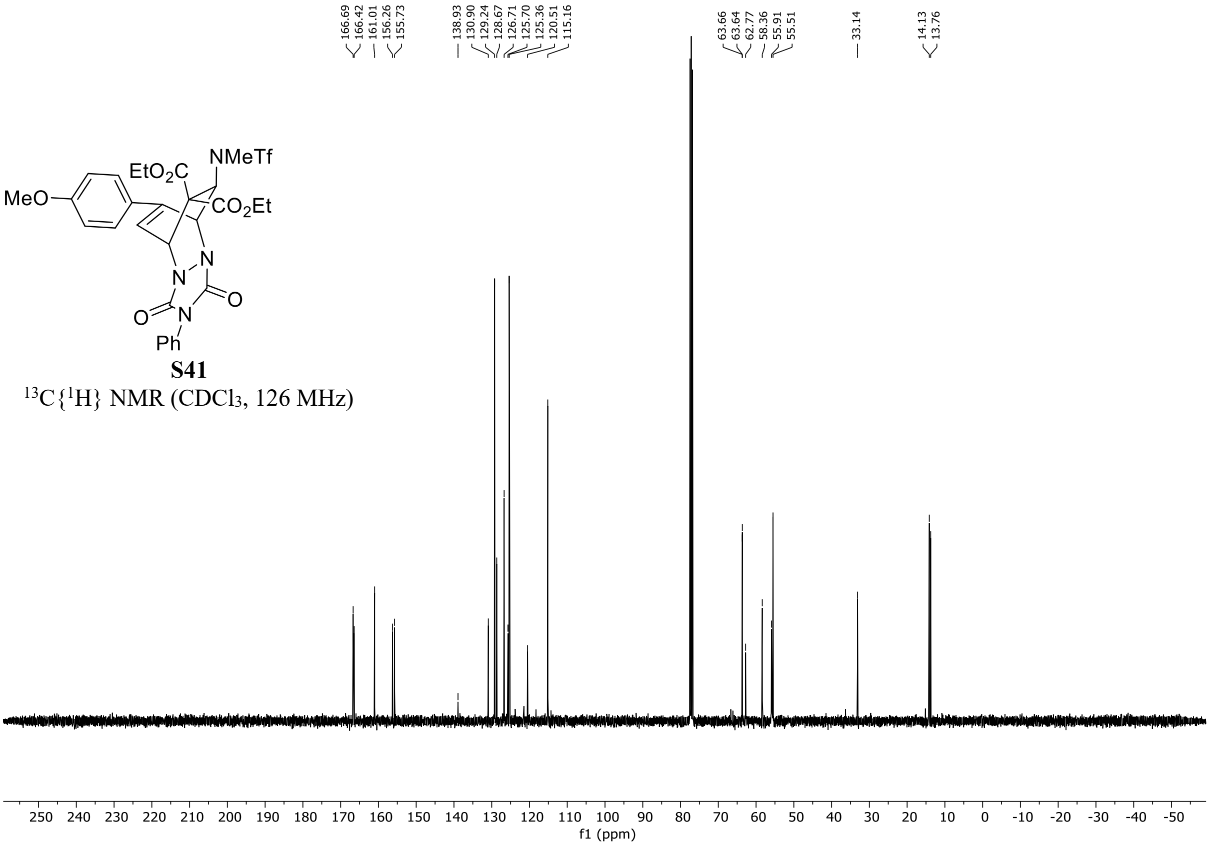

S41

^{13}C{^{1}H} NMR (CDCl$_3$, 126 MHz)

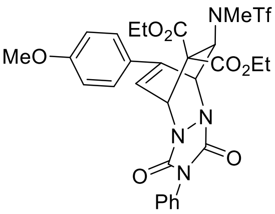

S41

$^{19}F\{^{1}H\}$ NMR ($CDCl_3$, 377 MHz)

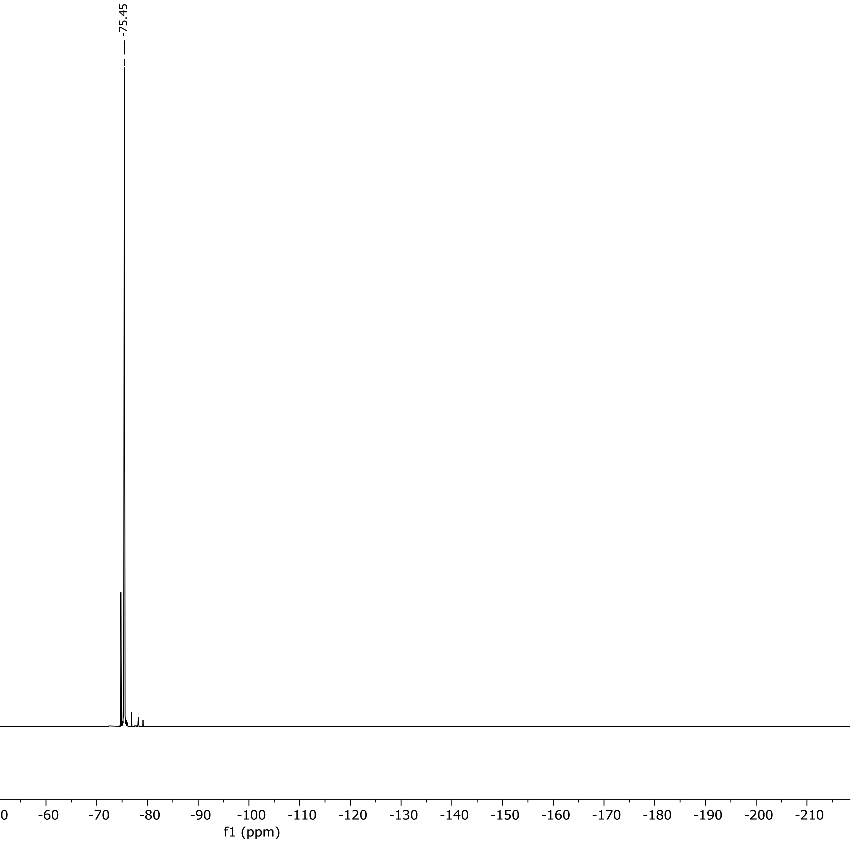

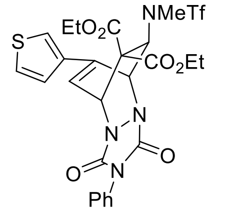

S42

^1H NMR ($CDCl_3$, 500 MHz)

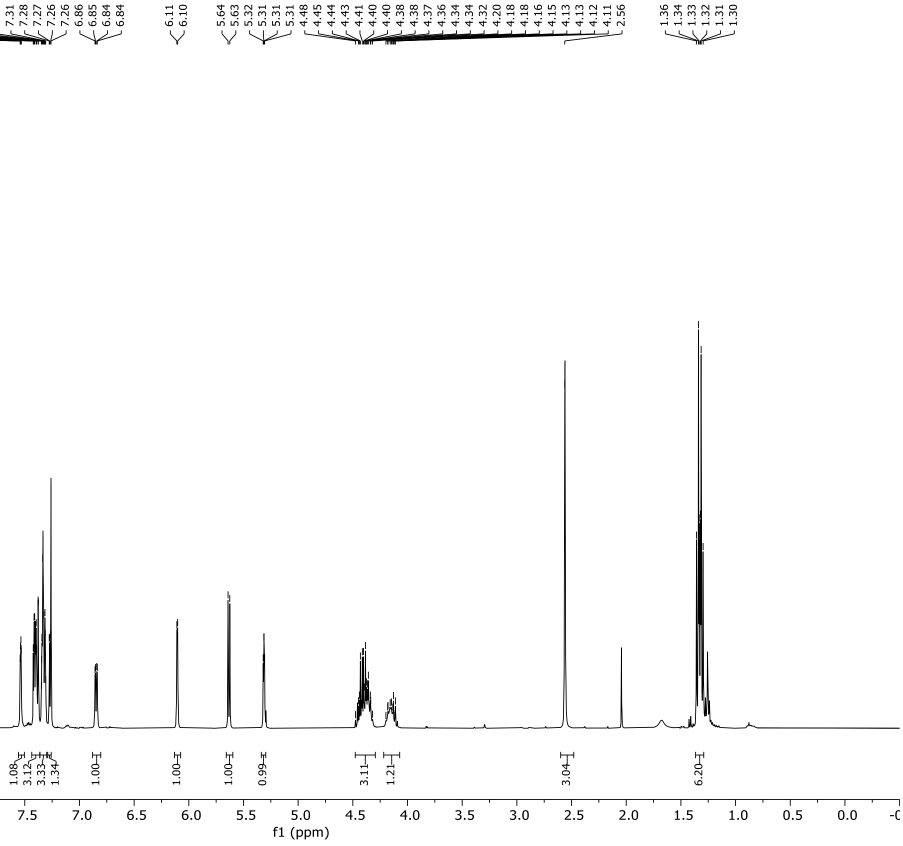

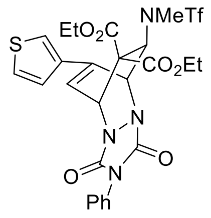

S42

$^{13}C\{^1H\}$ NMR ($CDCl_3$, 126 MHz)

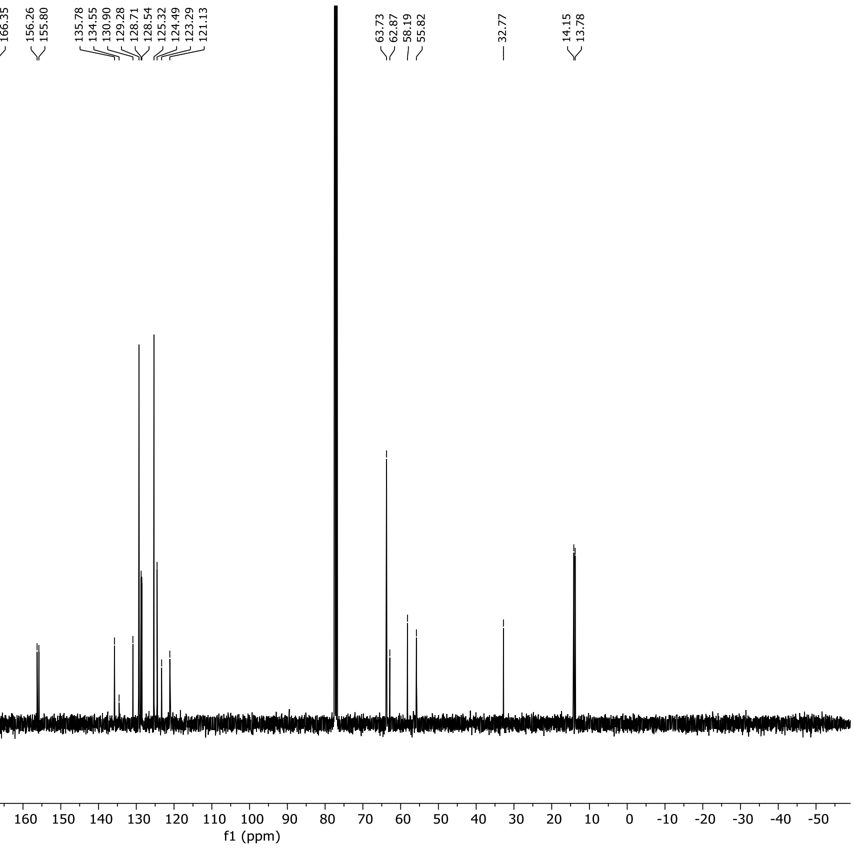

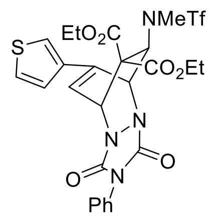

S42

$^{19}F\{^1H\}$ NMR ($CDCl_3$, 377 MHz)

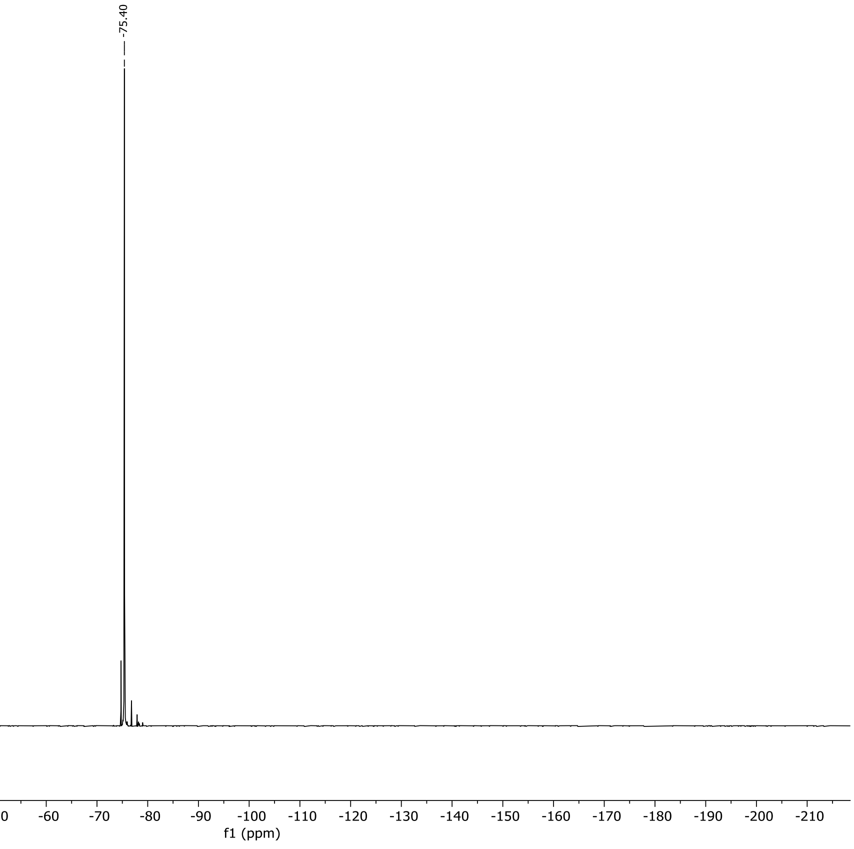

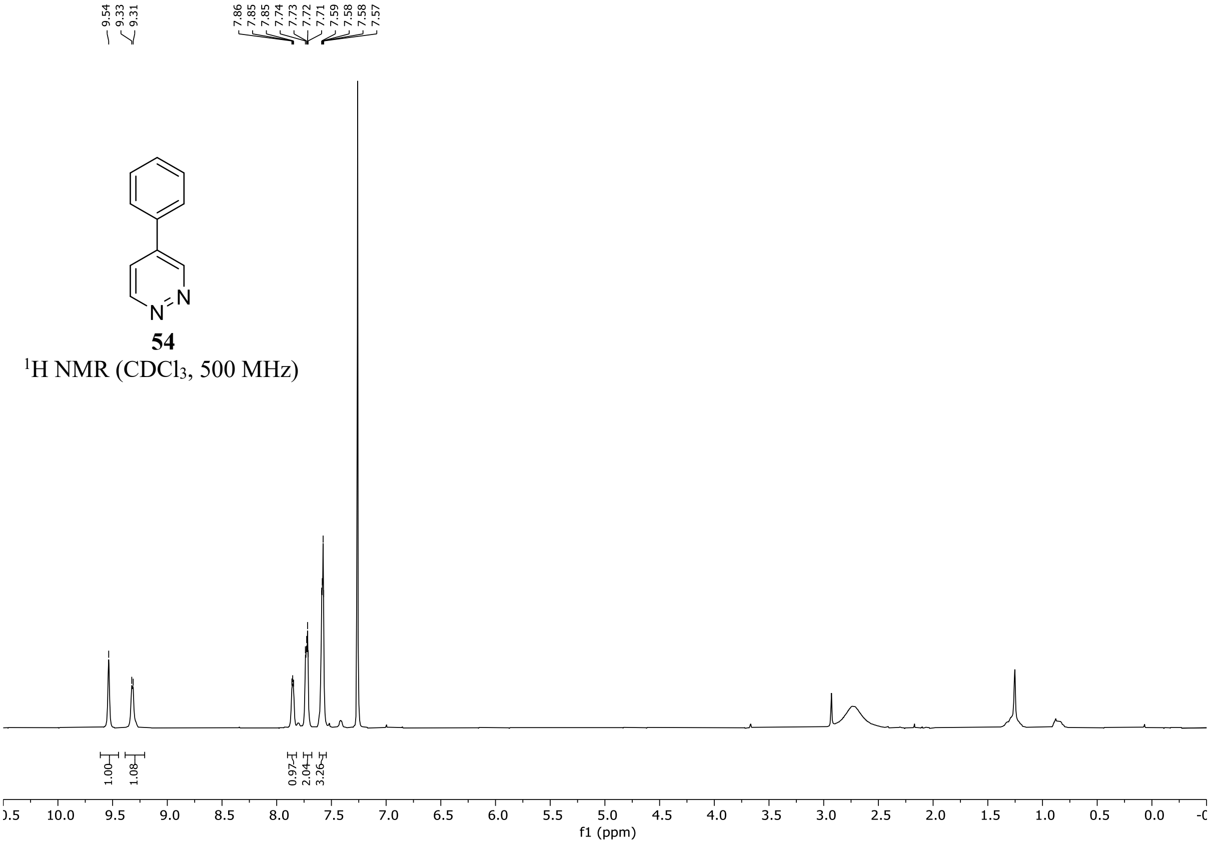

54
^{1}H NMR ($CDCl_3$, 500 MHz)

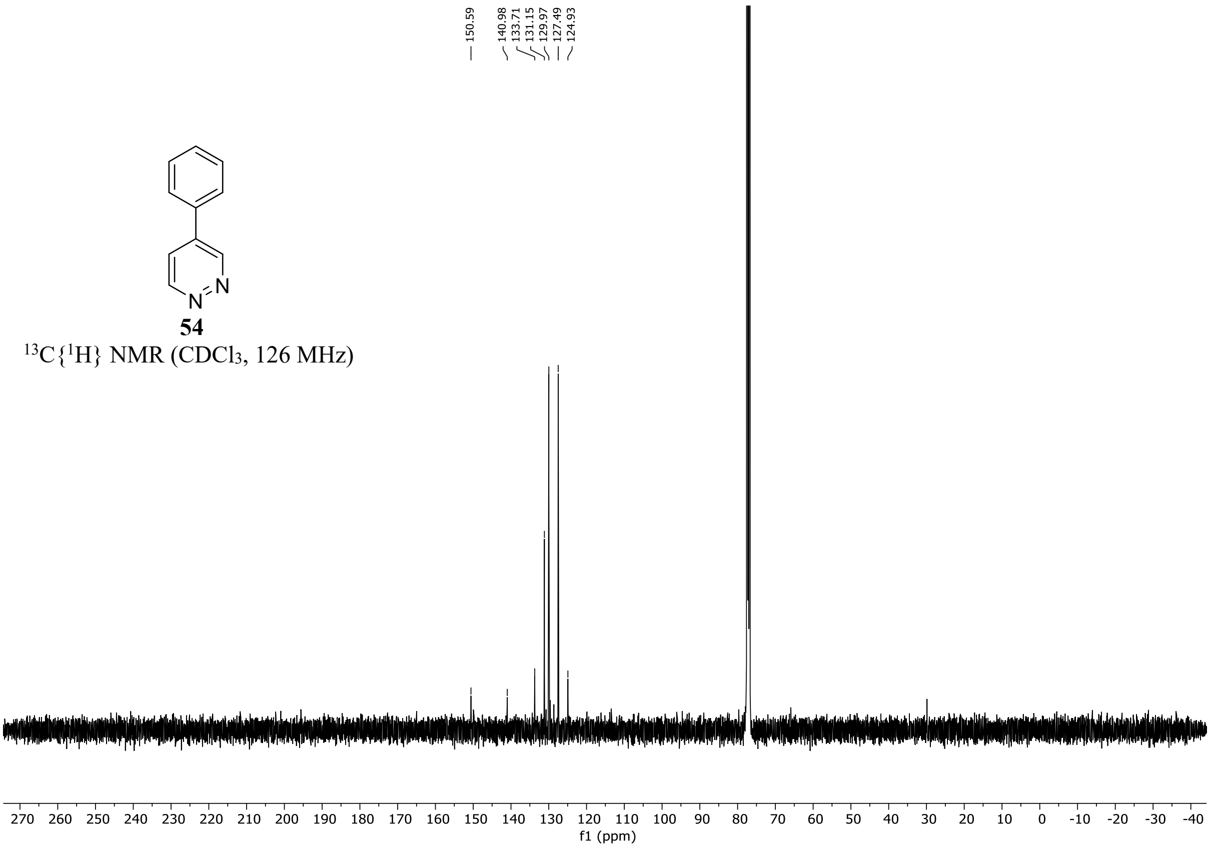

54
$^{13}C\{^{1}H\}$ NMR ($CDCl_3$, 126 MHz)

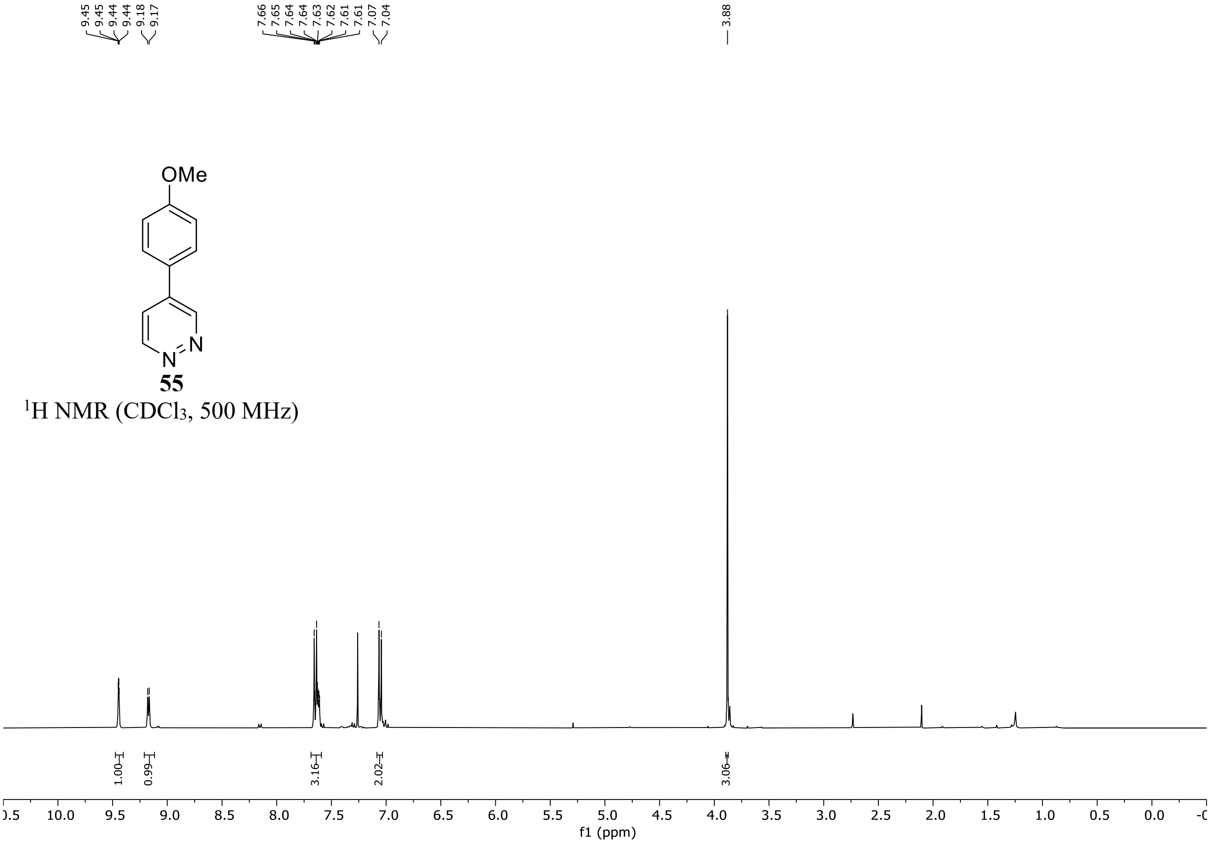

55

^{1}H NMR ($CDCl_3$, 500 MHz)

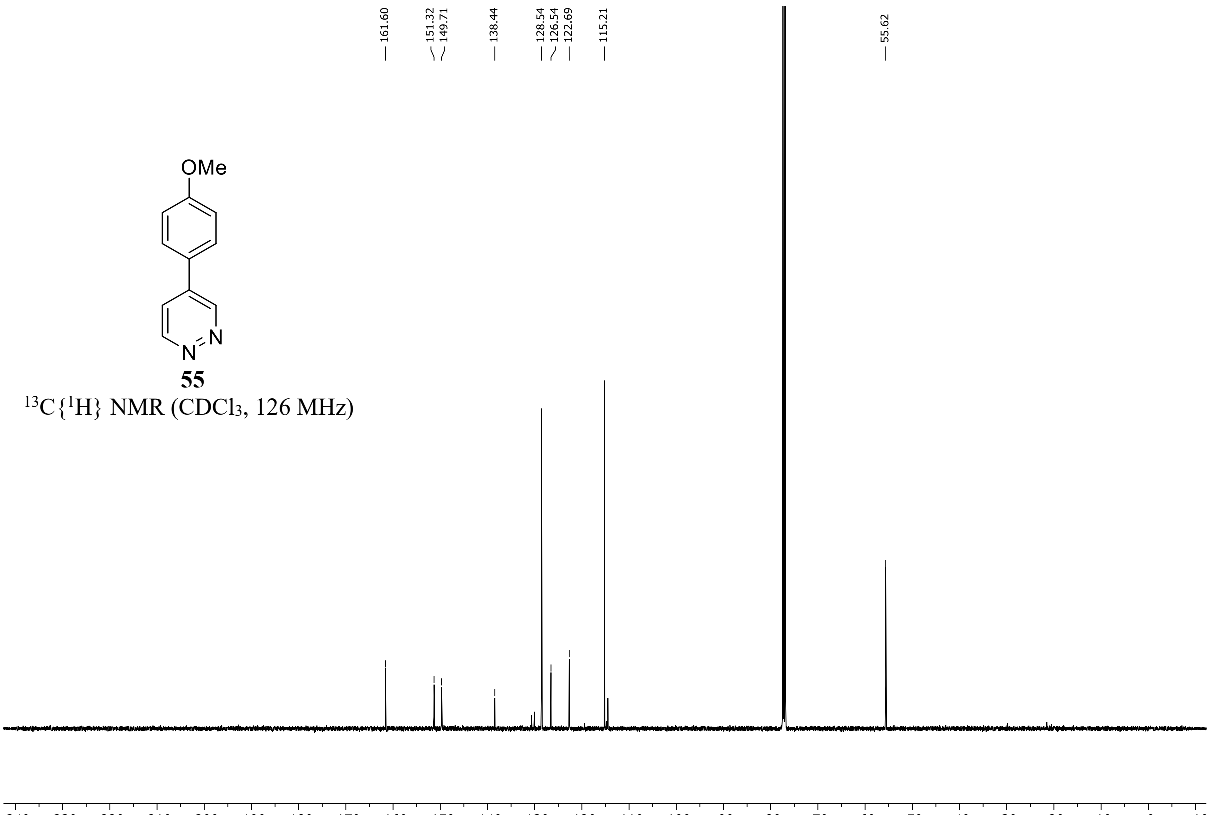

55

$^{13}C\{^{1}H\}$ NMR ($CDCl_3$, 126 MHz)

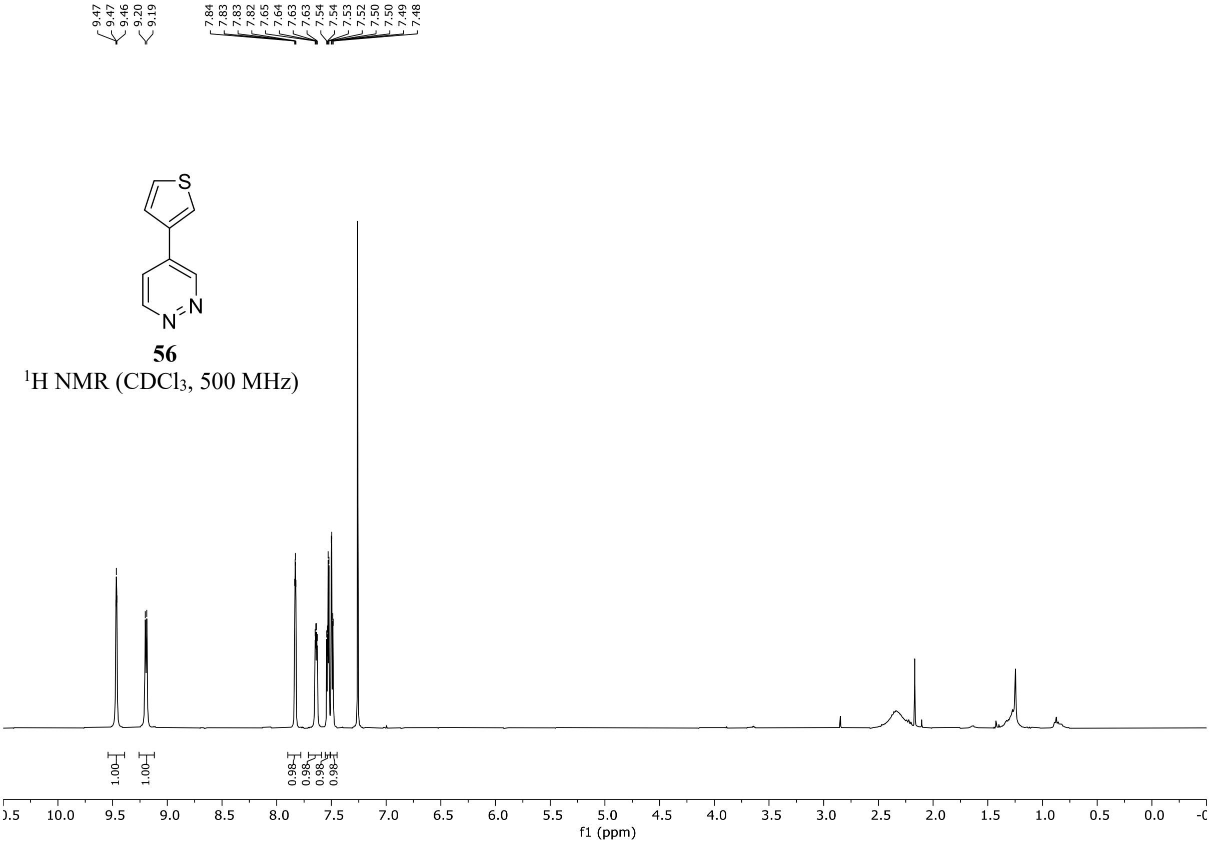

56
^{1}H NMR ($CDCl_3$, 500 MHz)

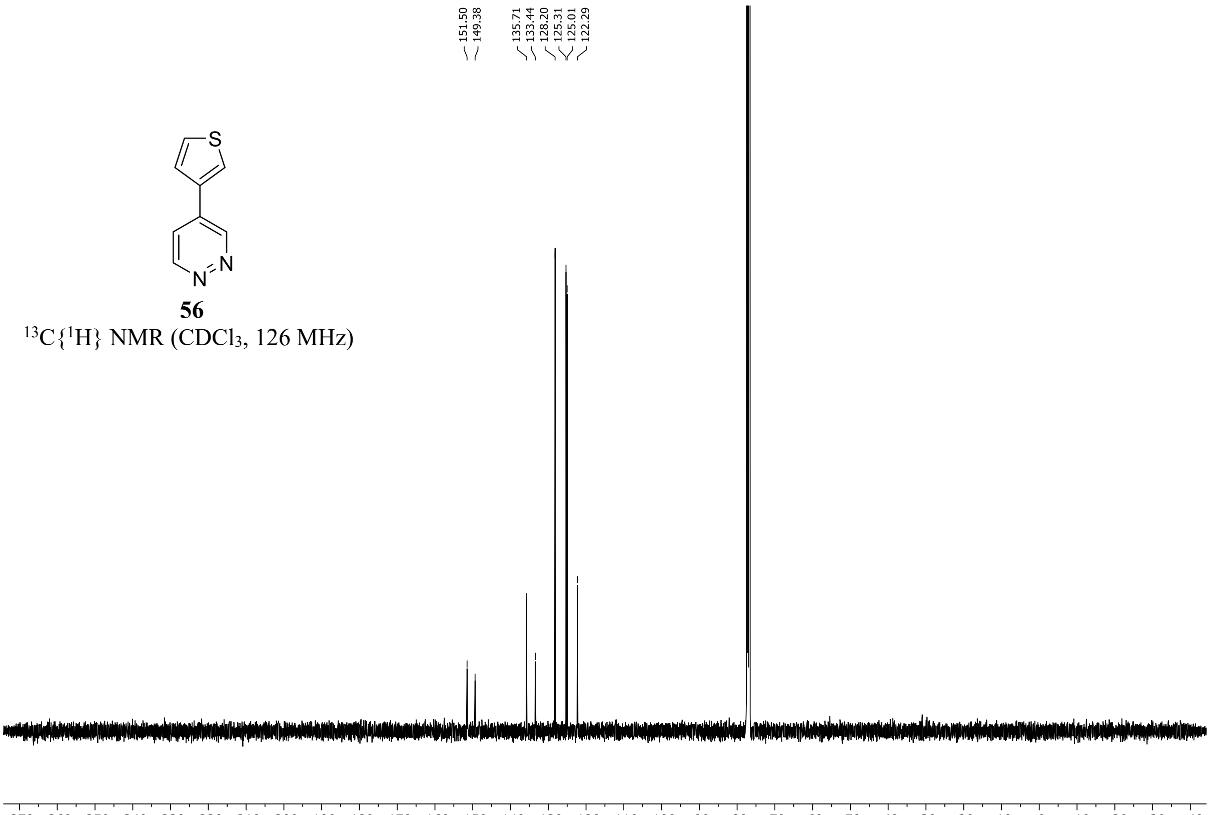

56
$^{13}C\{^{1}H\}$ NMR ($CDCl_3$, 126 MHz)
